# Assessing Patient Adherence to and Engagement With Digital Interventions for Depression in Clinical Trials: Systematic Literature Review

**DOI:** 10.2196/43727

**Published:** 2023-08-11

**Authors:** Ainslie Forbes, Madeline Rose Keleher, Michael Venditto, Faith DiBiasi

**Affiliations:** 1 Otsuka Pharmaceutical Development & Commercialization, Inc Princeton, NJ United States; 2 Oxford PharmaGenesis Inc Newtown, PA United States

**Keywords:** digital therapeutics, digital interventions, digital health, mobile health, mobile phone, depression, major depressive disorder, engagement, adherence, systematic literature review

## Abstract

**Background:**

New approaches to the treatment of depression are necessary for patients who do not respond to current treatments or lack access to them because of barriers such as cost, stigma, and provider shortage. Digital interventions for depression are promising; however, low patient engagement could limit their effectiveness.

**Objective:**

This systematic literature review (SLR) assessed how participant adherence to and engagement with digital interventions for depression have been measured in the published literature, what levels of adherence and engagement have been reported, and whether higher adherence and increased engagement are linked to increased efficacy.

**Methods:**

We focused on a participant population of adults (aged ≥18 years) with depression or major depressive disorder as the primary diagnosis and included clinical trials, feasibility studies, and pilot studies of digital interventions for treating depression, such as digital therapeutics. We screened 756 unique records from Ovid MEDLINE, Embase, and Cochrane published between January 1, 2000, and April 15, 2022; extracted data from and appraised the 94 studies meeting the inclusion criteria; and performed a primarily descriptive analysis. Otsuka Pharmaceutical Development & Commercialization, Inc (Princeton, New Jersey, United States) funded this study.

**Results:**

This SLR encompassed results from 20,111 participants in studies using 47 unique web-based interventions (an additional 10 web-based interventions were not described by name), 15 mobile app interventions, 5 app-based interventions that are also accessible via the web, and 1 CD-ROM. Adherence was most often measured as the percentage of participants who completed all available modules. Less than half (44.2%) of the participants completed all the modules; however, the average dose received was 60.7% of the available modules. Although engagement with digital interventions was measured differently in different studies, it was most commonly measured as the number of modules completed, the mean of which was 6.4 (means ranged from 1.0 to 19.7) modules. The mean amount of time participants engaged with the interventions was 3.9 (means ranged from 0.7 to 8.4) hours. Most studies of web-based (34/45, 76%) and app-based (8/9, 89%) interventions found that the intervention group had substantially greater improvement for at least 1 outcome than the control group (eg, care as usual, waitlist, or active control). Of the 14 studies that investigated the relationship between engagement and efficacy, 9 (64%) found that increased engagement with digital interventions was significantly associated with improved participant outcomes. The limitations of this SLR include publication bias, which may overstate engagement and efficacy, and low participant diversity, which reduces the generalizability.

**Conclusions:**

Patient adherence to and engagement with digital interventions for depression have been reported in the literature using various metrics. Arriving at more standardized ways of reporting adherence and engagement would enable more effective comparisons across different digital interventions, studies, and populations.

## Introduction

### Background

Depression has a substantial global humanistic burden [1] and is associated with lower academic performance [2,3], reduced adherence to treatments for other medical conditions [4], impaired quality of life [5,6], mental and somatic comorbidities [7], and premature death [1]. Worldwide, 279.6 million (5%) adults had depression in 2019 [8,9], including 19.4 million adults in the United States who experienced a major depressive episode [10]. Depression prevalence in the United States increased more than 3-fold during the COVID-19 pandemic; individuals with lower income and greater exposure to stress have had an even higher risk of depression during the pandemic [11].

In addition to the high humanistic costs, depression has considerable economic costs. Compared with people without depression, those with depression have higher excess direct medical costs and indirect costs of lower productivity (absence from work and not fully functioning when at work) [12,13]. In the United States, major depressive disorder (MDD) cost >US $326 billion and afflicted 17.5 million (7.1%) adults in 2018 [13]. Patients with MDD incur even higher medical costs and health care resource use when they experience relapse or recurrence [14].

Approximately 1 in 3 adults with MDD in the United States does not receive treatment [15]. Many obstacles prevent people from receiving treatment, including cost [16], a shortage of mental health care providers [16], an uneven distribution of providers [16,17], long wait times for appointments [18], side effects of medication [19], and stigma about mental illness [16].

Furthermore, among patients who do receive treatment, approximately half do not adhere to antidepressants for MDD [20], and between 40% and 60% of patients with depression do not respond to first-line antidepressant treatment (ADT) with adequate symptom relief [21,22]. Similarly, dropout is common in psychotherapy for depression [23,24], and approximately half of the patients with depression across 5 trials did not respond to cognitive behavioral therapy [25]. These patients have a heightened risk of impaired functioning, lower quality of life, comorbid conditions, and suicidal behavior [26-28]. Given the barriers to access and the limited response to existing treatments, there is an unmet need for new and more personalized treatments for depression [29-31].

Digital health technologies may help address many of the treatment needs of patients with depression [32,33]. These tools offer increased access to treatment (including asynchronous access outside the clinic) [34-36], can reduce stigma concerns [35], and have the potential to be cost-effective [34,37,38]. Digital technologies such as chatbots, telehealth, smartphone apps, and virtual reality are now more widely used in mental health care than before the COVID-19 pandemic [32,34,39]. Smartphones are being used to deliver on-demand mental health interventions based on cognitive and behavioral therapies [32]. The most popular depression and anxiety apps average 10,000 downloads each month, with thousands of daily users [40]. However, the effect of apps may be limited if users do not sustain engagement [41]. Indeed, not all apps are used for long; in general, 65% of people who download a smartphone app stop using it within 1 week [42]. Furthermore, not all apps are used equally; a study of the top 50 hits for depression apps and the top 50 hits for anxiety apps in the Google app store found that 6 apps accounted for 90% of active use, whereas most of the remaining apps had no monthly active users [40]. User engagement data from various studies indicate challenges with initial and sustained engagement [43-45]. For example, 1 real-world study of 12 depression and anxiety apps found that after downloading the apps, approximately two-thirds of the users stopped engaging within 1 week, with half of the users discontinuing within 1 day [43]. Another real-world study of 93 Android mental health apps found that only 3.9% of the users engaged with the apps 15 days later, and only 3.3% of the users sustained engagement on day 30 [44].

To design mental health apps that people will use, it is critical to understand how and why the most successful apps sustain user engagement [32,40,46]. Six core design principles have been identified by the professional services network PwC as essential to the success of these technologies: (1) integrating into the care and lives of patients; (2) fitting in with other relevant systems, such as health records and software; (3) delivering data to patients and providers in an intelligent and actionable way; (4) connecting with patients, providers, and payers; (5) measuring efficacy outcomes; and (6) being engaging so that patients use the technology regularly [47]. A recent analysis of 1000 digital health apps indicated that only 17.8% (n=178) had been studied scientifically, and only 5.6% (n=56) satisfied the criteria for being engaging (by incorporating gamification into the app design), with a mere 0.4% (n=4) that met all 6 aforementioned design principles [48]. There may be a difference in engagement and efficacy between a specific category of digital health tools called digital therapeutics (DTx) and other categories of digital health tools, although this is yet to be determined. DTx are evidence-based interventions delivered by high-quality software intended to prevent, manage, or treat specific diseases [37,49,50]. Unlike many currently available health and wellness apps, DTx must submit efficacy and safety data to be reviewed or cleared by regulatory bodies before reaching consumers [37,50,51]. This manuscript focuses on evidence-based digital interventions, rather than the broader pool of digital health tools.

### Adherence and Engagement

To ultimately improve mental health apps, it is important to first select meaningful criteria for measuring adherence and engagement. However, there is no universally accepted definition for either term; both are used interchangeably in the literature, and each word has been used when defining the other [46,52-55]. Li et al [56] defined engagement as “the degree to which a patient adheres to an intervention,” citing Christensen et al [57]. However, rather than engagement, Christensen et al [57] defined adherence as “the extent to which individuals experience the content of the Internet intervention.” Flett et al [58], also citing Christensen et al [57], defined adherence as “whether individuals access the content and use it in the manner it was designed to be optimally effective.” In a systematic literature review (SLR) of the definitions of adherence to eHealth technology, Sieverink et al [55] concluded that the term adherence is often incorrectly applied to variables that simply measure the amount of use of a technology, arguing that a true metric of adherence requires 3 components: use, intended use, and a justification for how the level of intended use was determined. Only 8% of the studies they reviewed included all 3 components, whereas 37% included the first 2 components [55]. For this SLR, we used the latter, less stringent definition of adherence but included only the first 2 components—actual use and recommended use—because very few studies reported the third component justifying the level of recommended use (Textbox 1).

Similar to adherence, engagement has also been conceptualized in several ways. In human-computer interaction research, it has been interpreted as having cognitive (eg, attention and effort), emotional (eg, feeling interested or bored), and behavioral (eg, participation and action) dimensions [59]. In this SLR, we use a behavioral conceptualization of engagement, defining it as the extent to which patients interacted with an intervention (eg, number of hours used, modules used, log-ins, and days used).

Few studies have investigated the association between user adherence or engagement and the efficacy of digital interventions [60]; however, these studies suggest a complicated relationship. Some studies found a dose-response relationship (where users with higher engagement with the technology achieved better outcomes) [61,62], whereas others either found no such relationship [53,63,64] or indicated that users can be impacted by interventions that they do not fully complete [55]. To gain insight into user adherence and engagement and—where possible—their relationship with efficacy, we conducted an SLR of clinical trials, feasibility studies, and pilot studies of digital interventions for depression delivered via mobile apps, the web, or both. We sought to better understand the levels of adherence and engagement reported in the literature and how these are affected by factors such as the method of intervention delivery, access to psychotherapy, and the reception of human support with delivery. By synthesizing this information, we aimed to support research on digital interventions by identifying ways of standardizing the collection and reporting of adherence and engagement data.

**Key terms used in this systematic literature review.**

**Adherence**

- Actual digital intervention use compared with intended use (ie, the average number of modules used divided by the total number of modules available)

**Engagement**

- To which extent patients interact with an intervention (eg, number of hours used, modules used, log-ins, and days used)

**Care as usual**

- Unrestricted access to psychiatrists, medication, psychotherapy, and primary care physicians

**Active control group**

- Use of a depression treatment other than the digital intervention under investigation (eg, in-person therapy or web-base progressive muscle relaxation)

## Methods

### Searches

Our search covered interventions, therapies, and cognitive trainings that were computer assisted, internet assisted, smartphone based, or digital in some other way; designed to treat MDD or depression; and reported engagement outcomes. We conducted a search for articles published between January 1, 2000, and April 15, 2022, in Ovid MEDLINE In-Process & Other Non-Indexed Citations, Embase, and the Cochrane Library (CDSR, DARE, CENTRAL, CMR, and NHS EED) using the search string in Textbox 2. We also included references cited by relevant review articles [44,57,65-68]. The decision to exclude studies published before 2000 was made because digital therapies for depression were rare before this year. The first round of screening was of the titles and abstracts, and the second round of screening was of the full text. Multiple people were involved in the screening, with each article being screened by 1 person at each round. In cases where a reviewer was uncertain whether an article should be included or excluded, 2 other reviewers were consulted, and consensus was reached. We used DistillerSR (version 2.39.0; DistillerSR Inc) to deduplicate the searches and help coordinate the screening and extraction.

**Ovid search string used to conduct this systematic literature review.**

(Cognitive behavio?r therapy technology OR Cognitive remediation technology OR Cognitive training technology OR Computer-assisted cognitive behavio?r therapy OR Computer-assisted cognitive remediation OR Computer-assisted cognitive training OR Computer-assisted intervention OR Computer-assisted psychotherapy OR Computer-assisted therapy OR Computerized cognitive behavio?r therapy OR Computerized cognitive remediation OR Computerized cognitive training OR Computerized intervention OR Computerized psychotherapy OR Computerized therapy OR Digital care OR Digital cognitive behavio?r therapy OR Digital cognitive remediation OR Digital cognitive training OR Digital health intervention OR Digital intervention OR Digital psychotherapy OR Digital therapy OR Digital therapeutic OR Internet cognitive behavio?r therapy OR Internet cognitive remediation OR Internet cognitive training OR Internet intervention OR Internet psychotherapy OR Internet therapy OR Internet-assisted cognitive behavio?r therapy OR Internet-assisted cognitive remediation OR Internet-assisted cognitive training OR Internet-assisted intervention OR Internet-assisted psychotherapy OR Internet-assisted therapy OR Mobile cognitive behavio?r therapy OR Mobile cognitive remediation OR Mobile cognitive training OR Mobile health application OR Mobile health intervention OR Mobile intervention OR Mobile psychotherapy OR Mobile therapy OR Online cognitive behavio?r therapy OR Online cognitive remediation OR Online cognitive training OR Online intervention OR Online psychotherapy OR Online therapy OR Psychotherapy technology OR Smartphone health application OR Smartphone health intervention OR Smartphone intervention OR Smartphone psychotherapy OR Smartphone therapy OR Therapy technology OR Web-based cognitive behavio?r therapy OR Web-based cognitive remediation OR Web-based cognitive training OR Web-based intervention OR Web-based psychotherapy OR Web-based therapy) AND (Major Depressive Disorder OR Depression) AND (Adherence OR Compliance OR Engagement OR Completion OR Complete OR Finish OR Download OR Log in OR Sign in OR Visit OR View OR Time)

### Study Selection Criteria

We included studies that were of adults with depression or MDD as the primary diagnosis; were written in English; and described the results of randomized controlled trials (RCTs), pilot studies, or feasibility studies of digital interventions to treat depression, such as DTx and mobile mental health apps. Studies using medication- or telemedicine-based interventions were excluded, as were those lacking the evaluation of participant adherence or engagement outcomes. Full inclusion and exclusion criteria are described in Table 1, and the PRISMA (Preferred Reporting Items for Systematic Reviews and Meta-Analyses) checklist can be found in Multimedia Appendix 1. The review was not registered, and a separate protocol was not prepared; all methods are described herein.

**Table 1:** PICOS^a^ criteria for the inclusion and exclusion of studies.

Criteria	Included	Excluded
Population	Adults with MDD^b^ or at least mild depression	Adults with a primary mental health diagnosis other than MDD (eg, bipolar disorder)
Interventions	Digital intervention	Medication Digital systems that include medication (eg, digital medicine) Telemedicine
Comparators	Not restricted	N/A^c^
Outcomes	Adherence metrics defined based on quantifiable data about participants’ engagement with a digital product (which may be referred to as adherence, compliance, or engagement). Examples include the following: number or percentage of participants who competed study or treatment, number or percentage of times participants logged into or started the intervention, duration of use or mean time spent on intervention, number of modules used or activities and assignments completed (either from the total program [if fixed amount] or over the course of the study), number of recommended modules or assignments completed, and number and types of web pages visited within the intervention	Economic outcomes Studies that do not report compliance, adherence, or engagement outcomes or metrics Studies that do not report the effects of digital mental health interventions on adherence, compliance, or engagement
Study designs	Clinical trials (will include RCTs^d^ in addition to non-RCTs, such as pilot or feasibility studies, and protocols)	Nonhuman studies Preclinical studies Short-term studies (study length <10 days) Studies interrupted or prematurely terminated Systematic reviews and meta-analyses Observational studies Real-world studies

^a^PICOS: patients, intervention, comparator, outcomes, study design.

^b^MDD: major depressive disorder.

^c^N/A: not applicable.

^d^RCT: randomized controlled trial.

### Extraction

We extracted data from the articles that passed the full-text screening based on the inclusion and exclusion criteria listed in Table 1. The following information was recorded from each study: (1) study design (RCT, feasibility, or pilot); (2) the primary diagnosis of participants (depression or MDD); (3) the metric used to diagnose depression for inclusion in the study; (4) the number of participants included in the study; (5) participant demographics (age, sex or gender, race, and ethnicity); (6) the type of digital intervention used (web based, app based, both, or CD-ROM); (7) the name of the digital intervention; (8) the number of days participants were allotted to use the intervention; (9) whether the intervention was unguided or delivered with human support; (10) whether other forms of treatment for depression, such as psychotherapy and antidepressants, were permitted during the study period; (11) how care as usual (CAU) was defined; (12) the type of control group used as a comparator to the digital intervention group (active, waitlist, CAU, or none); (13) the adherence and engagement metrics used; (14) the level of adherence and engagement reported; (15) the primary efficacy outcome and any other efficacy outcomes, if reported; and (16) the relationship of adherence and engagement with clinical outcomes, if reported.

We appraised the quality of the included studies using the tool developed by Hawker et al [69], which provides a rubric for assigning a score of “good,” “fair,” “poor,” or “very poor” to the clarity of the abstract, introduction, methods, and results; the sampling strategy; the rigor of the data analysis; the discussion of ethical issues and bias; and the generalizability and usefulness of the study. For each study, the same individual extracted the data and rated the quality of the study, and a second individual performed a quality control check of the extracted data to ensure accuracy.

### Analysis

The analysis in this SLR is primarily descriptive, whereby we calculated the overall averages of the means and median values reported by the studies and visually presented the topline findings. We analyzed the studies as a whole group and as subgroups formed according to the following factors: mode of intervention delivery (web based vs app based), access to psychotherapy (yes vs no), and reception of human support with intervention delivery (yes vs no). Studies were included in any analysis for which they had appropriate data and were excluded from analyses for which they were missing data. Because interventions varied in the number of modules available, we calculated the mean dose received (an adherence metric) by dividing the mean number of modules completed by the total number of available modules for each of the 38 studies in which this information was available. In addition, we calculated the Pearson correlation coefficient for the relationship between the number of hours participants spent engaging with digital interventions and the allotted treatment duration in the studies.

## Results

### Studies Selected

**Figure 1:**
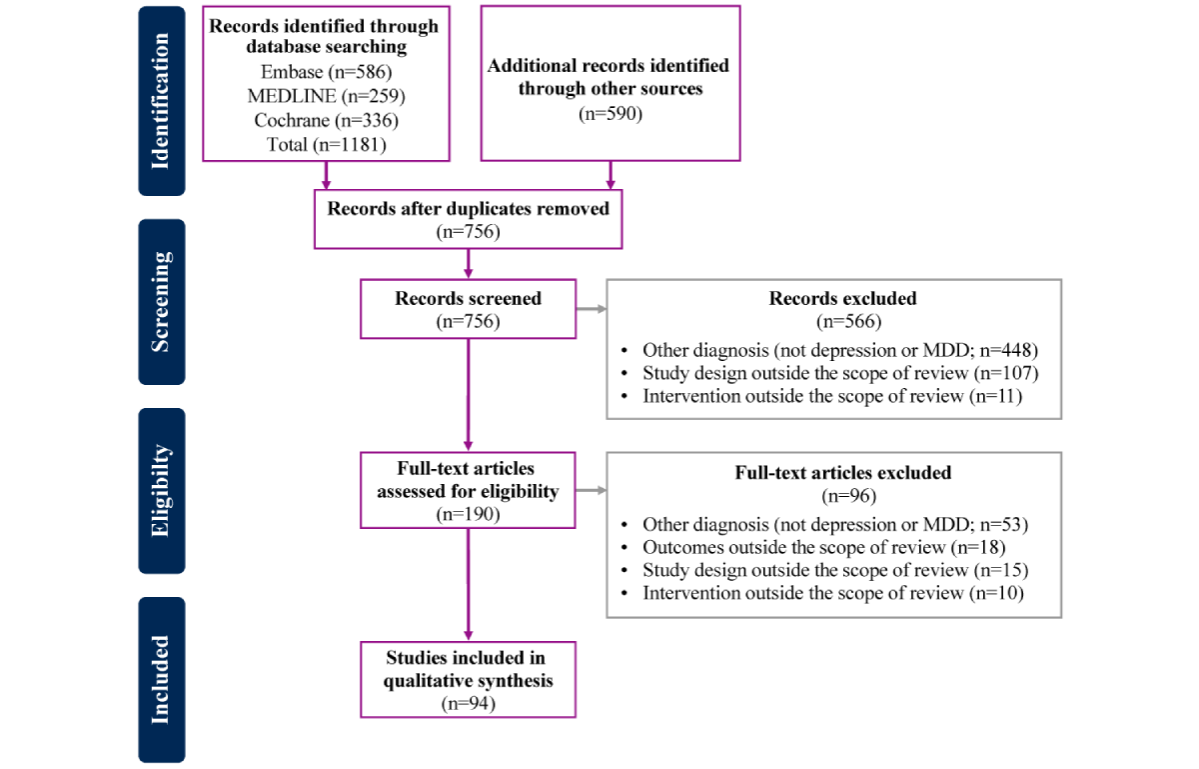
PRISMA (Preferred Reporting Items for Systematic Reviews and Meta-Analyses) diagram. MDD: major depressive disorder.

The search yielded 1181 records, and an additional 590 records were identified by manually searching the reference lists of relevant review articles [57,65-68,70] (Figure 1). After removing the duplicates, 756 records were screened. During abstract screening, we excluded 566 (74.9%) of the 756 records, most often when the study pertained to a diagnosis other than depression (488/756, 64.6%) and when the study design (107/756, 14.2%) or intervention (11/756, 1.5%) was deemed to be outside the scope of this review (Figure 1 and Multimedia Appendix 2). A total of 190 articles underwent a full-text screening for eligibility. At this step of screening, we excluded 27.9% (53/190) of articles that did not have depression as a primary diagnosis, 9.5% (18/190) of articles that did not have engagement outcomes, 7.9% (15/190) of articles that had a study design beyond the scope of this review (eg, SLR, meta-analysis, case study, or study of an adolescent patient population), and 5.3% (10/190) of articles that had an intervention outside the scope of this review (eg, an intervention delivered via the web in real time by a therapist or an intervention to help patients taper off antidepressants; Multimedia Appendix 2). Of 190 studies, we included the remaining 94 (49.5%) studies in this review [61-64,71-161]: 65/94 (69.1%) identified by database search and 29/94 (30.9%) identified from relevant review articles (Multimedia Appendix 3 [61-64,71-161]). Of the 94 studies, 12 (13%) were protocols for clinical trials, and although they did not report results, we included them in this review to gauge how they planned to assess participant engagement and adherence. A summary of the main features of the articles can be found in Multimedia Appendix 4 [61-64,71-85,88-107,109-161].

### Study Characteristics and Types of Digital Interventions

Of the 94 studies included in this SLR, the majority were RCTs (n=69, 73%), 18 (19%) were pilot studies, and 7 (7%) were feasibility studies (Multimedia Appendix 4). The number of studies reporting on adherence to or engagement with digital interventions for depression increased over time: of the 94 studies covered in this review, 8 (9%) were published between 2006 and 2010, another 30 (32%) were published between 2011 and 2015, another 41 (44%) were published between 2016 and 2020, and 15 (16%) were published in 2021 and early 2022 (Figure S1 in Multimedia Appendix 5). Most interventions were delivered with human support, and most participants were female; in the studies reporting data on race, the majority of the participants were White. A total of 77 (82%) out of 94 studies used depression symptoms as assessed by clinically validated scales as the primary diagnosis, whereas only 17 (18%) specifically used MDD as an inclusion criterion. In total, the studies covered at least 68 different interventions: 47 (69%) different web-based interventions (with 10 additional web-based interventions not mentioned by name in the publications), 15 (22%) app-based interventions, 5 (7%) app-based interventions that were also accessible via the web, and 1 (1%) CD-ROM intervention. Overall, 14 (21%) out of 68 digital interventions were delivered in a language other than English: 5 (7%) in Spanish; 4 (9%) in German; and 1 (1%) each in Chinese, Swedish, Bahasa, Indonesian, Norwegian, and Portuguese.

Of the total 94 studies, 10 (11%) studies had an active control group (as defined by the use of a depression treatment other than the digital intervention under investigation, such as in-person therapy or web-based progressive muscle relaxation); 50 (53%) studies had a CAU control group (the definition of CAU varied, but CAU generally included unrestricted access to psychiatrists, medication, psychotherapy, and primary care physicians); and 43 (46%) studies had a waitlist control (27 of which reported pairing with CAU). Moreover, 14 (15%) out of 94 studies mentioned providing financial incentives to the study participants for completing various tasks, such as trial questionnaires, follow-up assessments, and the study itself. The duration for which participants were given to access the intervention ranged from 14 to 365 days, with a mean of 77 days. Appraisal of the studies indicated that of the 94 studies, 45 (48%) were of high quality, 44 (47%) were of medium quality, and 5 (5%) were of low quality. The published clinical trial protocols could not achieve the highest appraisal score because they did not have results to rate.

### Criteria Used to Assess Depression

The most common depression criterion for inclusion in these studies was having a certain minimum score on the Patient Health Questionnaire-9 (PHQ-9; 40/94, 43% studies), followed by the Beck Depression Inventory-II (10/94, 11% studies) and the Center for Epidemiologic Studies Depression Scale (CES-D; 9/94, 10% studies; Figure S2 in Multimedia Appendix 5). Studies using the same tool for assessing depression often used different depression severity cutoffs for inclusion. For example, of the 36 studies using the PHQ-9 score (38% of the 94 studies), 15 (42%) set the minimum inclusion threshold at a score of at least 5 (mild depression); 16 (44%) used a threshold score of at least 10 (moderate depression); and the rest (5/36, 14%) used scores of 4 (minimal depression), 9 (mild depression), or 15 (moderately severe depression). Of the 17 studies with inclusion criteria requiring a diagnosis of MDD, they used the *Diagnostic and Statistical Manual of Mental Disorders* (*Fourth Edition,* n=7, 41% or *Fifth Edition,* n=1, 6%) or the Mini International Neuropsychiatric Interview (version 5.0.0; n=4, 24%; Spanish version 6.0.0, n=1, 6%; Swedish version 6.0.0b, n=1, 6%; or unspecified version, n=3, 18%).

### Participant Demographics

In total, the studies reviewed in this SLR included results from 20,111 participants. The mean number of participants included in each study was 314.9 (median 150; range 8-7884). The median age of the participants was 40.0 (mean age ranged from 20.9 to 69.6) years. Of the studies reporting data on participant sex or gender, all but 1 study (80/81, 99%) had a majority female population, with a median of 72.8% (range 48.7%-88.0%) female participants. Only 28% (23/82) of the studies reporting results included data on race, and of those studies, 91% (21/23) had a majority White participant population (median 76.7%).

### Most Reported Adherence and Engagement Metrics

Two primary adherence outcomes were reported: the most common was the percentage of participants who completed the entire intervention (finished all available modules), reported by 35% (33/94) of the studies (Figure S3 in Multimedia Appendix 5). The second most common adherence metric, reported by 15% (14/94) of the studies, was the percentage of participants who completed the recommended number of modules, which, in some studies, was less than the total number of modules available.

The most common engagement outcome reported was the number of modules used or completed by participants, which was described in 43 (46%) of the 94 studies (Figure S3 in Multimedia Appendix 5). This engagement metric was also the earliest reported in the literature, along with study attrition, in 2006 (Figure S4 in Multimedia Appendix 5). Other commonly reported measures of engagement were the duration of the use of the intervention (number of hours or days used; 34/94, 36%), number of log-ins (19/94, 20%), percentage of users beginning or completing the first module (18/94, 19%), and attrition from the study (14/94, 15%); the definition of attrition varied, such as cases where participants did not complete the follow-up assessments, withdrew from the study, or did not complete any sessions of the intervention. Less frequently used measures of engagement included the percentage of participants who logged in at least once, the number of page visits, the number of activities completed, the percentage of users completing the last module of the intervention, and homework completion.

### Efficacy Metrics Most Commonly Used

Efficacy metrics were often presented as change from baseline; however, some studies reported statistics comparing the scores of the intervention group with those of the control group at a specific time point without describing the change from baseline. The PHQ-9 score was the most common metric for assessing efficacy, used in 43 (46%) out of 94 studies (Figure S5 in Multimedia Appendix 1), followed by the Beck Depression Inventory (26/94, 28%), General Anxiety Disorder-7 (16/94, 17%), CES-D (10/94, 11%), EQ-5D (9/94, 10%), Hamilton Depression Rating Scale (9/94, 10%), and remission (7/94, 7%). Other efficacy outcomes reported in multiple studies included scores on the Quick Inventory of Depressive Symptomatology, Hospital Anxiety and Depression Scale, Montgomery-Asberg Depression Rating Scale, 12-Item Short Form Survey, Automatic Thoughts Questionnaire, Kessler Psychological Distress Scale, Dysfunctional Attitude Scale, Beck Anxiety Inventory, Work and Social Adjustment Scale, and Patient Health Questionnaire-8. The most common primary efficacy outcomes were the PHQ-9 and Beck Depression Inventory, assessed in 35 (37%) and 23 (24%) out of 94 studies, respectively.

### Analysis of Adherence and Engagement Levels

**Figure 2:**
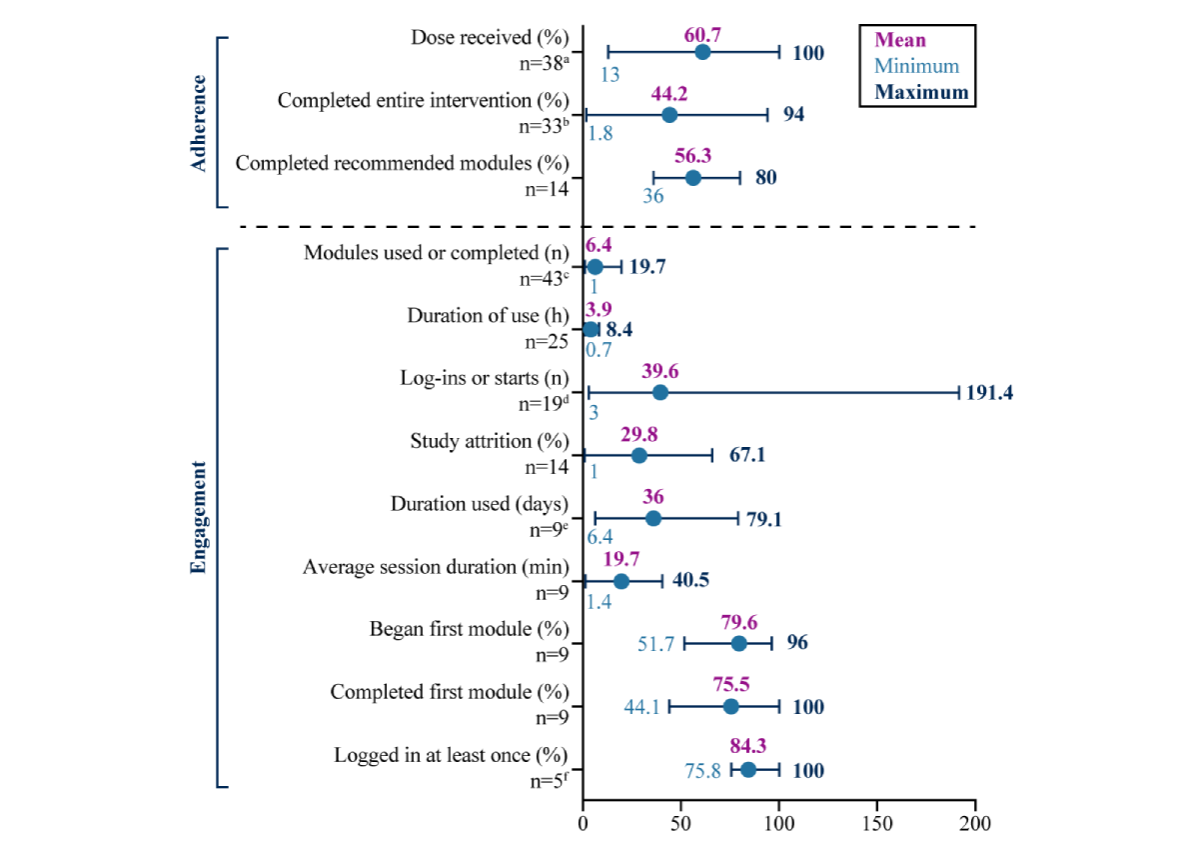
Engagement and adherence outcomes for commonly reported metrics. This graph displays the average values for the most reported engagement and adherence metrics, calculated from the means reported by the studies. The dose-received metric was calculated from the studies (38/94, 40%) that reported the number of modules used and the number of available modules (by dividing the former by the latter). For each metric, the highest and lowest means reported are also displayed, as well as the number of studies from which the metric was calculated (note: some studies reported means from multiple digital interventions, accounted for in the footnotes). ^a^42 values from 38 studies, ^b^37 values from 33 studies, ^c^51 values from 43 studies, ^d^20 values from 19 studies, ^e^12 values from 9 studies, ^f^7 values from 5 studies.

The mean percentage of participants who completed all the modules in the intervention—the most common adherence metric—was 44.2% (mean range 1.8%-94%; Figure 2). A similar adherence metric reported was the percentage of participants who completed the number of recommended modules. A mean of 56.3% of the participants completed the recommended number of modules (3-7 modules), ranging from 36% to 80% of the patients across the 14 (15%) out of 94 studies that reported this metric. We calculated the average dose received by dividing the mean number of modules used by the number of available modules. For the 38 (40%) studies reporting these 2 variables, the mean dose-received adherence metric was 60.7% (range 13%-100%). Of the 15 (16%) studies that found a statistically significant effect of the digital intervention on the primary outcome, the dose received was 66.4%.

On average, participants used or completed 6.4 modules (mean range 1.0-19.7 modules; Figure 2). In the 27% (25/94) of studies cataloging the number of hours spent engaging with the intervention, participants spent a mean of total 3.9 (mean range 0.7-8.4) hours using the digital interventions. Participants averaged 39.6 (mean range 3.0-191.4) log-ins over the course of the 20% (19/94) of studies that tracked the average number of user log-ins.

Study attrition ranged from 1% to 67.1% (mean 29.8%). The average number of days between when the participants first started using the intervention and when the participants stopped using the intervention spanned 6.4 to 79.1 (mean 36.0) days. The average percentage of participants beginning the first module ranged from 51.7% to 96%. Similarly, the average percentage of participants completing the first module ranged from 44.1% to 100%. A total of 10% (9/94) of studies reported the mean length of time participants spent on a session, which ranged from means of 1.4 to 40.5 minutes (the average was 19.7 min). A total of 5% (5/94) of studies reported the percentage of participants who logged in at least once to use the intervention, which was between 75.8% and 100% (mean 84.3%).

Analysis across the studies indicated that participants engaged with the intervention more when given a longer period to use it (ie, the number of hours participants spent engaging with digital interventions increased with the number of weeks allotted for treatment in the studies; Pearson correlation coefficient *r*=0.56; *P*=.01; n=19; Figure S6 in Multimedia Appendix 5).

### Efficacy

Overall, 21% (20/94) of studies did not use a waitlist, CAU, or active control group, including single-arm trials and studies where all groups received the digital intervention but differed from one another in some other way, such as the degree of support received or mode of intervention delivery. Of the 79% (74/94) of studies that did have a control group, 74% (55/74) reported results on the comparison of the control group with the intervention group, with 78% (43/55) finding that the digital intervention was effective for at least 1 outcome. The participants in the digital intervention group had significantly greater improvement for at least 1 outcome than the participants in the control group in 91% (30/33) of the studies with control groups that involved a waitlist versus 79% (30/38) of the studies with control groups that involved CAU. Breaking it down further by control group type, superior efficacy of the digital intervention was found in 100% (9/9) of the studies with waitlist-only groups, 90% (19/21) of the studies with groups receiving CAU while on the waitlist, 55% (6/11) of the studies with CAU-only groups, 71% (5/7) of the studies with enhanced CAU (eg, CAU plus 15-min weekly phone check-ins, a psychoeducation information session, and updated training on depression for patients’ primary care physicians), and 67% (4/6) of the studies with active control groups. Of the 55 studies that reported results on the comparison of the control group with the intervention group, 13 (24%) found no differences in outcomes between the digital intervention and control groups. Narrowing efficacy to just the primary outcome, 65% (36/55) of the studies found the digital intervention to be significantly more effective than the control. Of these studies, 61% (22/36) were appraised as high-quality studies, compared with 58% (25/43) of the studies reporting on any efficacy outcome that was significantly different in the digital intervention group compared with the control group.

### Comparison of the Use of Digital Interventions Between Studies That Allowed Psychotherapy and Those That Did Not

Only 1% (1/94) of studies listed ADT as an exclusion criterion. Some studies (33/94, 35%) allowed ADT while prohibiting other forms of treatment for depression, most commonly excluding participants from receiving psychotherapy. Most of the studies (60/94, 64%) used the digital intervention in conjunction with other forms of treatment, allowing for ADT, psychotherapy, and other forms of treatment such as inpatient care and distress call center lines. Some studies were explicit about permitting the use of other depression treatments, whereas others merely did not list the current use of psychotherapy or ADT as an exclusion criterion.

**Figure 3:**
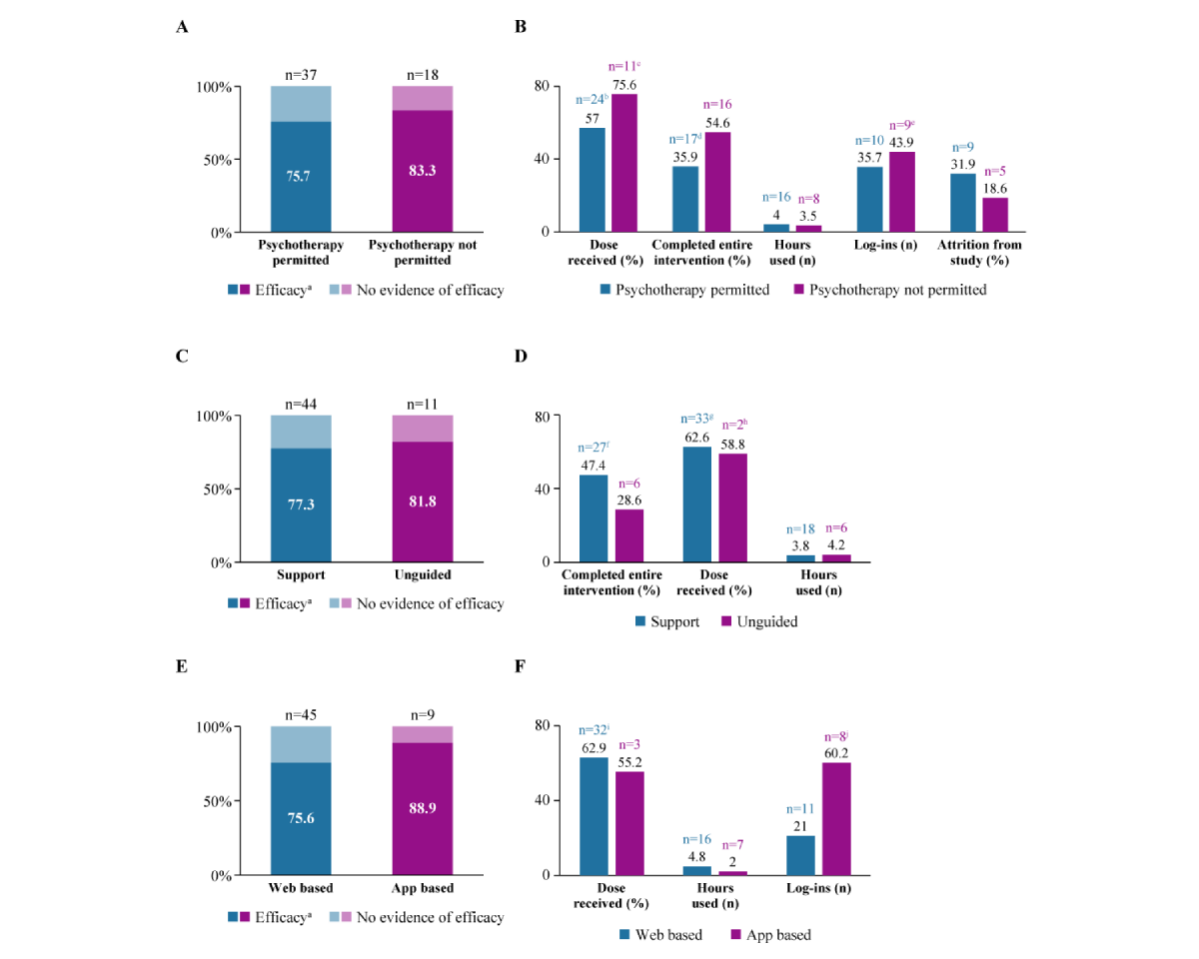
Summary of efficacy and engagement across the studies based on access to psychotherapy, intervention delivery with support, and mode of digital delivery. (A) Efficacy based on whether or not participants had access to psychotherapy. (B) Participant adherence and engagement based on whether access to psychotherapy was permitted. (C) Efficacy based on whether or not the digital interventions were delivered with support. (D) Participant adherence and engagement based on whether or not the interventions were delivered with support. (E) Efficacy based on whether the interventions were delivered via the web or via apps (the latter included interventions that were exclusively app-based and those that were app-based but could also be accessed via the web). (F) Participant adherence and engagement based on whether the interventions were delivered via the web or via apps. ^a^Efficacy refers to at least 1 outcome in which the digital intervention group experienced significantly greater improvement than the control group, ^b^30 values from 24 studies, ^c^12 values from 11 studies, ^d^21 values from 17 studies, ^e^10 values from 9 studies, ^f^31 values for 27 studies, ^g^39 values from 33 studies, ^h^3 values from 2 studies, ^i^39 values from 32 studies, ^j^9 values from 8 studies.

The percentage of studies finding efficacy for at least 1 outcome appeared to be slightly higher in the 18 studies of interventions used in a narrower context in which ADT was the only other depression treatment permitted (n=15, 83%) than in the 37 studies of interventions used in a more lenient context allowing a broader range of other treatments (n=28, 76%; Figure 3A).

In addition to moderately higher efficacy, interventions delivered in a setting with fewer permissible depression treatments had greater adherence and engagement. In studies that excluded psychotherapy compared with those that allowed a broader range of depression treatments, participants completed a higher dose of treatment (75.6% of the modules on average in 11 studies excluding psychotherapy vs 57% of the modules on average in 24 studies allowing a broader range of treatments) and were more likely to complete the entire digital intervention (54.6% vs 35.9%, n=16 and n=17 studies, respectively; Figure 3B). Furthermore, users in studies excluding psychotherapy logged in slightly more often (43.9 log-ins on average in 9 studies excluding psychotherapy vs 35.7 log-ins in 10 studies allowing a broader range of treatments) and had lower study attrition (18.6% vs 31.9%, n=5 and n=9 studies, respectively), although they spent fewer hours using the intervention (3.5 vs 4.0 hours, n=8 and n=16 studies, respectively; Figure 3B).

### Comparison of the Use of Digital Interventions Delivered With Support and Those Delivered Without Support

In most of the studies (77/94, 82%), the digital intervention was delivered with support (eg, an onboarding call, weekly check-in calls from coaches, written feedback after completing each lesson, or adherence reminder calls), whereas in 18% (17/94) of studies, delivery was unguided. The percentage of studies demonstrating that the intervention had efficacy for at least 1 participant outcome (such as depression severity or quality of life) appeared to be similar whether the digital intervention was delivered with human support (34/44, 77%) or without support (9/11, 82%; Figure 3C). More participants completed all the modules when given support (27/57, 47% of participants, n=27 studies) than when unguided (29% of participants, n=6 studies; Figure 3D); however, they spent slightly less time using the intervention on average (3.8 vs 4.2 hours, n=18 studies and n=6 studies, respectively; Figure 3D). Because only 2 studies of unguided interventions reported enough information to calculate the dose-received values, we refrained from interpreting that comparison.

### Comparison of the Use of Web-Based Versus App-Based Interventions

We investigated the difference between interventions that were web based and those that were app based (which included interventions that were exclusively app based and those that were app based but could also be accessed via the web). Studies of web-based interventions were published, on average, 3 years earlier than studies of app-based interventions (2015 vs 2018; Figure S1 in Multimedia Appendix 5). When assessed by delivery type, the percentage of studies finding efficacy for at least 1 outcome was higher for app-based interventions, with 76% (34/45) of the studies using web-based interventions and 89% (8/9) of the studies using app-based interventions finding the digital intervention to be significantly more effective than the control group (Figure 3E). However, this finding should be interpreted with caution because the sample was heterogeneous, and the number of app-based interventions was small (n=9). The mean number of hours of use of the intervention was higher in the web-based studies than in the app-based studies (4.8 hours in 16 studies of web-based interventions vs 2.0 hours in 7 studies of app-based interventions), as was the dose received (62.9% vs 55.2%; Figure 3F). The web-based studies had a lower mean number of log-ins (21.0 log-ins, n=11 studies) than the app-based studies (60.2 log-ins, n=8 studies; Figure 3F), perhaps owing to differences in intervention design.

### Relationship of Efficacy With Adherence and Engagement

Of the 94 studies, only 14 (15%) reported on a dose-response relationship, investigating an association between the level of adherence to or engagement with the digital intervention and efficacy outcomes (change in depression, anxiety, or stress scores; Table 2). Of these studies, 9 (64%) detected a significant relationship between adherence or engagement and at least 1 efficacy outcome.

Donkin et al [61] found that participants who improved by ≥5 points on the PHQ-9 had completed slightly more activities and logged in for slightly more time than those who did not. Similarly, MacLean et al [86] and Wright et al [73] reported a correlation between the PHQ-9 score and the number of modules completed [73,86]. Bur et al [71] noted a positive correlation between a composite adherence score (averaged from the time spent on the program and the number of clicks done, topics completed, and exercises completed) and improvement in the PHQ-9 score.

Moreover, Bolier et al [113] indicated that participants who completed >1 lesson had significantly greater improvement in their Hospital Anxiety and Depression Scale anxiety subscale scores at 2-month follow-up than those who completed ≤1 lesson. Mohr et al [62] reported that several engagement metrics were significantly associated with improvement in the PHQ-9 score: the number of days on which participants logged in, the number of lessons they viewed, the total number of tools they used, and the variety of tools they used. A subsequent study by Kelders et al [105] discovered that the completion of all modules and lesson reached were associated with improvement in CES-D and Hospital Anxiety and Depression Scale anxiety subscale scores after intervention and at follow-up. Krämer and Köhler [132] did not directly investigate the relationship between engagement and efficacy outcomes; however, the effect size of treatment on CES-D score improvement was greater in 52% of the participants who completed at least 5 modules than in all participants.

Moberg et al [88] found that the participants who used the digital intervention to record more of their thoughts in the app had greater reductions in anxiety and the Depression Anxiety and Stress Scales-21 stress subscale score from the posttreatment time point to follow-up; however, an initial increase in stress from baseline to the posttreatment time point was revealed in the same participants.

Of the 14 studies, 5 (36%) did not find a dose-response relationship. Of these 5 studies, 4 (80%) found the digital intervention to be effective for at least 1 outcome compared with the control, without finding efficacy to be correlated with engagement or adherence [63,64,87,102]. Batterham et al [78] reported that there was no relationship between module completion (none vs some) and either the PHQ-9 or General Anxiety Disorder-7 score; however, they also found no effect of the intervention on depression or anxiety overall.

**Table 2:** Summary of the studies that investigated a relationship between engagement and participant outcomes.

Intervention type	Study	Digital intervention	Treatment duration	Engagement outcome	Efficacy outcome	Is there a relationship between engagement and efficacy?
Web based	Donkin et al [61], 2013	E-couch	12 weeks	Number of activities completed per log-in	Participants whose PHQ-9^a^ score improved by ≥5 points compared with those whose score did not	Yes (mean difference 0.20 activities, *P=*.01)
Web based	Donkin et al [61], 2013	E-couch	12 weeks	Time spent per log-in	Participants whose PHQ-9 score improved by ≥5 points compared with those whose score did not	Yes (mean difference 3.26 min, *P=*.01)
Web based	Bolier et al [113], 2013	Psyfit	2 months	Participants completing >1 lesson compared with those completing ≤1 lesson	HADS-A^b^ at 2-month follow-up	Yes (Cohen *d*=0.16 vs Cohen *d*=0.43, *P=*.03)
Web based	Bolier et al [113], 2013	Psyfit	2 months	Participants completing >1 lesson compared with those completing ≤1 lesson	CES-D^c^ at 6-month follow-up	No (but there was a trend; Cohen *d*=0.47 vs Cohen *d*=0.87, *P=*.07)
Web based	Bolier et al [113], 2013	Psyfit	2 months	Number of lessons completed	Change in score on MHC-SF^d^, WHO-5^e^, CES-D, HADS-A, and MOS SF^f^	No (no clear relationship between adherence and effect size for any of the scales)
Web based	MacLean et al [86], 2020	The Journal	12 weeks	Number of lessons completed	PHQ-9 score at week 12	Yes (*r*=−0.436, *P=*.002)
Web based	Mohr et al [62], 2013	moodManager	12 weeks	Number of log-in days	Improvement in PHQ-9 score	Yes (β=.14; *P=*.02)
Web based	Mohr et al [62], 2013	moodManager	12 weeks	Number of lessons viewed	Improvement in PHQ-9 score	Yes (β=.40, *P=*.01)
Web based	Mohr et al [62], 2013	moodManager	12 weeks	Total tool use	Improvement in PHQ-9 score	Yes (β=.01, *P=*.01)
Web based	Mohr et al [62], 2013	moodManager	12 weeks	Variety of tools used	Improvement in PHQ-9 score	Yes (β=1.21, *P=*.03)
Web based	Kelders et al [105], 2015	Living to the full	12 weeks	Participants completing 0 to 5 lessons compared with those completing all 9 lessons	Change in CES-D score from baseline to follow-up	Yes (the effect size for participants who completed 0 to 5 lessons was Cohen *d*=0.64, compared with Cohen *d*=1.20 for participants who completed all 9 lessons, *P*<.001)
Web based	Kelders et al [105], 2015	Living to the full	12 weeks	Participants completing 0 to 5 lessons compared with those completing all 9 lessons	Change in HADS-A score from baseline to follow-up	Yes (the effect size for participants who completed 0 to 5 lessons was Cohen *d*=0.33, compared with Cohen *d*=1.12 for participants who completed all 9 lessons, *P*<.001)
Web based	Kelders et al [105], 2015	Living to the full	12 weeks	Adherence and lesson reached	CES-D and HADS-A at the postintervention time point and follow-up	Yes (all regression analyses were significant, with *P*<.001 and β=.242 to.422, supporting a dose-response relationship; specific values not reported)
App based	Moberg et al [88], 2019	Pacifica	30 days	Number of times a participant made a thought record	Anxiety reduction from the posttreatment time point to follow-up	Yes (the more the number of thought records made by a participant, the more their anxiety score reduced; β=−.10; *P=*.02)
App based	Moberg et al [88], 2019	Pacifica	30 days	Number of times a participant made a thought record	Stress reduction from baseline to the posttreatment time point and from the posttreatment time point to follow-up	Yes (the more the number of thought records made by a participant, the lesser the reduction in their stress from the pretreatment to posttreatment time points [β=−.30; *P*<.01] but the greater the stress reduction from the posttreatment time point to follow-up [β=−.47; *P*<.05])
App based	Moberg et al [88], 2019	Pacifica	30 days	Number of times a participant made a thought record	Change in depression composite score, based on a composite of scores on the PHQ-8^g^ and DASS-21^h^ depression subscale	No (*P*>.13)
Web based	Bur et al [71], 2022	HERMES	8 weeks	Composite adherence score (averaged *z* scores of the number of clicks, number of topics worked on, number of completed exercises, and time spent on the program)	Change in PHQ-9 score	Yes (Kendall τ=0.11, *P=*.03)
Web based	Krämer and Köhler [132], 2021	GET.ON Mood Enhancer	7 weeks	CES-D	Change in CES-D score	Yes (authors did not directly investigate; however, the effect size of the intervention on CES-D scores for all participants was Cohen *d*=0.55, whereas effect size for the participants who completed ≥5 modules was larger, Cohen *d*=0.75.)
Web based	Wright et al [73], 2022	Good Days Ahead	12 weeks	Number of modules completed	Change in PHQ-9 score	Yes (estimate, −0.85, *P=*.009)
Web based	Meyer et al [102], 2015	Deprexis	3 months	Mean minutes users engaged with the program (use time)	Change in PHQ-9 score	No (*P*>.20)
Web based	Moritz et al [63], 2012	Deprexis	8 weeks	Number of sessions completed	Change in BDI^i^ score	No (*r*<0.11, *P*>.30)
App based	Graham et al [87], 2020	IntelliCare suite	8 weeks	Number of app sessions	Change in PHQ-9 score	No (*r*=−0.03; 95% CI −0.22 to 0.15)
App based	Graham et al [87], 2020	IntelliCare suite	8 weeks	Time until last use	Change in PHQ-9 score	No (*r*=−0.14; 95% CI −0.31 to 0.05)
App based	Graham et al [87], 2020	IntelliCare suite	8 weeks	Number of days used	Change in PHQ-9 score	No (*r*=−0.05; 95% CI −0.23 to 0.14)
App based	Graham et al [87], 2020	IntelliCare suite	8 weeks	Number of app sessions	Change in GAD-7^j^ score	No (*r*=0.01; 95% CI −0.17 to 0.19)
App based	Graham et al [87], 2020	IntelliCare suite	8 weeks	Time until last use	Change in GAD-7 score	No (*r*=−0.04; 95% CI −0.22 to 0.13)
App based	Graham et al [87], 2020	IntelliCare suite	8 weeks	Number of days used	Change in GAD-7 score	No (*r*=0.02; 95%CI −0.15 to 0.20)
App based	Stiles-Shields et al [64], 2019	Boost Me and Thought Challenger	6 weeks	App use	Change in PHQ-9 score	No (*P*>.05)
Web based	Batterham et al [78], 2021	myCompass 2	7 weeks	Module completion (0 vs 1-14 modules)	PHQ-9 score	No (*P=*.74)
Web based	Batterham et al [78], 2021	myCompass 2	7 weeks	Module completion (0 vs 1-14 modules)	GAD-7 score	No (*P=*.87)

^a^PHQ-9: Patient Health Questionnaire-9.

^b^HADS-A: Hospital Anxiety and Depression Scale anxiety subscale.

^c^CES-D: Center for Epidemiological Studies Depression Scale.

^d^MHC-SF: Mental Health Continuum-Short Form.

^e^WHO-5: 5-item World Health Organization Well-being Index.

^f^MOS SF: Medical Outcomes Study-Short Form.

^g^PHQ-8: Patient Health Questionnaire-8.

^h^DASS-21: Depression Anxiety and Stress Scales-21.

^i^BDI: Beck Depression Inventory.

^j^GAD-7: General Anxiety Disorder-7.

## Discussion

### Principal Findings

#### Overview

This SLR of participant adherence to and engagement with digital interventions for depression was comprehensive, including 94 publications and 20,111 participants. Of the 55 publications that reported results on the comparison of a digital intervention group with a control group, 78% (n=43) found that the digital intervention group had greater improvement in at least 1 efficacy outcome. Participant adherence and engagement varied widely in terms of how they were defined and measured and their levels. Although acknowledging that the field lacks universal definitions of adherence to and engagement with digital interventions [46,52-55], we categorized adherence metrics as those that involved a comparison of intended use with actual use of the intervention [55] and engagement metrics as those that involved an assessment of the extent to which participants interacted with an intervention (eg, number of hours used, modules used, log-ins, days used). Only 6% (6/94) of studies of engagement with digital interventions for depression were published by 2010, with the majority (56/94, 60%) published in 2016 or after. As the number of such studies continues to increase, it is important for clinical trials of digital interventions to align with a common set of core adherence and engagement metrics. This alignment will encourage the consistent reporting of user engagement to make comparisons of digital interventions across studies more meaningful.

#### Engagement With Digital Interventions for Depression

Measuring DTx engagement is complicated. Studies of digital interventions often use metrics of engagement that do not describe the quality of the interaction with the intervention material [58]. In the studies reviewed in this SLR, the most common engagement metric was the number of modules used. However, this result does not account for the varying lengths and qualities of the modules across digital interventions, the degree of attention the users paid to the modules as they proceeded, or their level of retention of module content. It also does not distinguish between users who were repeating modules and those who moved sequentially through the intervention, nor does it reveal task time analytics. Moreover, for this SLR, the number of modules completed was not an ideal metric for comparing different types of digital interventions because the number of available modules varied considerably across interventions (from 4 to 20).

To address this issue, we calculated a dose-received metric of adherence from the 40% (38/94) of studies that made available both the mean number of modules used and the total number of available modules. This is not a perfect comparative metric because it does not factor in a module’s quality, length, or difficulty or the effort required to complete the module, which would require researchers to track and report more variables than have been published to date. Furthermore, not all digital interventions can be measured by this dose-received metric, such as chatbots and mood trackers. Thus, dose received is a rough but practical comparison tool. The dose-received metric calculated in this SLR revealed that the participants received an average dose of 60.7% of the modules available in the digital interventions.

Related adherence metrics were the percentage of participants who completed the recommended number of modules (56.3%, reported by 14/94, 15% of studies) and the percentage of participants who completed all available modules (44.2%, reported by 33/94, 35% of studies). These values were within the range of the 43% to 99% completion rates reported by other clinical studies of digital interventions [46] and higher than the 0.5% to 28.6% completion rates reported in the real-world use of depression and anxiety apps [46]. As a rough comparison to the use of other depression treatments in the real world, 35% of patients were classified as adherent to antidepressants by having at least 80% proportion of days covered [162], and although 12 to 20 sessions of cognitive behavioral therapy are recommended for depression [163], the median number of psychotherapy sessions attended was reported as 5 in 1 (1%) out of 94 studies [164] and another (n=1, 1%) found that most patients attended only 1 session [165].

It is possible that the quality of the digital interventions studied in clinical trials is higher than the quality of the apps that have not been tested for efficacy before being released to the public. Although 78% (74/94) of the studies with a control group reviewed here found the digital intervention to be effective for at least 1 participant outcome, most depression apps on the market do not have any efficacy data [32].

#### Adherence, Engagement, and Efficacy Based on Whether the Studies Allowed Participants to Access Psychotherapy

The effect size of a digital intervention can be impacted by the type of control group used for comparison [166,167]. A meta-analysis of internet-delivered cognitive behavioral therapy studies revealed that interventions had higher effect sizes when compared with a waitlist control versus a CAU group [166]. Similarly, in a meta-review of meta-analyses examining the outcomes of RCTs of app-based interventions, Goldberg et al [167] reported that digital interventions had a small but significant effect on improving depression symptoms compared with inactive controls but not compared with active controls. In line with these conclusions, in this SLR, the percentage of studies that found the digital intervention group to have a significantly greater improvement in at least 1 outcome depended on the type of control group used as a comparator, with a greater percentage of waitlist control studies finding efficacy than active control studies. This indicates that app-based interventions for depression may have the highest efficacy when used before initiating treatment, such as for patients on waitlists or those without feasible access to mental health care. Furthermore, it underscores the importance of noting whether the digital intervention was used as a monotherapy or an adjunctive treatment and of carefully considering what type of control group is used when analyzing studies of digital interventions.

All 94 studies reviewed here except 1 (1%) allowed antidepressant use along with the digital intervention. Just over one-third (33/94, 35%) of the studies excluded psychotherapy. The remaining studies allowed for the digital intervention to be used as an adjunct to medication, psychotherapy, and other forms of treatment. Compared with participants in studies allowing broad access to treatment, participants in studies where psychotherapy was not allowed had higher adherence and engagement, receiving a higher dose, completing the intervention at higher rates, and logging in more often (although spending less overall time using the intervention). There may have been more of an incentive to engage with the intervention when psychotherapy was not permitted as a treatment. Thus, digital interventions may be more valuable when psychotherapy is inaccessible or not the patient’s preference. However, there is no consensus in the field yet on whether digital interventions are most useful before beginning psychotherapy or best when used as stand-alone treatments [54,68,86,168,169]. Although some studies have indicated that digital interventions can decrease symptoms and benefit waitlisted patients before starting therapy [168,170], others have found no benefit [86,171].

A missing piece is the understanding of which patients will benefit most from digital interventions. Levin et al [172] found high satisfaction ratings from students using an app while on a waitlist for a college counseling center; however, recruitment was slow, and researchers concluded that the app may only interest a select subsample of the population. Prior research has indicated that adherence to digital interventions is affected by age, symptom severity, and gender, but the direction of the effects has differed from study to study [54]. Karyotaki et al [168] have called for future studies to include more participants from disadvantaged backgrounds (who may encounter issues using digital interventions owing to poverty or education level) and to methodically investigate factors that could impact the effectiveness of a digital treatment, including the duration of depression symptoms, comorbidities, the number of prior depressive episodes, and demographics. Such research is crucial for designing digital interventions for patients who may benefit the most from them and ensuring access for these populations.

#### The Diverse Support Offered With Digital Interventions

Previous research has indicated that digital interventions are more successful when support is provided [168]; however, the appropriate amount of guidance to offer has yet to be established [54]. The majority of the studies reviewed in this SLR offered some form of support in various ways. Support was sometimes marginal, such as a 10-minute phone call in the second week of the intervention [90], minimal email contact with psychologists [133], or an optional onboarding phone call [131]. Support could also be intensive, such as a psychiatrist appointment, 12 weekly 30- to 60-minute phone calls with a coach, and written contact between appointments [161]. One study used intense monitoring, facilitated early intervention, and assisted with personal crisis management [100].

Common support measures offered were weekly phone calls with a coach [62,64,73,74,80,81,84,86,94,103,109,116,119,132, 134,139,148,159,161] and adherence reminders [61,63,95,107,112,122,129,144,153,158]. Many studies provided coach feedback to participants after each lesson [72,75,77,99,106,108,111,122,124,130,135,141,144,145,154,155,157,158]. A few studies integrated the digital intervention with face-to-face meetings, either with a therapist [104,112] or in a group counseling session [85,125]. Moreover, 1 (1%) study ensured equal access by giving participants phones with phone plans for the duration of the study [116].

Interestingly, efficacy did not appear to be considerably impacted by whether support was provided. The interventions delivered with support were as likely to have efficacy for at least 1 participant outcome as those delivered without support. The literature on this topic may be less conclusive than previously thought. Although multiple studies have shown that interventions delivered with support have larger effect sizes than unguided interventions, it has been suggested that newer unguided digital interventions with features such as engagement reminders may have similar efficacy to interventions delivered with clinician support [173]. The patient population may also modulate the effect of guidance: Karyotaki et al [168] found that guidance did not impact the efficacy of a digital intervention for patients with a PHQ-9 score of 5 to 9; however, patients with higher PHQ-9 scores had better outcomes when the intervention was delivered with support.

Although support did not appear to impact efficacy in the reviewed studies, it did play a role in adherence. Studies that provided support had greater adherence than those that did not, with more participants completing all the modules in the former. It is not clear whether support helps improve adherence directly or by indirect mechanisms such as hope induction [54], nor what degree or form of support is best or if it differs among patients. Only 1 (1%) study differentiated support depending on baseline participant characteristics by offering email support from a therapist to participants with a PHQ-9 score >10 [143]. It is important to determine which patients will most benefit from support and to provide future resources to these patients for optimized personalized treatments.

#### Web-Based Versus App-Based Interventions

Engagement with web-based interventions for depression has been studied for longer than engagement with app-based interventions; however, the number of publications on the latter has been steadily increasing since 2016.

Despite there being a lower proportion of studies of web-based interventions reporting efficacy than studies of app-based interventions reporting efficacy, a higher degree of participant adherence and engagement was found for web-based interventions: participants spent a greater number of hours, on average, using the web-based intervention and averaged a higher dose. However, the number of studies with engagement data from apps was relatively low (n=9); thus, it is unknown whether such a pattern would hold for a larger sample size. It is also possible that web-based interventions may have been designed to take more time, on average, than mobile apps. One small study investigated the same intervention in both web-based and app-based forms and did not find a statistically significant difference in the number of participants completing all lessons (14/20, 70%, vs 10/15, 67%) or a difference in efficacy in terms of preintervention to postintervention scores on the PHQ-9, Beck Depression Inventory-II, or Kessler Psychological Distress Scale [156]. Future research should consider whether delivering a digital intervention via the web or an app is better for patient engagement and efficacy.

#### The Relationship of Adherence and Engagement With Efficacy

Many studies of digital interventions assume that there is a linear dose-response relationship, in which greater engagement with the intervention comes with greater efficacy, although such a relationship does not always exist or may not be linear [55,58]. As a result, the level of engagement recommended to achieve efficacy from digital interventions is often not justified with data [58]. Some patients may stop engaging with a digital intervention early because they are feeling better [174]. Further, an effective use pattern may differ from user to user [55]. Using machine learning and a data set of >54,000 adults using an internet-based cognitive behavioral therapy platform for depression and anxiety, Chien et al [175] identified 5 discrete subtypes of users based on engagement and concluded that the level of engagement was not always proportional to clinical improvements. They found that 1 subtype of users engaged more with core modules and mood tracking, whereas another engaged with relaxation and mindfulness tools; different forms of engagement still produced results, with each of the 5 subtypes experiencing an average reduction in the PHQ-9 score of at least 4.4 after 14 weeks [175]. Therefore, it may be useful to investigate >1 metric of engagement when measuring a dose-response relationship because the metrics alone may impact the ability to detect such a relationship [55].

In this SLR, the PHQ-9 was the most frequently used tool for both determining participant eligibility for the trial and reporting the efficacy of the intervention. Most of the studies with a control group found the digital intervention to be effective for at least 1 outcome compared with the control. However, only 14 (15%) of the 94 studies reported having analyzed the relationship between participant adherence or engagement and efficacy; approximately two-thirds (9/14, 64%) indicated such a relationship, with each finding increased efficacy with increased adherence or engagement.

The relationship between engagement and efficacy is complex. As reviewed here, it is not a unanimous finding in the existing literature that the more patients engage with the intervention, the better their outcomes. Therefore, it is difficult to determine the extent of engagement necessary to improve depressive symptoms.

#### Underreporting of Participant Race and Ethnicity Data

Sociocultural factors influence the acceptability and efficacy of digital interventions [176,177], yet only a few studies (23/82, 28%) in this SLR reported on the race or ethnicity of the participants. Despite the importance of diversity, many publications from clinical trials do not report race and ethnicity data [178,179]. Even after the requirement in 2017 to submit race and ethnicity data with trial results to the clinical trial database registry, only 62.4% of the studies have included these data in their publications [178]. In a review of 342 RCTs on interventions for depression from 1981 to 2016, Polo et al [179] found that only 43.3% (n=148) of the studies reported on the participants’ race or ethnicity. The studies of digital interventions for depression reviewed here performed far below this average, with only 29% (24/82) including race and ethnicity data. Among those that did report such data, the lack of diversity was striking. Polo et al [179] reported that a mere 16.7% (n=57) of the studies included at least 50% ethnic populations; in this review, it was only 8.7% (n=8). Presumably, a few more studies we reviewed did include at least 50% ethnic populations, such as those conducted in Latin America; however, these studies did not report race or ethnicity data; thus, they cannot be quantified. It is crucial that studies of digital interventions report race and ethnicity data and that they include unrepresented populations.

#### Considerations for Clinicians and Patients

There are several factors for patients and clinicians to consider when deciding whether to use a digital intervention for depression. First, apps should be evaluated for their efficacy and quality [163]. There are several organizations and tools to help evaluate digital interventions, including the American Psychiatric Association’s App Advisor [180], One Mind’s PsyberGuide [181], Organisation for the Review of Care and Health Apps’s App Library [182], Mobile App Rating Scale [183], mobile health app trustworthiness checklist [184], Framework to Assist Stakeholders in Technology Evaluation for Recovery [185], and Mhealth Index and Navigation Database [186]. A second factor to consider is patient traits. An SLR of 208 articles on digital mental health interventions concluded that the patients who were most likely to engage with digital interventions were women; had high digital health literacy; and had friends, family, and health care providers who supported their use of digital interventions [187]. Extroversion, fatigue, and more severe depression symptoms were barriers to engagement [187]. The same review pointed to program design as another important factor. Patients were more engaged with interventions that enabled them to connect with other users; had content that was credible, customizable, and relevant; and instilled a sense of privacy [187]. Additional considerations include patients’ commitment to engaging with a digital intervention for enough time to gain benefits, whether they have a preference for using an intervention that is guided or unguided, and whether they prefer to use the intervention as an adjunct to other depression treatments or as a stand-alone treatment.

### Limitations and Strengths

One of the limitations of this SLR is publication bias; on average, studies with statistically significant results and larger effect sizes are more likely to be published than those with negative results or small effect sizes [188]. In addition, there is a potential for bias toward studies with higher adherence and engagement. Furthermore, studies could have reported only some exploratory results, but not others, and some reported on only participants who completed the intervention. Considering all these factors, this review may overestimate the effects of and engagement with digital interventions. Additional limitations include that each article was screened by 1 reviewer, and that because this is an SLR rather than a meta-analysis, our results are descriptive rather than statistical. Another limitation is that fewer than half of the studies reported both the total number and average number of modules used; therefore, the dose-received metric we calculated was drawn from a limited number of studies (38/94, 40%), making it less generalizable. Furthermore, it is possible that the relationship between engagement and efficacy was examined in an exploratory or a post hoc analysis in some studies that did not report a negative or nonsignificant finding, which would overinflate the positive findings in the literature. Although this SLR includes data from >20,000 participants, it is possible that some of these individuals were not unique if they participated in >1 of the studies. Yet another limitation is that these studies were biased toward a White participant population, which restricts the generalizability of the findings. Other important demographic information such as socioeconomic status, level of education, health care coverage and accessibility, and geographic location was rarely, if ever, reported. This lack of reported demographic data can and must be addressed in future studies if we are to move toward equitable access and personalized medicine. In addition, studies using only the PHQ-9 as a screening tool may have unintentionally included some individuals experiencing a bipolar depressive episode. Other covariates that were not accounted for included concurrent medication, other health comorbidities, and the severity of depression. Further, these findings may not reflect more recent modes of treatment that were outside the scope of this review, such as chatbots.

Despite these limitations, this SLR highlights many current problems that, if addressed, will strengthen the field. The strengths of this review include the relatively large number of articles analyzed (n=94) and the cumulative number of participants included in the studies (n=20,111).

### Conclusions

These findings have different implications for different stakeholders. For digital intervention developers, a key takeaway is that even in the controlled environments of research trials, participants with depression used the interventions, on average, for only 3.9 hours in total (and app-based interventions, on average, for only 2.0 hours in total). Thus, it would be prudent for developers to front-load the most important content in the beginning modules. For mental health care providers, it may be helpful to conceive of digital interventions as short-term rather than long-term treatments, particularly for patients on waitlists. For patients, considerations include whether they are willing and able to make the time commitment involved in using a digital intervention long enough to receive the recommended dose, whether they would like to use a guided intervention, and whether they prefer to use the intervention as an adjunct to their standard of care treatment. For the research field, improvements could be made by using consistent metrics to report adherence (eg, dose received) and engagement (eg, hours spent using the intervention), through regular inclusion of control groups and patients of diverse backgrounds in studies, by always reporting race and ethnicity data in publications, by investigating the interplay of socioeconomic factors and the efficacy of digital interventions, and by measuring the dose-response relationship to make data-informed decisions about dose recommendations.

## Appendix

Multimedia Appendix 1 PRISMA (Preferred Reporting Items for Systematic Reviews and Meta-Analyses) 2020 item checklist.

Multimedia Appendix 2 Outcomes for each reference excluded during abstract and full-text screening.

Multimedia Appendix 3 Sources and study types of each reference included in the systematic literature review.

Multimedia Appendix 4 Study characteristics of references included in this systematic literature review.

Multimedia Appendix 5 Visualizations of adherence and engagement metrics.

## Abbreviations

ADT: antidepressant treatment
CAU: care as usual
CES-D: Center for Epidemiologic Studies Depression
DTx: digital therapeutics
MDD: major depressive disorder
PHQ-9: Patient Health Questionnaire-9
PRISMA: Preferred Reporting Items for Systematic Reviews and Meta-Analyses
RCT: randomized controlled trial
SLR: systematic literature review

## Data Availability

The data sets generated during this study are available from the corresponding author upon reasonable request.
