# Supplementary material for: Assessing Patient Adherence to and Engagement With Digital Interventions for Depression in Clinical Trials: Systematic Literature Review
Source: J Med Internet Res. 2023 Aug 11;25:e43727. doi: 10.2196/43727 (PMC10457707; doi:10.2196/43727)
Supplement: Multimedia Appendix 1 [file jmir_v25i1e43727_app1.docx]

**Forbes et al. 2023 Multimedia Appendix 1: Table S1**

Table S1. PRISMA 2020 item checklist.

| **Section and Topic** | **Item #** | **Checklist Item** | **Location Where Item  Is Reported** |
| --- | --- | --- | --- |
| **Title** | | | |
| Title | 1 | Identify the report as a systematic review | Title |
| **Abstract** | | | |
| Abstract title | 2a | Identify the report as a systematic review | Title |
| Objectives | 2b | Provide an explicit statement of the main objective(s) or question(s) the review addresses | Abstract, Objective section |
| Eligibility criteria | 2c | Specify the inclusion and exclusion criteria for  the review | Abstract, Methods section |
| Information sources | 2d | Specify the information sources (eg, databases, registers) used to identify studies and the date when each was last searched | Abstract, Methods section |
| Risk of bias | 2e | Specify the methods used to assess risk of bias in the included studies | Abstract, Results section |
| Synthesis of results | 2f | Specify the methods used to present and synthesize results | Abstract, Methods section |
| Included studies | 2g | Give the total number of included studies and participants and summarize relevant characteristics of studies | Abstract, Methods and  Results sections |
| Synthesis of results | 2h | Present results for main outcomes, preferably indicating the number of included studies and participants for each. If meta-analysis was done, report the summary estimate and confidence/credible interval. If comparing groups, indicate the direction of the effect (ie, which  group is favored). | Abstract, Results section |
| Limitations of evidence | 2i | Provide a brief summary of the limitations of the evidence included in the review (eg, study risk of bias, inconsistency and imprecision) | Abstract, Results section |
| Interpretation | 2j | Provide a general interpretation of the results and important implications | Abstract, Conclusion section |
| Funding | 2k | Specify the primary source of funding for  the review | Abstract, Methods section |
| Registration | 2l | Provide the register name and registration number | N/A |
| **Introduction** | | | |
| Rationale | 3 | Describe the rationale for the review in the context of existing knowledge | Introduction |
| Objectives | 4 | Provide an explicit statement of the objective(s) or question(s) the review addresses | Introduction |
| **Methods** | | | |
| Eligibility criteria | 5 | Specify the inclusion and exclusion criteria for the review and how studies were grouped for  the syntheses | Methods, “Study Selection Criteria” section; Table 2 |
| Information sources | 6 | Specify all databases, registers, websites, organizations, reference lists and other sources searched or consulted to identify studies. Specify the date when each source was last searched  or consulted | Methods, “Searches” section |
| Search strategy | 7 | Present the full search strategies for all databases, registers, and websites, including any filters and limits used | Textbox |
| Selection process | 8 | Specify the methods used to decide whether a study met the inclusion criteria of the review, including how many reviewers screened each record and each report retrieved, whether they worked independently, and if applicable, details of automation tools used in the process | Methods, “Searches” section |
| Data collection  process | 9 | Specify the methods used to collect data from reports, including how many reviewers collected data from each report, whether they worked independently, any processes for obtaining or confirming data from study investigators, and if applicable, details of automation tools used in  the process | Methods, “Searches” section |
| Data items | 10a | List and define all outcomes for which data were sought. Specify whether all results that were compatible with each outcome domain in each study were sought (eg, for all measures, time points, analyses), and if not, the methods used to decide which results to collect | Methods, “Extraction” section |
|  | 10b | List and define all other variables for which data were sought (eg, participant and intervention characteristics, funding sources). Describe any assumptions made about any missing or  unclear information. | Methods, “Extraction” section |
| Study risk of bias  assessment | 11 | Specify the methods used to assess risk of bias in the included studies, including details of the tool(s) used, how many reviewers assessed each study and whether they worked independently, and if applicable, details of automation tools used in the process | Methods, “Extraction” section |
| Effect measures | 12 | Specify for each outcome the effect measure(s) (eg, risk ratio, mean difference) used in the synthesis or presentation of results | Methods, “Analysis” section |
| Synthesis methods | 13a | Describe the processes used to decide which studies were eligible for each synthesis (eg, tabulating the study intervention characteristics and comparing against the planned groups for each synthesis [item #5]) | Methods, “Analysis” section |
|  | 13b | Describe any methods required to prepare the data for presentation or synthesis, such as handling of missing summary statistics, or data conversions | Methods, “Analysis” section |
|  | 13c | Describe any methods used to tabulate or visually display results of individual studies and syntheses | Methods, “Analysis” section |
|  | 13d | Describe any methods used to synthesize results and provide a rationale for the choice(s). If meta-analysis was performed, describe the model(s), method(s) to identify the presence and extent of statistical heterogeneity, and software  package(s) used. | N/A |
|  | 13e | Describe any methods used to explore possible causes of heterogeneity among study results (eg, subgroup analysis, metaregression) | Methods, “Analysis” section |
|  | 13f | Describe any sensitivity analyses conducted to assess robustness of the synthesized results | N/A |
| Reporting bias  assessment | 14 | Describe any methods used to assess risk of bias due to missing results in a synthesis (arising from reporting biases) | N/A |
| Certainty assessment | 15 | Describe any methods used to assess certainty (or confidence) in the body of evidence for  an outcome | N/A |
| **Results** | | | |
| Study selection | 16a | Describe the results of the search and selection process, from the number of records identified in the search to the number of studies included in the review, ideally using a flow diagram | Results, “Studies Selected” section; Figure 2 |
|  | 16b | Cite studies that might appear to meet the inclusion criteria, but which were excluded, and explain why they were excluded | N/A |
| Study characteristics | 17 | Cite each included study and present  its characteristics | Results, “Studies Selected,” “Study Characteristics and Types of Digital Interventions” sections; Table S2; Table S4 |
| Risk of bias  in studies | 18 | Present assessments of risk of bias for each included study | N/A |
| Results of  individual studies | 19 | For all outcomes, present, for each study: (a) summary statistics for each group (where appropriate); and (b) an effect estimate and its precision (eg, confidence/credible interval), ideally using structured tables or plots | N/A |
| Results of syntheses | 20a | For each synthesis, briefly summarize the characteristics and risk of bias among  contributing studies | Results, “Study Characteristics and Types of Digital Interventions,” “Criteria Used to Assess Depression,” “Participant Demographics,” “Most Reported Adherence and Engagement Metrics,” “Efficacy Metrics Most Commonly Used,” “Analysis of Adherence and Engagement Levels,” “Efficacy,” “Comparison of Use of Digital Interventions in Studies That Allowed Psychotherapy Versus Those That Did Not,” “Comparison of Use of Digital Interventions Delivered With and Without Support,” “Comparison of Use of Web-based Versus App-based Interventions,” “Relationship of Efficacy With Adherence and Engagement” sections |
|  | 20b | Present results of all statistical syntheses conducted. If meta-analysis was done, present for each the summary estimate and its precision (eg, confidence/credible interval) and measures of statistical heterogeneity. If comparing groups, describe the direction of the effect. | N/A |
|  | 20c | Present results of all investigations of possible causes of heterogeneity among study results | N/A |
|  | 20d | Present results of all sensitivity analyses conducted to assess the robustness of the synthesized results | N/A |
| Reporting biases | 21 | Present assessments of risk of bias due to missing results (arising from reporting biases) for each synthesis assessed | N/A |
| Certainty of evidence | 22 | Present assessments of certainty (or confidence) in the body of evidence for each outcome assessed | N/A |
| Discussion | | | |
| Discussion | 23a | Provide a general interpretation of the results in the context of other evidence | Discussion, “Engagement With Digital Interventions for Depression,” “Adherence, Engagement, and Efficacy Based on Whether the Studies Allowed Participants to Access Psychotherapy,” “Adherence and Engagement’s Relationship With Efficacy,” “Underreporting of  Participant Race and Ethnicity Data” sections |
|  | 23b | Discuss any limitations of the evidence included in the review | Discussion, “Limitations and Strengths” section |
|  | 23c | Discuss any limitations of the review  processes used | Discussion, “Limitations and Strengths” section |
|  | 23d | Discuss implications of the results for practice, policy, and future research | Discussion, “Considerations for Clinicians and Patients” section |
| **Other information** | | | |
| Registration and  protocol | 24a | Provide registration information for the review, including register name and registration number, or state that the review was not registered | Methods, “Study  Selection” section |
|  | 24b | Indicate where the review protocol can be accessed, or state that a protocol was not prepared | Methods, “Study  Selection” section |
|  | 24c | Describe and explain any amendments to information provided at registration or in  the protocol | N/A |
| Support | 25 | Describe sources of financial or non-financial support for the review, and the role of the funders or sponsors in the review | Acknowledgements |
| Competing interests | 26 | Declare any competing interests of review authors | Conflicts of Interest |
| Availability of  data, code, and  other materials | 27 | Report which of the following are publicly available and where they can be found: template data collection forms; data extracted from included studies; data used for all analyses; analytic code; any other materials used in the review | Data Availability section |

Adapted from: Page MJ, McKenzie JE, Bossuyt PM, Boutron I, Hoffmann TC, Mulrow CD, et al. The PRISMA 2020 statement: an updated guideline for reporting systematic reviews. BMJ 2021;372:n71. doi: 10.1136/bmj.n71

N/A, not applicable.
