# Supplementary material for: Assessing Patient Adherence to and Engagement With Digital Interventions for Depression in Clinical Trials: Systematic Literature Review
Source: J Med Internet Res. 2023 Aug 11;25:e43727. doi: 10.2196/43727 (PMC10457707; doi:10.2196/43727)
Supplement: Multimedia Appendix 2 [file jmir_v25i1e43727_app2.docx]

**Forbes et al. 2023 Multimedia Appendix 2: Table S2**

Table S2. Outcomes for each reference excluded during abstract and full text screening.

| **Reference** | **Decision From Abstract Screening** | **Decision From Full Text Screening** |
| --- | --- | --- |
| 1 , Sanatkar, S., Heinsch, M., Baldwin, P. A., Rubin, M., Geddes, J., Hunt, S., Baker, A. L., Woodcock, K., Lewin, T. J., Brady, K., Deady, M., Thornton, L., Teesson, M., Kay-Lambkin, F. Factors predicting trial engagement, treatment satisfaction, and health-related quality of life during a web-based treatment and social networking trial for binge drinking and depression in young adults: Secondary analysis of a randomized controlled trial. JMIR Mental Health. 2021. 8:https://mental.jmir.org/2021/6/e23986/PDF https://ovidsp.ovid.com/ovidweb.cgi?T=JS&CSC=Y&NEWS=N&PAGE=fulltext&D=emedx&AN=2013028324 | Include this article |  |
|  |  | Other diagnosis (not depression/MDD) |
| 2 , Everitt, N., Broadbent, J., Richardson, B., Smyth, J. M., Heron, K., Teague, S., Fuller-Tyszkiewicz, M. Exploring the features of an app-based just-in-time intervention for depression. Journal of Affective Disorders. 2021. 291:279-287 , http://www.elsevier.com/locate/jad https://ovidsp.ovid.com/ovidweb.cgi?T=JS&CSC=Y&NEWS=N&PAGE=fulltext&D=emexb&AN=2012259242 | Other diagnosis (not depression/MDD) |  |
| 3 , Sveen, J., Jernelov, S., Pohlkamp, L., Kreicbergs, U., Kaldo, V. Feasibility and preliminary efficacy of guided internet-delivered cognitive behavioral therapy for insomnia after the loss of a child to cancer: Randomized controlled trial. Internet Interventions. 2021. 25 (no pagination):http://www.journals.elsevier.com/internet-interventions/ https://ovidsp.ovid.com/ovidweb.cgi?T=JS&CSC=Y&NEWS=N&PAGE=fulltext&D=emexb&AN=2012834528 | Other diagnosis (not depression/MDD) |  |
| 4 , Jang, S., Kim, J. J., Kim, S. J., Hong, J., Kim, S., Kim, E. Mobile app-based chatbot to deliver cognitive behavioral therapy and psychoeducation for adults with attention deficit: A development and feasibility/usability study. International Journal of Medical Informatics. 2021. 150 (no pagination):http://www.elsevier.com/inca/publications/store/5/0/6/0/4/0/ https://ovidsp.ovid.com/ovidweb.cgi?T=JS&CSC=Y&NEWS=N&PAGE=fulltext&D=emexb&AN=2011536711 | Other diagnosis (not depression/MDD) |  |
| 5 , Young, C. L., Mohebbi, M., Staudacher, H., Berk, M., Jacka, F. N., O'Neil, A. Assessing the feasibility of an m-Health intervention for changing diet quality and mood in individuals with depression: the My Food & Mood program. International Review of Psychiatry. 2021. :http://www.tandfonline.com/loi/iirp20 https://ovidsp.ovid.com/ovidweb.cgi?T=JS&CSC=Y&NEWS=N&PAGE=fulltext&D=emexb&AN=2011635216 |  | Inappropriate intervention |
|  | Include this article |  |
| 6 , Graham, A. K., Kwasny, M. J., Lattie, E. G., Greene, C. J., Gupta, N. V., Reddy, M., Mohr, D. C. Targeting subjective engagement in experimental therapeutics for digital mental health interventions. Internet Interventions. 2021. 25 (no pagination):http://www.journals.elsevier.com/internet-interventions/ https://ovidsp.ovid.com/ovidweb.cgi?T=JS&CSC=Y&NEWS=N&PAGE=fulltext&D=emexb&AN=2012136654 | Inappropriate study design |  |
| 7 , Schouten, M. J. E., Dekker, J. J. M., de Bruijn, T. Q., Ebert, D. D., Koomen, L. M., Kosterman, S. L. A., Riper, H., Schaub, M. P., Goudriaan, A. E., Blankers, M. Effectiveness of a digital alcohol moderation intervention as an add-on to depression treatment for young adults: study protocol of a multicentre pragmatic randomized controlled trial. BMC Psychiatry. 2021. 21:https://bmcpsychiatry.biomedcentral.com/ https://ovidsp.ovid.com/ovidweb.cgi?T=JS&CSC=Y&NEWS=N&PAGE=fulltext&D=emexb&AN=2011857766 |  | Inappropriate intervention |
|  | Include this article |  |
| 8 , Hunt, M., Miguez, S., Dukas, B., Onwude, O., White, S. Efficacy of Zemedy, a Mobile Digital Therapeutic for the Self-management of Irritable Bowel Syndrome: Crossover Randomized Controlled Trial. JMIR mHealth and uHealth. 2021. 9:e26152 , https://ovidsp.ovid.com/ovidweb.cgi?T=JS&CSC=Y&NEWS=N&PAGE=fulltext&D=emexb&AN=634843444 | Other diagnosis (not depression/MDD) |  |
| 9 , Alvarez-Jimenez, M., Koval, P., Schmaal, L., Bendall, S., O'Sullivan, S., Cagliarini, D., D'Alfonso, S., Rice, S., Valentine, L., Penn, D. L., Miles, C., Russon, P., Phillips, J., McEnery, C., Lederman, R., Killackey, E., Mihalopoulos, C., Gonzalez-Blanch, C., Gilbertson, T., Lal, S., Cotton, S. M., Herrman, H., McGorry, P. D., Gleeson, J. F. M. The Horyzons project: a randomized controlled trial of a novel online social therapy to maintain treatment effects from specialist first-episode psychosis services. World Psychiatry. 2021. 20:233-243 , http://onlinelibrary.wiley.com/journal/10.1002/(ISSN)2051-5545 https://ovidsp.ovid.com/ovidweb.cgi?T=JS&CSC=Y&NEWS=N&PAGE=fulltext&D=emexb&AN=2011526331 | Other diagnosis (not depression/MDD) |  |
| 10 , Batterham, P. J., Calear, A. L., Farrer, L., Gulliver, A., Kurz, E. Efficacy of a Transdiagnostic Self-Help Internet Intervention for Reducing Depression, Anxiety, and Suicidal Ideation in Adults: Randomized Controlled Trial. Journal of Medical Internet Research. 2021. 23:https://www.jmir.org/2021/1/e22698/PDF https://ovidsp.ovid.com/ovidweb.cgi?T=JS&CSC=Y&NEWS=N&PAGE=fulltext&D=emexb&AN=2010813363 |  | Other diagnosis (not depression/MDD) |
|  | Include this article |  |
| 11 , Si, M. Y., Xiao, W. J., Pan, C., Wang, H., Huang, Y. M., Lian, J., Mak, W. W. S., Leng, Z. W., Su, X. Y., Tang, Q. P., Jiang, Y., Feng, L. Z., Yang, W. Z., Wang, C. Mindfulness-based online intervention on mental health and quality of life among COVID-19 patients in China: an intervention design. Infectious Diseases of Poverty. 2021. 10:http://www.idpjournal.com/ https://ovidsp.ovid.com/ovidweb.cgi?T=JS&CSC=Y&NEWS=N&PAGE=fulltext&D=emexb&AN=2011573033 | Other diagnosis (not depression/MDD) |  |
| 12 , Beukes, E. W., Andersson, G., Fagelson, M. A., Manchaiah, V. Dismantling internet-based cognitive behavioral therapy for tinnitus. The contribution of applied relaxation: A randomized controlled trial. Internet Interventions. 2021. 25 (no pagination):http://www.journals.elsevier.com/internet-interventions/ https://ovidsp.ovid.com/ovidweb.cgi?T=JS&CSC=Y&NEWS=N&PAGE=fulltext&D=emexb&AN=2012042388 | Other diagnosis (not depression/MDD) |  |
| 13 , Schlosser, D. A., Campellone, T. R., Truong, B., Etter, K., Vergani, S., Komaiko, K., Vinogradov, S. Efficacy of PRIME, a mobile app intervention designed to improve motivation in young people with schizophrenia. Schizophrenia Bulletin. 2018. 44:1010-1020 , http://schizophreniabulletin.oxfordjournals.org/ https://ovidsp.ovid.com/ovidweb.cgi?T=JS&CSC=Y&NEWS=N&PAGE=fulltext&D=emexb&AN=624420876 | Other diagnosis (not depression/MDD) |  |
| 14 , Bove, R. M., Rush, G., Zhao, C., Rowles, W., Garcha, P., Morrissey, J., Schembri, A., Alailima, T., Langdon, D., Possin, K., Gazzaley, A., Feinstein, A., Anguera, J. A videogame-based digital therapeutic to improve processing speed in people with multiple sclerosis: A feasibility study. Neurology and Therapy. 2019. 8:135-145 , http://www.springer.com/springer+healthcare/journal/40120 https://ovidsp.ovid.com/ovidweb.cgi?T=JS&CSC=Y&NEWS=N&PAGE=fulltext&D=emexb&AN=2005287531 | Other diagnosis (not depression/MDD) |  |
| 15 , Hess Engstrom, A. H., Kullinger, M., Jawad, I., Hesselman, S., Buhrman, M., Hogberg, U., Skalkidou, A. Internet-based treatment for vulvodynia (EMBLA) - Study protocol for a randomised controlled study. Internet Interventions. 2021. 25 (no pagination):http://www.journals.elsevier.com/internet-interventions/ https://ovidsp.ovid.com/ovidweb.cgi?T=JS&CSC=Y&NEWS=N&PAGE=fulltext&D=emexb&AN=2011767841 | Other diagnosis (not depression/MDD) |  |
| 17 , Heffner, J. L., Mull, K. E., Watson, N. L., McClure, J. B., Bricker, J. B. Long-Term smoking cessation outcomes for sexual minority versus nonminority smokers in a large randomized controlled trial of two web-based interventions. Nicotine and Tobacco Research. 2020. 22:1596-1604 , http://ntr.oxfordjournals.org/ https://ovidsp.ovid.com/ovidweb.cgi?T=JS&CSC=Y&NEWS=N&PAGE=fulltext&D=emexb&AN=2010688401 | Other diagnosis (not depression/MDD) |  |
| 19 , Bove, R., Rowles, W., Zhao, C., Anderson, A., Friedman, S., Langdon, D., Alexander, A., Sacco, S., Henry, R., Gazzaley, A., Feinstein, A., Anguera, J. A. A novel in-home digital treatment to improve processing speed in people with multiple sclerosis: A pilot study. Multiple Sclerosis Journal. 2021. 27:778-789 , http://msj.sagepub.com/ https://ovidsp.ovid.com/ovidweb.cgi?T=JS&CSC=Y&NEWS=N&PAGE=fulltext&D=emexb&AN=2005369993 | Other diagnosis (not depression/MDD) |  |
| 20 , Huang, L., Shen, Q., Fang, Q., Zheng, X. Effects of internet-based support program on parenting outcomes for primiparous women: A pilot study. International Journal of Environmental Research and Public Health. 2021. 18:https://www.mdpi.com/1660-4601/18/9/4402/pdf https://ovidsp.ovid.com/ovidweb.cgi?T=JS&CSC=Y&NEWS=N&PAGE=fulltext&D=emexb&AN=2006997812 | Other diagnosis (not depression/MDD) |  |
| 21 , Zeng, Y., Guo, Y., Li, L., Hong, Y. A., Li, Y., Zhu, M., Zeng, C., Zhang, H., Cai, W., Liu, C., Wu, S., Chi, P., Monroe-Wise, A., Hao, Y., Ho, R. T. H. Relationship Between Patient Engagement and Depressive Symptoms Among People Living With HIV in a Mobile Health Intervention: Secondary Analysis of a Randomized Controlled Trial. JMIR mHealth and uHealth. 2020. 8:e20847 , https://ovidsp.ovid.com/ovidweb.cgi?T=JS&CSC=Y&NEWS=N&PAGE=fulltext&D=emexb&AN=633295182 | Include this article |  |
| 21 , Zeng, Y., Guo, Y., Li, L., Hong, Y. A., Li, Y., Zhu, M., Zeng, C., Zhang, H., Cai, W., Liu, C., Wu, S., Chi, P., Monroe-Wise, A., Hao, Y., Ho, R. T. H. Relationship Between Patient Engagement and Depressive Symptoms Among People Living With HIV in a Mobile Health Intervention: Secondary Analysis of a Randomized Controlled Trial. JMIR mHealth and uHealth. 2020. 8:e20847 , https://ovidsp.ovid.com/ovidweb.cgi?T=JS&CSC=Y&NEWS=N&PAGE=fulltext&D=emexb&AN=633295182 |  | Other diagnosis (not depression/MDD) |
| 22 , Cillessen, L., van de Ven, M. O., Compen, F. R., Bisseling, E. M., van der Lee, M. L., Speckens, A. E. M. Predictors and effects of usage of an online mindfulness intervention for distressed cancer patients: Usability study. Journal of Medical Internet Research. 2020. 22:https://www.jmir.org/2020/10/e17526 https://ovidsp.ovid.com/ovidweb.cgi?T=JS&CSC=Y&NEWS=N&PAGE=fulltext&D=emexb&AN=2008401815 | Other diagnosis (not depression/MDD) |  |
| 23 , Ponzo, S., Morelli, D., Kawadler, J. M., Hemmings, N. R., Bird, G., Plans, D. Efficacy of the Digital Therapeutic Mobile App BioBase to Reduce Stress and Improve Mental Well-Being Among University Students: Randomized Controlled Trial. JMIR mHealth and uHealth. 2020. 8:e17767 , https://ovidsp.ovid.com/ovidweb.cgi?T=JS&CSC=Y&NEWS=N&PAGE=fulltext&D=emexb&AN=630593993 |  | Other diagnosis (not depression/MDD) |
|  | Include this article |  |
| 24 , Sesel, A. L., Sharpe, L., Beadnall, H. N., Barnett, M. H., Szabo, M., Naismith, S. L. Development of a web-based mindfulness program for people with multiple sclerosis: Qualitative co-design study. Journal of Medical Internet Research. 2021. 23:https://www.jmir.org/2021/3/e19309/PDF https://ovidsp.ovid.com/ovidweb.cgi?T=JS&CSC=Y&NEWS=N&PAGE=fulltext&D=emexb&AN=2011414192 | Other diagnosis (not depression/MDD) |  |
| 26 , Baldwin, P. A., Sanatkar, S., Clarke, J., Fletcher, S., Gunn, J., Wilhelm, K., Campbell, L., Zwar, N., Harris, M., Lapsley, H., Hadzi-Pavlovic, D., Christensen, H., Proudfoot, J. A web-based mental health intervention to improve social and occupational functioning in adults with type 2 diabetes (the springboard trial): 12-month outcomes of a randomized controlled trial. Journal of Medical Internet Research. 2020. 22:https://www.jmir.org/2020/12/e16729/PDF https://ovidsp.ovid.com/ovidweb.cgi?T=JS&CSC=Y&NEWS=N&PAGE=fulltext&D=emexb&AN=2010270961 | Other diagnosis (not depression/MDD) |  |
| 27 , Moritz, S., Goritz, A. S., Kraj, M., Gehlenborg, J., Hottenrott, B., Tonn, P., Ascone, L., Pedersen, A., Kuhn, S. Imaginal Retraining Reduces Cigarette Smoking: A Randomized Controlled Study. European Addiction Research. 2020. 26:355-364 , http://www.karger.com/journals/ear/ear_jh.htm https://ovidsp.ovid.com/ovidweb.cgi?T=JS&CSC=Y&NEWS=N&PAGE=fulltext&D=emexb&AN=632869039 | Other diagnosis (not depression/MDD) |  |
| 28 , Sin, J., Galeazzi, G., McGregor, E., Collom, J., Taylor, A., Barrett, B., Lawrence, V., Henderson, C. Digital interventions for screening and treating common mental disorders or symptoms of common mental illness in adults: Systematic review and meta-analysis. Journal of Medical Internet Research. 2020. 22:https://www.jmir.org/2020/9/e20581/pdf https://ovidsp.ovid.com/ovidweb.cgi?T=JS&CSC=Y&NEWS=N&PAGE=fulltext&D=emexb&AN=2010074831 | Inappropriate study design |  |
| 29 , Callan, J. A., Dunbar Jacob, J., Siegle, G. J., Dey, A., Thase, M. E., DeVito Dabbs, A., Kazantzis, N., Rotondi, A., Tamres, L., Van Slyke, A., Sereika, S. CBT MobileWork©: User-Centered Development and Testing of a Mobile Mental Health Application for Depression. Cognitive Therapy and Research. 2021. 45:287-302 , https://www.springer.com/medicine/journal/10608 https://ovidsp.ovid.com/ovidweb.cgi?T=JS&CSC=Y&NEWS=N&PAGE=fulltext&D=emexb&AN=2006794501 | Include this article |  |
|  |  | Inappropriate study design |
| 30 , Sunnhed, R., Hesser, H., Andersson, G., Carlbring, P., Morin, C. M., Harvey, A. G., Jansson-Frojmark, M. Comparing internet-delivered cognitive therapy and behavior therapy with telephone support for insomnia disorder: A randomized controlled trial. Sleep. 2020. 43:https://academic.oup.com/sleep https://ovidsp.ovid.com/ovidweb.cgi?T=JS&CSC=Y&NEWS=N&PAGE=fulltext&D=emexb&AN=2010099651 | Other diagnosis (not depression/MDD) |  |
| 33 , Kahl, B. L., Miller, H. M., Cairns, K., Giniunas, H., Nicholas, M. Evaluation of ReachOut.com, an unstructured digital youth mental health intervention: Prospective cohort study. JMIR Mental Health. 2020. 7:https://mental.jmir.org/2020/10/e21280/PDF https://ovidsp.ovid.com/ovidweb.cgi?T=JS&CSC=Y&NEWS=N&PAGE=fulltext&D=emexb&AN=2010268252 | Include this article |  |
| 33 , Kahl, B. L., Miller, H. M., Cairns, K., Giniunas, H., Nicholas, M. Evaluation of ReachOut.com, an unstructured digital youth mental health intervention: Prospective cohort study. JMIR Mental Health. 2020. 7:https://mental.jmir.org/2020/10/e21280/PDF https://ovidsp.ovid.com/ovidweb.cgi?T=JS&CSC=Y&NEWS=N&PAGE=fulltext&D=emexb&AN=2010268252 |  | Other diagnosis (not depression/MDD) |
| 34 , Chavez, L. J., Kelleher, K., Slesnick, N., Holowacz, E., Luthy, E., Moore, L., Ford, J. Virtual reality meditation among youth experiencing homelessness: Pilot randomized controlled trial of feasibility. JMIR Mental Health. 2020. 7:https://mental.jmir.org/2020/9/e18244/PDF https://ovidsp.ovid.com/ovidweb.cgi?T=JS&CSC=Y&NEWS=N&PAGE=fulltext&D=emexb&AN=2010268247 | Other diagnosis (not depression/MDD) |  |
| 35 , Prochaska, J. J., Vogel, E. A., Chieng, A., Kendra, M., Baiocchi, M., Pajarito, S., Robinson, A. A therapeutic relational agent for reducing problematic substance use (Woebot): Development and usability study. Journal of Medical Internet Research. 2021. 23:https://www.jmir.org/2021/3/e24850/PDF https://ovidsp.ovid.com/ovidweb.cgi?T=JS&CSC=Y&NEWS=N&PAGE=fulltext&D=emexb&AN=2011598192 | Other diagnosis (not depression/MDD) |  |
| 36 , Andriopoulos, A., Olsson, E. M. G., Sylven, Y. H., Sjostrom, J., Johansson, B., von Essen, L., Gronqvist, H. Commencement of and retention in web-based interventions and response to prompts and reminders: Longitudinal observational study based on two randomized controlled trials. Journal of Medical Internet Research. 2021. 23:https://www.jmir.org/2021/3/e24590/PDF https://ovidsp.ovid.com/ovidweb.cgi?T=JS&CSC=Y&NEWS=N&PAGE=fulltext&D=emexb&AN=2011536584 | Other diagnosis (not depression/MDD) |  |
| 37 , Wang, H., Zhao, Q., Mu, W., Rodriguez, M., Qian, M., Berger, T. The effect of shame on patients with social anxiety disorder in internet-based cognitive behavioral therapy: Randomized controlled trial. JMIR Mental Health. 2020. 7:https://mental.jmir.org/2020/7/e15797/PDF https://ovidsp.ovid.com/ovidweb.cgi?T=JS&CSC=Y&NEWS=N&PAGE=fulltext&D=emexb&AN=2010268217 | Other diagnosis (not depression/MDD) |  |
| 38 , Walter, F. M., Pannebakker, M. M., Barclay, M. E., Mills, K., Saunders, C. L., Murchie, P., Corrie, P., Hall, P., Burrows, N., Emery, J. D. Effect of a Skin Self-monitoring Smartphone Application on Time to Physician Consultation among Patients with Possible Melanoma: A Phase 2 Randomized Clinical Trial. JAMA Network Open. 2020. 3:https://jamanetwork.com/journals/jamanetworkopen https://ovidsp.ovid.com/ovidweb.cgi?T=JS&CSC=Y&NEWS=N&PAGE=fulltext&D=emexb&AN=631074140 | Other diagnosis (not depression/MDD) |  |
| 39 , Casey Orr, L., Graham, A. K., Mohr, D. C., Greene, C. J. Engagement and clinical improvement among older adult primary care patients using a mobile intervention for depression and anxiety: Case studies. JMIR Mental Health. 2020. 7:https://mental.jmir.org/2020/7/e16341/PDF https://ovidsp.ovid.com/ovidweb.cgi?T=JS&CSC=Y&NEWS=N&PAGE=fulltext&D=emexb&AN=2010268206 | Include this article |  |
|  |  | Inappropriate study design |
| 40 , McCloud, T., Jones, R., Lewis, G., Bell, V., Tsakanikos, E. Effectiveness of a Mobile App Intervention for Anxiety and Depression Symptoms in University Students: Randomized Controlled Trial. JMIR mHealth and uHealth. 2020. 8:e15418 , https://ovidsp.ovid.com/ovidweb.cgi?T=JS&CSC=Y&NEWS=N&PAGE=fulltext&D=emexb&AN=632499491 | Include this article |  |
| 40 , McCloud, T., Jones, R., Lewis, G., Bell, V., Tsakanikos, E. Effectiveness of a Mobile App Intervention for Anxiety and Depression Symptoms in University Students: Randomized Controlled Trial. JMIR mHealth and uHealth. 2020. 8:e15418 , https://ovidsp.ovid.com/ovidweb.cgi?T=JS&CSC=Y&NEWS=N&PAGE=fulltext&D=emexb&AN=632499491 |  | Other diagnosis (not depression/MDD) |
| 41 , Gladstone, T. R. G., Terrizzi, D. A., Paulson, A., Nidetz, J., Canel, J., Ching, E., Berry, A. D., Cantorna, J., Fogel, J., Eder, M., Bolotin, M., Thomann, L. O., Griffiths, K., Ip, P., Aaby, D. A., Brown, C. H., Beardslee, W., Bell, C., Crawford, T. J., Fitzgibbon, M., Schiffer, L., Liu, N., Marko-Holguin, M., Van Voorhees, B. W. Effect of Internet-Based Cognitive Behavioral Humanistic and Interpersonal Training vs Internet-Based General Health Education on Adolescent Depression in Primary Care: A Randomized Clinical Trial. JAMA Network Open. 2018. 1:https://jamanetwork.com/journals/jamanetworkopen https://ovidsp.ovid.com/ovidweb.cgi?T=JS&CSC=Y&NEWS=N&PAGE=fulltext&D=emexb&AN=634471774 | Inappropriate study design |  |
| 43 , Schroder, J., Werkle, N., Cludius, B., Jelinek, L., Moritz, S., Westermann, S. Unguided Internet-based cognitive-behavioral therapy for obsessive-compulsive disorder: A randomized controlled trial. Depression and Anxiety. 2020. 37:1208-1220 , http://onlinelibrary.wiley.com/journal/10.1002/(ISSN)1520-6394 https://ovidsp.ovid.com/ovidweb.cgi?T=JS&CSC=Y&NEWS=N&PAGE=fulltext&D=emexb&AN=2007423066 | Other diagnosis (not depression/MDD) |  |
| 44 , Lambert, S. D., Duncan, L. R., Ellis, J., Robinson, J. W., Sears, C., Culos-Reed, N., Matthew, A., De Raad, M., Schaffler, J. L., Mina, D. S., Saha-Chaudhuri, P., McTaggart-Cowan, H., Peacock, S. A study protocol for a multicenter randomized pilot trial of a dyadic, tailored, web-based, psychosocial, and physical activity self-management program (TEMPO) for men with prostate cancer and their caregivers. Pilot and Feasibility Studies. 2021. 7:https://pilotfeasibilitystudies.biomedcentral.com/ https://ovidsp.ovid.com/ovidweb.cgi?T=JS&CSC=Y&NEWS=N&PAGE=fulltext&D=emexb&AN=2010852472 | Other diagnosis (not depression/MDD) |  |
| 45 , Li, J., Mo, P. K. H., Kahler, C. W., Lau, J. T. F. A three-arm randomised controlled trial to evaluate the efficacy of a positive psychology and social networking intervention in promoting mental health among HIV-infected men who have sex with men in China. Epidemiology and Psychiatric Sciences. 2021. (no pagination):http://journals.cambridge.org/action/displayJournal?jid=EPS https://ovidsp.ovid.com/ovidweb.cgi?T=JS&CSC=Y&NEWS=N&PAGE=fulltext&D=emexb&AN=634560132 | Other diagnosis (not depression/MDD) |  |
| 47 , Kim, D. Y., Kwon, H., Nam, K. W., Lee, Y., Kwon, H. M., Chung, Y. S. Remote management of poststroke patients with a smartphone-based management system integrated in clinical care: Prospective, nonrandomized, interventional study. Journal of Medical Internet Research. 2020. 22:https://www.jmir.org/2020/2/e15377/pdf https://ovidsp.ovid.com/ovidweb.cgi?T=JS&CSC=Y&NEWS=N&PAGE=fulltext&D=emexb&AN=2010074778 | Other diagnosis (not depression/MDD) |  |
| 48 , Eylem, O., van Straten, A., de Wit, L., Rathod, S., Bhui, K., Kerkhof, A. J. F. M. Reducing suicidal ideation among Turkish migrants in the Netherlands and in the UK: the feasibility of a randomised controlled trial of a guided online intervention. Pilot and Feasibility Studies. 2021. 7:https://pilotfeasibilitystudies.biomedcentral.com/ https://ovidsp.ovid.com/ovidweb.cgi?T=JS&CSC=Y&NEWS=N&PAGE=fulltext&D=emexb&AN=2010258073 | Other diagnosis (not depression/MDD) |  |
| 49 , Hallford, D. J., Austin, D. W., Takano, K., Fuller-Tyszkiewicz, M., Raes, F. Computerized Memory Specificity Training (c-MeST) for major depression: A randomised controlled trial. Behaviour Research and Therapy. 2021. 136 (no pagination):http://www.elsevier.com/locate/brat https://ovidsp.ovid.com/ovidweb.cgi?T=JS&CSC=Y&NEWS=N&PAGE=fulltext&D=emexb&AN=2010249408 | Include this article |  |
| 49 , Hallford, D. J., Austin, D. W., Takano, K., Fuller-Tyszkiewicz, M., Raes, F. Computerized Memory Specificity Training (c-MeST) for major depression: A randomised controlled trial. Behaviour Research and Therapy. 2021. 136 (no pagination):http://www.elsevier.com/locate/brat https://ovidsp.ovid.com/ovidweb.cgi?T=JS&CSC=Y&NEWS=N&PAGE=fulltext&D=emexb&AN=2010249408 |  | Inappropriate outcomes |
| 51 , Niles, A. N., Axelsson, E., Andersson, E., Hedman-Lagerlof, E., Carlbring, P., Andersson, G., Johansson, R., Widen, S., Driessen, J., Santoft, F., Ljotsson, B. Internet-based cognitive behavior therapy for depression, social anxiety disorder, and panic disorder: Effectiveness and predictors of response in a teaching clinic. Behaviour Research and Therapy. 2021. 136 (no pagination):http://www.elsevier.com/locate/brat https://ovidsp.ovid.com/ovidweb.cgi?T=JS&CSC=Y&NEWS=N&PAGE=fulltext&D=emexb&AN=2010110567 | Include this article |  |
|  |  | Inappropriate intervention |
| 53 , Liu, G., Wang, S., Liao, J., Ou, P., Huang, L., Xie, N., He, Y., Lin, J., He, H. G., Hu, R. The efficacy of wechat-based parenting training on the psychological well-being of mothers with children with autism during the COVID-19 pandemic: Quasi-experimental study. JMIR Mental Health. 2021. 8:https://mental.jmir.org/2021/2/e23917/PDF https://ovidsp.ovid.com/ovidweb.cgi?T=JS&CSC=Y&NEWS=N&PAGE=fulltext&D=emexb&AN=2011094841 | Other diagnosis (not depression/MDD) |  |
| 54 , Thompson, E. M., Destree, L., Albertella, L., Fontenelle, L. F. Internet-Based Acceptance and Commitment Therapy: A Transdiagnostic Systematic Review and Meta-Analysis for Mental Health Outcomes. Behavior Therapy. 2021. 52:492-507 , http://www.journals.elsevier.com/behavior-therapy/ https://ovidsp.ovid.com/ovidweb.cgi?T=JS&CSC=Y&NEWS=N&PAGE=fulltext&D=emexb&AN=2007794730 | Inappropriate study design |  |
| 55 , Kohle, N., Drossaert, C. H. C., ten Klooster, P. M., Schreurs, K. M. G., Hagedoorn, M., Van Uden-Kraan, C. F., Verdonck-de Leeuw, I. M., Bohlmeijer, E. T. Web-based self-help intervention for partners of cancer patients based on acceptance and commitment therapy and self-compassion training: a randomized controlled trial with automated versus personal feedback. Supportive Care in Cancer. 2021. :http://link.springer.de/link/service/journals/00520/index.htm https://ovidsp.ovid.com/ovidweb.cgi?T=JS&CSC=Y&NEWS=N&PAGE=fulltext&D=emexb&AN=2010536739 | Other diagnosis (not depression/MDD) |  |
| 57 , Baggett, K. M., Davis, B., Sheeber, L. B., Ammerman, R. T., Mosley, E. A., Miller, K., Feil, E. G. Minding the gatekeepers: Referral and recruitment of postpartum mothers with depression into a randomized controlled trial of a mobile internet parenting intervention to improve mood and optimize infant social communication outcomes. International Journal of Environmental Research and Public Health. 2020. 17:1-13 , https://www.mdpi.com/1660-4601/17/23/8978/pdf https://ovidsp.ovid.com/ovidweb.cgi?T=JS&CSC=Y&NEWS=N&PAGE=fulltext&D=emexb&AN=2005535804 | Include this article |  |
|  |  | Inappropriate study design |
| 58 , Bossen, D., Veenhof, C., Van Beek, K. E., Spreeuwenberg, P. M., Dekker, J., De Bakker, D. H. Effectiveness of a web-based physical activity intervention in patients with knee and/or hip osteoarthritis: randomized controlled trial. Journal of medical Internet research. 2013. 15:e257 , https://ovidsp.ovid.com/ovidweb.cgi?T=JS&CSC=Y&NEWS=N&PAGE=fulltext&D=emexb&AN=563067785 | Other diagnosis (not depression/MDD) |  |
| 59 , Kalmbach, D. A., Cheng, P., O'Brien, L. M., Swanson, L. M., Sangha, R., Sen, S., Guille, C., Cuamatzi-Castelan, A., Henry, A. L., Roth, T., Drake, C. L. A randomized controlled trial of digital cognitive behavioral therapy for insomnia in pregnant women. Sleep Medicine. 2020. 72:82-92 , http://www.elsevier.com/inca/publications/store/6/2/0/2/8/2 https://ovidsp.ovid.com/ovidweb.cgi?T=JS&CSC=Y&NEWS=N&PAGE=fulltext&D=emexb&AN=2006742809 | Other diagnosis (not depression/MDD) |  |
| 61 , Newcombe, P. A., Dunn, T. L., Casey, L. M., Sheffield, J. K., Petsky, H., Anderson-James, S., Chang, A. B. Breathe Easier Online: evaluation of a randomized controlled pilot trial of an Internet-based intervention to improve well-being in children and adolescents with a chronic respiratory condition. Journal of medical Internet research. 2012. 14:e23 , https://ovidsp.ovid.com/ovidweb.cgi?T=JS&CSC=Y&NEWS=N&PAGE=fulltext&D=emexb&AN=364731717 | Other diagnosis (not depression/MDD) |  |
| 62 , Kuchler, A. M., Schultchen, D., Pollatos, O., Moshagen, M., Ebert, D. D., Baumeister, H. StudiCare mindfulness-study protocol of a randomized controlled trial evaluating an internet- and mobile-based intervention for college students with no and "on demand" guidance. Trials. 2020. 21:http://www.trialsjournal.com/home/ https://ovidsp.ovid.com/ovidweb.cgi?T=JS&CSC=Y&NEWS=N&PAGE=fulltext&D=emexb&AN=2007391105 | Other diagnosis (not depression/MDD) |  |
| 63 , Huang, F., Wu, X., Xie, Y., Liu, F., Li, J., Li, X., Zhou, Z. An automated structured education intervention based on a smartphone app in Chinese patients with type 1 diabetes: a protocol for a single-blinded randomized controlled trial. Trials. 2020. 21:http://www.trialsjournal.com/home/ https://ovidsp.ovid.com/ovidweb.cgi?T=JS&CSC=Y&NEWS=N&PAGE=fulltext&D=emexb&AN=2007355102 | Other diagnosis (not depression/MDD) |  |
| 64 , Gomez Penedo, J. M., Berger, T., Grosse Holtforth, M., Krieger, T., Schroder, J., Hohagen, F., Meyer, B., Moritz, S., Klein, J. P. The Working Alliance Inventory for guided Internet interventions (WAI-I). Journal of clinical psychology. 2020. 76:973-986 , https://ovidsp.ovid.com/ovidweb.cgi?T=JS&CSC=Y&NEWS=N&PAGE=fulltext&D=emexb&AN=628515684 | Inappropriate study design |  |
| 65 , Kessler, D., Lewis, G., Kaur, S., Wiles, N., King, M., Weich, S., Sharp, D. J., Araya, R., Hollinghurst, S., Peters, T. J. Therapist-delivered internet psychotherapy for depression in primary care: a randomised controlled trial. The Lancet. 2009. 374:628-634 , http://www.journals.elsevier.com/the-lancet/ https://ovidsp.ovid.com/ovidweb.cgi?T=JS&CSC=Y&NEWS=N&PAGE=fulltext&D=emexb&AN=355110270 | Inappropriate intervention |  |
| 66 , Blair, M., Goveas, D., Safi, A., Marshall, C., Rosehart, H., Orenczuk, S., Morrow, S. A. Does cognitive training improve attention/working memory in persons with MS? A pilot study using the Cogmed Working Memory Training program. Multiple Sclerosis and Related Disorders. 2021. 49 (no pagination):http://www.elsevier.com/wps/find/journaldescription.cws_home/725776/description#description https://ovidsp.ovid.com/ovidweb.cgi?T=JS&CSC=Y&NEWS=N&PAGE=fulltext&D=emexb&AN=2010770386 | Other diagnosis (not depression/MDD) |  |
| 67 , Swiatkiewicz, I., Mila-Kierzenkowska, C., Wozniak, A., Szewczyk-Golec, K., Nuszkiewicz, J., Wroblewska, J., Rajewski, P., Eussen, S. J. P. M., Faerch, K., Manoogian, E. N. C., Panda, S., Taub, P. R. Pilot clinical trial of time-restricted eating in patients with metabolic syndrome. Nutrients. 2021. 13:1-18 , https://www.mdpi.com/2072-6643/13/2/346/pdf https://ovidsp.ovid.com/ovidweb.cgi?T=JS&CSC=Y&NEWS=N&PAGE=fulltext&D=emexb&AN=2005866663 | Other diagnosis (not depression/MDD) |  |
| 68 , Pedersen, S. S., Andersen, C. M., Ahm, R., Skovbakke, S. J., Kok, R., Helmark, C., Wiil, U. K., Schmidt, T., Olsen, K. R., Hjelmborg, J., Zwisler, A. D., Frostholm, L. Efficacy and cost-effectiveness of a therapist-assisted web-based intervention for depression and anxiety in patients with ischemic heart disease attending cardiac rehabilitation [eMindYourHeart trial]: a randomised controlled trial protocol. BMC Cardiovascular Disorders. 2021. 21:http://www.biomedcentral.com/bmccardiovascdisord/ https://ovidsp.ovid.com/ovidweb.cgi?T=JS&CSC=Y&NEWS=N&PAGE=fulltext&D=emexb&AN=2010128365 | Include this article |  |
| 68 , Pedersen, S. S., Andersen, C. M., Ahm, R., Skovbakke, S. J., Kok, R., Helmark, C., Wiil, U. K., Schmidt, T., Olsen, K. R., Hjelmborg, J., Zwisler, A. D., Frostholm, L. Efficacy and cost-effectiveness of a therapist-assisted web-based intervention for depression and anxiety in patients with ischemic heart disease attending cardiac rehabilitation [eMindYourHeart trial]: a randomised controlled trial protocol. BMC Cardiovascular Disorders. 2021. 21:http://www.biomedcentral.com/bmccardiovascdisord/ https://ovidsp.ovid.com/ovidweb.cgi?T=JS&CSC=Y&NEWS=N&PAGE=fulltext&D=emexb&AN=2010128365 |  | Other diagnosis (not depression/MDD) |
| 69 , Yuan, J., Chen, Y., Yu, P., Luo, F., Gao, Y., Chen, J., Wang, P., Wang, Y., Zhao, Y., Lei, Y. Effect of magnetic stimulation of Shenmen point on cognitive function of chronic insomnia: A randomized controlled clinical trial. Medicine. 2020. 99:e23807 , https://ovidsp.ovid.com/ovidweb.cgi?T=JS&CSC=Y&NEWS=N&PAGE=fulltext&D=emexb&AN=633895903 | Other diagnosis (not depression/MDD) |  |
| 70 , Kageyama, K., Kato, Y., Mesaki, T., Uchida, H., Takahashi, K., Marume, R., Sejima, Y., Hirao, K. Effects of video viewing smartphone application intervention involving positive word stimulation in people with subthreshold depression: A pilot randomized controlled trial. Journal of Affective Disorders. 2021. 282:74-81 , http://www.elsevier.com/locate/jad https://ovidsp.ovid.com/ovidweb.cgi?T=JS&CSC=Y&NEWS=N&PAGE=fulltext&D=emexb&AN=2010535671 | Include this article |  |
|  |  | Other diagnosis (not depression/MDD) |
| 71 , Jiao, N., Zhu, L., Chong, Y. S., Chan, W. C. S., Luo, N., Wang, W., Hu, R., Chan, Y. H., He, H. G. Web-based versus home-based postnatal psychoeducational interventions for first-time mothers: A randomised controlled trial. International journal of nursing studies. 2019. 99:103385 , https://ovidsp.ovid.com/ovidweb.cgi?T=JS&CSC=Y&NEWS=N&PAGE=fulltext&D=emexb&AN=629164720 | Other diagnosis (not depression/MDD) |  |
| 72 , Barrett, K., Stewart, I. A preliminary comparison of the efficacy of online Acceptance and Commitment Therapy (ACT) and Cognitive Behavioural Therapy (CBT) stress management interventions for social and healthcare workers. Health & social care in the community. 2021. 29:113-126 , https://ovidsp.ovid.com/ovidweb.cgi?T=JS&CSC=Y&NEWS=N&PAGE=fulltext&D=emexb&AN=632250219 | Other diagnosis (not depression/MDD) |  |
| 73 , Lien, M., Bredeli, E., Sivertsen, B., Kallestad, H., Pallesen, S., Smith, O. R. F., Faaland, P., Ritterband, L. M., Thorndike, F. P., Vedaa, O. Short and long-term effects of unguided internet-based cognitive behavioral therapy for chronic insomnia in morning and evening persons: a post-hoc analysis. Chronobiology international. 2019. 36:1384-1398 , https://ovidsp.ovid.com/ovidweb.cgi?T=JS&CSC=Y&NEWS=N&PAGE=fulltext&D=emexb&AN=628980798 | Other diagnosis (not depression/MDD) |  |
| 74 , Whitehead, L., Emery, L., Kirk, D., Twigg, D., Brown, D., Dewar, J. Evaluation of a remote symptom assessment and management (SAM) system for people receiving adjuvant chemotherapy for breast or colorectal cancer: Mixed methods study. JMIR Cancer. 2020. 6:https://cancer.jmir.org/2020/2/e22825/PDF https://ovidsp.ovid.com/ovidweb.cgi?T=JS&CSC=Y&NEWS=N&PAGE=fulltext&D=emexb&AN=2010408766 | Other diagnosis (not depression/MDD) |  |
| 75 , Watkins, E. R., Newbold, A. Factorial designs help to understand how psychological therapy works. Frontiers in Psychiatry. 2020. 11 (no pagination):http://www.frontiersin.org/Psychiatry https://ovidsp.ovid.com/ovidweb.cgi?T=JS&CSC=Y&NEWS=N&PAGE=fulltext&D=emexb&AN=631873729 | Include this article |  |
|  |  | Inappropriate outcomes |
| 77 , Braun, L., Titzler, I., Terhorst, Y., Freund, J., Thielecke, J., Ebert, D. D., Baumeister, H. Effectiveness of guided internet-based interventions in the indicated prevention of depression in green professions (PROD-A): Results of a pragmatic randomized controlled trial. Journal of Affective Disorders. 2021. 278:658-671 , http://www.elsevier.com/locate/jad https://ovidsp.ovid.com/ovidweb.cgi?T=JS&CSC=Y&NEWS=N&PAGE=fulltext&D=emexb&AN=2008360510 | Include this article |  |
| 77 , Braun, L., Titzler, I., Terhorst, Y., Freund, J., Thielecke, J., Ebert, D. D., Baumeister, H. Effectiveness of guided internet-based interventions in the indicated prevention of depression in green professions (PROD-A): Results of a pragmatic randomized controlled trial. Journal of Affective Disorders. 2021. 278:658-671 , http://www.elsevier.com/locate/jad https://ovidsp.ovid.com/ovidweb.cgi?T=JS&CSC=Y&NEWS=N&PAGE=fulltext&D=emexb&AN=2008360510 |  | Other diagnosis (not depression/MDD) |
| 78 , Watanabe, N., Horikoshi, M., Yamada, M., Shimodera, S., Akechi, T., Miki, K., Inagaki, M., Yonemoto, N., Imai, H., Tajika, A., Ogawa, Y., Takeshima, N., Hayasaka, Y., Furukawa, T. A. Adding smartphone-based cognitive-behavior therapy to pharmacotherapy for major depression (FLATT project): Study protocol for a randomized controlled trial. Trials. 2015. 07:http://www.trialsjournal.com/home/ https://ovidsp.ovid.com/ovidweb.cgi?T=JS&CSC=Y&NEWS=N&PAGE=fulltext&D=emexb&AN=605189403 | Include this article |  |
|  |  | Inappropriate outcomes |
| 79 , Gladstone, T. G., Marko-Holguin, M., Rothberg, P., Nidetz, J., Diehl, A., DeFrino, D. T., Harris, M., Ching, E., Eder, M., Canel, J., Bell, C., Beardslee, W. R., Brown, C. H., Griffiths, K., Van Voorhees, B. W. An internet-based adolescent depression preventive intervention: Study protocol for a randomized control trial. Trials. 2015. 01:http://www.trialsjournal.com/home/ https://ovidsp.ovid.com/ovidweb.cgi?T=JS&CSC=Y&NEWS=N&PAGE=fulltext&D=emexb&AN=604295352 | Inappropriate study design |  |
| 80 , Renfrew, M. E., Morton, D. P., Morton, J. K., Hinze, J. S., Przybylko, G., Craig, B. A. The influence of three modes of human support on attrition and adherence to a web- And mobile app-based mental health promotion intervention in a nonclinical cohort: Randomized comparative study. Journal of Medical Internet Research. 2020. 22:http://www.jmir.org/2020/9/e19945/ https://ovidsp.ovid.com/ovidweb.cgi?T=JS&CSC=Y&NEWS=N&PAGE=fulltext&D=emexa&AN=2008401805 | Other diagnosis (not depression/MDD) |  |
| 81 , Christ, C., Schouten, M. J. E., Blankers, M., van Schaik, D. J. F., Beekman, A. T. F., Wisman, M. A., Stikkelbroek, Y. A. J., Dekker, J. J. M. Internet and computer-based cognitive behavioral therapy for anxiety and depression in adolescents and young adults: Systematic review and meta-analysis. Journal of Medical Internet Research. 2020. 22:https://www.jmir.org/2020/9/e17831 https://ovidsp.ovid.com/ovidweb.cgi?T=JS&CSC=Y&NEWS=N&PAGE=fulltext&D=emexa&AN=2008344520 | Inappropriate study design |  |
| 82 , Lally, R. M., Kupzyk, K., Gallo, S., Berry, D. Use of an unguided, web-based distress self-management program after breast cancer diagnosis: Sub-analysis of caringguidance pilot study. Journal of Medical Internet Research. 2020. 22:https://www.jmir.org/2020/7/e19734 https://ovidsp.ovid.com/ovidweb.cgi?T=JS&CSC=Y&NEWS=N&PAGE=fulltext&D=emexa&AN=2007406787 | Other diagnosis (not depression/MDD) |  |
| 83 , Dulli, L., Ridgeway, K., Packer, C., Murray, K. R., Mumuni, T., Plourde, K. F., Chen, M., Olumide, A., Ojengbede, O., McCarraher, D. R. A Social Media-Based Support Group for Youth Living with HIV in Nigeria (SMART Connections): Randomized Controlled Trial. Journal of Medical Internet Research. 2020. 22:https://www.jmir.org/2020/6/e18343 https://ovidsp.ovid.com/ovidweb.cgi?T=JS&CSC=Y&NEWS=N&PAGE=fulltext&D=emexa&AN=2006974455 | Other diagnosis (not depression/MDD) |  |
| 84 , Guzman-Parra, J., Barnestein-Fonseca, P., Guerrero-Pertinez, G., Anderberg, P., Jimenez-Fernandez, L., Valero-Moreno, E., Goodman-Casanova, J. M., Cuesta-Vargas, A., Garolera, M., Quintana, M., Garcia-Betances, R. I., Lemmens, E., Berglund, J. S., Mayoral-Cleries, F. Attitudes and use of information and communication technologies in older adults with mild cognitive impairment or early stages of dementia and their caregivers: Cross-sectional study. Journal of Medical Internet Research. 2020. 22:https://www.jmir.org/2020/6/e17253 https://ovidsp.ovid.com/ovidweb.cgi?T=JS&CSC=Y&NEWS=N&PAGE=fulltext&D=emexa&AN=2006974441 | Other diagnosis (not depression/MDD) |  |
| 86 , Igelstrom, H., Hauffman, A., Alfonsson, S., Sjostrom, J., Cajander, A., Johansson, B. User experiences of an internet-based stepped-care intervention for individuals with cancer and concurrent symptoms of anxiety or depression (the U-CARE AdultCan Trial): Qualitative study. Journal of Medical Internet Research. 2020. 22:http://www.jmir.org/2020/5/e16604/ https://ovidsp.ovid.com/ovidweb.cgi?T=JS&CSC=Y&NEWS=N&PAGE=fulltext&D=emexa&AN=2006974398 | Include this article |  |
| 86 , Igelstrom, H., Hauffman, A., Alfonsson, S., Sjostrom, J., Cajander, A., Johansson, B. User experiences of an internet-based stepped-care intervention for individuals with cancer and concurrent symptoms of anxiety or depression (the U-CARE AdultCan Trial): Qualitative study. Journal of Medical Internet Research. 2020. 22:http://www.jmir.org/2020/5/e16604/ https://ovidsp.ovid.com/ovidweb.cgi?T=JS&CSC=Y&NEWS=N&PAGE=fulltext&D=emexa&AN=2006974398 |  | Other diagnosis (not depression/MDD) |
| 88 , M. Ajd N.R,Brostrom, A., Ulander, M., Lin, C. Y., Griffiths, M. D., Imani, V., Ahorsu, D. K., Ohayon, M. M., Pakpour, A. H. Efficacy of a theory-based cognitive behavioral technique app-based intervention for patients with insomnia: Randomized controlled trial. Journal of Medical Internet Research. 2020. 22:https://www.jmir.org/2020/4/e15841/PDF https://ovidsp.ovid.com/ovidweb.cgi?T=JS&CSC=Y&NEWS=N&PAGE=fulltext&D=emexa&AN=2005831935 | Other diagnosis (not depression/MDD) |  |
| 89 , Haque, M. S., Kangas, M., Jamsa, T. A persuasive mhealth behavioral change intervention for promoting physical activity in the workplace: Feasibility randomized controlled trial. Journal of Medical Internet Research. 2020. 4:https://formative.jmir.org/2020/5/e15083/ https://ovidsp.ovid.com/ovidweb.cgi?T=JS&CSC=Y&NEWS=N&PAGE=fulltext&D=emexa&AN=2005811030 | Other diagnosis (not depression/MDD) |  |
| 90 , NeCamp, T., Sen, S., Frank, E., Walton, M. A., Ionides, E. L., Fang, Y., Tewari, A., Wu, Z. Assessing real-time moderation for developing adaptive mobile health interventions for medical interns: Micro-randomized trial. Journal of Medical Internet Research. 2020. 22:https://www.jmir.org/2020/3/e15033/ https://ovidsp.ovid.com/ovidweb.cgi?T=JS&CSC=Y&NEWS=N&PAGE=fulltext&D=emexa&AN=2005789016 | Other diagnosis (not depression/MDD) |  |
| 91 , Hauffman, A., Alfonsson, S., Igelstrom, H., Johansson, B. Experiences of internet-based stepped care in individuals with cancer and concurrent symptoms of anxiety and depression: Qualitative exploration conducted alongside the U-Care Adultcan randomized controlled trial. Journal of Medical Internet Research. 2020. 22:https://www.jmir.org/2020/3/e16547/ https://ovidsp.ovid.com/ovidweb.cgi?T=JS&CSC=Y&NEWS=N&PAGE=fulltext&D=emexa&AN=2005789015 | Include this article |  |
|  |  | Other diagnosis (not depression/MDD) |
| 92 , Heller, H. M., Hoogendoorn, A. W., Honig, A., Broekman, B. F. P., van Straten, A. The effectiveness of a guided Internet-based tool for the treatment of depression and anxiety in pregnancy (Mamakits online): Randomized controlled trial. Journal of Medical Internet Research. 2020. 22:https://www.jmir.org/2020/3/e15172/ https://ovidsp.ovid.com/ovidweb.cgi?T=JS&CSC=Y&NEWS=N&PAGE=fulltext&D=emexa&AN=2005789011 | Include this article |  |
|  |  | Other diagnosis (not depression/MDD) |
| 93 , Powell, J., Williams, V., Atherton, H., Bennett, K., Yang, Y., Davoudianfar, M., Hellsing, A., Martin, A., Mollison, J., Shanyinde, M., Yu, L. M., Griffiths, K. M. Effectiveness and cost-effectiveness of a self-guided internet intervention for social anxiety symptoms in a general population sample: Randomized controlled trial. Journal of Medical Internet Research. 2020. 22:https://www.jmir.org/2020/1/e16804/pdf https://ovidsp.ovid.com/ovidweb.cgi?T=JS&CSC=Y&NEWS=N&PAGE=fulltext&D=emexa&AN=2005773458 | Other diagnosis (not depression/MDD) |  |
| 94 , Guo, Y., Hong, Y. A., Cai, W., Li, L., Hao, Y., Qiao, J., Xu, Z., Zhang, H., Zeng, C., Liu, C., Li, Y., Zhu, M., Zeng, Y., Penedo, F. J. Effect of a WeChat-Based intervention (Run4Love) on depressive symptoms among people living with HIV in China: A randomized controlled trial. Journal of Medical Internet Research. 2020. 22:https://www.jmir.org/2020/2/e16715/pdf https://ovidsp.ovid.com/ovidweb.cgi?T=JS&CSC=Y&NEWS=N&PAGE=fulltext&D=emexa&AN=2005773445 | Include this article |  |
|  |  | Other diagnosis (not depression/MDD) |
| 95 , Renfrew, M. E., Morton, D. P., Morton, J. K., Hinze, J. S., Beamish, P. J., Przybylko, G., Craig, B. A. A web- And mobile app-based mental health promotion intervention comparing email, short message service, and videoconferencing support for a healthy cohort: Randomized comparative study. Journal of Medical Internet Research. 2020. 22:https://www.jmir.org/2020/1/e15592/PDF https://ovidsp.ovid.com/ovidweb.cgi?T=JS&CSC=Y&NEWS=N&PAGE=fulltext&D=emexa&AN=2004555941 | Other diagnosis (not depression/MDD) |  |
| 96 , Nobakht, Z., Rassafiani, M., Hosseini, S. A., Hosseinzadeh, S. A web-based daily care training to improve the quality of life of mothers of children with cerebral palsy: A randomized controlled trial. Research in Developmental Disabilities. 2020. 105 (no pagination):http://www.elsevier.com/locate/redevdis https://ovidsp.ovid.com/ovidweb.cgi?T=JS&CSC=Y&NEWS=N&PAGE=fulltext&D=emexa&AN=2007006436 | Other diagnosis (not depression/MDD) |  |
| 97 , Srivastava, P., Mehta, M., Sagar, R., Ambekar, A. Smartteen- a computer assisted cognitive behavior therapy for Indian adolescents with depression- a pilot study. Asian Journal of Psychiatry. 2020. 50 (no pagination):http://www.elsevier.com/wps/find/journaldescription.cws_home/717029/description#description https://ovidsp.ovid.com/ovidweb.cgi?T=JS&CSC=Y&NEWS=N&PAGE=fulltext&D=emexa&AN=2005075842 | Inappropriate study design |  |
| 98 , Torous, J., Lipschitz, J., Ng, M., Firth, J. Dropout rates in clinical trials of smartphone apps for depressive symptoms: A systematic review and meta-analysis. Journal of Affective Disorders. 2020. 263:413-419 , http://www.elsevier.com/locate/jad https://ovidsp.ovid.com/ovidweb.cgi?T=JS&CSC=Y&NEWS=N&PAGE=fulltext&D=emexa&AN=2004208482 | Inappropriate study design |  |
| 99 , Hui, V. K. Y., Wong, C. Y. F., Ma, E. K. Y., Ho, F. Y. Y., Chan, C. S. Treating depression with a smartphone-delivered self-help cognitive behavioral therapy for insomnia: Study protocol for a parallel group randomized controlled trial. Trials. 2020. 21:http://www.trialsjournal.com/home/ https://ovidsp.ovid.com/ovidweb.cgi?T=JS&CSC=Y&NEWS=N&PAGE=fulltext&D=emexa&AN=633044814 | Include this article |  |
| 99 , Hui, V. K. Y., Wong, C. Y. F., Ma, E. K. Y., Ho, F. Y. Y., Chan, C. S. Treating depression with a smartphone-delivered self-help cognitive behavioral therapy for insomnia: Study protocol for a parallel group randomized controlled trial. Trials. 2020. 21:http://www.trialsjournal.com/home/ https://ovidsp.ovid.com/ovidweb.cgi?T=JS&CSC=Y&NEWS=N&PAGE=fulltext&D=emexa&AN=633044814 |  | Inappropriate outcomes |
| 100 , Bodschwinna, D., Lorenz, I., Bauereiss, N., Gundel, H., Baumeister, H., Hoenig, K. PartnerCARE-a psycho-oncological online intervention for partners of patients with cancer: Study protocol for a randomised controlled feasibility trial. BMJ Open. 2020. 10:http://bmjopen.bmj.com/content/early/by/section https://ovidsp.ovid.com/ovidweb.cgi?T=JS&CSC=Y&NEWS=N&PAGE=fulltext&D=emexa&AN=633027403 | Other diagnosis (not depression/MDD) |  |
| 101 , Bossert, M., Westermann, C., Schilling, T. M., Weisbrod, M., Roesch-Ely, D., Aschenbrenner, S. Computer-Assisted Cognitive Remediation in Schizophrenia: Efficacy of an Individualized vs. Generic Exercise Plan. Frontiers in Psychiatry. 2020. 11 (no pagination):http://www.frontiersin.org/Psychiatry https://ovidsp.ovid.com/ovidweb.cgi?T=JS&CSC=Y&NEWS=N&PAGE=fulltext&D=emexa&AN=632990367 | Other diagnosis (not depression/MDD) |  |
| 102 , Sander, L. B., Paganini, S., Terhorst, Y., Schlicker, S., Lin, J., Spanhel, K., Buntrock, C., Ebert, D. D., Baumeister, H. Effectiveness of a Guided Web-Based Self-help Intervention to Prevent Depression in Patients with Persistent Back Pain: The PROD-BP Randomized Clinical Trial. JAMA Psychiatry. 2020. 77:1001-1011 , http://archpsyc.jamanetwork.com/issues.aspx https://ovidsp.ovid.com/ovidweb.cgi?T=JS&CSC=Y&NEWS=N&PAGE=fulltext&D=emexa&AN=631949878 | Other diagnosis (not depression/MDD) |  |
| 103 , Chapman, S., Sibelli, A., St-Clair Jones, A., Forbes, A., Chater, A., Horne, R. Personalised Adherence Support for Maintenance Treatment of Inflammatory Bowel Disease: A Tailored Digital Intervention to Change Adherence-related Beliefs and Barriers. Journal of Crohn's and Colitis. 2020. 14:1394-1404 , https://academic.oup.com/ecco-jcc/issue https://ovidsp.ovid.com/ovidweb.cgi?T=JS&CSC=Y&NEWS=N&PAGE=fulltext&D=emexa&AN=2010037768 | Other diagnosis (not depression/MDD) |  |
| 104 , Kantrowitz-Gordon, I., McCurry, S. M., Landis, C. A., Lee, R., Wi, D. Online prenatal trial in mindfulness sleep management (OPTIMISM): Protocol for a pilot randomized controlled trial. Pilot and Feasibility Studies. 2020. 6:https://pilotfeasibilitystudies.biomedcentral.com/ https://ovidsp.ovid.com/ovidweb.cgi?T=JS&CSC=Y&NEWS=N&PAGE=fulltext&D=emexa&AN=632864857 | Other diagnosis (not depression/MDD) |  |
| 105 , Terhorst, Y., Braun, L., Titzler, I., Buntrock, C., Freund, J., Thielecke, J., Ebert, D., Baumeister, H. Clinical and cost-effectiveness of a guided internet-based Acceptance and Commitment Therapy to improve chronic pain-related disability in green professions (PACT-A): Study protocol of a pragmatic randomised controlled trial. BMJ Open. 2020. 10:http://bmjopen.bmj.com/content/early/by/section https://ovidsp.ovid.com/ovidweb.cgi?T=JS&CSC=Y&NEWS=N&PAGE=fulltext&D=emexa&AN=632758089 | Other diagnosis (not depression/MDD) |  |
| 106 , Aguilera, A., Figueroa, C. A., Hernandez-Ramos, R., Sarkar, U., Cemballi, A., Gomez-Pathak, L., Miramontes, J., Yom-Tov, E., Chakraborty, B., Yan, X., Xu, J., Modiri, A., Aggarwal, J., Jay Williams, J., Lyles, C. R. MHealth app using machine learning to increase physical activity in diabetes and depression: Clinical trial protocol for the DIAMANTE Study. BMJ Open. 2020. 10:http://bmjopen.bmj.com/content/early/by/section https://ovidsp.ovid.com/ovidweb.cgi?T=JS&CSC=Y&NEWS=N&PAGE=fulltext&D=emexa&AN=632632251 | Include this article |  |
|  |  | Other diagnosis (not depression/MDD) |
| 107 , Bountress, K. E., Gilmore, A. K., Metzger, I. W., Aggen, S. H., Tomko, R. L., Danielson, C. K., Williamson, V., Vladmirov, V., Ruggiero, K., Amstadter, A. B. Impact of disaster exposure severity: Cascading effects across parental distress, adolescent PTSD symptoms, as well as parent-child conflict and communication. Social Science and Medicine. 2020. 264 (no pagination):http://www.elsevier.com/locate/socscimed https://ovidsp.ovid.com/ovidweb.cgi?T=JS&CSC=Y&NEWS=N&PAGE=fulltext&D=emexa&AN=2007573931 | Other diagnosis (not depression/MDD) |  |
| 108 , Bennion, K. A., Tate, D., Munoz-Christian, K., Phelan, S. Impact of an Internet-Based Lifestyle Intervention on Behavioral and Psychosocial Factors During Postpartum Weight Loss. Obesity. 2020. 28:1860-1867 , http://onlinelibrary.wiley.com/journal/10.1002/(ISSN)1930-739X https://ovidsp.ovid.com/ovidweb.cgi?T=JS&CSC=Y&NEWS=N&PAGE=fulltext&D=emexa&AN=2006069404 | Other diagnosis (not depression/MDD) |  |
| 109 , Collins, D. A. J., Harvey, S. B., Lavender, I., Glozier, N., Christensen, H., Deady, M. A pilot evaluation of a smartphone application for workplace depression. International Journal of Environmental Research and Public Health. 2020. 17:1-14 , https://www.mdpi.com/1660-4601/17/18/6753/pdf https://ovidsp.ovid.com/ovidweb.cgi?T=JS&CSC=Y&NEWS=N&PAGE=fulltext&D=emexa&AN=2005075133 | Other diagnosis (not depression/MDD) |  |
| 110 , Wadon, M. E., Winter, M., Peall, K. J. Internet-based cognitive behavioural therapy programme as an intervention for people diagnosed with adult-onset, focal, isolated, idiopathic cervical dystonia: A feasibility study protocol. Pilot and Feasibility Studies. 2020. 6:https://pilotfeasibilitystudies.biomedcentral.com/ https://ovidsp.ovid.com/ovidweb.cgi?T=JS&CSC=Y&NEWS=N&PAGE=fulltext&D=emexa&AN=632334887 | Other diagnosis (not depression/MDD) |  |
| 111 , Lin, J., Wurst, R., Paganini, S., Hohberg, V., Kinkel, S., Gohner, W., Ramsenthaler, C., Fuchs, R. A group- And smartphone-based psychological intervention to increase and maintain physical activity in patients with musculoskeletal conditions: Study protocol for a randomized controlled trial ("MoVo-App"). Trials. 2020. 21:http://www.trialsjournal.com/home/ https://ovidsp.ovid.com/ovidweb.cgi?T=JS&CSC=Y&NEWS=N&PAGE=fulltext&D=emexa&AN=632000477 | Other diagnosis (not depression/MDD) |  |
| 112 , Humer, E., Kocsis-Bogar, K., Berger, T., Schroder, J., Spath, C., Meyer, B., Moritz, S., Lutz, W., Probst, T., Klein, J. P. A comparison of the three year course between chronic depression and depression with multiple vs. few prior episodes. Psychiatry Research. 2020. 291 (no pagination):http://www.elsevier.com/locate/psychres https://ovidsp.ovid.com/ovidweb.cgi?T=JS&CSC=Y&NEWS=N&PAGE=fulltext&D=emexa&AN=2006855461 | Include this article |  |
|  |  | Inappropriate outcomes |
| 114 , Anastasiadou, D., Folkvord, F., Brugnera, A., Canas Vinader, L., SerranoTroncoso, E., Carretero Jardi, C., Linares Bertolin, R., Munoz Rodriguez, R., Martinez Nunez, B., Graell Berna, M., Torralbas-Ortega, J., Torrent-Sola, L., Punti-Vidal, J., Carrera Ferrer, M., Munoz Domenjo, A., Diaz Marsa, M., Gunnard, K., Cusido, J., Arcal Cunillera, J., Lupianez-Villanueva, F. An mHealth intervention for the treatment of patients with an eating disorder: A multicenter randomized controlled trial. International Journal of Eating Disorders. 2020. 53:1120-1131 , http://onlinelibrary.wiley.com/journal/10.1002/(ISSN)1098-108X https://ovidsp.ovid.com/ovidweb.cgi?T=JS&CSC=Y&NEWS=N&PAGE=fulltext&D=emexa&AN=2004856949 | Other diagnosis (not depression/MDD) |  |
| 115 , Monteiro, F., Pereira, M., Canavarro, M. C., Fonseca, A. Be a mom's efficacy in enhancing positive mental health among postpartum women presenting low risk for postpartum depression: Results from a pilot randomized trial. International Journal of Environmental Research and Public Health. 2020. 17:1-19 , https://www.mdpi.com/1660-4601/17/13/4679/pdf https://ovidsp.ovid.com/ovidweb.cgi?T=JS&CSC=Y&NEWS=N&PAGE=fulltext&D=emexa&AN=2004639580 | Other diagnosis (not depression/MDD) |  |
| 116 , Fonseca, A., Alves, S., Monteiro, F., Gorayeb, R., Canavarro, M. C. Be a Mom, a Web-Based Intervention to Prevent Postpartum Depression: Results From a Pilot Randomized Controlled Trial. Behavior Therapy. 2020. 51:616-633 , http://www.journals.elsevier.com/behavior-therapy/ https://ovidsp.ovid.com/ovidweb.cgi?T=JS&CSC=Y&NEWS=N&PAGE=fulltext&D=emexa&AN=2004599572 | Other diagnosis (not depression/MDD) |  |
| 117 , Truttmann, S., Philipp, J., Zeiler, M., Franta, C., Wittek, T., Merl, E., Schofbeck, G., Koubek, D., Laczkovics, C., Imgart, H., Zanko, A., Auer-welsbach, E., Treasure, J., Karwautz, A. F. K., Wagner, G. Long-term efficacy of the workshop vs. Online succeat (supporting carers of children and adolescents with eating disorders) intervention for parents: A quasi-randomised feasibility trial. Journal of Clinical Medicine. 2020. 9:1-18 , https://www.mdpi.com/2077-0383/9/6/1912/pdf https://ovidsp.ovid.com/ovidweb.cgi?T=JS&CSC=Y&NEWS=N&PAGE=fulltext&D=emexa&AN=2004571906 | Other diagnosis (not depression/MDD) |  |
| 118 , Bjureberg, J., Enander, J., Andersson, E., Ivanov, V. Z., Ruck, C., Fernandez de la Cruz, L. Sudden Gains in Internet-Based Cognitive Behavior Therapy for Body Dysmorphic Disorder. Behavior Therapy. 2020. 51:753-763 , http://www.journals.elsevier.com/behavior-therapy/ https://ovidsp.ovid.com/ovidweb.cgi?T=JS&CSC=Y&NEWS=N&PAGE=fulltext&D=emexa&AN=2004520425 | Other diagnosis (not depression/MDD) |  |
| 120 , Blessing, E., Virani, S., Rotrosen, J. Clinical trials for opioid use disorder. Handbook of Experimental Pharmacology. 2020. 258:167-202 , http://www.springer.com/series/164 https://ovidsp.ovid.com/ovidweb.cgi?T=JS&CSC=Y&NEWS=N&PAGE=fulltext&D=emexa&AN=631988793 | Other diagnosis (not depression/MDD) |  |
| 121 , Kendrick, T., Geraghty, A. W. A., Bowers, H., Stuart, B., Leydon, G., May, C., Yao, G., O'Brien, W., Glowacka, M., Holley, S., Williams, S., Zhu, S., Dewar-Haggart, R., Palmer, B., Bell, M., Collinson, S., Fry, I., Lewis, G., Griffiths, G., Gilbody, S., Moncrieff, J., Moore, M., Macleod, U., Little, P., Dowrick, C. REDUCE (Reviewing long-term antidepressant use by careful monitoring in everyday practice) internet and telephone support to people coming off long-term antidepressants: Protocol for a randomised controlled trial. Trials. 2020. 21:http://www.trialsjournal.com/home/ https://ovidsp.ovid.com/ovidweb.cgi?T=JS&CSC=Y&NEWS=N&PAGE=fulltext&D=emexa&AN=631835062 | Include this article |  |
| 121 , Kendrick, T., Geraghty, A. W. A., Bowers, H., Stuart, B., Leydon, G., May, C., Yao, G., O'Brien, W., Glowacka, M., Holley, S., Williams, S., Zhu, S., Dewar-Haggart, R., Palmer, B., Bell, M., Collinson, S., Fry, I., Lewis, G., Griffiths, G., Gilbody, S., Moncrieff, J., Moore, M., Macleod, U., Little, P., Dowrick, C. REDUCE (Reviewing long-term antidepressant use by careful monitoring in everyday practice) internet and telephone support to people coming off long-term antidepressants: Protocol for a randomised controlled trial. Trials. 2020. 21:http://www.trialsjournal.com/home/ https://ovidsp.ovid.com/ovidweb.cgi?T=JS&CSC=Y&NEWS=N&PAGE=fulltext&D=emexa&AN=631835062 |  | Inappropriate intervention |
| 122 , Nishi, D., Imamura, K., Watanabe, K., Obikane, E., Sasaki, N., Yasuma, N., Sekiya, Y., Matsuyama, Y., Kawakami, N. Internet-based cognitive-behavioural therapy for prevention of depression during pregnancy and in the post partum (iPDP): A protocol for a large-scale randomised controlled trial. BMJ Open. 2020. 10:http://bmjopen.bmj.com/content/early/by/section https://ovidsp.ovid.com/ovidweb.cgi?T=JS&CSC=Y&NEWS=N&PAGE=fulltext&D=emexa&AN=631797833 | Other diagnosis (not depression/MDD) |  |
| 123 , Van Der Zweerde, T., Lancee, J., Slottje, P., Bosmans, J. E., Van Someren, E. J. W., Van Straten, A. Nurse-guided internet-delivered cognitive behavioral therapy for insomnia in general practice: Results from a pragmatic randomized clinical trial. Psychotherapy and Psychosomatics. 2020. 89:174-184 , http://www.karger.com/journals/pps/pps_jh.htm https://ovidsp.ovid.com/ovidweb.cgi?T=JS&CSC=Y&NEWS=N&PAGE=fulltext&D=emexa&AN=631175864 | Other diagnosis (not depression/MDD) |  |
| 124 , Anton, M. T., Ridings, L. E., Hanson, R., Davidson, T., Saunders, B., Price, M., Kmett Danielson, C., Chu, B., Dismuke, C. E., Adams, Z. W., Ruggiero, K. J. Hybrid type 1 randomized controlled trial of a tablet-based application to improve quality of care in child mental health treatment. Contemporary Clinical Trials. 2020. 94 (no pagination):http://www.elsevier.com/wps/find/journaldescription.cws_home/704636/description#description https://ovidsp.ovid.com/ovidweb.cgi?T=JS&CSC=Y&NEWS=N&PAGE=fulltext&D=emexa&AN=2006082658 | Inappropriate study design |  |
| 125 , Gaigg, S. B., Flaxman, P. E., McLaven, G., Shah, R., Bowler, D. M., Meyer, B., Roestorf, A., Haenschel, C., Rodgers, J., South, M. Self-guided mindfulness and cognitive behavioural practices reduce anxiety in autistic adults: A pilot 8-month waitlist-controlled trial of widely available online tools. Autism. 2020. 24:867-883 , http://www.sageltd.co.uk/journals/details/j0192.html https://ovidsp.ovid.com/ovidweb.cgi?T=JS&CSC=Y&NEWS=N&PAGE=fulltext&D=emexa&AN=2004644101 | Other diagnosis (not depression/MDD) |  |
| 126 , Patti, F., Gines, M. L. M., Norenberg, C., Caron, F. D. Betaeval global: Prospective, multinational, observational cohort study of patients using betaconnect. Patient Preference and Adherence. 2020. 14:771-779 , https://www.dovepress.com/getfile.php?fileID=57689 https://ovidsp.ovid.com/ovidweb.cgi?T=JS&CSC=Y&NEWS=N&PAGE=fulltext&D=emexa&AN=2004275483 | Inappropriate study design |  |
| 127 , Sunjaya, A. P., Chris, A., Novianti, D. Efficacy, patient-doctor relationship, costs and benefits of utilizing telepsychiatry for the management of posttraumatic stress disorder (PTSD): A systematic review. Trends in Psychiatry and Psychotherapy. 2020. 42:102-110 , http://www.scielo.br/pdf/trends/v42n1/2238-0019-trends-42-01-0102.pdf https://ovidsp.ovid.com/ovidweb.cgi?T=JS&CSC=Y&NEWS=N&PAGE=fulltext&D=emexa&AN=2004242032 | Inappropriate study design |  |
| 128 , Stefanopoulou, E., Hogarth, H., Taylor, M., Russell-Haines, K., Lewis, D., Larkin, J. Are digital interventions effective in reducing suicidal ideation and self-harm? A systematic review. Journal of Mental Health. 2020. 29:207-216 , http://www.tandfonline.com/loi/ijmh20#.V6QcJU1f1Fo https://ovidsp.ovid.com/ovidweb.cgi?T=JS&CSC=Y&NEWS=N&PAGE=fulltext&D=emexa&AN=2004210062 | Inappropriate study design |  |
| 129 , Kraepelien, M., Schibbye, R., Mansson, K., Sundstrom, C., Riggare, S., Andersson, G., Lindefors, N., Svenningsson, P., Kaldo, V. Individually Tailored Internet-Based Cognitive-Behavioral Therapy for Daily Functioning in Patients with Parkinson's Disease: A Randomized Controlled Trial. Journal of Parkinson's Disease. 2020. 10:653-664 , http://www.iospress.nl/journal/journal-of-parkinsons-disease/ https://ovidsp.ovid.com/ovidweb.cgi?T=JS&CSC=Y&NEWS=N&PAGE=fulltext&D=emexa&AN=631504731 | Other diagnosis (not depression/MDD) |  |
| 130 , Schultchen, D., Kuchler, A. M., Schillings, C., Weineck, F., Karabatsiakis, A., Ebert, D. D., Baumeister, H., Pollatos, O. Effectiveness of a guided online mindfulness-focused intervention in a student population: Study protocol for a randomised control trial. BMJ Open. 2020. 10:http://bmjopen.bmj.com/content/early/by/section https://ovidsp.ovid.com/ovidweb.cgi?T=JS&CSC=Y&NEWS=N&PAGE=fulltext&D=emexa&AN=631351717 | Other diagnosis (not depression/MDD) |  |
| 131 , Bell, L. V., Cornish, P., Flusk, D., Garland, S. N., Rash, J. A. The INternet ThERapy for deprESsion Trial (INTEREST): Protocol for a patient-preference, randomised controlled feasibility trial comparing iACT, iCBT and attention control among individuals with comorbid chronic pain and depression. BMJ Open. 2020. 10:http://bmjopen.bmj.com/content/early/by/section https://ovidsp.ovid.com/ovidweb.cgi?T=JS&CSC=Y&NEWS=N&PAGE=fulltext&D=emexa&AN=631089441 | Include this article |  |
| 131 , Bell, L. V., Cornish, P., Flusk, D., Garland, S. N., Rash, J. A. The INternet ThERapy for deprESsion Trial (INTEREST): Protocol for a patient-preference, randomised controlled feasibility trial comparing iACT, iCBT and attention control among individuals with comorbid chronic pain and depression. BMJ Open. 2020. 10:http://bmjopen.bmj.com/content/early/by/section https://ovidsp.ovid.com/ovidweb.cgi?T=JS&CSC=Y&NEWS=N&PAGE=fulltext&D=emexa&AN=631089441 |  | Other diagnosis (not depression/MDD) |
| 132 , Bernd, M., Schick, M., Rosner, S., Germeyer, A., Strowitzki, T., Moessner, M., Bauer, S., Ditzen, B., Wischmann, T. Predictors for the Early Termination of a Psychological Intervention during Treatment with Assisted Reproductive Technologies. Geburtshilfe und Frauenheilkunde. 2020. 80:190-199 , http://www.thieme-connect.com/ejournals/toc/gebfra https://ovidsp.ovid.com/ovidweb.cgi?T=JS&CSC=Y&NEWS=N&PAGE=fulltext&D=emexa&AN=631021313 | Other diagnosis (not depression/MDD) |  |
| 133 , Greer, J. A., Jacobs, J. M., Pensak, N., Nisotel, L. E., Fishbein, J. N., MacDonald, J. J., Ream, M. E., Walsh, E. A., Buzaglo, J., Muzikansky, A., Lennes, I. T., Safren, S. A., Pirl, W. F., Temel, J. S. Randomized trial of a smartphone mobile app to improve symptoms and adherence to oral therapy for cancer. JNCCN Journal of the National Comprehensive Cancer Network. 2020. 18:133-141 , https://jnccn.org/view/journals/jnccn/18/2/article-p116.xml https://ovidsp.ovid.com/ovidweb.cgi?T=JS&CSC=Y&NEWS=N&PAGE=fulltext&D=emexa&AN=2005383887 | Other diagnosis (not depression/MDD) |  |
| 134 , Holtdirk, F., Mehnert, A., Weiss, M., Meyer, B., Watzl, C. Protocol for the Optimune trial: A randomized controlled trial evaluating a novel Internet intervention for breast cancer survivors. Trials. 2020. 21:http://www.trialsjournal.com/home/ https://ovidsp.ovid.com/ovidweb.cgi?T=JS&CSC=Y&NEWS=N&PAGE=fulltext&D=emexa&AN=630805882 | Other diagnosis (not depression/MDD) |  |
| 135 , Armitage, L. C., Kassavou, A., Sutton, S. Do mobile device apps designed to support medication adherence demonstrate efficacy? A systematic review of randomised controlled trials, with meta-analysis. BMJ Open. 2020. 10:http://bmjopen.bmj.com/content/early/by/section https://ovidsp.ovid.com/ovidweb.cgi?T=JS&CSC=Y&NEWS=N&PAGE=fulltext&D=emexa&AN=630771295 | Inappropriate study design |  |
| 136 , Werner-Seidler, A., Huckvale, K., Larsen, M. E., Calear, A. L., Maston, K., Johnston, L., Torok, M., O'Dea, B., Batterham, P. J., Schweizer, S., Skinner, S. R., Steinbeck, K., Ratcliffe, J., Oei, J. L., Patton, G., Wong, I., Beames, J., Wong, Q. J. J., Lingam, R., Boydell, K., Salmon, A. M., Cockayne, N., Mackinnon, A., Christensen, H. A trial protocol for the effectiveness of digital interventions for preventing depression in adolescents: The Future Proofing Study. Trials. 2020. 21:http://www.trialsjournal.com/home/ https://ovidsp.ovid.com/ovidweb.cgi?T=JS&CSC=Y&NEWS=N&PAGE=fulltext&D=emexa&AN=630438121 | Inappropriate study design |  |
| 137 , Eccles, H., Nannarone, M., Lashewicz, B., Attridge, M., Marchand, A., Aiken, A., Ho, K., Wang, J. Perceived effectiveness and motivations for the use of web-based mental health programs: Qualitative study. Journal of Medical Internet Research. 2020. 22:http://www.jmir.org/2020/7/e16961/ https://ovidsp.ovid.com/ovidweb.cgi?T=JS&CSC=Y&NEWS=N&PAGE=fulltext&D=emexa&AN=2007555318 | Inappropriate study design |  |
| 138 , Provoost, S., Kleiboer, A., Ornelas, J., Bosse, T., Ruwaard, J., Rocha, A., Cuijpers, P., Riper, H. Improving adherence to an online intervention for low mood with a virtual coach: study protocol of a pilot randomized controlled trial. Trials. 2020. 21:http://www.trialsjournal.com/home/ https://ovidsp.ovid.com/ovidweb.cgi?T=JS&CSC=Y&NEWS=N&PAGE=fulltext&D=emexa&AN=2007009132 | Other diagnosis (not depression/MDD) |  |
| 139 , Lai, K. S. P., Watt, C., Ionson, E., Baruss, I., Forchuk, C., Sukhera, J., Burhan, A. M., Vasudev, A. Breath Regulation and yogic Exercise An online Therapy for calm and Happiness (BREATH) for frontline hospital and long-term care home staff managing the COVID-19 pandemic: A structured summary of a study protocol for a feasibility study for a randomised controlled trial. Trials. 2020. 21:648 , https://ovidsp.ovid.com/ovidweb.cgi?T=JS&CSC=Y&NEWS=N&PAGE=fulltext&D=emexa&AN=632364009 | Other diagnosis (not depression/MDD) |  |
| 140 , Li, Y. H., Mu, T. Y., Zhang, L., Zhang, C. L., Wu, D., Chen, J. J., Wang, F. Internet-based intervention for postpartum depression in China ("Mommy go"): Protocol for a randomized controlled trial. Journal of advanced nursing. 2020. 76:2416-2425 , https://ovidsp.ovid.com/ovidweb.cgi?T=JS&CSC=Y&NEWS=N&PAGE=fulltext&D=emexa&AN=631871756 | Other diagnosis (not depression/MDD) |  |
| 141 , Henry, B. L., Quintana, E., Moore, D. J., Garcia, J., Montoya, J. L. Focus groups inform a mobile health intervention to promote adherence to a Mediterranean diet and engagement in physical activity among people living with HIV. BMC public health. 2019. 19:101 , https://ovidsp.ovid.com/ovidweb.cgi?T=JS&CSC=Y&NEWS=N&PAGE=fulltext&D=emexa&AN=626062170 | Other diagnosis (not depression/MDD) |  |
| 142 , Westerhof, G. J., Lamers, S. M. A., Postel, M. G., Bohlmeijer, E. T. Online Therapy for Depressive Symptoms: An Evaluation of Counselor-Led and Peer-Supported Life Review Therapy. The Gerontologist. 2019. 59:135-146 , https://ovidsp.ovid.com/ovidweb.cgi?T=JS&CSC=Y&NEWS=N&PAGE=fulltext&D=emexa&AN=625875996 | Other diagnosis (not depression/MDD) |  |
| 143 , Rice, S., O'Bree, B., Wilson, M., McEnery, C., Lim, M. H., Hamilton, M., Gleeson, J., Bendall, S., D'Alfonso, S., Russon, P., Valentine, L., Cagliarini, D., Howell, S., Miles, C., Pearson, M., Nicholls, L., Garland, N., Mullen, E., McGorry, P. D., Alvarez-Jimenez, M. Leveraging the social network for treatment of social anxiety: Pilot study of a youth-specific digital intervention with a focus on engagement of young men. Internet Interventions. 2020. 20 (no pagination):http://www.journals.elsevier.com/internet-interventions/ https://ovidsp.ovid.com/ovidweb.cgi?T=JS&CSC=Y&NEWS=N&PAGE=fulltext&D=emexa&AN=2005879566 | Other diagnosis (not depression/MDD) |  |
| 144 , Rogala, A., Szczepaniak, M., Michalak, N., Andersson, G. Internet-based self-help intervention aimed at increasing social self-efficacy among internal migrants in Poland: Study protocol for a randomized controlled trial. Internet Interventions. 2020. 21 (no pagination):http://www.journals.elsevier.com/internet-interventions/ https://ovidsp.ovid.com/ovidweb.cgi?T=JS&CSC=Y&NEWS=N&PAGE=fulltext&D=emexa&AN=2005728157 | Other diagnosis (not depression/MDD) |  |
| 145 , Moman, R. N., Dvorkin, J., Pollard, E. M., Wanderman, R., Murad, M. H., Warner, D. O., Hooten, W. M. A Systematic Review and Meta-analysis of Unguided Electronic and Mobile Health Technologies for Chronic Pain - Is It Time to Start Prescribing Electronic Health Applications?. Pain Medicine (United States). 2019. 20:2238-2255 , http://oxfordjournals.org/en/press/announcements-from-oup/pain-medicine.html https://ovidsp.ovid.com/ovidweb.cgi?T=JS&CSC=Y&NEWS=N&PAGE=fulltext&D=emed20&AN=631498171 | Inappropriate study design |  |
| 146 , Al-Shammari, I., Roa, L., Yorlets, R. R., Akerman, C., Dekker, A., Kelley, T., Koech, R., Mutuku, J., Nyarango, R., Nzorubara, D., Spieker, N., Vaidya, M., Meara, J. G., Ljungman, D. Implementation of an international standardized set of outcome indicators in pregnancy and childbirth in Kenya: Utilizing mobile technology to collect patient-reported outcomes. PLoS ONE. 2019. 14:https://journals.plos.org/plosone/article/file?id=10.1371/journal.pone.0222978&type=printable https://ovidsp.ovid.com/ovidweb.cgi?T=JS&CSC=Y&NEWS=N&PAGE=fulltext&D=emed20&AN=2003397131 | Other diagnosis (not depression/MDD) |  |
| 147 , Cox, N. S., Eldridge, B., Rawlings, S., Dreger, J., Corda, J., Hauser, J., Button, B. M., Bishop, J., Nichols, A., Middleton, A., Ward, N., Dwyer, T., Tomlinson, O. W., Denford, S., Barker, A. R., Williams, C. A., Kingsley, M., O'Halloran, P., Holland, A. E. A web-based intervention to promote physical activity in adolescents and young adults with cystic fibrosis: Protocol for a randomized controlled trial. BMC Pulmonary Medicine. 2019. 19:http://www.biomedcentral.com/bmcpulmmed/ https://ovidsp.ovid.com/ovidweb.cgi?T=JS&CSC=Y&NEWS=N&PAGE=fulltext&D=emed20&AN=630288104 | Other diagnosis (not depression/MDD) |  |
| 148 , Murphy, M. J., Newby, J. M., Butow, P., Loughnan, S. A., Joubert, A. E., Kirsten, L., Allison, K., Shaw, J., Shepherd, H. L., Smith, J., Andrews, G. Randomised controlled trial of internet-delivered cognitive behaviour therapy for clinical depression and/or anxiety in cancer survivors (iCanADAPT Early). Psycho-Oncology. 2020. 29:76-85 , http://onlinelibrary.wiley.com/journal/10.1002/(ISSN)1099-1611 https://ovidsp.ovid.com/ovidweb.cgi?T=JS&CSC=Y&NEWS=N&PAGE=fulltext&D=emed20&AN=2003635356 | Include this article |  |
| 148 , Murphy, M. J., Newby, J. M., Butow, P., Loughnan, S. A., Joubert, A. E., Kirsten, L., Allison, K., Shaw, J., Shepherd, H. L., Smith, J., Andrews, G. Randomised controlled trial of internet-delivered cognitive behaviour therapy for clinical depression and/or anxiety in cancer survivors (iCanADAPT Early). Psycho-Oncology. 2020. 29:76-85 , http://onlinelibrary.wiley.com/journal/10.1002/(ISSN)1099-1611 https://ovidsp.ovid.com/ovidweb.cgi?T=JS&CSC=Y&NEWS=N&PAGE=fulltext&D=emed20&AN=2003635356 |  | Other diagnosis (not depression/MDD) |
| 149 , Barroso, J., Madisetti, M., Mueller, M. A Feasibility Study to Develop and Test a Cognitive Behavioral Stress Management Mobile Health Application for HIV-Related Fatigue. Journal of Pain and Symptom Management. 2020. 59:242-253 , http://www.elsevier.com/locate/jpainsymman https://ovidsp.ovid.com/ovidweb.cgi?T=JS&CSC=Y&NEWS=N&PAGE=fulltext&D=emed20&AN=2003439622 | Other diagnosis (not depression/MDD) |  |
| 150 , Sanatkar, S., Baldwin, P. A., Huckvale, K., Clarke, J., Christensen, H., Harvey, S., Proudfoot, J. Using Cluster Analysis to Explore Engagement and e-Attainment as Emergent Behavior in Electronic Mental Health. Journal of medical Internet research. 2019. 21:e14728 , https://ovidsp.ovid.com/ovidweb.cgi?T=JS&CSC=Y&NEWS=N&PAGE=fulltext&D=emed20&AN=630003091 | Inappropriate study design |  |
| 151 , Enrique, A., Palacios, J. E., Ryan, H., Richards, D. Exploring the Relationship Between Usage and Outcomes of an Internet-Based Intervention for Individuals With Depressive Symptoms: Secondary Analysis of Data From a Randomized Controlled Trial. Journal of medical Internet research. 2019. 21:e12775 , https://ovidsp.ovid.com/ovidweb.cgi?T=JS&CSC=Y&NEWS=N&PAGE=fulltext&D=emed20&AN=628996636 | Include this article |  |
|  |  | Other diagnosis (not depression/MDD) |
| 152 , Clarke, J., Sanatkar, S., Baldwin, P. A., Fletcher, S., Gunn, J., Wilhelm, K., Campbell, L., Zwar, N., Harris, M., Lapsley, H., Hadzi-Pavlovic, D., Christensen, H., Proudfoot, J. A Web-Based Cognitive Behavior Therapy Intervention to Improve Social and Occupational Functioning in Adults With Type 2 Diabetes (The SpringboarD Trial): Randomized Controlled Trial. Journal of medical Internet research. 2019. 21:e12246 , https://ovidsp.ovid.com/ovidweb.cgi?T=JS&CSC=Y&NEWS=N&PAGE=fulltext&D=emed20&AN=627933163 | Other diagnosis (not depression/MDD) |  |
| 153 , Haga, S. M., Drozd, F., Lisoy, C., Wentzel-Larsen, T., Slinning, K. Mamma Mia - A randomized controlled trial of an internet-based intervention for perinatal depression. Psychological medicine. 2019. 49:1850-1858 , https://ovidsp.ovid.com/ovidweb.cgi?T=JS&CSC=Y&NEWS=N&PAGE=fulltext&D=emed20&AN=628880214 | Other diagnosis (not depression/MDD) |  |
| 154 , Oikonomidi, T., Vivot, A., Tran, V. T., Riveros, C., Robin, E., Ravaud, P. A Methodologic Systematic Review of Mobile Health Behavior Change Randomized Trials. American Journal of Preventive Medicine. 2019. 57:836-843 , http://www.elsevier.com/locate/amepre https://ovidsp.ovid.com/ovidweb.cgi?T=JS&CSC=Y&NEWS=N&PAGE=fulltext&D=emed20&AN=2003793836 | Inappropriate study design |  |
| 155 , Krieger, T., Urech, A., Duss, S. B., Blattler, L., Schmitt, W., Gast, H., Bassetti, C., Berger, T. A randomized controlled trial comparing guided internet-based multi-component treatment and internet-based guided sleep restriction treatment to care as usual in insomnia. Sleep Medicine. 2019. 62:43-52 , http://www.elsevier.com/inca/publications/store/6/2/0/2/8/2 https://ovidsp.ovid.com/ovidweb.cgi?T=JS&CSC=Y&NEWS=N&PAGE=fulltext&D=emed20&AN=2002916953 | Other diagnosis (not depression/MDD) |  |
| 156 , Murawski, B., Plotnikoff, R. C., Rayward, A. T., Oldmeadow, C., Vandelanotte, C., Brown, W. J., Duncan, M. J. Efficacy of an m-Health Physical Activity and Sleep Health Intervention for Adults: A Randomized Waitlist-Controlled Trial. American Journal of Preventive Medicine. 2019. 57:503-514 , http://www.elsevier.com/locate/amepre https://ovidsp.ovid.com/ovidweb.cgi?T=JS&CSC=Y&NEWS=N&PAGE=fulltext&D=emed20&AN=2002889148 | Other diagnosis (not depression/MDD) |  |
| 158 , Shu, C. Y., Watson, H. J., Anderson, R. A., Wade, T. D., Kane, R. T., Egan, S. J. A randomized controlled trial of unguided internet cognitive behaviour therapy for perfectionism in adolescents: Impact on risk for eating disorders. Behaviour Research and Therapy. 2019. 120 (no pagination):http://www.elsevier.com/locate/brat https://ovidsp.ovid.com/ovidweb.cgi?T=JS&CSC=Y&NEWS=N&PAGE=fulltext&D=emed20&AN=2002217651 | Other diagnosis (not depression/MDD) |  |
| 160 , Fox, R. S., Moreno, P. I., Yanez, B., Estabrook, R., Thomas, J., Bouchard, L. C., McGinty, H. L., Mohr, D. C., Begale, M. J., Flury, S. C., Perry, K. T., Kundu, S. D., Penedo, F. J. Integrating PROMIS computerized adaptive tests into a web-based intervention for prostate cancer. Health Psychology. 2019. 38:403-409 , http://www.apa.org/pubs/journals/hea/ https://ovidsp.ovid.com/ovidweb.cgi?T=JS&CSC=Y&NEWS=N&PAGE=fulltext&D=emed20&AN=627520618 | Other diagnosis (not depression/MDD) |  |
| 162 , Guercio, G. D., Thomas, M. E., Cisneros-Franco, J. M., Voss, P., Panizzutti, R., de Villers-Sidani, E. Improving cognitive training for schizophrenia using neuroplasticity enhancers: Lessons from decades of basic and clinical research. Schizophrenia Research. 2019. 207:80-92 , http://www.elsevier.com/locate/schres https://ovidsp.ovid.com/ovidweb.cgi?T=JS&CSC=Y&NEWS=N&PAGE=fulltext&D=emed20&AN=2000733370 | Other diagnosis (not depression/MDD) |  |
| 163 , Meyer, B., Weiss, M., Holtkamp, M., Arnold, S., Bruckner, K., Schroder, J., Scheibe, F., Nestoriuc, Y. Effects of an epilepsy-specific Internet intervention (Emyna) on depression: Results of the ENCODE randomized controlled trial. Epilepsia. 2019. 60:656-668 , http://onlinelibrary.wiley.com/journal/10.1111/(ISSN)1528-1167 https://ovidsp.ovid.com/ovidweb.cgi?T=JS&CSC=Y&NEWS=N&PAGE=fulltext&D=emed20&AN=626600688 | Include this article |  |
| 163 , Meyer, B., Weiss, M., Holtkamp, M., Arnold, S., Bruckner, K., Schroder, J., Scheibe, F., Nestoriuc, Y. Effects of an epilepsy-specific Internet intervention (Emyna) on depression: Results of the ENCODE randomized controlled trial. Epilepsia. 2019. 60:656-668 , http://onlinelibrary.wiley.com/journal/10.1111/(ISSN)1528-1167 https://ovidsp.ovid.com/ovidweb.cgi?T=JS&CSC=Y&NEWS=N&PAGE=fulltext&D=emed20&AN=626600688 |  | Other diagnosis (not depression/MDD) |
| 164 , Cavalera, C., Rovaris, M., Mendozzi, L., Pugnetti, L., Garegnani, M., Castelnuovo, G., Molinari, E., Pagnini, F. Online meditation training for people with multiple sclerosis: A randomized controlled trial. Multiple Sclerosis Journal. 2019. 25:610-617 , http://msj.sagepub.com/ https://ovidsp.ovid.com/ovidweb.cgi?T=JS&CSC=Y&NEWS=N&PAGE=fulltext&D=emed20&AN=621159260 | Other diagnosis (not depression/MDD) |  |
| 166 , Krieger, T., Reber, F., von Glutz, B., Urech, A., Moser, C. T., Schulz, A., Berger, T. An Internet-Based Compassion-Focused Intervention for Increased Self-Criticism: A Randomized Controlled Trial. Behavior Therapy. 2019. 50:430-445 , http://www.journals.elsevier.com/behavior-therapy/ https://ovidsp.ovid.com/ovidweb.cgi?T=JS&CSC=Y&NEWS=N&PAGE=fulltext&D=emed20&AN=2001103980 | Other diagnosis (not depression/MDD) |  |
| 168 , Garrido, S., Millington, C., Cheers, D., Boydell, K., Schubert, E., Meade, T., Nguyen, Q. V. What Works and What Doesn't Work? A Systematic Review of Digital Mental Health Interventions for Depression and Anxiety in Young People. Frontiers in Psychiatry. 2019. 10 (no pagination):http://www.frontiersin.org/Psychiatry https://ovidsp.ovid.com/ovidweb.cgi?T=JS&CSC=Y&NEWS=N&PAGE=fulltext&D=emed20&AN=629992010 | Inappropriate study design |  |
| 169 , Mills, L., Meiser, B., Ahmad, R., Schofield, P. R., Peate, M., Levitan, C., Trevena, L., Barlow-Stewart, K., Dobbins, T., Christensen, H., Sherman, K. A., Dunlop, K., Mitchell, P. B. A cluster randomized controlled trial of an online psychoeducational intervention for people with a family history of depression 11 Medical and Health Sciences 1117 Public Health and Health Services 11 Medical and Health Sciences 1103 Clinical Sciences. BMC Psychiatry. 2019. 19:https://bmcpsychiatry.biomedcentral.com/ https://ovidsp.ovid.com/ovidweb.cgi?T=JS&CSC=Y&NEWS=N&PAGE=fulltext&D=emed20&AN=625938961 | Other diagnosis (not depression/MDD) |  |
| 170 , Farrer, L. M., Gulliver, A., Katruss, N., Fassnacht, D. B., Kyrios, M., Batterham, P. J. A novel multi-component online intervention to improve the mental health of university students: Randomised controlled trial of the Uni Virtual Clinic. Internet Interventions. 2019. 18 (no pagination):http://www.journals.elsevier.com/internet-interventions/ https://ovidsp.ovid.com/ovidweb.cgi?T=JS&CSC=Y&NEWS=N&PAGE=fulltext&D=emed20&AN=2003762924 | Other diagnosis (not depression/MDD) |  |
| 171 , Fleming, T. M., Gillham, B., Bavin, L. M., Stasiak, K., Lewycka, S., Moore, J., Shepherd, M., Merry, S. N. SPARX-R computerized therapy among adolescents in youth offenders' program: Step-wise cohort study. Internet Interventions. 2019. 18 (no pagination):http://www.journals.elsevier.com/internet-interventions/ https://ovidsp.ovid.com/ovidweb.cgi?T=JS&CSC=Y&NEWS=N&PAGE=fulltext&D=emed20&AN=2003555580 | Other diagnosis (not depression/MDD) |  |
| 172 , Ramphos, E. S., Kelman, A. R., Stanley, M. L., Barrera, A. Z. Responding to women's needs and preferences in an online program to prevent postpartum depression. Internet Interventions. 2019. 18 (no pagination):http://www.journals.elsevier.com/internet-interventions/ https://ovidsp.ovid.com/ovidweb.cgi?T=JS&CSC=Y&NEWS=N&PAGE=fulltext&D=emed20&AN=2003323719 | Other diagnosis (not depression/MDD) |  |
| 173 , Moser, C., Bachem, R., Berger, T., Maercker, A. ZIEL: Internet-based self-help for adjustment problems: Results of a randomized controlled trial. Journal of Clinical Medicine. 2019. 8:https://www.mdpi.com/2077-0383/8/10/1655/pdf https://ovidsp.ovid.com/ovidweb.cgi?T=JS&CSC=Y&NEWS=N&PAGE=fulltext&D=emed20&AN=2002799556 | Other diagnosis (not depression/MDD) |  |
| 174 , Leigh, E., Clark, D. M. Online Social anxiety Cognitive therapy for Adolescents (OSCA): Protocol for a randomised controlled trial. Trials. 2019. 20:http://www.trialsjournal.com/home/ https://ovidsp.ovid.com/ovidweb.cgi?T=JS&CSC=Y&NEWS=N&PAGE=fulltext&D=emed20&AN=629512022 | Other diagnosis (not depression/MDD) |  |
| 175 , Depp, C. A., Perivoliotis, D., Holden, J., Dorr, J., Granholm, E. L. Single-session mobile-augmented intervention in serious mental illness: A three-arm randomized controlled trial. Schizophrenia Bulletin. 2019. 45:752-762 , http://schizophreniabulletin.oxfordjournals.org/ https://ovidsp.ovid.com/ovidweb.cgi?T=JS&CSC=Y&NEWS=N&PAGE=fulltext&D=emed20&AN=629293614 | Other diagnosis (not depression/MDD) |  |
| 176 , Gruwez, A., Bruyneel, A. V., Bruyneel, M. The validity of two commercially-available sleep trackers and actigraphy for assessment of sleep parameters in obstructive sleep apnea patients. PLoS ONE. 2019. 14:https://journals.plos.org/plosone/article/file?id=10.1371/journal.pone.0210569&type=printable https://ovidsp.ovid.com/ovidweb.cgi?T=JS&CSC=Y&NEWS=N&PAGE=fulltext&D=emed20&AN=625826185 | Other diagnosis (not depression/MDD) |  |
| 177 , Papadatou-Pastou, M., Campbell-Thompson, L., Barley, E., Haddad, M., Lafarge, C., McKeown, E., Simeonov, L., Tzotzoli, P. Exploring the feasibility and acceptability of the contents, design, and functionalities of an online intervention promoting mental health, wellbeing, and study skills in Higher Education students. International Journal of Mental Health Systems. 2019. 13:http://www.ijmhs.com/ https://ovidsp.ovid.com/ovidweb.cgi?T=JS&CSC=Y&NEWS=N&PAGE=fulltext&D=emed20&AN=628648089 | Other diagnosis (not depression/MDD) |  |
| 179 , Kubo, A., Kurtovich, E., McGinnis, M., Aghaee, S., Altschuler, A., Quesenberry, C., Kolevska, T., Avins, A. L. A Randomized Controlled Trial of mHealth Mindfulness Intervention for Cancer Patients and Informal Cancer Caregivers: A Feasibility Study Within an Integrated Health Care Delivery System. Integrative Cancer Therapies. 2019. 18:http://ict.sagepub.com/ https://ovidsp.ovid.com/ovidweb.cgi?T=JS&CSC=Y&NEWS=N&PAGE=fulltext&D=emed20&AN=627801290 | Other diagnosis (not depression/MDD) |  |
| 180 , Takahashi, K., Takada, K., Hirao, K. Feasibility and preliminary efficacy of a smartphone application intervention for subthreshold depression. Early Intervention in Psychiatry. 2019. 13:133-136 , http://onlinelibrary.wiley.com/journal/10.1111/(ISSN)1751-7893 https://ovidsp.ovid.com/ovidweb.cgi?T=JS&CSC=Y&NEWS=N&PAGE=fulltext&D=emed20&AN=625810133 | Other diagnosis (not depression/MDD) |  |
| 182 , Wright, J. H., Owen, J. J., Richards, D., Eells, T. D., Richardson, T., Brown, G. K., Barrett, M., Rasku, M. A., Polser, G., Thase, M. E. Computer-assisted cognitive-behavior therapy for depression: A systematic review and meta-analysis. Journal of Clinical Psychiatry. 2019. 80:https://www.psychiatrist.com/JCP/article/_layouts/ppp.psych.controls/BinaryViewer.ashx?Article=/JCP/article/Pages/2019/v80/18r12188.aspx&Type=Article https://ovidsp.ovid.com/ovidweb.cgi?T=JS&CSC=Y&NEWS=N&PAGE=fulltext&D=emed20&AN=2002075960 | Inappropriate study design |  |
| 183 , Krusche, A., Bradbury, K., Corbett, T., Barnett, J., Stuart, B., Yao, G. L., Bacon, R., Bohning, D., Cheetham-Blake, T., Eccles, D., Foster, C., Geraghty, A. W. A., Leydon, G., Muller, A., Neal, R. D., Osborne, R., Rathod, S., Richardson, A., Sharman, G., Summers, K., Watson, E., Wilde, L., Wilkinson, C., Yardley, L., Little, P. Renewed: Protocol for a randomised controlled trial of a digital intervention to support quality of life in cancer survivors. BMJ Open. 2019. 9:http://bmjopen.bmj.com/content/early/by/section https://ovidsp.ovid.com/ovidweb.cgi?T=JS&CSC=Y&NEWS=N&PAGE=fulltext&D=emed20&AN=626568362 | Other diagnosis (not depression/MDD) |  |
| 184 , Ciani, O., Cucciniello, M., Petracca, F., Apolone, G., Merlini, G., Novello, S., Pedrazzoli, P., Zilembo, N., Broglia, C., Capelletto, E., Garassino, M., Nicod, E., Tarricone, R. Lung Cancer App (LuCApp) study protocol: A randomised controlled trial to evaluate a mobile supportive care app for patients with metastatic lung cancer. BMJ Open. 2019. 9:http://bmjopen.bmj.com/content/early/by/section https://ovidsp.ovid.com/ovidweb.cgi?T=JS&CSC=Y&NEWS=N&PAGE=fulltext&D=emed20&AN=626420251 | Other diagnosis (not depression/MDD) |  |
| 185 , Kuchler, A. M., Albus, P., Ebert, D. D., Baumeister, H. Effectiveness of an internet-based intervention for procrastination in college students (StudiCare Procrastination): Study protocol of a randomized controlled trial. Internet Interventions. 2019. 17 (no pagination):http://www.journals.elsevier.com/internet-interventions/ https://ovidsp.ovid.com/ovidweb.cgi?T=JS&CSC=Y&NEWS=N&PAGE=fulltext&D=emed20&AN=2001788542 | Other diagnosis (not depression/MDD) |  |
| 186 , Spencer, L., Schmidt-Hantke, J., Allen, K., Gordon, G., Potterton, R., Musiat, P., Hagner, F., Beintner, I., Vollert, B., Nacke, B., Gorlich, D., Beecham, J., Bonin, E. M., Jacobi, C., Schmidt, U. A web-based intervention for carers of individuals with anorexia nervosa (We Can): Trial protocol of a randomised controlled trial investigating the effectiveness of different levels of support. Internet Interventions. 2019. 16:76-85 , http://www.journals.elsevier.com/internet-interventions/ https://ovidsp.ovid.com/ovidweb.cgi?T=JS&CSC=Y&NEWS=N&PAGE=fulltext&D=emed20&AN=2000643446 | Other diagnosis (not depression/MDD) |  |
| 187 , Kootker, J. A., van Heugten, C. M., Kral, B., Rasquin, S. M., Geurts, A. C., Fasotti, L. Caregivers' effects of augmented cognitive-behavioural therapy for post-stroke depressive symptoms in patients: secondary analyses to a randomized controlled trial. Clinical rehabilitation. 2019. 33:1056-1065 , https://ovidsp.ovid.com/ovidweb.cgi?T=JS&CSC=Y&NEWS=N&PAGE=fulltext&D=emed20&AN=628168491 | Other diagnosis (not depression/MDD) |  |
| 189 , Lappalainen, P., Pakkala, I., Nikander, R. CareACT - internet-based intervention for enhancing the psychological well-being of elderly caregivers - a study protocol of a controlled trial. BMC geriatrics. 2019. 19:72 , https://ovidsp.ovid.com/ovidweb.cgi?T=JS&CSC=Y&NEWS=N&PAGE=fulltext&D=emed20&AN=626688260 | Other diagnosis (not depression/MDD) |  |
| 190 , Ramalho, S., Saint-Maurice, P. F., Silva, D., Mansilha, H. F., Silva, C., Goncalves, S., Machado, P., Conceicao, E. APOLO-Teens, a web-based intervention for treatment-seeking adolescents with overweight or obesity: study protocol and baseline characterization of a Portuguese sample. Eating and Weight Disorders. 2020. 25:453-463 , http://link.springer.com/journal/40519 https://ovidsp.ovid.com/ovidweb.cgi?T=JS&CSC=Y&NEWS=N&PAGE=fulltext&D=emed19&AN=625382650 | Other diagnosis (not depression/MDD) |  |
| 191 , Bucker, L., Bierbrodt, J., Hand, I., Wittekind, C., Moritz, S. Correction: Effects of a depression-focused internet intervention in slot machine gamblers: A randomized controlled trial (PLoS ONE (2018) 13: 6 (e0198859) DOI: 10.1371/journal.pone.0198859). PLoS ONE. 2018. 13:http://journals.plos.org/plosone/article/file?id=10.1371/journal.pone.0203145&type=printable https://ovidsp.ovid.com/ovidweb.cgi?T=JS&CSC=Y&NEWS=N&PAGE=fulltext&D=emed19&AN=623586950 | Other diagnosis (not depression/MDD) |  |
| 192 , Gellatly, J., Chisnall, L., Seccombe, N., Ragan, K., Lidbetter, N., Cavanagh, K. @Home eTherapy Service for People with Common Mental Health Problems: an Evaluation. Behavioural and cognitive psychotherapy. 2018. 46:115-120 , https://ovidsp.ovid.com/ovidweb.cgi?T=JS&CSC=Y&NEWS=N&PAGE=fulltext&D=emed19&AN=624805332 | Other diagnosis (not depression/MDD) |  |
| 193 , Tiburcio, M., Lara, M. A., Martinez, N., Fernandez, M., Aguilar, A. Web-Based Intervention to Reduce Substance Abuse and Depression: A Three Arm Randomized Trial in Mexico. Substance use & misuse. 2018. 53:2220-2231 , https://ovidsp.ovid.com/ovidweb.cgi?T=JS&CSC=Y&NEWS=N&PAGE=fulltext&D=emed19&AN=627494701 | Include this article |  |
| 193 , Tiburcio, M., Lara, M. A., Martinez, N., Fernandez, M., Aguilar, A. Web-Based Intervention to Reduce Substance Abuse and Depression: A Three Arm Randomized Trial in Mexico. Substance use & misuse. 2018. 53:2220-2231 , https://ovidsp.ovid.com/ovidweb.cgi?T=JS&CSC=Y&NEWS=N&PAGE=fulltext&D=emed19&AN=627494701 |  | Other diagnosis (not depression/MDD) |
| 195 , Zarski, A. C., Berking, M., Reis, D., Lehr, D., Buntrock, C., Schwarzer, R., Ebert, D. D. Turning Good Intentions Into Actions by Using the Health Action Process Approach to Predict Adherence to Internet-Based Depression Prevention: Secondary Analysis of a Randomized Controlled Trial. Journal of medical Internet research. 2018. 20:e9 , https://ovidsp.ovid.com/ovidweb.cgi?T=JS&CSC=Y&NEWS=N&PAGE=fulltext&D=emed19&AN=626548231 | Include this article |  |
| 195 , Zarski, A. C., Berking, M., Reis, D., Lehr, D., Buntrock, C., Schwarzer, R., Ebert, D. D. Turning Good Intentions Into Actions by Using the Health Action Process Approach to Predict Adherence to Internet-Based Depression Prevention: Secondary Analysis of a Randomized Controlled Trial. Journal of medical Internet research. 2018. 20:e9 , https://ovidsp.ovid.com/ovidweb.cgi?T=JS&CSC=Y&NEWS=N&PAGE=fulltext&D=emed19&AN=626548231 |  | Other diagnosis (not depression/MDD) |
| 196 , Yap, M. B. H., Mahtani, S., Rapee, R. M., Nicolas, C., Lawrence, K. A., Mackinnon, A., Jorm, A. F. A Tailored Web-Based Intervention to Improve Parenting Risk and Protective Factors for Adolescent Depression and Anxiety Problems: Postintervention Findings From a Randomized Controlled Trial. Journal of medical Internet research. 2018. 20:e17 , https://ovidsp.ovid.com/ovidweb.cgi?T=JS&CSC=Y&NEWS=N&PAGE=fulltext&D=emed19&AN=626546628 | Inappropriate study design |  |
| 197 , Schleider, J., Weisz, J. A single-session growth mindset intervention for adolescent anxiety and depression: 9-month outcomes of a randomized trial. Journal of child psychology and psychiatry, and allied disciplines. 2018. 59:160-170 , https://ovidsp.ovid.com/ovidweb.cgi?T=JS&CSC=Y&NEWS=N&PAGE=fulltext&D=emed19&AN=625816609 | Inappropriate study design |  |
| 198 , Hershner, S., O'Brien, L. M. The impact of a randomized sleep education intervention for college students. Journal of Clinical Sleep Medicine. 2018. 14:337-347 , http://jcsm.aasm.org/ViewAbstract.aspx?pid=31205 https://ovidsp.ovid.com/ovidweb.cgi?T=JS&CSC=Y&NEWS=N&PAGE=fulltext&D=emed19&AN=621416813 | Other diagnosis (not depression/MDD) |  |
| 199 , Young, C. L., Trapani, K., Dawson, S., O'Neil, A., Kay-Lambkin, F., Berk, M., Jacka, F. N. Efficacy of online lifestyle interventions targeting lifestyle behaviour change in depressed populations: A systematic review. Australian and New Zealand Journal of Psychiatry. 2018. 52:834-846 , http://anp.sagepub.com/content/by/year https://ovidsp.ovid.com/ovidweb.cgi?T=JS&CSC=Y&NEWS=N&PAGE=fulltext&D=emed19&AN=623656754 | Inappropriate study design |  |
| 200 , Urech, C., Grossert, A., Alder, J., Scherer, S., Handschin, B., Kasenda, B., Borislavova, B., Degen, S., Erb, J., Faessler, A., Gattlen, L., Schibli, S., Werndli, C., Gaab, J., Berger, T., Zumbrunn, T., Hess, V. Web-based stress management for newly diagnosed patients with cancer (STREAM): A randomized, wait-list controlled intervention study. Journal of Clinical Oncology. 2018. 36:780-788 , http://ascopubs.org/doi/pdf/10.1200/JCO.2017.74.8491 https://ovidsp.ovid.com/ovidweb.cgi?T=JS&CSC=Y&NEWS=N&PAGE=fulltext&D=emed19&AN=621093876 | Other diagnosis (not depression/MDD) |  |
| 201 , Ben-Zeev, D., Brian, R. M., Jonathan, G., Razzano, L., Pashka, N., Carpenter-Song, E., Drake, R. E., Scherer, E. A. Mobile health (mHealth) versus clinic-based group intervention for people with serious mental illness: A randomized controlled trial. Psychiatric Services. 2018. 69:978-985 , https://ps.psychiatryonline.org/doi/pdf/10.1176/appi.ps.201800063 https://ovidsp.ovid.com/ovidweb.cgi?T=JS&CSC=Y&NEWS=N&PAGE=fulltext&D=emed19&AN=623753115 | Other diagnosis (not depression/MDD) |  |
| 202 , Fletcher, K., Foley, F., Thomas, N., Michalak, E., Berk, L., Berk, M., Bowe, S., Cotton, S., Engel, L., Johnson, S. L., Jones, S., Kyrios, M., Lapsley, S., Mihalopoulos, C., Perich, T., Murray, G. Web-based intervention to improve quality of life in late stage bipolar disorder (ORBIT): Randomised controlled trial protocol. BMC Psychiatry. 2018. 18:https://bmcpsychiatry.biomedcentral.com/ https://ovidsp.ovid.com/ovidweb.cgi?T=JS&CSC=Y&NEWS=N&PAGE=fulltext&D=emed19&AN=622989213 | Other diagnosis (not depression/MDD) |  |
| 203 , Chandra, P. S., Parameshwaran, S., Satyanarayana, V. A., Varghese, M., Liberti, L., Duggal, M., Singh, P., Jeon, S., Reynolds, N. R. I have no peace of mind-psychosocial distress expressed by rural women living with HIV in India as part of a mobile health intervention-a qualitative study. Archives of Women's Mental Health. 2018. 21:525-531 , http://www.springer.co.at/springer.py?Page=40&Key=545&cat=11&id_journal=40 https://ovidsp.ovid.com/ovidweb.cgi?T=JS&CSC=Y&NEWS=N&PAGE=fulltext&D=emed19&AN=621305376 | Other diagnosis (not depression/MDD) |  |
| 204 , Bucker, L., Bierbrodt, J., Hand, I., Wittekind, C., Moritz, S. Effects of a depression-focused internet intervention in slot machine gamblers: A randomized controlled trial. PLoS ONE. 2018. 13:http://journals.plos.org/plosone/article/file?id=10.1371/journal.pone.0198859&type=printable https://ovidsp.ovid.com/ovidweb.cgi?T=JS&CSC=Y&NEWS=N&PAGE=fulltext&D=emed19&AN=622493255 | Other diagnosis (not depression/MDD) |  |
| 205 , Anastasiadou, D., Lupianez-Villanueva, F., Fauli, C., Arcal Cunillera, J., Serrano-Troncoso, E. Cost-effectiveness of the mobile application TCApp combined with face-to-face CBT treatment compared to face-to-face CBT treatment alone for patients with an eating disorder: Study protocol of a multi-centre randomised controlled trial. BMC Psychiatry. 2018. 18:https://bmcpsychiatry.biomedcentral.com/ https://ovidsp.ovid.com/ovidweb.cgi?T=JS&CSC=Y&NEWS=N&PAGE=fulltext&D=emed19&AN=621918555 | Other diagnosis (not depression/MDD) |  |
| 207 , Boele, F. W., Klein, M., Verdonck-de Leeuw, I. M., Cuijpers, P., Heimans, J. J., Snijders, T. J., Vos, M., Bosma, I., Tijssen, C. C., Reijneveld, J. C. Internet-based guided self-help for glioma patients with depressive symptoms: a randomized controlled trial. Journal of Neuro-Oncology. 2018. 137:191-203 , http://www.wkap.nl/journalhome.htm/0167-594X https://ovidsp.ovid.com/ovidweb.cgi?T=JS&CSC=Y&NEWS=N&PAGE=fulltext&D=emed19&AN=619735003 | Include this article |  |
|  |  | Other diagnosis (not depression/MDD) |
| 208 , Hennemann, S., Bohme, K., Baumeister, H., Bendig, E., Kleinstauber, M., Ebert, D. D., Witthoft, M. Efficacy of a guided internet-based intervention (iSOMA) for somatic symptoms and related distress in university students: Study protocol of a randomised controlled trial. BMJ Open. 2018. 8:http://bmjopen.bmj.com/content/early/by/section https://ovidsp.ovid.com/ovidweb.cgi?T=JS&CSC=Y&NEWS=N&PAGE=fulltext&D=emed19&AN=625826378 | Other diagnosis (not depression/MDD) |  |
| 209 , Smith, M., Jones, M. P., Dotson, M. M., Wolinsky, F. D. Computerized cognitive training to improve mood in senior living settings: Design of a randomized controlled trial. Open Access Journal of Clinical Trials. 2018. 10:29-41 , http://www.dovepress.com/open-access-journal-of-clinical-trials-journal https://ovidsp.ovid.com/ovidweb.cgi?T=JS&CSC=Y&NEWS=N&PAGE=fulltext&D=emed19&AN=2001839949 | Other diagnosis (not depression/MDD) |  |
| 215 , Kladnitski, N., Smith, J., Allen, A., Andrews, G., Newby, J. M. Online mindfulness-enhanced cognitive behavioural therapy for anxiety and depression: Outcomes of a pilot trial. Internet Interventions. 2018. 13:41-50 , http://www.journals.elsevier.com/internet-interventions/ https://ovidsp.ovid.com/ovidweb.cgi?T=JS&CSC=Y&NEWS=N&PAGE=fulltext&D=emed19&AN=2000922137 | Other diagnosis (not depression/MDD) |  |
| 217 , Ebert, D. D., Buntrock, C., Lehr, D., Smit, F., Riper, H., Baumeister, H., Cuijpers, P., Berking, M. Effectiveness of Web- and Mobile-Based Treatment of Subthreshold Depression With Adherence-Focused Guidance: A Single-Blind Randomized Controlled Trial. Behavior Therapy. 2018. 49:71-83 , http://www.journals.elsevier.com/behavior-therapy/ https://ovidsp.ovid.com/ovidweb.cgi?T=JS&CSC=Y&NEWS=N&PAGE=fulltext&D=emed19&AN=616825512 | Include this article |  |
|  |  | Other diagnosis (not depression/MDD) |
| 218 , Weil, R., Feist, A., Moritz, S., Wittekind, C. E. Approaching contamination-related stimuli with an implicit Approach-Avoidance Task: Can it reduce OCD symptoms? An online pilot study. Journal of Behavior Therapy and Experimental Psychiatry. 2017. 57:180-188 , http://www.elsevier.com/locate/jbtep https://ovidsp.ovid.com/ovidweb.cgi?T=JS&CSC=Y&NEWS=N&PAGE=fulltext&D=emed18&AN=616649627 | Other diagnosis (not depression/MDD) |  |
| 221 , Elison, S., Jones, A., Ward, J., Davies, G., Dugdale, S. Examining effectiveness of tailorable computer-assisted therapy programmes for substance misuse: Programme usage and clinical outcomes data from Breaking Free Online. Addictive Behaviors. 2017. 74:140-147 , http://www.elsevier.com/locate/addictbeh https://ovidsp.ovid.com/ovidweb.cgi?T=JS&CSC=Y&NEWS=N&PAGE=fulltext&D=emed18&AN=616859290 | Other diagnosis (not depression/MDD) |  |
| 223 , Cooney, P., Jackman, C., Coyle, D., O'Reilly, G. Computerised cognitive-behavioural therapy for adults with intellectual disability: Randomised controlled trial. British Journal of Psychiatry. 2017. 211:95-102 , http://bjp.rcpsych.org/content/211/2/95.full-text.pdf https://ovidsp.ovid.com/ovidweb.cgi?T=JS&CSC=Y&NEWS=N&PAGE=fulltext&D=emed18&AN=617691201 | Other diagnosis (not depression/MDD) |  |
| 224 , Van De Ven, R. M., Murre, J. M. J., Buitenweg, J. I. V., Veltman, D. J., Aaronson, J. A., Nijboer, T. C. W., Kruiper-Doesborgh, S. J. C., Van Bennekom, C. A. M., Ridderinkhof, K. R., Schmand, B. The influence of computer-based cognitive flexibility training on subjective cognitive well-being after stroke: A multi-center randomized controlled trial. PLoS ONE. 2017. 12:http://journals.plos.org/plosone/article/file?id=10.1371/journal.pone.0187582&type=printable https://ovidsp.ovid.com/ovidweb.cgi?T=JS&CSC=Y&NEWS=N&PAGE=fulltext&D=emed18&AN=619286368 | Other diagnosis (not depression/MDD) |  |
| 225 , Lin, J., Sander, L., Paganini, S., Schlicker, S., Ebert, D., Berking, M., Bengel, J., Nobis, S., Lehr, D., Mittag, O., Riper, H., Baumeister, H. Effectiveness and cost-effectiveness of a guided internet- and mobile-based depression intervention for individuals with chronic back pain: Protocol of a multi-centre randomised controlled trial. BMJ Open. 2017. 7:http://bmjopen.bmj.com/content/early/by/section https://ovidsp.ovid.com/ovidweb.cgi?T=JS&CSC=Y&NEWS=N&PAGE=fulltext&D=emed18&AN=623794038 |  | Other diagnosis (not depression/MDD) |
|  | Include this article |  |
| 226 , McCabe, C., McCann, M., Brady, A. M. Computer and mobile technology interventions for self-management in chronic obstructive pulmonary disease. Cochrane Database of Systematic Reviews. 2017. 2017:http://as.wiley.com/WileyCDA/Brand/id-6.html https://ovidsp.ovid.com/ovidweb.cgi?T=JS&CSC=Y&NEWS=N&PAGE=fulltext&D=emed18&AN=616317838 | Inappropriate study design |  |
| 227 , Lauder, S., Cosgrove, V. E., Gliddon, E., Grimm, D., Dodd, S., Berk, L., Castle, D., Suppes, T. S., Berk, M. Progressing MoodSwings. The upgrade and evaluation of MoodSwings 2.0: An online intervention for bipolar disorder. Contemporary Clinical Trials. 2017. 56:18-24 , http://www.elsevier.com/wps/find/journaldescription.cws_home/704636/description#description https://ovidsp.ovid.com/ovidweb.cgi?T=JS&CSC=Y&NEWS=N&PAGE=fulltext&D=emed18&AN=614845214 | Other diagnosis (not depression/MDD) |  |
| 228 , Gleeson, J., Lederman, R., Herrman, H., Koval, P., Eleftheriadis, D., Bendall, S., Cotton, S. M., Alvarez-Jimenez, M. Moderated online social therapy for carers of young people recovering from first-episode psychosis: Study protocol for a randomised controlled trial. Trials. 2017. 18:http://www.trialsjournal.com/home/ https://ovidsp.ovid.com/ovidweb.cgi?T=JS&CSC=Y&NEWS=N&PAGE=fulltext&D=emed18&AN=614271809 | Other diagnosis (not depression/MDD) |  |
| 229 , Sundstrom, C., Kraepelien, M., Eek, N., Fahlke, C., Kaldo, V., Berman, A. H. High-intensity therapist-guided internetbased cognitive behavior therapy for alcohol use disorder: A pilot study. BMC Psychiatry. 2017. 17:http://www.biomedcentral.com/bmcpsychiatr/ https://ovidsp.ovid.com/ovidweb.cgi?T=JS&CSC=Y&NEWS=N&PAGE=fulltext&D=emed18&AN=616587093 | Other diagnosis (not depression/MDD) |  |
| 230 , Rosso, I. M., Killgore, W. D. S., Olson, E. A., Webb, C. A., Fukunaga, R., Auerbach, R. P., Gogel, H., Buchholz, J. L., Rauch, S. L. Internet-based cognitive behavior therapy for major depressive disorder: A randomized controlled trial. Depression and Anxiety. 2017. 34:236-245 , http://www.interscience.wiley.com/jpages/1091-4269 https://ovidsp.ovid.com/ovidweb.cgi?T=JS&CSC=Y&NEWS=N&PAGE=fulltext&D=emed18&AN=613919312 |  | Inappropriate outcomes |
|  | Include this article |  |
| 231 , Johansson, R., Hesslow, T., Ljotsson, B., Jansson, A., Jonsson, L., Fardig, S., Karlsson, J., Hesser, H., Frederick, R. J., Lilliengren, P., Carlbring, P., Andersson, G. Internet-based affect-focused psychodynamic therapy for social anxiety disorder: A randomized controlled trial with 2-year follow-up. Psychotherapy. 2017. 54:351-360 , http://ttp://www.apa.org/pubs/journals/pst/ https://ovidsp.ovid.com/ovidweb.cgi?T=JS&CSC=Y&NEWS=N&PAGE=fulltext&D=emed18&AN=619868258 | Other diagnosis (not depression/MDD) |  |
| 232 , Iakimova, G., Dimitrova, S., Burte, T. Can we do therapy without a therapist? Active components of computer-based CBT for depression. Encephale. 2017. 43:582-593 , http://www.elsevier.com/wps/find/journaldescription.cws_home/709657/description#description https://ovidsp.ovid.com/ovidweb.cgi?T=JS&CSC=Y&NEWS=N&PAGE=fulltext&D=emed18&AN=613182617 | Inappropriate study design |  |
| 233 , Fann, J. R., Hong, F., Halpenny, B., Blonquist, T. M., Berry, D. L. Psychosocial outcomes of an electronic self-report assessment and self-care intervention for patients with cancer: a randomized controlled trial. Psycho-Oncology. 2017. 26:1866-1871 , http://onlinelibrary.wiley.com/journal/10.1002/(ISSN)1099-1611 https://ovidsp.ovid.com/ovidweb.cgi?T=JS&CSC=Y&NEWS=N&PAGE=fulltext&D=emed18&AN=612213633 | Other diagnosis (not depression/MDD) |  |
| 234 , Schroder, J., Jelinek, L., Moritz, S. A randomized controlled trial of a transdiagnostic Internet intervention for individuals with panic and phobias - One size fits all. Journal of Behavior Therapy and Experimental Psychiatry. 2017. 54:17-24 , http://www.elsevier.com/locate/jbtep https://ovidsp.ovid.com/ovidweb.cgi?T=JS&CSC=Y&NEWS=N&PAGE=fulltext&D=emed18&AN=610462847 | Other diagnosis (not depression/MDD) |  |
| 235 , Hightow-Weidman, L., LeGrand, S., Choi, S. K., Egger, J., Hurt, C. B., Muessig, K. E. Exploring the HIV continuum of care among young black MSM. PLoS ONE. 2017. 12:http://journals.plos.org/plosone/article/file?id=10.1371/journal.pone.0179688&type=printable https://ovidsp.ovid.com/ovidweb.cgi?T=JS&CSC=Y&NEWS=N&PAGE=fulltext&D=emed18&AN=617004559 | Other diagnosis (not depression/MDD) |  |
| 236 , Beukes, E. W., Allen, P. M., Manchaiah, V., Baguley, D. M., Andersson, G. Internet-based intervention for tinnitus: Outcome of a single-group open trial. Journal of the American Academy of Audiology. 2017. 28:340-351 , http://docserver.ingentaconnect.com/deliver/connect/aaa/10500545/v28n4/s7.pdf?expires=1492845506&id=90502125&titleid=72010016&accname=Elsevier+BV&checksum=AF541AE7BAD1595AAA68CD88C89885C3 https://ovidsp.ovid.com/ovidweb.cgi?T=JS&CSC=Y&NEWS=N&PAGE=fulltext&D=emed18&AN=615605619 | Other diagnosis (not depression/MDD) |  |
| 237 , Newby, J. M., Mewton, L., Andrews, G. Transdiagnostic versus disorder-specific internet-delivered cognitive behaviour therapy for anxiety and depression in primary care. Journal of Anxiety Disorders. 2017. 46:25-34 , http://www.elsevier.com/inca/publications/store/8/0/1 https://ovidsp.ovid.com/ovidweb.cgi?T=JS&CSC=Y&NEWS=N&PAGE=fulltext&D=emed18&AN=613684493 |  | Inappropriate study design |
|  | Include this article |  |
| 239 , Hetrick, S. E., Yuen, H. P., Bailey, E., Cox, G. R., Templer, K., Rice, S. M., Bendall, S., Robinson, J. Internet-based cognitive behavioural therapy for young people with suicide-related behaviour (Reframe-IT): a randomised controlled trial. Evidence-based mental health. 2017. 20:76-82 , https://ovidsp.ovid.com/ovidweb.cgi?T=JS&CSC=Y&NEWS=N&PAGE=fulltext&D=emed18&AN=623335135 | Inappropriate study design |  |
| 240 , Dodd, A. L., Mallinson, S., Griffiths, M., Morriss, R., Jones, S. H., Lobban, F. Users' experiences of an online intervention for bipolar disorder: important lessons for design and evaluation. Evidence-based mental health. 2017. 20:133-139 , https://ovidsp.ovid.com/ovidweb.cgi?T=JS&CSC=Y&NEWS=N&PAGE=fulltext&D=emed18&AN=623336399 | Other diagnosis (not depression/MDD) |  |
| 241 , Martorella, G., Boitor, M., Berube, M., Fredericks, S., Le May, S., Gelinas, C. Tailored Web-Based Interventions for Pain: Systematic Review and Meta-Analysis. Journal of medical Internet research. 2017. 19:e385 , https://ovidsp.ovid.com/ovidweb.cgi?T=JS&CSC=Y&NEWS=N&PAGE=fulltext&D=emed18&AN=621333053 | Inappropriate study design |  |
| 242 , Perry, Y., Werner-Seidler, A., Calear, A., Mackinnon, A., King, C., Scott, J., Merry, S., Fleming, T., Stasiak, K., Christensen, H., Batterham, P. J. Preventing Depression in Final Year Secondary Students: School-Based Randomized Controlled Trial. Journal of medical Internet research. 2017. 19:e369 , https://ovidsp.ovid.com/ovidweb.cgi?T=JS&CSC=Y&NEWS=N&PAGE=fulltext&D=emed18&AN=621332072 | Inappropriate study design |  |
| 243 , Berger, T., Urech, A., Krieger, T., Stolz, T., Schulz, A., Vincent, A., Moser, C. T., Moritz, S., Meyer, B. Effects of a transdiagnostic unguided Internet intervention ('velibra') for anxiety disorders in primary care: results of a randomized controlled trial. Psychological medicine. 2017. 47:67-80 , https://ovidsp.ovid.com/ovidweb.cgi?T=JS&CSC=Y&NEWS=N&PAGE=fulltext&D=emed18&AN=619689201 | Other diagnosis (not depression/MDD) |  |
| 244 , Carleton, R. N., Teale Sapach, M. J., Oriet, C., LeBouthillier, D. M. Online attention modification for social anxiety disorder: replication of a randomized controlled trial. Cognitive behaviour therapy. 2017. 46:44-59 , https://ovidsp.ovid.com/ovidweb.cgi?T=JS&CSC=Y&NEWS=N&PAGE=fulltext&D=emed18&AN=618712184 | Other diagnosis (not depression/MDD) |  |
| 245 , Hollis, C., Falconer, C. J., Martin, J. L., Whittington, C., Stockton, S., Glazebrook, C., Davies, E. B. Annual Research Review: Digital health interventions for children and young people with mental health problems - a systematic and meta-review. Journal of child psychology and psychiatry, and allied disciplines. 2017. 58:474-503 , https://ovidsp.ovid.com/ovidweb.cgi?T=JS&CSC=Y&NEWS=N&PAGE=fulltext&D=emed18&AN=618601306 | Inappropriate study design |  |
| 246 , Fernandez-Alvarez, J., Diaz-Garcia, A., Gonzalez-Robles, A., Banos, R., Garcia-Palacios, A., Botella, C. Dropping out of a transdiagnostic online intervention: A qualitative analysis of client's experiences. Internet Interventions. 2017. 10:29-38 , http://www.journals.elsevier.com/internet-interventions/ https://ovidsp.ovid.com/ovidweb.cgi?T=JS&CSC=Y&NEWS=N&PAGE=fulltext&D=emed18&AN=618471876 | Inappropriate study design |  |
| 247 , Meyer, B., Weiss, M., Holtkamp, M., Arnold, S., Bruckner, K., Schroder, J., Scheibe, F., Nestoriuc, Y. Protocol for the ENCODE trial: Evaluating a novel online depression intervention for persons with epilepsy. BMC Psychiatry. 2017. 17:http://www.biomedcentral.com/bmcpsychiatr/ https://ovidsp.ovid.com/ovidweb.cgi?T=JS&CSC=Y&NEWS=N&PAGE=fulltext&D=emed18&AN=614311835 | Include this article |  |
|  |  | Other diagnosis (not depression/MDD) |
| 248 , Kootker, J. A., Rasquin, S. M. C., Lem, F. C., van Heugten, C. M., Fasotti, L., Geurts, A. C. H. Augmented Cognitive Behavioral Therapy for Poststroke Depressive Symptoms: A Randomized Controlled Trial. Archives of Physical Medicine and Rehabilitation. 2017. 98:687-694 , http://www.elsevier.com/inca/publications/store/6/2/3/3/5/4/index.htt https://ovidsp.ovid.com/ovidweb.cgi?T=JS&CSC=Y&NEWS=N&PAGE=fulltext&D=emed18&AN=614277083 | Other diagnosis (not depression/MDD) |  |
| 249 , Mira, A., Breton-Lopez, J., Garcia-Palacios, A., Quero, S., Banos, R. M., Botella, C. An internet-based program for depressive symptoms using human and automated support: A randomized controlled trial. Neuropsychiatric Disease and Treatment. 2017. 13:987-1006 , https://www.dovepress.com/getfile.php?fileID=35774 https://ovidsp.ovid.com/ovidweb.cgi?T=JS&CSC=Y&NEWS=N&PAGE=fulltext&D=emed18&AN=615479519 | Other diagnosis (not depression/MDD) |  |
| 250 , Twomey, C., O'Reilly, G. Effectiveness of a freely available computerised cognitive behavioural therapy programme (MoodGYM) for depression: Meta-analysis. Australian and New Zealand Journal of Psychiatry. 2017. 51:260-269 , http://anp.sagepub.com/content/by/year https://ovidsp.ovid.com/ovidweb.cgi?T=JS&CSC=Y&NEWS=N&PAGE=fulltext&D=emed18&AN=614517876 | Inappropriate study design |  |
| 251 , Munoz, R. F., Leykin, Y., Barrera, A. Z., Brown, C. H., Bunge, E. L. The impact of phone calls on follow-up rates in an online depression prevention study. Internet Interventions. 2017. 8:10-14 , http://www.journals.elsevier.com/internet-interventions/ https://ovidsp.ovid.com/ovidweb.cgi?T=JS&CSC=Y&NEWS=N&PAGE=fulltext&D=emed18&AN=614600147 |  | Other diagnosis (not depression/MDD) |
|  | Include this article |  |
| 252 , Duan, Y. P., Wienert, J., Hu, C., Si, G. Y., Lippke, S. Web-Based Intervention for Physical Activity and Fruit and Vegetable Intake Among Chinese University Students: A Randomized Controlled Trial. Journal of medical Internet research. 2017. 19:e106 , https://ovidsp.ovid.com/ovidweb.cgi?T=JS&CSC=Y&NEWS=N&PAGE=fulltext&D=emed18&AN=618381181 | Other diagnosis (not depression/MDD) |  |
| 253 , Chan, C., West, S., Glozier, N. Commencing and Persisting With a Web-Based Cognitive Behavioral Intervention for Insomnia: A Qualitative Study of Treatment Completers. Journal of medical Internet research. 2017. 19:e37 , https://ovidsp.ovid.com/ovidweb.cgi?T=JS&CSC=Y&NEWS=N&PAGE=fulltext&D=emed18&AN=617822362 |  | Other diagnosis (not depression/MDD) |
| 253 , Chan, C., West, S., Glozier, N. Commencing and Persisting With a Web-Based Cognitive Behavioral Intervention for Insomnia: A Qualitative Study of Treatment Completers. Journal of medical Internet research. 2017. 19:e37 , https://ovidsp.ovid.com/ovidweb.cgi?T=JS&CSC=Y&NEWS=N&PAGE=fulltext&D=emed18&AN=617822362 | Include this article |  |
| 254 , Rogers, M. A., Lemmen, K., Kramer, R., Mann, J., Chopra, V. Internet-Delivered Health Interventions That Work: Systematic Review of Meta-Analyses and Evaluation of Website Availability. Journal of medical Internet research. 2017. 19:e90 , https://ovidsp.ovid.com/ovidweb.cgi?T=JS&CSC=Y&NEWS=N&PAGE=fulltext&D=emed18&AN=617834132 | Inappropriate study design |  |
| 255 , McLean, G., Band, R., Saunderson, K., Hanlon, P., Murray, E., Little, P., McManus, R. J., Yardley, L., Mair, F. S. Digital interventions to promote self-management in adults with hypertension systematic review and meta-analysis. Journal of Hypertension. 2016. 34:600-612 , http://journals.lww.com/jhypertension https://ovidsp.ovid.com/ovidweb.cgi?T=JS&CSC=Y&NEWS=N&PAGE=fulltext&D=emed17&AN=608230147 | Other diagnosis (not depression/MDD) |  |
| 256 , Sharifi, V., Sedighnia, A., Ataie, S., Tabatabaee, M., Tehranidoost, M. The effect of a computer-assisted cognitive remediation on improving cognitive functions in patients with schizophrenia: A before-after study. Iranian Journal of Psychiatry and Behavioral Sciences. 2016. 10:http://cdn.neoscriber.org/cdn/dl/957fe06a-e707-11e6-9e28-f3535918be70 https://ovidsp.ovid.com/ovidweb.cgi?T=JS&CSC=Y&NEWS=N&PAGE=fulltext&D=emed17&AN=614416462 | Other diagnosis (not depression/MDD) |  |
| 257 , Bijker, L., Kleiboer, A., Riper, H., Cuijpers, P., Donker, T. E-care 4 caregivers - an online intervention for nonprofessional caregivers of patients with depression: Study protocol for a pilot randomized controlled trial. Trials. 2016. 17:http://www.trialsjournal.com/home/ https://ovidsp.ovid.com/ovidweb.cgi?T=JS&CSC=Y&NEWS=N&PAGE=fulltext&D=emed17&AN=609914290 | Other diagnosis (not depression/MDD) |  |
| 259 , Moritz, S., Schroder, J., Klein, J. P., Lincoln, T. M., Andreou, C., Fischer, A., Arlt, S. Effects of online intervention for depression on mood and positive symptoms in schizophrenia. Schizophrenia Research. 2016. 175:216-222 , http://www.elsevier.com/locate/schres https://ovidsp.ovid.com/ovidweb.cgi?T=JS&CSC=Y&NEWS=N&PAGE=fulltext&D=emed17&AN=610446009 | Other diagnosis (not depression/MDD) |  |
| 260 , Lee, J. A., Evangelista, L. S., Moore, A. A., Juth, V., Guo, Y., Gago-Masague, S., Lem, C. G., Nguyen, M., Khatibi, P., Baje, M., Amin, A. N. Feasibility Study of a Mobile Health Intervention for Older Adults on Oral Anticoagulation Therapy. Gerontology and Geriatric Medicine. 2016. 2:http://www.sagepub.com/journals/Journal202312?productType=Journals&status=New&sortBy=sortTitle%20asc&fs=1 https://ovidsp.ovid.com/ovidweb.cgi?T=JS&CSC=Y&NEWS=N&PAGE=fulltext&D=emed17&AN=624172184 | Other diagnosis (not depression/MDD) |  |
| 262 , Becker, J., Beutel, M. E., Gerzymisch, K., Schulz, D., Siepmann, M., Knickenberg, R. J., Schmadeke, S., Ferdinand, P., Zwerenz, R. Evaluation of a video-based Internet intervention as preparation for inpatient psychosomatic rehabilitation: Study protocol for a randomized controlled trial. Trials. 2016. 17:http://www.trialsjournal.com/home/ https://ovidsp.ovid.com/ovidweb.cgi?T=JS&CSC=Y&NEWS=N&PAGE=fulltext&D=emed17&AN=610770226 | Other diagnosis (not depression/MDD) |  |
| 263 , Ebert, D. D., Lehr, D., Heber, E., Riper, H., Cuijpers, P., Berking, M. Internet- and mobile-based stress management for employees with adherence-focused guidance: Efficacy and mechanism of change. Scandinavian Journal of Work, Environment and Health. 2016. 42:382-394 , http://www.sjweh.fi/download.php?abstract_id=3573&file_nro=1 https://ovidsp.ovid.com/ovidweb.cgi?T=JS&CSC=Y&NEWS=N&PAGE=fulltext&D=emed17&AN=612156051 | Other diagnosis (not depression/MDD) |  |
| 264 , Kayyali, R., Savickas, V., Spruit, M. A., Kaimakamis, E., Siva, R., Costello, R. W., Chang, J., Pierscionek, B., Davies, N., Vaes, A. W., Paradiso, R., Philip, N., Perantoni, E., D'Arcy, S., Raptopoulos, A., Nabhani-Gebara, S. Qualitative investigation into a wearable system for chronic obstructive pulmonary disease: The stakeholders' perspective. BMJ Open. 2016. 6:http://bmjopen.bmj.com/content/early/by/section https://ovidsp.ovid.com/ovidweb.cgi?T=JS&CSC=Y&NEWS=N&PAGE=fulltext&D=emed17&AN=612046635 | Other diagnosis (not depression/MDD) |  |
| 265 , Littleton, H., Grills, A. E., Kline, K. D., Schoemann, A. M., Dodd, J. C. The From Survivor to Thriver program: RCT of an online therapist-facilitated program for rape-related PTSD. Journal of Anxiety Disorders. 2016. 43:41-51 , http://www.elsevier.com/inca/publications/store/8/0/1 https://ovidsp.ovid.com/ovidweb.cgi?T=JS&CSC=Y&NEWS=N&PAGE=fulltext&D=emed17&AN=611572663 | Other diagnosis (not depression/MDD) |  |
| 266 , Schulz, A., Stolz, T., Vincent, A., Krieger, T., Andersson, G., Berger, T. A sorrow shared is a sorrow halved? A three-arm randomized controlled trial comparing internet-based clinician-guided individual versus group treatment for social anxiety disorder. Behaviour Research and Therapy. 2016. 84:14-26 , http://www.elsevier.com/locate/brat https://ovidsp.ovid.com/ovidweb.cgi?T=JS&CSC=Y&NEWS=N&PAGE=fulltext&D=emed17&AN=611220317 | Other diagnosis (not depression/MDD) |  |
| 267 , Grossert, A., Urech, C., Alder, J., Gaab, J., Berger, T., Hess, V. Web-based stress management for newly diagnosed cancer patients (STREAM-1): A randomized, wait-list controlled intervention study. BMC Cancer. 2016. 16:http://www.biomedcentral.com/bmccancer/ https://ovidsp.ovid.com/ovidweb.cgi?T=JS&CSC=Y&NEWS=N&PAGE=fulltext&D=emed17&AN=614038568 | Other diagnosis (not depression/MDD) |  |
| 268 , Christensen, H., Batterham, P. J., Gosling, J. A., Ritterband, L. M., Griffiths, K. M., Thorndike, F. P., Glozier, N., O'Dea, B., Hickie, I. B., Mackinnon, A. J. Effectiveness of an online insomnia program (SHUTi) for prevention of depressive episodes (the GoodNight Study): A randomised controlled trial. The Lancet Psychiatry. 2016. 3:333-341 , https://ovidsp.ovid.com/ovidweb.cgi?T=JS&CSC=Y&NEWS=N&PAGE=fulltext&D=emed17&AN=609467117 | Other diagnosis (not depression/MDD) |  |
| 269 , Beatty, L., Koczwara, B., Wade, T. Evaluating the efficacy of a self-guided Web-based CBT intervention for reducing cancer-distress: a randomised controlled trial. Supportive Care in Cancer. 2016. 24:1043-1051 , http://link.springer.de/link/service/journals/00520/index.htm https://ovidsp.ovid.com/ovidweb.cgi?T=JS&CSC=Y&NEWS=N&PAGE=fulltext&D=emed17&AN=605542950 | Other diagnosis (not depression/MDD) |  |
| 270 , Dyrbye, L. N., West, C. P., Richards, M. L., Ross, H. J., Satele, D., Shanafelt, T. D. A randomized, controlled study of an online intervention to promote job satisfaction and well-being among physicians. Burnout Research. 2016. 3:69-75 , http://www.journals.elsevier.com/burnout-research/ https://ovidsp.ovid.com/ovidweb.cgi?T=JS&CSC=Y&NEWS=N&PAGE=fulltext&D=emed17&AN=611209672 | Other diagnosis (not depression/MDD) |  |
| 272 , Moritz, S., Stepulovs, O., Schroder, J., Hottenrott, B., Meyer, B., Hauschildt, M. Is the whole less than the sum of its parts? Full versus individually adapted metacognitive self-help for obsessive-compulsive disorder: A randomized controlled trial. Journal of Obsessive-Compulsive and Related Disorders. 2016. 9:107-115 , http://www.journals.elsevier.com/journal-of-obsessive-compulsive-and-related-disorders https://ovidsp.ovid.com/ovidweb.cgi?T=JS&CSC=Y&NEWS=N&PAGE=fulltext&D=emed17&AN=609863440 | Other diagnosis (not depression/MDD) |  |
| 273 , Adamski, N., Adler, M., Opwis, K., Penner, I. K. A pilot study on the benefit of cognitive rehabilitation in Parkinson's disease. Therapeutic Advances in Neurological Disorders. 2016. 9:153-164 , http://tan.sagepub.com/ https://ovidsp.ovid.com/ovidweb.cgi?T=JS&CSC=Y&NEWS=N&PAGE=fulltext&D=emed17&AN=609352604 | Other diagnosis (not depression/MDD) |  |
| 274 , Oromendia, P., Orrego, J., Bonillo, A., Molinuevo, B. Internet-based self-help treatment for panic disorder: a randomized controlled trial comparing mandatory versus optional complementary psychological support. Cognitive behaviour therapy. 2016. 45:270-286 , https://ovidsp.ovid.com/ovidweb.cgi?T=JS&CSC=Y&NEWS=N&PAGE=fulltext&D=emed17&AN=620669947 | Other diagnosis (not depression/MDD) |  |
| 275 , Spoelstra, S. L., Given, C. W., Sikorskii, A., Coursaris, C. K., Majumder, A., DeKoekkoek, T., Schueller, M., Given, B. A. Proof of Concept of a Mobile Health Short Message Service Text Message Intervention That Promotes Adherence to Oral Anticancer Agent Medications: A Randomized Controlled Trial. Telemedicine journal and e-health : the official journal of the American Telemedicine Association. 2016. 22:497-506 , https://ovidsp.ovid.com/ovidweb.cgi?T=JS&CSC=Y&NEWS=N&PAGE=fulltext&D=emed17&AN=619697211 | Other diagnosis (not depression/MDD) |  |
| 276 , Bunge, E. L., Williamson, R. E., Cano, M., Leykin, Y., Munoz, R. F. Mood management effects of brief unsupported internet interventions. Internet Interventions. 2016. 5:36-43 , http://www.journals.elsevier.com/internet-interventions/ https://ovidsp.ovid.com/ovidweb.cgi?T=JS&CSC=Y&NEWS=N&PAGE=fulltext&D=emed17&AN=611514856 | Inappropriate study design |  |
| 277 , Kanera, I. M., Willems, R. A., Bolman, C. A., Mesters, I., Zambon, V., Gijsen, B. C., Lechner, L. Use and Appreciation of a Tailored Self-Management eHealth Intervention for Early Cancer Survivors: Process Evaluation of a Randomized Controlled Trial. Journal of medical Internet research. 2016. 18:e229 , https://ovidsp.ovid.com/ovidweb.cgi?T=JS&CSC=Y&NEWS=N&PAGE=fulltext&D=emed17&AN=618360949 | Other diagnosis (not depression/MDD) |  |
| 279 , Wang, Z., Wang, J., Maercker, A. Program Use and Outcome Change in a Web-Based Trauma Intervention: Individual and Social Factors. Journal of medical Internet research. 2016. 18:e243 , https://ovidsp.ovid.com/ovidweb.cgi?T=JS&CSC=Y&NEWS=N&PAGE=fulltext&D=emed17&AN=617823466 | Other diagnosis (not depression/MDD) |  |
| 280 , Milgrom, J., Danaher, B. G., Gemmill, A. W., Holt, C., Holt, C. J., Seeley, J. R., Tyler, M. S., Ross, J., Ericksen, J. Internet Cognitive Behavioral Therapy for Women With Postnatal Depression: A Randomized Controlled Trial of MumMoodBooster. Journal of medical Internet research. 2016. 18:e54 , https://ovidsp.ovid.com/ovidweb.cgi?T=JS&CSC=Y&NEWS=N&PAGE=fulltext&D=emed17&AN=616125879 | Other diagnosis (not depression/MDD) |  |
| 281 , Millan-Calenti, J. C., Lorenzo, T., Nunez-Naveira, L., Bujan, A., Rodriguez-Villamil, J. L., Maseda, A. Efficacy of a computerized cognitive training application on cognition and depressive symptomatology in a group of healthy older adults: A randomized controlled trial. Archives of Gerontology and Geriatrics. 2015. 61:337-343 , http://www.elsevier.com/locate/archger https://ovidsp.ovid.com/ovidweb.cgi?T=JS&CSC=Y&NEWS=N&PAGE=fulltext&D=emed16&AN=605789166 | Other diagnosis (not depression/MDD) |  |
| 286 , O'Dea, B., Calear, A. L., Perry, Y. Is e-health the answer to gaps in adolescent mental health service provision?. Current Opinion in Psychiatry. 2015. 28:336-342 , http://journals.lww.com/co-psychiatry/pages/default.aspx https://ovidsp.ovid.com/ovidweb.cgi?T=JS&CSC=Y&NEWS=N&PAGE=fulltext&D=emed16&AN=606206284 | Inappropriate study design |  |
| 287 , Knowles, S. E., Lovell, K., Bower, P., Gilbody, S., Littlewood, E., Lester, H. Patient experience of computerised therapy for depression in primary care. BMJ Open. 2015. 5:http://bmjopen.bmj.com/content/5/11/e008581.full.pdf+html https://ovidsp.ovid.com/ovidweb.cgi?T=JS&CSC=Y&NEWS=N&PAGE=fulltext&D=emed16&AN=608042417 | Include this article |  |
| 287 , Knowles, S. E., Lovell, K., Bower, P., Gilbody, S., Littlewood, E., Lester, H. Patient experience of computerised therapy for depression in primary care. BMJ Open. 2015. 5:http://bmjopen.bmj.com/content/5/11/e008581.full.pdf+html https://ovidsp.ovid.com/ovidweb.cgi?T=JS&CSC=Y&NEWS=N&PAGE=fulltext&D=emed16&AN=608042417 |  | Inappropriate study design |
| 289 , Law, E. F., Beals-Erickson, S. E., Noel, M., Claar, R., Palermo, T. M. Pilot randomized controlled trial of internet-delivered cognitive-behavioral treatment for pediatric headache. Headache. 2015. 55:1410-1425 , http://www.wiley.com/bw/editors.asp?ref=0017-8748&site=1 https://ovidsp.ovid.com/ovidweb.cgi?T=JS&CSC=Y&NEWS=N&PAGE=fulltext&D=emed16&AN=605842173 | Other diagnosis (not depression/MDD) |  |
| 290 , Vallejo, M. A., Ortega, J., Rivera, J., Comeche, M. I., Vallejo-Slocker, L. Internet versus face-to-face group cognitive-behavioral therapy for fibromyalgia: A randomized control trial. Journal of Psychiatric Research. 2015. 68:106-113 , http://www.elsevier.com/locate/jpsychires https://ovidsp.ovid.com/ovidweb.cgi?T=JS&CSC=Y&NEWS=N&PAGE=fulltext&D=emed16&AN=605393342 | Other diagnosis (not depression/MDD) |  |
| 291 , Pressler, S. J., Titler, M., Koelling, T. M., Riley, P. L., Jung, M., Hoyland-Domenico, L., Ronis, D. L., Smith, D. G., Bleske, B. E., Dorsey, S. G., Giordani, B. Nurse-Enhanced Computerized Cognitive Training Increases Serum Brain-Derived Neurotropic Factor Levels and Improves Working Memory in Heart Failure. Journal of Cardiac Failure. 2015. 21:630-641 , http://www.elsevier.com/inca/publications/store/6/2/3/3/0/6/index.htt https://ovidsp.ovid.com/ovidweb.cgi?T=JS&CSC=Y&NEWS=N&PAGE=fulltext&D=emed16&AN=604952892 | Other diagnosis (not depression/MDD) |  |
| 292 , Pereira, C. A., Wen, C. L., Miguel, E. C., Polanczyk, G. V. A randomised controlled trial of a web-based educational program in child mental health for schoolteachers. European Child and Adolescent Psychiatry. 2015. 24:931-940 , http://www.springerlink.com/content/1018-8827 https://ovidsp.ovid.com/ovidweb.cgi?T=JS&CSC=Y&NEWS=N&PAGE=fulltext&D=emed16&AN=600645573 | Other diagnosis (not depression/MDD) |  |
| 293 , Imamura, K., Kawakami, N., Furukawa, T. A., Matsuyama, Y., Shimazu, A., Umanodan, R., Kawakami, S., Kasai, K. Effects of an internet-based cognitive behavioral therapy intervention on improving work engagement and other work-related outcomes: An analysis of secondary outcomes of a randomized controlled trial. Journal of Occupational and Environmental Medicine. 2015. 57:578-584 , http://journals.lww.com/joem https://ovidsp.ovid.com/ovidweb.cgi?T=JS&CSC=Y&NEWS=N&PAGE=fulltext&D=emed16&AN=606212646 | Other diagnosis (not depression/MDD) |  |
| 295 , King, C. A., Eisenberg, D., Zheng, K., Czyz, E., Kramer, A., Horwitz, A., Chermack, S. Online suicide risk screening and intervention with college students: A pilot randomized controlled trial. Journal of Consulting and Clinical Psychology. 2015. 83:630-636 , http://www.apa.org/pubs/journals/ccp/index.aspx https://ovidsp.ovid.com/ovidweb.cgi?T=JS&CSC=Y&NEWS=N&PAGE=fulltext&D=emed16&AN=602331258 | Include this article |  |
|  |  | Inappropriate intervention |
| 296 , Ruggiero, K. J., Price, M., Adams, Z., Stauffacher, K., McCauley, J., Danielson, C. K., Knapp, R., Hanson, R. F., Davidson, T. M., Amstadter, A. B., Carpenter, M. J., Saunders, B. E., Kilpatrick, D. G., Resnick, H. S. Web Intervention for Adolescents Affected by Disaster: Population-Based Randomized Controlled Trial. Journal of the American Academy of Child and Adolescent Psychiatry. 2015. 54:709-717 , http://www.jaacap.com https://ovidsp.ovid.com/ovidweb.cgi?T=JS&CSC=Y&NEWS=N&PAGE=fulltext&D=emed16&AN=605710613 | Other diagnosis (not depression/MDD) |  |
| 297 , Hickman, N. J., Delucchi, K. L., Prochaska, J. J. Treating tobacco dependence at the intersection of diversity, poverty, and mental illness: A randomized feasibility and replication trial. Nicotine and Tobacco Research. 2015. 17:1012-1021 , http://ntr.oxfordjournals.org/ https://ovidsp.ovid.com/ovidweb.cgi?T=JS&CSC=Y&NEWS=N&PAGE=fulltext&D=emed16&AN=605708776 | Other diagnosis (not depression/MDD) |  |
| 298 , Fergus, K., Ahmad, S., McLeod, D. L., Stephen, J., Gardner, S., Pereira, A., Warner, E., Carter, W. Couplelinks - an online intervention for young women with breast cancer and their male partners: Study protocol for a randomized controlled trial. Trials. 2015. 16:http://www.trialsjournal.com/home/ https://ovidsp.ovid.com/ovidweb.cgi?T=JS&CSC=Y&NEWS=N&PAGE=fulltext&D=emed16&AN=604122888 | Other diagnosis (not depression/MDD) |  |
| 299 , Priebe, S., Kelley, L., Omer, S., Golden, E., Walsh, S., Khanom, H., Kingdon, D., Rutterford, C., McCrone, P., McCabe, R. The Effectiveness of a Patient-Centred Assessment with a Solution-Focused Approach (DIALOG+) for Patients with Psychosis: A Pragmatic Cluster-Randomised Controlled Trial in Community Care. Psychotherapy and Psychosomatics. 2015. 84:304-313 , http://www.karger.com/journals/pps/pps_jh.htm https://ovidsp.ovid.com/ovidweb.cgi?T=JS&CSC=Y&NEWS=N&PAGE=fulltext&D=emed16&AN=605694759 | Other diagnosis (not depression/MDD) |  |
| 300 , Imamura, K., Kawakami, N., Furukawa, T. A., Matsuyama, Y., Shimazu, A., Kasai, K. Effects of an internet-based cognitive behavioural therapy intervention on preventing major depressive episodes among workers: A protocol for a randomised controlled trial. BMJ Open. 2015. 5:http://bmjopen.bmj.com/content/5/5/e007590.full.pdf+html https://ovidsp.ovid.com/ovidweb.cgi?T=JS&CSC=Y&NEWS=N&PAGE=fulltext&D=emed16&AN=604465196 | Other diagnosis (not depression/MDD) |  |
| 301 , Depp, C. A., Ceglowski, J., Wang, V. C., Yaghouti, F., Mausbach, B. T., Thompson, W. K., Granholm, E. L. Augmenting psychoeducation with a mobile intervention for bipolar disorder: A randomized controlled trial. Journal of Affective Disorders. 2015. 174:23-30 , http://www.elsevier.com/locate/jad https://ovidsp.ovid.com/ovidweb.cgi?T=JS&CSC=Y&NEWS=N&PAGE=fulltext&D=emed16&AN=601026270 | Other diagnosis (not depression/MDD) |  |
| 302 , Ruggiero, K. J., Davidson, T. M., McCauley, J., Gros, K. S., Welsh, K., Price, M., Resnick, H. S., Danielson, C. K., Soltis, K., Galea, S., Kilpatrick, D. G., Saunders, B. E., Nissenboim, J., Muzzy, W., Fleeman, A., Amstadter, A. B. Bounce back now! protocol of a population-based randomized controlled trial to examine the efficacy of a web-based intervention with disaster-affected families. Contemporary Clinical Trials. 2015. 40:138-149 , http://www.elsevier.com/wps/find/journaldescription.cws_home/704636/description#description https://ovidsp.ovid.com/ovidweb.cgi?T=JS&CSC=Y&NEWS=N&PAGE=fulltext&D=emed16&AN=600790779 | Other diagnosis (not depression/MDD) |  |
| 303 , Saekow, J., Jones, M., Gibbs, E., Jacobi, C., Fitzsimmons-Craft, E. E., Wilfley, D., Barr Taylor, C. StudentBodies-eating disorders: A randomized controlled trial of a coached online intervention for subclinical eating disorders. Internet Interventions. 2015. 2:419-428 , http://www.journals.elsevier.com/internet-interventions/ https://ovidsp.ovid.com/ovidweb.cgi?T=JS&CSC=Y&NEWS=N&PAGE=fulltext&D=emed16&AN=606782479 | Other diagnosis (not depression/MDD) |  |
| 305 , Barrera, A. Z., Wickham, R. E., Munoz, R. F. Online prevention of postpartum depression for Spanish- and English-speaking pregnant women: A pilot randomized controlled trial. Internet Interventions. 2015. 2:257-265 , http://www.journals.elsevier.com/internet-interventions/ https://ovidsp.ovid.com/ovidweb.cgi?T=JS&CSC=Y&NEWS=N&PAGE=fulltext&D=emed16&AN=604984697 | Other diagnosis (not depression/MDD) |  |
| 306 , Dear, B. F., Zou, J. B., Ali, S., Lorian, C. N., Johnston, L., Terides, M. D., Staples, L. G., Gandy, M., Fogliati, V. J., Klein, B., Titov, N. Examining self-guided internet-delivered cognitive behavior therapy for older adults with symptoms of anxiety and depression: Two feasibility open trials. Internet Interventions. 2015. 2:17-23 , http://www.journals.elsevier.com/internet-interventions/ https://ovidsp.ovid.com/ovidweb.cgi?T=JS&CSC=Y&NEWS=N&PAGE=fulltext&D=emed16&AN=601048599 | Include this article |  |
| 306 , Dear, B. F., Zou, J. B., Ali, S., Lorian, C. N., Johnston, L., Terides, M. D., Staples, L. G., Gandy, M., Fogliati, V. J., Klein, B., Titov, N. Examining self-guided internet-delivered cognitive behavior therapy for older adults with symptoms of anxiety and depression: Two feasibility open trials. Internet Interventions. 2015. 2:17-23 , http://www.journals.elsevier.com/internet-interventions/ https://ovidsp.ovid.com/ovidweb.cgi?T=JS&CSC=Y&NEWS=N&PAGE=fulltext&D=emed16&AN=601048599 |  | Other diagnosis (not depression/MDD) |
| 307 , Cook, F., Seymour, M., Giallo, R., Cann, W., Nicholson, J. M., Green, J., Hiscock, H. Comparison of methods for recruiting and engaging parents in online interventions: Study protocol for the Cry Baby infant sleep and settling program. BMC Pediatrics. 2015. 15:http://www.biomedcentral.com/bmcpediatr/ https://ovidsp.ovid.com/ovidweb.cgi?T=JS&CSC=Y&NEWS=N&PAGE=fulltext&D=emed16&AN=606816522 | Other diagnosis (not depression/MDD) |  |
| 309 , Piette, J. D., Striplin, D., Marinec, N., Chen, J., Trivedi, R. B., Aron, D. C., Fisher, L., Aikens, J. E. A Mobile Health Intervention Supporting Heart Failure Patients and Their Informal Caregivers: A Randomized Comparative Effectiveness Trial. Journal of medical Internet research. 2015. 17:e142 , https://ovidsp.ovid.com/ovidweb.cgi?T=JS&CSC=Y&NEWS=N&PAGE=fulltext&D=emed16&AN=615135003 | Other diagnosis (not depression/MDD) |  |
| 310 , Wilson, M., Roll, J. M., Corbett, C., Barbosa-Leiker, C. Empowering Patients with Persistent Pain Using an Internet-based Self-Management Program. Pain management nursing : official journal of the American Society of Pain Management Nurses. 2015. 16:503-514 , https://ovidsp.ovid.com/ovidweb.cgi?T=JS&CSC=Y&NEWS=N&PAGE=fulltext&D=emed16&AN=615133142 | Other diagnosis (not depression/MDD) |  |
| 311 , Jimenez-Serrano, S., Tortajada, S., Garcia-Gomez, J. M. A Mobile Health Application to Predict Postpartum Depression Based on Machine Learning. Telemedicine journal and e-health : the official journal of the American Telemedicine Association. 2015. 21:567-574 , https://ovidsp.ovid.com/ovidweb.cgi?T=JS&CSC=Y&NEWS=N&PAGE=fulltext&D=emed16&AN=615100493 | Other diagnosis (not depression/MDD) |  |
| 312 , Morris, R. R., Schueller, S. M., Picard, R. W. Efficacy of a Web-based, crowdsourced peer-to-peer cognitive reappraisal platform for depression: randomized controlled trial. Journal of medical Internet research. 2015. 17:e72 , https://ovidsp.ovid.com/ovidweb.cgi?T=JS&CSC=Y&NEWS=N&PAGE=fulltext&D=emed16&AN=613682606 | Other diagnosis (not depression/MDD) |  |
| 313 , Volker, D., Zijlstra-Vlasveld, M. C., Anema, J. R., Beekman, A. T., Brouwers, E. P., Emons, W. H., van Lomwel, A. G., van der Feltz-Cornelis, C. M. Effectiveness of a blended web-based intervention on return to work for sick-listed employees with common mental disorders: results of a cluster randomized controlled trial. Journal of medical Internet research. 2015. 17:e116 , https://ovidsp.ovid.com/ovidweb.cgi?T=JS&CSC=Y&NEWS=N&PAGE=fulltext&D=emed16&AN=609665483 | Other diagnosis (not depression/MDD) |  |
| 314 , Trompetter, H. R., Bohlmeijer, E. T., Veehof, M. M., Schreurs, K. M. Internet-based guided self-help intervention for chronic pain based on Acceptance and Commitment Therapy: a randomized controlled trial. Journal of behavioral medicine. 2015. 38:66-80 , https://ovidsp.ovid.com/ovidweb.cgi?T=JS&CSC=Y&NEWS=N&PAGE=fulltext&D=emed16&AN=606351555 | Other diagnosis (not depression/MDD) |  |
| 315 , Choi, I., Sharpe, L., Li, S., Hunt, C. Acceptability of psychological treatment to Chinese- and Caucasian-Australians: Internet treatment reduces barriers but face-to-face care is preferred. Social psychiatry and psychiatric epidemiology. 2015. 50:77-87 , https://ovidsp.ovid.com/ovidweb.cgi?T=JS&CSC=Y&NEWS=N&PAGE=fulltext&D=emed16&AN=606343926 | Include this article |  |
| 315 , Choi, I., Sharpe, L., Li, S., Hunt, C. Acceptability of psychological treatment to Chinese- and Caucasian-Australians: Internet treatment reduces barriers but face-to-face care is preferred. Social psychiatry and psychiatric epidemiology. 2015. 50:77-87 , https://ovidsp.ovid.com/ovidweb.cgi?T=JS&CSC=Y&NEWS=N&PAGE=fulltext&D=emed16&AN=606343926 |  | Inappropriate study design |
| 316 , Schneider, B. C., Wittekind, C. E., Talhof, A., Korrelboom, K., Moritz, S. Competitive Memory Training (COMET) for OCD: a self-treatment approach to obsessions. Cognitive behaviour therapy. 2015. 44:142-152 , https://ovidsp.ovid.com/ovidweb.cgi?T=JS&CSC=Y&NEWS=N&PAGE=fulltext&D=emed16&AN=606689746 | Other diagnosis (not depression/MDD) |  |
| 318 , Carbon, M., Correll, C. U. Thinking and acting beyond the positive: The role of the cognitive and negative symptoms in schizophrenia. CNS Spectrums. 2014. 19:38-52 , http://journals.cambridge.org/action/displayJournal?jid=CNS https://ovidsp.ovid.com/ovidweb.cgi?T=JS&CSC=Y&NEWS=N&PAGE=fulltext&D=emed15&AN=621638925 | Other diagnosis (not depression/MDD) |  |
| 319 , Sergeant, S., Mongrain, M. An online optimism intervention reduces depression in pessimistic individuals. Journal of Consulting and Clinical Psychology. 2014. 82:263-274 , http://www.apa.org/pubs/journals/ccp/index.aspx https://ovidsp.ovid.com/ovidweb.cgi?T=JS&CSC=Y&NEWS=N&PAGE=fulltext&D=emed15&AN=53002588 | Other diagnosis (not depression/MDD) |  |
| 320 , Heller, H. M., van Straten, A., de Groot, C. J. M., Honig, A. The (cost) effectiveness of an online intervention for pregnant women with affective symptoms: Protocol of a randomised controlled trial. BMC Pregnancy and Childbirth. 2014. 14:1-7 , http://www.biomedcentral.com/1471-2393/14/273 https://ovidsp.ovid.com/ovidweb.cgi?T=JS&CSC=Y&NEWS=N&PAGE=fulltext&D=emed15&AN=600230171 | Other diagnosis (not depression/MDD) |  |
| 321 , Drozd, F., Skeie, L. G., Kraft, P., Kvale, D. A web-based intervention trial for depressive symptoms and subjective well-being in patients with chronic HIV infection. AIDS Care - Psychological and Socio-Medical Aspects of AIDS/HIV. 2014. 26:1080-1089 , https://ovidsp.ovid.com/ovidweb.cgi?T=JS&CSC=Y&NEWS=N&PAGE=fulltext&D=emed15&AN=52934580 | Other diagnosis (not depression/MDD) |  |
| 322 , Crisp, D., Griffiths, K., Mackinnon, A., Bennett, K., Christensen, H. An online intervention for reducing depressive symptoms: Secondary benefits for self-esteem, empowerment and quality of life. Psychiatry Research. 2014. 216:60-66 , http://www.elsevier.com/locate/psychres https://ovidsp.ovid.com/ovidweb.cgi?T=JS&CSC=Y&NEWS=N&PAGE=fulltext&D=emed15&AN=372569418 | Other diagnosis (not depression/MDD) |  |
| 323 , Bolier, L., Ketelaar, S. M., Nieuwenhuijsen, K., Smeets, O., Gartner, F. R., Sluiter, J. K. Workplace mental health promotion online to enhance well-being of nurses and allied health professionals: A cluster-randomized controlled trial. Internet Interventions. 2014. 1:196-204 , http://www.journals.elsevier.com/internet-interventions/ https://ovidsp.ovid.com/ovidweb.cgi?T=JS&CSC=Y&NEWS=N&PAGE=fulltext&D=emed15&AN=601068895 | Other diagnosis (not depression/MDD) |  |
| 324 , Lindner, P., Olsson, E. L., Johnsson, A., Dahlin, M., Andersson, G., Carlbring, P. The impact of telephone versus e-mail therapist guidance on treatment outcomes, therapeutic alliance and treatment engagement in Internet-delivered CBT for depression: A randomised pilot trial. Internet Interventions. 2014. 1:182-187 , http://www.journals.elsevier.com/internet-interventions/ https://ovidsp.ovid.com/ovidweb.cgi?T=JS&CSC=Y&NEWS=N&PAGE=fulltext&D=emed15&AN=601068891 | Inappropriate intervention |  |
| 325 , Christensen, H., Mackinnon, A. J., Batterham, P. J., O'Dea, B., Guastella, A. J., Griffiths, K. M., Eagleson, C., Kalia Hehir, K., Kenardy, J., Bennett, K., Hickie, I. The effectiveness of an online e-health application compared to attention placebo or Sertraline in the treatment of Generalised Anxiety Disorder. Internet Interventions. 2014. 1:169-174 , http://www.journals.elsevier.com/internet-interventions/ https://ovidsp.ovid.com/ovidweb.cgi?T=JS&CSC=Y&NEWS=N&PAGE=fulltext&D=emed15&AN=601068889 | Other diagnosis (not depression/MDD) |  |
| 326 , Buntrock, C., Ebert, D. D., Lehr, D., Cuijpers, P., Riper, H., Smit, F., Berking, M. Evaluating the efficacy and cost-effectiveness of web-based indicated prevention of major depression: Design of a randomised controlled trial. BMC Psychiatry. 2014. 14:http://www.biomedcentral.com/1471-244X/14/25 https://ovidsp.ovid.com/ovidweb.cgi?T=JS&CSC=Y&NEWS=N&PAGE=fulltext&D=emed15&AN=52990768 | Include this article |  |
| 326 , Buntrock, C., Ebert, D. D., Lehr, D., Cuijpers, P., Riper, H., Smit, F., Berking, M. Evaluating the efficacy and cost-effectiveness of web-based indicated prevention of major depression: Design of a randomised controlled trial. BMC Psychiatry. 2014. 14:http://www.biomedcentral.com/1471-244X/14/25 https://ovidsp.ovid.com/ovidweb.cgi?T=JS&CSC=Y&NEWS=N&PAGE=fulltext&D=emed15&AN=52990768 |  | Other diagnosis (not depression/MDD) |
| 329 , Andrews, G., Newby, J. M., Williams, A. D. Internet-Delivered Cognitive Behavior Therapy for Anxiety Disorders Is Here to Stay. Current Psychiatry Reports. 2014. 17:http://www.springerlink.com/content/1523-3812/ https://ovidsp.ovid.com/ovidweb.cgi?T=JS&CSC=Y&NEWS=N&PAGE=fulltext&D=emed15&AN=600563678 | Inappropriate study design |  |
| 330 , Pugh, N. E., Hadjistavropoulos, H. D., Klein, B., Austin, D. W. A Case Study Illustrating Therapist-Assisted Internet Cognitive Behavior Therapy for Depression. Cognitive and Behavioral Practice. 2014. 21:64-77 , https://ovidsp.ovid.com/ovidweb.cgi?T=JS&CSC=Y&NEWS=N&PAGE=fulltext&D=emed15&AN=372074641 | Inappropriate study design |  |
| 331 , Kok, G., Bockting, C., Burger, H., Smit, F., Riper, H. Mobile cognitive therapy: Adherence and acceptability of an online intervention in remitted recurrently depressed patients. Internet Interventions. 2014. 1:65-73 , http://www.journals.elsevier.com/internet-interventions/ https://ovidsp.ovid.com/ovidweb.cgi?T=JS&CSC=Y&NEWS=N&PAGE=fulltext&D=emed15&AN=373527640 | Include this article |  |
| 331 , Kok, G., Bockting, C., Burger, H., Smit, F., Riper, H. Mobile cognitive therapy: Adherence and acceptability of an online intervention in remitted recurrently depressed patients. Internet Interventions. 2014. 1:65-73 , http://www.journals.elsevier.com/internet-interventions/ https://ovidsp.ovid.com/ovidweb.cgi?T=JS&CSC=Y&NEWS=N&PAGE=fulltext&D=emed15&AN=373527640 |  | Other diagnosis (not depression/MDD) |
| 332 , Wright, B., Tindall, L., Littlewood, E., Adamson, J., Allgar, V., Bennett, S., Gilbody, S., Verduyn, C., Alderson-Day, B., Dyson, L., Trepel, D., Ali, S. Computerised cognitive behaviour therapy for depression in adolescents: Study protocol for a feasibility randomised controlled trial. BMJ Open. 2014. 4:http://bmjopen.bmj.com/content/4/10/e006488.full.pdf+html https://ovidsp.ovid.com/ovidweb.cgi?T=JS&CSC=Y&NEWS=N&PAGE=fulltext&D=emed15&AN=600505401 | Inappropriate study design |  |
| 333 , Jasper, K., Weise, C., Conrad, I., Andersson, G., Hiller, W., Kleinstauber, M. Internet-based guided self-help versus group cognitive behavioral therapy for chronic tinnitus: A randomized controlled trial. Psychotherapy and Psychosomatics. 2014. 83:234-246 , http://www.karger.com/journals/pps/pps_jh.htm https://ovidsp.ovid.com/ovidweb.cgi?T=JS&CSC=Y&NEWS=N&PAGE=fulltext&D=emed15&AN=53211683 | Other diagnosis (not depression/MDD) |  |
| 334 , Farrer, L. M., Griffiths, K. M., Christensen, H., Mackinnon, A. J., Batterham, P. J. Predictors of adherence and outcome in internet-based cognitive behavior therapy delivered in a telephone counseling setting. Cognitive Therapy and Research. 2014. 38:358-367 , http://www.kluweronline.com/issn/0147-5916/ https://ovidsp.ovid.com/ovidweb.cgi?T=JS&CSC=Y&NEWS=N&PAGE=fulltext&D=emed15&AN=52835713 | Other diagnosis (not depression/MDD) |  |
| 335 , Mahoney, A. E., Mackenzie, A., Williams, A. D., Smith, J., Andrews, G. Internet cognitive behavioural treatment for obsessive compulsive disorder: A randomised controlled trial. Behaviour research and therapy. 2014. 63:99-106 , https://ovidsp.ovid.com/ovidweb.cgi?T=JS&CSC=Y&NEWS=N&PAGE=fulltext&D=emed15&AN=620261335 | Other diagnosis (not depression/MDD) |  |
| 336 , Brough, C., Boyce, S., Houchen-Wolloff, L., Sewell, L., Singh, S. Evaluating the interactive web-based program, activate your heart, for cardiac rehabilitation patients: a pilot study. Journal of medical Internet research. 2014. 16:e242 , https://ovidsp.ovid.com/ovidweb.cgi?T=JS&CSC=Y&NEWS=N&PAGE=fulltext&D=emed15&AN=613661476 | Other diagnosis (not depression/MDD) |  |
| 338 , Gaffney, H., Mansell, W., Edwards, R., Wright, J. Manage Your Life Online (MYLO): a pilot trial of a conversational computer-based intervention for problem solving in a student sample. Behavioural and cognitive psychotherapy. 2014. 42:731-746 , https://ovidsp.ovid.com/ovidweb.cgi?T=JS&CSC=Y&NEWS=N&PAGE=fulltext&D=emed15&AN=605703948 | Other diagnosis (not depression/MDD) |  |
| 339 , Borosund, E., Cvancarova, M., Moore, S. M., Ekstedt, M., Ruland, C. M. Comparing effects in regular practice of e-communication and Web-based self-management support among breast cancer patients: preliminary results from a randomized controlled trial. Journal of medical Internet research. 2014. 16:e295 , https://ovidsp.ovid.com/ovidweb.cgi?T=JS&CSC=Y&NEWS=N&PAGE=fulltext&D=emed15&AN=609247235 | Other diagnosis (not depression/MDD) |  |
| 341 , Stasiak, K., Hatcher, S., Frampton, C., Merry, S. N. A pilot double blind randomized placebo controlled trial of a prototype computer-based cognitive behavioural therapy program for adolescents with symptoms of depression. Behavioural and cognitive psychotherapy. 2014. 42:385-401 , https://ovidsp.ovid.com/ovidweb.cgi?T=JS&CSC=Y&NEWS=N&PAGE=fulltext&D=emed15&AN=609192061 | Inappropriate study design |  |
| 342 , Antypas, K., Wangberg, S. C. An Internet- and mobile-based tailored intervention to enhance maintenance of physical activity after cardiac rehabilitation: short-term results of a randomized controlled trial. Journal of medical Internet research. 2014. 16:e77 , https://ovidsp.ovid.com/ovidweb.cgi?T=JS&CSC=Y&NEWS=N&PAGE=fulltext&D=emed15&AN=604316520 | Other diagnosis (not depression/MDD) |  |
| 344 , Bantum, E. O., Albright, C. L., White, K. K., Berenberg, J. L., Layi, G., Ritter, P. L., Laurent, D., Plant, K., Lorig, K. Surviving and thriving with cancer using a Web-based health behavior change intervention: randomized controlled trial. Journal of medical Internet research. 2014. 16:e54 , https://ovidsp.ovid.com/ovidweb.cgi?T=JS&CSC=Y&NEWS=N&PAGE=fulltext&D=emed15&AN=373968906 | Other diagnosis (not depression/MDD) |  |
| 345 , Mananes, G., Vallejo, M. A. Usage and effectiveness of a fully automated, open-access, Spanish Web-based smoking cessation program: randomized controlled trial. Journal of medical Internet research. 2014. 16:e111 , https://ovidsp.ovid.com/ovidweb.cgi?T=JS&CSC=Y&NEWS=N&PAGE=fulltext&D=emed15&AN=373973730 | Other diagnosis (not depression/MDD) |  |
| 346 , Akerlund, E., Esbjornsson, E., Sunnerhagen, K. S., Bjorkdahl, A. Can computerized working memory training improve impaired working memory, cognition and psychological health?. Brain Injury. 2013. 27:1649-1657 , https://ovidsp.ovid.com/ovidweb.cgi?T=JS&CSC=Y&NEWS=N&PAGE=fulltext&D=emed14&AN=370382616 | Other diagnosis (not depression/MDD) |  |
| 347 , Lancee, J., van den Bout, J., Sorbi, M. J., van Straten, A. Motivational support provided via email improves the effectiveness of internet-delivered self-help treatment for insomnia: A randomized trial. Behaviour Research and Therapy. 2013. 51:797-805 , http://www.elsevier.com/locate/brat https://ovidsp.ovid.com/ovidweb.cgi?T=JS&CSC=Y&NEWS=N&PAGE=fulltext&D=emed14&AN=369999157 | Other diagnosis (not depression/MDD) |  |
| 348 , Cavanagh, K., Strauss, C., Cicconi, F., Griffiths, N., Wyper, A., Jones, F. A randomised controlled trial of a brief online mindfulness-based intervention. Behaviour Research and Therapy. 2013. 51:573-578 , http://www.elsevier.com/locate/brat https://ovidsp.ovid.com/ovidweb.cgi?T=JS&CSC=Y&NEWS=N&PAGE=fulltext&D=emed14&AN=369404325 | Other diagnosis (not depression/MDD) |  |
| 349 , Buhrman, M., Skoglund, A., Husell, J., Bergstrom, K., Gordh, T., Hursti, T., Bendelin, N., Furmark, T., Andersson, G. Guided internet-delivered acceptance and commitment therapy for chronic pain patients: A randomized controlled trial. Behaviour Research and Therapy. 2013. 51:307-315 , http://www.elsevier.com/locate/brat https://ovidsp.ovid.com/ovidweb.cgi?T=JS&CSC=Y&NEWS=N&PAGE=fulltext&D=emed14&AN=368670379 | Other diagnosis (not depression/MDD) |  |
| 350 , Reins, J. A., Ebert, D. D., Lehr, D., Riper, H., Cuijpers, P., Berking, M. Internet-based treatment of major depression for patients on a waiting list for inpatient psychotherapy: Protocol for a multi-centre randomised controlled trial. BMC Psychiatry. 2013. 13 (no pagination):http://www.biomedcentral.com/1471-244X/13/318 https://ovidsp.ovid.com/ovidweb.cgi?T=JS&CSC=Y&NEWS=N&PAGE=fulltext&D=emed14&AN=52890936 |  | Inappropriate outcomes |
|  | Include this article |  |
| 351 , Brown, L. A., Craske, M. G., Glenn, D. E., Stein, M. B., Sullivan, G., Sherbourne, C., Bystritsky, A., Welch, S. S., Campbell-Sills, L., Lang, A., Roy-Byrne, P., Rose, R. D. CBT competence in novice therapists improves anxiety outcomes. Depression and Anxiety. 2013. 30:97-115 , https://ovidsp.ovid.com/ovidweb.cgi?T=JS&CSC=Y&NEWS=N&PAGE=fulltext&D=emed14&AN=52342435 | Other diagnosis (not depression/MDD) |  |
| 352 , Meiser, B., Schofield, P. R., Trevena, L., Wilde, A., Barlow-Stewart, K., Proudfoot, J., Peate, M., Dobbins, T., Christensen, H., Sherman, K. A., Karatas, J., Mitchell, P. B. Cluster randomized controlled trial of a psycho-educational intervention for people with a family history of depression for use in general practice. BMC Psychiatry. 2013. 13 (no pagination):http://www.biomedcentral.com/1471-244X/13/325 https://ovidsp.ovid.com/ovidweb.cgi?T=JS&CSC=Y&NEWS=N&PAGE=fulltext&D=emed14&AN=52896729 | Other diagnosis (not depression/MDD) |  |
| 354 , Montero-Marin, J., Carrasco, J. M., Roca, M., Serrano-Blanco, A., Gili, M., Mayoral, F., Luciano, J. V., Lopez-del-Hoyo, Y., Olivan, B., Collazo, F., Araya, R., Banos, R., Botella, C., Garcia-Campayo, J. Expectations, experiences and attitudes of patients and primary care health professionals regarding online psychotherapeutic interventions for depression: Protocol for a qualitative study. BMC Psychiatry. 2013. 13 (no pagination):http://www.biomedcentral.com/1471-244X/13/64 https://ovidsp.ovid.com/ovidweb.cgi?T=JS&CSC=Y&NEWS=N&PAGE=fulltext&D=emed14&AN=52456228 | Inappropriate study design |  |
| 355 , Lee, G. Y., Yip, C. C. K., Yu, E. C. S., Man, D. W. K. Evaluation of a computer-assisted errorless learning-based memory training program for patients with early Alzheimer's disease in Hong Kong: A pilot study. Clinical Interventions in Aging. 2013. 8:623-633 , http://www.dovepress.com/getfile.php?fileID=16324 https://ovidsp.ovid.com/ovidweb.cgi?T=JS&CSC=Y&NEWS=N&PAGE=fulltext&D=emed14&AN=369093736 | Other diagnosis (not depression/MDD) |  |
| 356 , Leff, J., Williams, G., Huckvale, M. A., Arbuthnot, M., Leff, A. P. Computer-assisted therapy for medication-resistant auditory hallucinations: Proof-of-concept study. British Journal of Psychiatry. 2013. 202:428-433 , http://bjp.rcpsych.org/content/202/6/428.full.pdf+html https://ovidsp.ovid.com/ovidweb.cgi?T=JS&CSC=Y&NEWS=N&PAGE=fulltext&D=emed14&AN=369073935 | Other diagnosis (not depression/MDD) |  |
| 358 , Heber, E., Ebert, D. D., Lehr, D., Nobis, S., Berking, M., Riper, H. Efficacy and cost-effectiveness of a web-based and mobile stress-management intervention for employees: design of a randomized controlled trial. BMC public health. 2013. 13:655 , https://ovidsp.ovid.com/ovidweb.cgi?T=JS&CSC=Y&NEWS=N&PAGE=fulltext&D=emed14&AN=369910616 | Other diagnosis (not depression/MDD) |  |
| 359 , Tancredi, D. J., Slee, C. K., Jerant, A., Franks, P., Nettiksimmons, J., Cipri, C., Gottfeld, D., Huerta, J., Feldman, M. D., Jackson-Triche, M., Kelly-Reif, S., Hudnut, A., Olson, S., Shelton, J., Kravitz, R. L. Targeted versus tailored multimedia patient engagement to enhance depression recognition and treatment in primary care: randomized controlled trial protocol for the AMEP2 study. BMC health services research. 2013. 13:141 , https://ovidsp.ovid.com/ovidweb.cgi?T=JS&CSC=Y&NEWS=N&PAGE=fulltext&D=emed14&AN=369910069 | Include this article |  |
| 359 , Tancredi, D. J., Slee, C. K., Jerant, A., Franks, P., Nettiksimmons, J., Cipri, C., Gottfeld, D., Huerta, J., Feldman, M. D., Jackson-Triche, M., Kelly-Reif, S., Hudnut, A., Olson, S., Shelton, J., Kravitz, R. L. Targeted versus tailored multimedia patient engagement to enhance depression recognition and treatment in primary care: randomized controlled trial protocol for the AMEP2 study. BMC health services research. 2013. 13:141 , https://ovidsp.ovid.com/ovidweb.cgi?T=JS&CSC=Y&NEWS=N&PAGE=fulltext&D=emed14&AN=369910069 |  | Inappropriate intervention |
| 362 , Mouthaan, J., Sijbrandij, M., de Vries, G. J., Reitsma, J. B., van de Schoot, R., Goslings, J. C., Luitse, J. S., Bakker, F. C., Gersons, B. P., Olff, M. Internet-based early intervention to prevent posttraumatic stress disorder in injury patients: randomized controlled trial. Journal of medical Internet research. 2013. 15:e165 , https://ovidsp.ovid.com/ovidweb.cgi?T=JS&CSC=Y&NEWS=N&PAGE=fulltext&D=emed14&AN=563041139 | Other diagnosis (not depression/MDD) |  |
| 364 , Powell, J., Hamborg, T., Stallard, N., Burls, A., McSorley, J., Bennett, K., Griffiths, K. M., Christensen, H. Effectiveness of a web-based cognitive-behavioral tool to improve mental well-being in the general population: randomized controlled trial. Journal of medical Internet research. 2013. 15:e2 , https://ovidsp.ovid.com/ovidweb.cgi?T=JS&CSC=Y&NEWS=N&PAGE=fulltext&D=emed14&AN=369159658 | Other diagnosis (not depression/MDD) |  |
| 366 , Donker, T., Bennett, K., Bennett, A., Mackinnon, A., van Straten, A., Cuijpers, P., Christensen, H., Griffiths, K. M. Internet-delivered interpersonal psychotherapy versus internet-delivered cognitive behavioral therapy for adults with depressive symptoms: randomized controlled noninferiority trial. Journal of medical Internet research. 2013. 15:e82 , https://ovidsp.ovid.com/ovidweb.cgi?T=JS&CSC=Y&NEWS=N&PAGE=fulltext&D=emed14&AN=563002480 | Other diagnosis (not depression/MDD) |  |
| 367 , Spates, C. R., Kalata, A. H., Ozeki, S., Stanton, C. E., Peters, S. Initial Open Trial of a Computerized Behavioral Activation Treatment for Depression. Behavior Modification. 2013. 37:259-297 , https://ovidsp.ovid.com/ovidweb.cgi?T=JS&CSC=Y&NEWS=N&PAGE=fulltext&D=emed14&AN=1368799180 | Include this article |  |
|  |  | Inappropriate study design |
| 368 , Hackworth, N. J., Matthews, J., Burke, K., Petrovic, Z., Klein, B., Northam, E. A., Kyrios, M., Chiechomski, L., Cameron, F. J. Improving mental health of adolescents with Type 1 diabetes: protocol for a randomized controlled trial of the Nothing Ventured Nothing Gained online adolescent and parenting support intervention. BMC public health. 2013. 13:1185 , https://ovidsp.ovid.com/ovidweb.cgi?T=JS&CSC=Y&NEWS=N&PAGE=fulltext&D=emed14&AN=563086701 | Other diagnosis (not depression/MDD) |  |
| 369 , Jones, R. B., Goldsmith, L., Williams, C. J., Kamel Boulos, M. N. Accuracy of geographically targeted internet advertisements on google adwords for recruitment in a randomized trial. Journal of medical Internet research. 2012. 14:e84 , https://ovidsp.ovid.com/ovidweb.cgi?T=JS&CSC=Y&NEWS=N&PAGE=fulltext&D=emed13&AN=365731240 | Inappropriate study design |  |
| 370 , Ritterband, L. M., Bailey, E. T., Thorndike, F. P., Lord, H. R., Farrell-Carnahan, L., Baum, L. D. Initial evaluation of an Internet intervention to improve the sleep of cancer survivors with insomnia. Psycho-Oncology. 2012. 21:695-705 , https://ovidsp.ovid.com/ovidweb.cgi?T=JS&CSC=Y&NEWS=N&PAGE=fulltext&D=emed13&AN=51405712 | Other diagnosis (not depression/MDD) |  |
| 371 , Bowler, J. O., MacKintosh, B., Dunn, B. D., Mathews, A., Dalgleish, T., Hoppitt, L. A comparison of cognitive bias modification for interpretation and computerized cognitive behavior therapy: Effects on anxiety, depression, attentional control, and interpretive bias. Journal of Consulting and Clinical Psychology. 2012. 80:1021-1033 , http://www.apa.org/pubs/journals/ccp/index.aspx https://ovidsp.ovid.com/ovidweb.cgi?T=JS&CSC=Y&NEWS=N&PAGE=fulltext&D=emed13&AN=368358430 | Other diagnosis (not depression/MDD) |  |
| 372 , Proudfoot, J., Parker, G., Manicavasagar, V., Hadzi-Pavlovic, D., Whitton, A., Nicholas, J., Smith, M., Burckhardt, R. Effects of adjunctive peer support on perceptions of illness control and understanding in an online psychoeducation program for bipolar disorder: A randomised controlled trial. Journal of Affective Disorders. 2012. 142:98-105 , https://ovidsp.ovid.com/ovidweb.cgi?T=JS&CSC=Y&NEWS=N&PAGE=fulltext&D=emed13&AN=52140627 | Other diagnosis (not depression/MDD) |  |
| 373 , Kelly, P. J., Kay-Lambkin, F. J., Baker, A. L., Deane, F. P., Brooks, A. C., Mitchell, A., Marshall, S., Whittington, M., Dingle, G. A. Study protocol: a randomized controlled trial of a computer-based depression and substance abuse intervention for people attending residential substance abuse treatment. BMC public health. 2012. 12:113 , https://ovidsp.ovid.com/ovidweb.cgi?T=JS&CSC=Y&NEWS=N&PAGE=fulltext&D=emed13&AN=365051019 | Other diagnosis (not depression/MDD) |  |
| 374 , Elgan, T. H., Hansson, H., Zetterlind, U., Kartengren, N., Leifman, H. Design of a Web-based individual coping and alcohol-intervention program (web-ICAIP) for children of parents with alcohol problems: study protocol for a randomized controlled trial. BMC public health. 2012. 12:35 , https://ovidsp.ovid.com/ovidweb.cgi?T=JS&CSC=Y&NEWS=N&PAGE=fulltext&D=emed13&AN=365050974 | Other diagnosis (not depression/MDD) |  |
| 375 , Fleming, T., Dixon, R., Frampton, C., Merry, S. A pragmatic randomized controlled trial of computerized CBT (SPARX) for symptoms of depression among adolescents excluded from mainstream education. Behavioural and cognitive psychotherapy. 2012. 40:529-541 , https://ovidsp.ovid.com/ovidweb.cgi?T=JS&CSC=Y&NEWS=N&PAGE=fulltext&D=emed13&AN=366380450 | Inappropriate study design |  |
| 377 , Carlbring, P., Degerman, N., Jonsson, J., Andersson, G. Internet-Based Treatment of Pathological Gambling with a Three-Year Follow-Up. Cognitive Behaviour Therapy. 2012. 41:321-334 , https://ovidsp.ovid.com/ovidweb.cgi?T=JS&CSC=Y&NEWS=N&PAGE=fulltext&D=emed13&AN=368717227 | Other diagnosis (not depression/MDD) |  |
| 378 , Bessell, A., Brough, V., Clarke, A., Harcourt, D., Moss, T. P., Rumsey, N. Evaluation of the effectiveness of Face IT, a computer-based psychosocial intervention for disfigurement-related distress. Psychology, health & medicine. 2012. 17:565-577 , https://ovidsp.ovid.com/ovidweb.cgi?T=JS&CSC=Y&NEWS=N&PAGE=fulltext&D=emed13&AN=366358113 | Other diagnosis (not depression/MDD) |  |
| 379 , Farrer, L., Christensen, H., Griffiths, K. M., Mackinnon, A. Internet-based CBT for depression with and without telephone tracking in a national helpline: Randomised controlled trial. PLoS ONE. 2011. 6:http://www.plosone.org/article/fetchObjectAttachment.action?uri=info%3Adoi%2F10.1371%2Fjournal.pone.0028099&representation=PDF https://ovidsp.ovid.com/ovidweb.cgi?T=JS&CSC=Y&NEWS=N&PAGE=fulltext&D=emed12&AN=363007801 | Other diagnosis (not depression/MDD) |  |
| 382 , Welch, G., Allen, N. A., Zagarins, S. E., Stamp, K. D., Bursell, S. E., Kedziora, R. J. Comprehensive diabetes management program for poorly controlled hispanic type 2 patients at a community health center. Diabetes Educator. 2011. 37:680-688 , https://ovidsp.ovid.com/ovidweb.cgi?T=JS&CSC=Y&NEWS=N&PAGE=fulltext&D=emed12&AN=362560322 | Other diagnosis (not depression/MDD) |  |
| 383 , Tossmann, H. P., Jonas, B., Tensil, M. D., Lang, P., Struber, E. A controlled trial of an internet-based intervention program for cannabis users. Cyberpsychology, behavior and social networking. 2011. 14:673-679 , https://ovidsp.ovid.com/ovidweb.cgi?T=JS&CSC=Y&NEWS=N&PAGE=fulltext&D=emed12&AN=560066828 | Other diagnosis (not depression/MDD) |  |
| 386 , Kay-Lambkin, F., Baker, A., Lewin, T., Carr, V. Acceptability of a clinician-assisted computerized psychological intervention for comorbid mental health and substance use problems: treatment adherence data from a randomized controlled trial. Journal of medical Internet research. 2011. 13:e11 , https://ovidsp.ovid.com/ovidweb.cgi?T=JS&CSC=Y&NEWS=N&PAGE=fulltext&D=emed12&AN=361842601 |  | Other diagnosis (not depression/MDD) |
| 386 , Kay-Lambkin, F., Baker, A., Lewin, T., Carr, V. Acceptability of a clinician-assisted computerized psychological intervention for comorbid mental health and substance use problems: treatment adherence data from a randomized controlled trial. Journal of medical Internet research. 2011. 13:e11 , https://ovidsp.ovid.com/ovidweb.cgi?T=JS&CSC=Y&NEWS=N&PAGE=fulltext&D=emed12&AN=361842601 | Include this article |  |
| 387 , Paxling, B., Almlov, J., Dahlin, M., Carlbring, P., Breitholtz, E., Eriksson, T., Andersson, G. Guided Internet-delivered cognitive behavior therapy for generalized anxiety disorder: A randomized controlled trial. Cognitive Behaviour Therapy. 2011. 40:159-173 , https://ovidsp.ovid.com/ovidweb.cgi?T=JS&CSC=Y&NEWS=N&PAGE=fulltext&D=emed12&AN=362455450 | Other diagnosis (not depression/MDD) |  |
| 388 , Zhu, L., Song, W. Q., Yue, Y. H., Liu, L. Effect of computer-assisted cognitive training on the cognitive function and depression in patients with brain injury. [Chinese]. Chinese Journal of Cerebrovascular Diseases. 2011. 8:508-512 , https://ovidsp.ovid.com/ovidweb.cgi?T=JS&CSC=Y&NEWS=N&PAGE=fulltext&D=emed12&AN=362879418 | Inappropriate intervention |  |
| 390 , Gainsbury, S., Blaszczynski, A. A systematic review of Internet-based therapy for the treatment of addictions. Clinical Psychology Review. 2011. 31:490-498 , https://ovidsp.ovid.com/ovidweb.cgi?T=JS&CSC=Y&NEWS=N&PAGE=fulltext&D=emed12&AN=51186508 | Inappropriate study design |  |
| 391 , Hoifodt, R. S., Strom, C., Kolstrup, N., Eisemann, M., Waterloo, K. Effectiveness of cognitive behavioural therapy in primary health care: A review. Family Practice. 2011. 28:489-504 , https://ovidsp.ovid.com/ovidweb.cgi?T=JS&CSC=Y&NEWS=N&PAGE=fulltext&D=emed12&AN=362585803 | Inappropriate study design |  |
| 392 , Spence, J., Titov, N., Dear, B. F., Johnston, L., Solley, K., Lorian, C., Wootton, B., Zou, J., Schwenke, G. Randomized controlled trial of Internet-delivered cognitive behavioral therapy for posttraumatic stress disorder. Depression and Anxiety. 2011. 28:541-550 , https://ovidsp.ovid.com/ovidweb.cgi?T=JS&CSC=Y&NEWS=N&PAGE=fulltext&D=emed12&AN=362075025 | Other diagnosis (not depression/MDD) |  |
| 393 , Andrews, G., Cuijpers, P., Craske, M. G., McEvoy, P., Titov, N. Computer therapy for the anxiety and depressive disorders is effective, acceptable and practical health care: a meta-analysis. PloS one. 2010. 5:e13196 , https://ovidsp.ovid.com/ovidweb.cgi?T=JS&CSC=Y&NEWS=N&PAGE=fulltext&D=emed11&AN=360272934 | Inappropriate study design |  |
| 394 , Gerhards, S. A., de Graaf, L. E., Jacobs, L. E., Severens, J. L., Huibers, M. J., Arntz, A., Riper, H., Widdershoven, G., Metsemakers, J. F., Evers, S. M. Economic evaluation of online computerised cognitive-behavioural therapy without support for depression in primary care: randomised trial. The British journal of psychiatry : the journal of mental science. 2010. 196:310-318 , https://ovidsp.ovid.com/ovidweb.cgi?T=JS&CSC=Y&NEWS=N&PAGE=fulltext&D=emed11&AN=358815623 | Include this article |  |
|  |  | Inappropriate outcomes |
| 395 , Richardson, T., Stallard, P., Velleman, S. Computerised cognitive behavioural therapy for the prevention and treatment of depression and anxiety in children and adolescents: A systematic review. Clinical Child and Family Psychology Review. 2010. 13:275-290 , https://ovidsp.ovid.com/ovidweb.cgi?T=JS&CSC=Y&NEWS=N&PAGE=fulltext&D=emed11&AN=50945355 | Inappropriate study design |  |
| 397 , Glynn, S. M., Randolph, E. T., Garrick, T., Lui, A. A proof of concept trial of an online psychoeducational program for relatives of both veterans and civilians living with schizophrenia. Psychiatric rehabilitation journal. 2010. 33:278-287 , https://ovidsp.ovid.com/ovidweb.cgi?T=JS&CSC=Y&NEWS=N&PAGE=fulltext&D=emed11&AN=358847559 | Other diagnosis (not depression/MDD) |  |
| 398 , Knaevelsrud, C., Maercker, A. Long-term effects of an internet-based treatment for posttraumatic stress. Cognitive Behaviour Therapy. 2010. 39:72-77 , https://ovidsp.ovid.com/ovidweb.cgi?T=JS&CSC=Y&NEWS=N&PAGE=fulltext&D=emed11&AN=358654735 | Other diagnosis (not depression/MDD) |  |
| 400 , Wims, E., Titov, N., Andrews, G., Choi, I. Clinician-assisted Internet-based treatment is effective for panic: A randomized controlled trial. Australian and New Zealand Journal of Psychiatry. 2010. 44:599-607 , http://anp.sagepub.com/content/by/year https://ovidsp.ovid.com/ovidweb.cgi?T=JS&CSC=Y&NEWS=N&PAGE=fulltext&D=emed11&AN=359052177 | Other diagnosis (not depression/MDD) |  |
| 401 , Christensen, H., Griffiths, K. M., Farrer, L. Adherence in internet interventions for anxiety and depression. Journal of Medical Internet Research. 2009. 11:http://www.jmir.org/article/viewFile/jmir_v11i2e13/2 https://ovidsp.ovid.com/ovidweb.cgi?T=JS&CSC=Y&NEWS=N&PAGE=fulltext&D=emed11&AN=354703306 | Inappropriate study design |  |
| 402 , Waller, R., Gilbody, S. Barriers to the uptake of computerized cognitive behavioural therapy: A systematic review of the quantitative and qualitative evidence. Psychological Medicine. 2009. 39:705-712 , https://ovidsp.ovid.com/ovidweb.cgi?T=JS&CSC=Y&NEWS=N&PAGE=fulltext&D=emed11&AN=355183395 | Inappropriate study design |  |
| 403 , Askins, M. A., Sahler, O. J., Sherman, S. A., Fairclough, D. L., Butler, R. W., Katz, E. R., Dolgin, M. J., Varni, J. W., Noll, R. B., Phipps, S. Report from a multi-institutional randomized clinical trial examining computer-assisted problem-solving skills training for English- and Spanish-speaking mothers of children with newly diagnosed cancer. Journal of pediatric psychology. 2009. 34:551-563 , https://ovidsp.ovid.com/ovidweb.cgi?T=JS&CSC=Y&NEWS=N&PAGE=fulltext&D=emed11&AN=355166018 | Other diagnosis (not depression/MDD) |  |
| 404 , Abbott, J. A. M., Kaldo, V., Klein, B., Austin, D., Hamilton, C., Piterman, L., Williams, B., Andersson, G. A cluster randomised trial of an internet-based intervention program for tinnitus distress in an industrial setting. Cognitive Behaviour Therapy. 2009. 38:162-173 , https://ovidsp.ovid.com/ovidweb.cgi?T=JS&CSC=Y&NEWS=N&PAGE=fulltext&D=emed11&AN=358169673 | Other diagnosis (not depression/MDD) |  |
| 405 , Pierce, L. L., Steiner, V. L., Khuder, S. A., Govoni, A. L., Horn, L. J. The effect of a Web-based stroke intervention on carers' well-being and survivors' use of healthcare services. Disability and rehabilitation. 2009. 31:1676-1684 , https://ovidsp.ovid.com/ovidweb.cgi?T=JS&CSC=Y&NEWS=N&PAGE=fulltext&D=emed11&AN=355870603 | Other diagnosis (not depression/MDD) |  |
| 406 , Hoek, W., Schuurmans, J., Koot, H. M., Cuijpers, P. Prevention of depression and anxiety in adolescents: A randomized controlled trial testing the efficacy and mechanisms of Internet-based self-help problem-solving therapy. Trials. 2009. 10:93 , http://www.trialsjournal.com/home/ https://ovidsp.ovid.com/ovidweb.cgi?T=JS&CSC=Y&NEWS=N&PAGE=fulltext&D=emed11&AN=355697982 | Inappropriate study design |  |
| 407 , Ruwaard, J., Broeksteeg, J., Schrieken, B., Emmelkamp, P., Lange, A. Web-based therapist-assisted cognitive behavioral treatment of panic symptoms: A randomized controlled trial with a three-year follow-up. Journal of Anxiety Disorders. 2010. 24:387-396 , https://ovidsp.ovid.com/ovidweb.cgi?T=JS&CSC=Y&NEWS=N&PAGE=fulltext&D=emed11&AN=50829140 | Other diagnosis (not depression/MDD) |  |
| 408 , Williams, D. A., Kuper, D., Segar, M., Mohan, N., Sheth, M., Clauw, D. J. Internet-enhanced management of fibromyalgia: A randomized controlled trial. Pain. 2010. 151:694-702 , https://ovidsp.ovid.com/ovidweb.cgi?T=JS&CSC=Y&NEWS=N&PAGE=fulltext&D=emed11&AN=51076471 | Other diagnosis (not depression/MDD) |  |
| 409 , Titov, N., Andrews, G., Robinson, E., Schwencke, G., Johnston, L., Solley, K., Choi, I. Clinician-assisted Internet-based treatment is effective for generalized anxiety disorder: Randomized controlled trial. Australian and New Zealand Journal of Psychiatry. 2009. 43:905-912 , https://ovidsp.ovid.com/ovidweb.cgi?T=JS&CSC=Y&NEWS=N&PAGE=fulltext&D=emed11&AN=355735795 | Other diagnosis (not depression/MDD) |  |
| 410 , McHugh, R. K., Murray, H. W., Barlow, D. H. Balancing fidelity and adaptation in the dissemination of empirically-supported treatments: The promise of transdiagnostic interventions. Behaviour Research and Therapy. 2009. 47:946-953 , https://ovidsp.ovid.com/ovidweb.cgi?T=JS&CSC=Y&NEWS=N&PAGE=fulltext&D=emed11&AN=50594420 | Inappropriate study design |  |
| 411 , van Bastelaar, K. M. P., Pouwer, F., Cuijpers, P., Twisk, J. W. R., Snoek, F. J. Web-based cognitive behavioural therapy (W-CBT) for diabetes patients with co-morbid depression: Design of a randomised controlled trial. BMC Psychiatry. 2008. 8 (no pagination):https://ovidsp.ovid.com/ovidweb.cgi?T=JS&CSC=Y&NEWS=N&PAGE=fulltext&D=emed10&AN=351486696 | Other diagnosis (not depression/MDD) |  |
| 413 , Rabius, V., Pike, K. J., Wiatrek, D., McAlister, A. L. Comparing internet assistance for smoking cessation: 13-month follow-up of a six-arm randomized controlled trial. Journal of medical Internet research. 2008. 10:e45 , https://ovidsp.ovid.com/ovidweb.cgi?T=JS&CSC=Y&NEWS=N&PAGE=fulltext&D=emed10&AN=550129004 | Other diagnosis (not depression/MDD) |  |
| 414 , Carey, J. C., Wade, S. L., Wolfe, C. R. Lessons learned: The effect of prior technology use on web-based interventions. Cyberpsychology and Behavior. 2008. 11:188-195 , https://ovidsp.ovid.com/ovidweb.cgi?T=JS&CSC=Y&NEWS=N&PAGE=fulltext&D=emed10&AN=351555917 | Other diagnosis (not depression/MDD) |  |
| 415 , Pier, C., Austin, D. W., Klein, B., Mitchell, J., Schattner, P., Ciechomski, L., Gilson, K. J., Pierce, D., Shandley, K., Wade, V. A controlled trial of internet-based cognitive-behavioural therapy for panic disorder with face-to-face support from a general practitioner or email support from a psychologist. Mental Health in Family Medicine. 2008. 5:29-39 , https://ovidsp.ovid.com/ovidweb.cgi?T=JS&CSC=Y&NEWS=N&PAGE=fulltext&D=emed10&AN=352331677 | Other diagnosis (not depression/MDD) |  |
| 416 , Ruwaard, J., Lange, A., Bouwman, M., Broeksteeg, J., Schrieken, B. E-mailed standardized cognitive behavioural treatment of work-related stress: A randomized controlled trial. Cognitive Behaviour Therapy. 2007. 36:179-192 , https://ovidsp.ovid.com/ovidweb.cgi?T=JS&CSC=Y&NEWS=N&PAGE=fulltext&D=emed10&AN=47604690 | Other diagnosis (not depression/MDD) |  |
| 417 , Titov, N. Status of computerized cognitive behavioural therapy for adults. Australian and New Zealand Journal of Psychiatry. 2007. 41:95-114 , https://ovidsp.ovid.com/ovidweb.cgi?T=JS&CSC=Y&NEWS=N&PAGE=fulltext&D=emed10&AN=46705622 | Inappropriate study design |  |
| 418 , Knaevelsrud, C., Maercker, A. Does the quality of the working alliance predict treatment outcome in online psychotherapy for traumatized patients?. Journal of Medical Internet Research. 2006. 8:e31 , http://www.jmir.org/2006/4/e31 https://ovidsp.ovid.com/ovidweb.cgi?T=JS&CSC=Y&NEWS=N&PAGE=fulltext&D=emed9&AN=47450202 | Other diagnosis (not depression/MDD) |  |
| 419 , Kaltenthaler, E., Brazier, J., De Nigris, E., Tumur, I., Ferriter, M., Beverley, C., Parry, G., Rooney, G., Sutcliffe, P. Computerised cognitive behaviour therapy for depression and anxiety update: a systematic review and economic evaluation. Health technology assessment (Winchester, England). 2006. 10:iii, xi-xiv, 1-168 , https://ovidsp.ovid.com/ovidweb.cgi?T=JS&CSC=Y&NEWS=N&PAGE=fulltext&D=emed9&AN=44996783 | Inappropriate study design |  |
| 420 , Wright, J. H., Wright, A. S., Albano, A. M., Basco, M. R., Goldsmith, L. J., Raffield, T., Otto, M. W. Computer-assisted cognitive therapy for depression: Maintaining efficacy while reducing therapist time. American Journal of Psychiatry. 2005. 162:1158-1164 , https://ovidsp.ovid.com/ovidweb.cgi?T=JS&CSC=Y&NEWS=N&PAGE=fulltext&D=emed9&AN=40770699 | Include this article |  |
|  |  | Inappropriate intervention |
| 421 , Van Den Berg, S., Shapiro, D. A., Bickerstaffe, D., Cavanagh, K. Computerized cognitive-behaviour therapy for anxiety and depression: A practical solution to the shortage of trained therapists. Journal of Psychiatric and Mental Health Nursing. 2004. 11:508-513 , https://ovidsp.ovid.com/ovidweb.cgi?T=JS&CSC=Y&NEWS=N&PAGE=fulltext&D=emed8&AN=39329321 | Include this article |  |
| 421 , Van Den Berg, S., Shapiro, D. A., Bickerstaffe, D., Cavanagh, K. Computerized cognitive-behaviour therapy for anxiety and depression: A practical solution to the shortage of trained therapists. Journal of Psychiatric and Mental Health Nursing. 2004. 11:508-513 , https://ovidsp.ovid.com/ovidweb.cgi?T=JS&CSC=Y&NEWS=N&PAGE=fulltext&D=emed8&AN=39329321 |  | Inappropriate study design |
| 422 , Proudfoot, J. G. Computer-based treatment for anxiety and depression: Is it feasible? Is it effective?. Neuroscience and Biobehavioral Reviews. 2004. 28:353-363 , https://ovidsp.ovid.com/ovidweb.cgi?T=JS&CSC=Y&NEWS=N&PAGE=fulltext&D=emed8&AN=38844382 | Inappropriate study design |  |
| 423 , Christensen, H., Griffiths, K. M., Korten, A. Web-based cognitive behavior therapy: Analysis of site usage and changes in depression and anxiety scores. Journal of Medical Internet Research. 2002. 4:29-40 , http://www.jmir.org/2002/1/e3/ https://ovidsp.ovid.com/ovidweb.cgi?T=JS&CSC=Y&NEWS=N&PAGE=fulltext&D=emed7&AN=38964178 | Other diagnosis (not depression/MDD) |  |
| 425 , Dominguez-Rodriguez, A., Martinez-Luna, S. C., Hernandez Jimenez, M. J., De La Rosa-Gomez, A., Arenas-Landgrave, P., Esquivel Santovena, E. E., Arzola-Sanchez, C., Alvarez Silva, J., Solis Nicolas, A. M., Colmenero Guadian, A. M., Ramirez-Martinez, F. R., Vargas, R. O. C. A Self-Applied Multi-Component Psychological Online Intervention Based on UX, for the Prevention of Complicated Grief Disorder in the Mexican Population During the COVID-19 Outbreak: Protocol of a Randomized Clinical Trial. Frontiers in Psychology. 2021. 12:644782 , https://ovidsp.ovid.com/ovidweb.cgi?T=JS&CSC=Y&NEWS=N&PAGE=fulltext&D=pmnm&AN=33854466 | Other diagnosis (not depression/MDD) |  |
| 428 , LeLaurin, J. H., Lamba, A. H., Eliazar-Macke, N. D., Schmitzberger, M. K., Freytes, I. M., Dang, S., Vogel, W. B., Levy, C. E., Klanchar, S. A., Beyth, R. J., Shorr, R. I., Uphold, C. R. Postdischarge Intervention for Stroke Caregivers: Protocol for a Randomized Controlled Trial. JMIR Research Protocols. 2020. 9:e21799 , https://ovidsp.ovid.com/ovidweb.cgi?T=JS&CSC=Y&NEWS=N&PAGE=fulltext&D=pmnm&AN=33174856 | Other diagnosis (not depression/MDD) |  |
| 429 , Maybery, D., Reupert, A., Bartholomew, C., Cuff, R., Duncan, Z., Foster, K., Matar, J., Pettenuzzo, L. A Web-Based Intervention for Young Adults Whose Parents Have a Mental Illness or Substance Use Concern: Protocol for a Randomized Controlled Trial. JMIR Research Protocols. 2020. 9:e15626 , https://ovidsp.ovid.com/ovidweb.cgi?T=JS&CSC=Y&NEWS=N&PAGE=fulltext&D=pmnm&AN=32554368 | Other diagnosis (not depression/MDD) |  |
| 430 , Bade, B. C., Gan, G., Li, F., Lu, L., Tanoue, L., Silvestri, G. A., Irwin, M. L. "Randomized trial of physical activity on quality of life and lung cancer biomarkers in patients with advanced stage lung cancer: a pilot study". BMC Cancer. 2021. 21:352 , https://ovidsp.ovid.com/ovidweb.cgi?T=JS&CSC=Y&NEWS=N&PAGE=fulltext&D=medl&AN=33794808 | Other diagnosis (not depression/MDD) |  |
| 431 , Do, R., Lee, S., Kim, J. S., Cho, M., Shin, H., Jang, M., Shin, M. S. Effectiveness and dissemination of computer-based cognitive behavioral therapy for depressed adolescents: Effective and accessible to whom?. Journal of Affective Disorders. 2021. 282:885-893 , https://ovidsp.ovid.com/ovidweb.cgi?T=JS&CSC=Y&NEWS=N&PAGE=fulltext&D=medl&AN=33601732 | Inappropriate study design |  |
| 432 , Haile, C., Kirk, A., Cogan, N., Janssen, X., Gibson, A. M., MacDonald, B. Pilot Testing of a Nudge-Based Digital Intervention (Welbot) to Improve Sedentary Behaviour and Wellbeing in the Workplace. International Journal of Environmental Research & Public Health [Electronic Resource]. 2020. 17:10 , https://ovidsp.ovid.com/ovidweb.cgi?T=JS&CSC=Y&NEWS=N&PAGE=fulltext&D=medl&AN=32784956 | Other diagnosis (not depression/MDD) |  |
| 437 , De Jaegere, E., van Landschoot, R., van Heeringen, K., van Spijker, B. A. J., Kerkhof, Ajfm,Mokkenstorm, J. K., Portzky, G. The online treatment of suicidal ideation: A randomised controlled trial of an unguided web-based intervention. Behaviour Research & Therapy. 2019. 119:103406 , https://ovidsp.ovid.com/ovidweb.cgi?T=JS&CSC=Y&NEWS=N&PAGE=fulltext&D=med16&AN=31176889 |  | Other diagnosis (not depression/MDD) |
| 437 , De Jaegere, E., van Landschoot, R., van Heeringen, K., van Spijker, B. A. J., Kerkhof, Ajfm,Mokkenstorm, J. K., Portzky, G. The online treatment of suicidal ideation: A randomised controlled trial of an unguided web-based intervention. Behaviour Research & Therapy. 2019. 119:103406 , https://ovidsp.ovid.com/ovidweb.cgi?T=JS&CSC=Y&NEWS=N&PAGE=fulltext&D=med16&AN=31176889 | Include this article |  |
| 439 , Vicent-Gil, M., Raventos, B., Marin-Martinez, E. D., Gonzalez-Simarro, S., Martinez-Aran, A., Bonnin, C. D. M., Trujols, J., Perez-Blanco, J., de Diego-Adelino, J., Puigdemont, D., Serra-Blasco, M., Cardoner, N., Portella, M. J. Testing the efficacy of INtegral Cognitive REMediation (INCREM) in major depressive disorder: study protocol for a randomized clinical trial. BMC Psychiatry. 2019. 19:135 , https://ovidsp.ovid.com/ovidweb.cgi?T=JS&CSC=Y&NEWS=N&PAGE=fulltext&D=med16&AN=31060604 |  | Inappropriate outcomes |
|  | Include this article |  |
| 440 , Pierce, J. S., Wasserman, R., Enlow, P., Aroian, K., Lee, J., Wysocki, T. Benefit finding among parents of young children with type 1 diabetes. Pediatric Diabetes. 2019. 20:652-660 , https://ovidsp.ovid.com/ovidweb.cgi?T=JS&CSC=Y&NEWS=N&PAGE=fulltext&D=med16&AN=31006941 | Other diagnosis (not depression/MDD) |  |
| 441 , Mills, L., Meiser, B., Ahmad, R., Schofield, P. R., Peate, M., Levitan, C., Trevena, L., Barlow-Stewart, K., Dobbins, T., Christensen, H., Sherman, K. A., Dunlop, K., Mitchell, P. B. A cluster randomized controlled trial of an online psychoeducational intervention for people with a family history of depression. BMC Psychiatry. 2019. 19:29 , https://ovidsp.ovid.com/ovidweb.cgi?T=JS&CSC=Y&NEWS=N&PAGE=fulltext&D=med16&AN=30654777 | Other diagnosis (not depression/MDD) |  |
| 442 , Shann, C., Martin, A., Chester, A., Ruddock, S. Effectiveness and application of an online leadership intervention to promote mental health and reduce depression-related stigma in organizations. Journal of Occupational Health Psychology. 2019. 24:20-35 , https://ovidsp.ovid.com/ovidweb.cgi?T=JS&CSC=Y&NEWS=N&PAGE=fulltext&D=med16&AN=29300098 | Other diagnosis (not depression/MDD) |  |
| 443 , Lin, J., Klatt, L. I., McCracken, L. M., Baumeister, H. Psychological flexibility mediates the effect of an online-based acceptance and commitment therapy for chronic pain: an investigation of change processes. Pain. 2018. 159:663-672 , https://ovidsp.ovid.com/ovidweb.cgi?T=JS&CSC=Y&NEWS=N&PAGE=fulltext&D=med15&AN=29320375 | Other diagnosis (not depression/MDD) |  |
| 444 , Witlox, M., Kraaij, V., Garnefski, N., de Waal, M. W. M., Smit, F., Hoencamp, E., Gussekloo, J., Bohlmeijer, E. T., Spinhoven, P. An Internet-based Acceptance and Commitment Therapy intervention for older adults with anxiety complaints: study protocol for a cluster randomized controlled trial. Trials [Electronic Resource]. 2018. 19:502 , https://ovidsp.ovid.com/ovidweb.cgi?T=JS&CSC=Y&NEWS=N&PAGE=fulltext&D=med15&AN=30223873 | Other diagnosis (not depression/MDD) |  |
| 445 , Wells, M. J., Owen, J. J., McCray, L. W., Bishop, L. B., Eells, T. D., Brown, G. K., Richards, D., Thase, M. E., Wright, J. H. Computer-Assisted Cognitive-Behavior Therapy for Depression in Primary Care: Systematic Review and Meta-Analysis. The Primary Care Companion to CNS Disorders. 2018. 20:01 , https://ovidsp.ovid.com/ovidweb.cgi?T=JS&CSC=Y&NEWS=N&PAGE=fulltext&D=med15&AN=29570963 | Inappropriate study design |  |
| 446 , Pottgen, J., Moss-Morris, R., Wendebourg, J. M., Feddersen, L., Lau, S., Kopke, S., Meyer, B., Friede, T., Penner, I. K., Heesen, C., Gold, S. M. Randomised controlled trial of a self-guided online fatigue intervention in multiple sclerosis. Journal of Neurology, Neurosurgery & Psychiatry. 2018. 89:970-976 , https://ovidsp.ovid.com/ovidweb.cgi?T=JS&CSC=Y&NEWS=N&PAGE=fulltext&D=med15&AN=29549193 | Other diagnosis (not depression/MDD) |  |
| 447 , Wallin, E., Norlund, F., Olsson, E. M. G., Burell, G., Held, C., Carlsson, T. Treatment Activity, User Satisfaction, and Experienced Usability of Internet-Based Cognitive Behavioral Therapy for Adults With Depression and Anxiety After a Myocardial Infarction: Mixed-Methods Study. Journal of Medical Internet Research. 2018. 20:e87 , https://ovidsp.ovid.com/ovidweb.cgi?T=JS&CSC=Y&NEWS=N&PAGE=fulltext&D=med15&AN=29549067 | Include this article |  |
|  |  | Other diagnosis (not depression/MDD) |
| 448 , Lin, J., Paganini, S., Sander, L., Luking, M., Ebert, D. D., Buhrman, M., Andersson, G., Baumeister, H. An Internet-Based Intervention for Chronic Pain. Deutsches Arzteblatt International. 2017. 114:681-688 , https://ovidsp.ovid.com/ovidweb.cgi?T=JS&CSC=Y&NEWS=N&PAGE=fulltext&D=med14&AN=29082858 | Other diagnosis (not depression/MDD) |  |
| 449 , Newby, J., Robins, L., Wilhelm, K., Smith, J., Fletcher, T., Gillis, I., Ma, T., Finch, A., Campbell, L., Andrews, G. Web-Based Cognitive Behavior Therapy for Depression in People With Diabetes Mellitus: A Randomized Controlled Trial. Journal of Medical Internet Research. 2017. 19:e157 , https://ovidsp.ovid.com/ovidweb.cgi?T=JS&CSC=Y&NEWS=N&PAGE=fulltext&D=med14&AN=28506956 | Include this article |  |
|  |  | Other diagnosis (not depression/MDD) |
| 450 , Beukes, E. W., Baguley, D. M., Allen, P. M., Manchaiah, V., Andersson, G. Guided Internet-based versus face-to-face clinical care in the management of tinnitus: study protocol for a multi-centre randomised controlled trial. Trials [Electronic Resource]. 2017. 18:186 , https://ovidsp.ovid.com/ovidweb.cgi?T=JS&CSC=Y&NEWS=N&PAGE=fulltext&D=med14&AN=28431551 | Other diagnosis (not depression/MDD) |  |
| 453 , Martinez, V., Rojas, G., Martinez, P., Gaete, J., Zitko, P., Vohringer, P. A., Araya, R. Computer-Assisted Cognitive-Behavioral Therapy to Treat Adolescents With Depression in Primary Health Care Centers in Santiago, Chile: A Randomized Controlled Trial. Frontiers in psychiatry Frontiers Research Foundation. 2019. 10:552 , https://ovidsp.ovid.com/ovidweb.cgi?T=JS&CSC=Y&NEWS=N&PAGE=fulltext&D=pmnm4&AN=31417440 | Inappropriate study design |  |
| 454 , Rocha, A., Camacho, R., Ruwaard, J., Riper, H. Using multi-relational data mining to discriminate blended therapy efficiency on patients based on log data. Internet Interventions. 2018. 12:176-180 , https://ovidsp.ovid.com/ovidweb.cgi?T=JS&CSC=Y&NEWS=N&PAGE=fulltext&D=pmnm4&AN=30135781 | Inappropriate study design |  |
| 455 , Cavanagh, K., Churchard, A., O'Hanlon, P., Mundy, T., Votolato, P., Jones, F., Gu, J., Strauss, C. A Randomised Controlled Trial of a Brief Online Mindfulness-Based Intervention in a Non-clinical Population: Replication and Extension. Mindfulness. 2018. 9:1191-1205 , https://ovidsp.ovid.com/ovidweb.cgi?T=JS&CSC=Y&NEWS=N&PAGE=fulltext&D=pmnm4&AN=30100934 | Other diagnosis (not depression/MDD) |  |
| 456 , Pfirrmann, D., Haller, N., Huber, Y., Jung, P., Lieb, K., Gockel, I., Poplawska, K., Schattenberg, J. M., Simon, P. Applicability of a Web-Based, Individualized Exercise Intervention in Patients With Liver Disease, Cystic Fibrosis, Esophageal Cancer, and Psychiatric Disorders: Process Evaluation of 4 Ongoing Clinical Trials. JMIR Research Protocols. 2018. 7:e106 , https://ovidsp.ovid.com/ovidweb.cgi?T=JS&CSC=Y&NEWS=N&PAGE=fulltext&D=pmnm4&AN=29789277 | Other diagnosis (not depression/MDD) |  |
| 457 , Fleming, J. N., Treiber, F., McGillicuddy, J., Gebregziabher, M., Taber, D. J. Improving Transplant Medication Safety Through a Pharmacist-Empowered, Patient-Centered, mHealth-Based Intervention: TRANSAFE Rx Study Protocol. JMIR Research Protocols. 2018. 7:e59 , https://ovidsp.ovid.com/ovidweb.cgi?T=JS&CSC=Y&NEWS=N&PAGE=fulltext&D=pmnm4&AN=29500161 | Other diagnosis (not depression/MDD) |  |
| 458 , Fletcher, R., May, C., Attia, J., Garfield, C. F., Skinner, G. Text-Based Program Addressing the Mental Health of Soon-to-be and New Fathers (SMS4dads): Protocol for a Randomized Controlled Trial. JMIR Research Protocols. 2018. 7:e37 , https://ovidsp.ovid.com/ovidweb.cgi?T=JS&CSC=Y&NEWS=N&PAGE=fulltext&D=pmnm4&AN=29410387 | Other diagnosis (not depression/MDD) |  |
| 460 , Ownby, R. L., Acevedo, A., Waldrop-Valverde, D., Caballero, J., Simonson, M., Davenport, R., Kondwani, K., Jacobs, R. J. A Mobile App for Chronic Disease Self-Management: Protocol for a Randomized Controlled Trial. JMIR Research Protocols. 2017. 6:e53 , https://ovidsp.ovid.com/ovidweb.cgi?T=JS&CSC=Y&NEWS=N&PAGE=fulltext&D=pmnm4&AN=28381395 | Other diagnosis (not depression/MDD) |  |
| 461 , Crawford, J., Wilhelm, K., Robins, L., Proudfoot, J. Writing for Health: Rationale and Protocol for a Randomized Controlled Trial of Internet-Based Benefit-Finding Writing for Adults With Type 1 or Type 2 Diabetes. JMIR Research Protocols. 2017. 6:e42 , https://ovidsp.ovid.com/ovidweb.cgi?T=JS&CSC=Y&NEWS=N&PAGE=fulltext&D=pmnm4&AN=28292741 | Other diagnosis (not depression/MDD) |  |
| 463 , Boots, L. M., de Vugt, M. E., Kempen, G. I., Verhey, F. R. Effectiveness of the blended care self-management program "Partner in Balance" for early-stage dementia caregivers: study protocol for a randomized controlled trial. Trials [Electronic Resource]. 2016. 17:231 , https://ovidsp.ovid.com/ovidweb.cgi?T=JS&CSC=Y&NEWS=N&PAGE=fulltext&D=med13&AN=27142676 | Other diagnosis (not depression/MDD) |  |
| 464 , Weidt, S., Zai, G., Drabe, N., Delsignore, A., Bruehl, A. B., Klaghofer, R., Rufer, M. Affective regulation in trichotillomania before and after self-help interventions. Journal of Psychiatric Research. 2016. 75:7-13 , https://ovidsp.ovid.com/ovidweb.cgi?T=JS&CSC=Y&NEWS=N&PAGE=fulltext&D=med13&AN=26783728 | Other diagnosis (not depression/MDD) |  |
| 465 , van Eeden, M., Kootker, J. A., Evers, S. M., van Heugten, C. M., Geurts, A. C., van Mastrigt, G. A. An economic evaluation of an augmented cognitive behavioural intervention vs. computerized cognitive training for post-stroke depressive symptoms. BMC Neurology. 2015. 15:266 , https://ovidsp.ovid.com/ovidweb.cgi?T=JS&CSC=Y&NEWS=N&PAGE=fulltext&D=med12&AN=26715040 | Include this article |  |
| 465 , van Eeden, M., Kootker, J. A., Evers, S. M., van Heugten, C. M., Geurts, A. C., van Mastrigt, G. A. An economic evaluation of an augmented cognitive behavioural intervention vs. computerized cognitive training for post-stroke depressive symptoms. BMC Neurology. 2015. 15:266 , https://ovidsp.ovid.com/ovidweb.cgi?T=JS&CSC=Y&NEWS=N&PAGE=fulltext&D=med12&AN=26715040 |  | Other diagnosis (not depression/MDD) |
| 468 , Nobis, S., Lehr, D., Ebert, D. D., Baumeister, H., Snoek, F., Riper, H., Berking, M. Efficacy of a web-based intervention with mobile phone support in treating depressive symptoms in adults with type 1 and type 2 diabetes: a randomized controlled trial. Diabetes Care. 2015. 38:776-83 , https://ovidsp.ovid.com/ovidweb.cgi?T=JS&CSC=Y&NEWS=N&PAGE=fulltext&D=med12&AN=25710923 | Include this article |  |
|  |  | Other diagnosis (not depression/MDD) |
| 470 , Robinson, J., Hetrick, S., Cox, G., Bendall, S., Yung, A., Pirkis, J. The safety and acceptability of delivering an online intervention to secondary students at risk of suicide: findings from a pilot study. Early intervention in psychiatry. 2015. 9:498-506 , https://ovidsp.ovid.com/ovidweb.cgi?T=JS&CSC=Y&NEWS=N&PAGE=fulltext&D=med12&AN=24684927 | Inappropriate study design |  |
| 471 , Ahmedani, B. K., Crotty, N., Abdulhak, M. M., Ondersma, S. J. Pilot feasibility study of a brief, tailored mobile health intervention for depression among patients with chronic pain. Behavioral Medicine. 2015. 41:25-32 , https://ovidsp.ovid.com/ovidweb.cgi?T=JS&CSC=Y&NEWS=N&PAGE=fulltext&D=med12&AN=24313728 | Include this article |  |
| 471 , Ahmedani, B. K., Crotty, N., Abdulhak, M. M., Ondersma, S. J. Pilot feasibility study of a brief, tailored mobile health intervention for depression among patients with chronic pain. Behavioral Medicine. 2015. 41:25-32 , https://ovidsp.ovid.com/ovidweb.cgi?T=JS&CSC=Y&NEWS=N&PAGE=fulltext&D=med12&AN=24313728 |  | Other diagnosis (not depression/MDD) |
| 472 , Habibovic, M., Denollet, J., Cuijpers, P., Spek, V. R., van den Broek, K. C., Warmerdam, L., van der Voort, P. H., Herrman, J. P., Bouwels, L., Valk, S. S., Alings, M., Theuns, D. A., Pedersen, S. S. E-health to manage distress in patients with an implantable cardioverter-defibrillator: primary results of the WEBCARE trial. Psychosomatic Medicine. 2014. 76:593-602 , https://ovidsp.ovid.com/ovidweb.cgi?T=JS&CSC=Y&NEWS=N&PAGE=fulltext&D=med11&AN=25264974 | Other diagnosis (not depression/MDD) |  |
| 474 , Morimoto, S. S., Wexler, B. E., Liu, J., Hu, W., Seirup, J., Alexopoulos, G. S. Neuroplasticity-based computerized cognitive remediation for treatment-resistant geriatric depression. Nature communications. 2014. 5:4579 , https://ovidsp.ovid.com/ovidweb.cgi?T=JS&CSC=Y&NEWS=N&PAGE=fulltext&D=med11&AN=25093396 | Include this article |  |
|  |  | Inappropriate outcomes |
| 475 , Christensen, H., Batterham, P., Calear, A. Online interventions for anxiety disorders. Current Opinion in Psychiatry. 2014. 27:7-13 , https://ovidsp.ovid.com/ovidweb.cgi?T=JS&CSC=Y&NEWS=N&PAGE=fulltext&D=med11&AN=24257123 | Inappropriate study design |  |
| 476 , Elbers, N. A., Akkermans, A. J., Cuijpers, P., Bruinvels, D. J. Effectiveness of a web-based intervention for injured claimants: a randomized controlled trial. Trials [Electronic Resource]. 2013. 14:227 , https://ovidsp.ovid.com/ovidweb.cgi?T=JS&CSC=Y&NEWS=N&PAGE=fulltext&D=med10&AN=23870540 | Other diagnosis (not depression/MDD) |  |
| 477 , Shigaki, C. L., Smarr, K. L., Siva, C., Ge, B., Musser, D., Johnson, R. RAHelp: an online intervention for individuals with rheumatoid arthritis. Arthritis care & research. 2013. 65:1573-81 , https://ovidsp.ovid.com/ovidweb.cgi?T=JS&CSC=Y&NEWS=N&PAGE=fulltext&D=med10&AN=23666599 | Other diagnosis (not depression/MDD) |  |
| 478 , Blom, M. M., Bosmans, J. E., Cuijpers, P., Zarit, S. H., Pot, A. M. Effectiveness and cost-effectiveness of an internet intervention for family caregivers of people with dementia: design of a randomized controlled trial. BMC Psychiatry. 2013. 13:17 , https://ovidsp.ovid.com/ovidweb.cgi?T=JS&CSC=Y&NEWS=N&PAGE=fulltext&D=med10&AN=23305463 | Other diagnosis (not depression/MDD) |  |
| 479 , Duffecy, J., Sanford, S., Wagner, L., Begale, M., Nawacki, E., Mohr, D. C. Project onward: an innovative e-health intervention for cancer survivors. Psycho-Oncology. 2013. 22:947-51 , https://ovidsp.ovid.com/ovidweb.cgi?T=JS&CSC=Y&NEWS=N&PAGE=fulltext&D=med10&AN=22438297 | Other diagnosis (not depression/MDD) |  |
| 480 , Leone de Voogd, E., Wiers, R. W., Zwitser, R. J., Salemink, E. Emotional working memory training as an online intervention for adolescent anxiety and depression: A randomised controlled trial. Australian Journal of Psychology. 2016. 68:228-238 , https://ovidsp.ovid.com/ovidweb.cgi?T=JS&CSC=Y&NEWS=N&PAGE=fulltext&D=pmnm3&AN=27917000 | Inappropriate study design |  |
| 481 , Gander, F., Proyer, R. T., Ruch, W. Positive Psychology Interventions Addressing Pleasure, Engagement, Meaning, Positive Relationships, and Accomplishment Increase Well-Being and Ameliorate Depressive Symptoms: A Randomized, Placebo-Controlled Online Study. Frontiers in Psychology. 2016. 7:686 , https://ovidsp.ovid.com/ovidweb.cgi?T=JS&CSC=Y&NEWS=N&PAGE=fulltext&D=pmnm3&AN=27242600 | Other diagnosis (not depression/MDD) |  |
| 482 , Clarke, J., Proudfoot, J., Whitton, A., Birch, M. R., Boyd, M., Parker, G., Manicavasagar, V., Hadzi-Pavlovic, D., Fogarty, A. Therapeutic Alliance With a Fully Automated Mobile Phone and Web-Based Intervention: Secondary Analysis of a Randomized Controlled Trial. JMIR Mental Health. 2016. 3:e10 , https://ovidsp.ovid.com/ovidweb.cgi?T=JS&CSC=Y&NEWS=N&PAGE=fulltext&D=pmnm3&AN=26917096 | Include this article |  |
|  |  | Other diagnosis (not depression/MDD) |
| 483 , Proyer, R. T., Gander, F., Wellenzohn, S., Ruch, W. Strengths-based positive psychology interventions: a randomized placebo-controlled online trial on long-term effects for a signature strengths- vs. a lesser strengths-intervention. Frontiers in Psychology. 2015. 6:456 , https://ovidsp.ovid.com/ovidweb.cgi?T=JS&CSC=Y&NEWS=N&PAGE=fulltext&D=pmnm3&AN=25954221 | Other diagnosis (not depression/MDD) |  |
| 484 , Clarke, J., Vatiliotis, V., Verge, C. F., Holmes-Walker, J., Campbell, L. V., Wilhelm, K., Proudfoot, J. A mobile phone and web-based intervention for improving mental well-being in young people with type 1 diabetes: design of a randomized controlled trial. JMIR Research Protocols. 2015. 4:e50 , https://ovidsp.ovid.com/ovidweb.cgi?T=JS&CSC=Y&NEWS=N&PAGE=fulltext&D=pmnm3&AN=25944212 | Other diagnosis (not depression/MDD) |  |
| 485 , Antezana, G., Bidargaddi, N., Blake, V., Schrader, G., Kaambwa, B., Quinn, S., Orlowski, S., Winsall, M., Battersby, M. Development of an online well-being intervention for young people: an evaluation protocol. JMIR Research Protocols. 2015. 4:e48 , https://ovidsp.ovid.com/ovidweb.cgi?T=JS&CSC=Y&NEWS=N&PAGE=fulltext&D=pmnm3&AN=25929201 | Other diagnosis (not depression/MDD) |  |
| 488 , Seidman, D. F., Westmaas, J. L., Goldband, S., Rabius, V., Katkin, E. S., Pike, K. J., Wiatrek, D., Sloan, R. P. Randomized controlled trial of an interactive internet smoking cessation program with long-term follow-up. Annals of Behavioral Medicine. 2010. 39:48-60 , https://ovidsp.ovid.com/ovidweb.cgi?T=JS&CSC=Y&NEWS=N&PAGE=fulltext&D=med8&AN=20177844 | Other diagnosis (not depression/MDD) |  |
| 490 , Meyer, B., Berger, T., Caspar, F., Beevers, C. G., Andersson, G., Weiss, M. Effectiveness of a novel integrative online treatment for depression (Deprexis): randomized controlled trial. Journal of Medical Internet Research. 2009. 11:e15 , https://ovidsp.ovid.com/ovidweb.cgi?T=JS&CSC=Y&NEWS=N&PAGE=fulltext&D=med7&AN=19632969 | Include this article |  |
| 490 , Meyer, B., Berger, T., Caspar, F., Beevers, C. G., Andersson, G., Weiss, M. Effectiveness of a novel integrative online treatment for depression (Deprexis): randomized controlled trial. Journal of Medical Internet Research. 2009. 11:e15 , https://ovidsp.ovid.com/ovidweb.cgi?T=JS&CSC=Y&NEWS=N&PAGE=fulltext&D=med7&AN=19632969 |  | Other diagnosis (not depression/MDD) |
| 491 , Vinogradov, S., Fisher, M., Holland, C., Shelly, W., Wolkowitz, O., Mellon, S. H. Is serum brain-derived neurotrophic factor a biomarker for cognitive enhancement in schizophrenia?. Biological Psychiatry. 2009. 66:549-53 , https://ovidsp.ovid.com/ovidweb.cgi?T=JS&CSC=Y&NEWS=N&PAGE=fulltext&D=med7&AN=19368899 | Other diagnosis (not depression/MDD) |  |
| 492 , O'Kearney, R., Kang, K., Christensen, H., Griffiths, K. A controlled trial of a school-based Internet program for reducing depressive symptoms in adolescent girls. Depression & Anxiety. 2009. 26:65-72 , https://ovidsp.ovid.com/ovidweb.cgi?T=JS&CSC=Y&NEWS=N&PAGE=fulltext&D=med7&AN=18828141 | Inappropriate study design |  |
| 494 , Elgamal, S., McKinnon, M. C., Ramakrishnan, K., Joffe, R. T., MacQueen, G. Successful computer-assisted cognitive remediation therapy in patients with unipolar depression: a proof of principle study. Psychological Medicine. 2007. 37:1229-38 , https://ovidsp.ovid.com/ovidweb.cgi?T=JS&CSC=Y&NEWS=N&PAGE=fulltext&D=med6&AN=17610766 | Include this article |  |
| 494 , Elgamal, S., McKinnon, M. C., Ramakrishnan, K., Joffe, R. T., MacQueen, G. Successful computer-assisted cognitive remediation therapy in patients with unipolar depression: a proof of principle study. Psychological Medicine. 2007. 37:1229-38 , https://ovidsp.ovid.com/ovidweb.cgi?T=JS&CSC=Y&NEWS=N&PAGE=fulltext&D=med6&AN=17610766 |  | Inappropriate intervention |
| 495 , Ames, M. R. Heather,Glenton, Claire,Lewin, Simon,Tamrat, Tigest,Akama, Eliud,Leon, Natalie . Clients' perceptions and experiences of targeted digital communication accessible via mobile devices for reproductive, maternal, newborn, child, and adolescent health: a qualitative evidence synthesis [Systematic Review]. Cochrane Database of Systematic Reviews. 2019. 10:10 , https://ovidsp.ovid.com/ovidweb.cgi?T=JS&CSC=Y&NEWS=N&PAGE=fulltext&D=coch&AN=00075320-100000000-11849 | Other diagnosis (not depression/MDD) |  |
| 496 , Boele, Florien W., Rooney, Alasdair G., Bulbeck, Helen,Sherwood, Paula . Interventions to help support caregivers of people with a brain or spinal cord tumour [Systematic Review]. Cochrane Database of Systematic Reviews. 2019. 7:7 , https://ovidsp.ovid.com/ovidweb.cgi?T=JS&CSC=Y&NEWS=N&PAGE=fulltext&D=coch&AN=00075320-100000000-10995 | Inappropriate study design |  |
| 497 , Behbod, Behrooz,Sharma, Mohit,Baxi, Ruchi,Roseby, Robert,Webster, Premila . Family and carer smoking control programmes for reducing children's exposure to environmental tobacco smoke [Systematic Review]. Cochrane Database of Systematic Reviews. 2018. 1:1 , https://ovidsp.ovid.com/ovidweb.cgi?T=JS&CSC=Y&NEWS=N&PAGE=fulltext&D=coch&AN=00075320-100000000-00184 | Inappropriate study design |  |
| 498 , Laver, Kate E., Adey-Wakeling, Zoe,Crotty, Maria,Lannin, Natasha A., George, Stacey,Sherrington, Catherine . Telerehabilitation services for stroke [Systematic Review]. Cochrane Database of Systematic Reviews. 2020. 1:1 , https://ovidsp.ovid.com/ovidweb.cgi?T=JS&CSC=Y&NEWS=N&PAGE=fulltext&D=coch&AN=00075320-100000000-08647 | Inappropriate study design |  |
| 499 , Gates, Nicola J., Rutjes, W. S. Anne,Di Nisio, Marcello,Karim, Salman,Chong, Lee-Yee,March, Evrim,Martinez, Gabriel,Vernooij, W. M. Robin . Computerised cognitive training for 12 or more weeks for maintaining cognitive function in cognitively healthy people in late life [Systematic Review]. Cochrane Database of Systematic Reviews. 2020. 2:2 , https://ovidsp.ovid.com/ovidweb.cgi?T=JS&CSC=Y&NEWS=N&PAGE=fulltext&D=coch&AN=00075320-100000000-10685 | Other diagnosis (not depression/MDD) |  |
| 500 , Burge, Angela T., Cox, Narelle S., Abramson, Michael J., Holland, Anne E. Interventions for promoting physical activity in people with chronic obstructive pulmonary disease (COPD) [Systematic Review]. Cochrane Database of Systematic Reviews. 2020. 4:4 , https://ovidsp.ovid.com/ovidweb.cgi?T=JS&CSC=Y&NEWS=N&PAGE=fulltext&D=coch&AN=00075320-100000000-11029 | Inappropriate study design |  |
| 501 , Taylor, M. Gemma,Dalili, Michael N., Semwal, Monika,Civljak, Marta,Sheikh, Aziz,Car, Josip . Internet-based interventions for smoking cessation [Systematic Review]. Cochrane Database of Systematic Reviews. 2017. 9:9 , https://ovidsp.ovid.com/ovidweb.cgi?T=JS&CSC=Y&NEWS=N&PAGE=fulltext&D=coch&AN=00075320-100000000-05753 | Inappropriate study design |  |
| 502 , Schell, Lisa K., Monsef, Ina,Wockel, Achim,Skoetz, Nicole . Mindfulness-based stress reduction for women diagnosed with breast cancer [Systematic Review]. Cochrane Database of Systematic Reviews. 2019. 3:3 , https://ovidsp.ovid.com/ovidweb.cgi?T=JS&CSC=Y&NEWS=N&PAGE=fulltext&D=coch&AN=00075320-100000000-09925 | Inappropriate study design |  |
| 503 , Perry, Amanda E., Martyn-St James, Marrissa,Burns, Lucy,Hewitt, Catherine,Glanville, Julie M., Aboaja, Anne,Thakkar, Pratish,Santosh Kumar, Murthy Keshava,Pearson, Caroline,Wright, Kath . Interventions for female drug-using offenders [Systematic Review]. Cochrane Database of Systematic Reviews. 2019. 12:12 , https://ovidsp.ovid.com/ovidweb.cgi?T=JS&CSC=Y&NEWS=N&PAGE=fulltext&D=coch&AN=00075320-100000000-09321 | Other diagnosis (not depression/MDD) |  |
| 504 , Morgan, Emily H., Schoonees, Anel,Sriram, Urshila,Faure, Marlyn,Seguin-Fowler, Rebecca A. Caregiver involvement in interventions for improving children's dietary intake and physical activity behaviors [Systematic Review]. Cochrane Database of Systematic Reviews. 2020. 1:1 , https://ovidsp.ovid.com/ovidweb.cgi?T=JS&CSC=Y&NEWS=N&PAGE=fulltext&D=coch&AN=00075320-100000000-10949 | Inappropriate study design |  |
| 505 , Verkuijlen, Jolijn,Verhaak, Christianne,Nelen, L. D. M. Willianne,Wilkinson, Jack,Farquhar, Cindy . Psychological and educational interventions for subfertile men and women [Systematic Review]. Cochrane Database of Systematic Reviews. 2016. 3:3 , https://ovidsp.ovid.com/ovidweb.cgi?T=JS&CSC=Y&NEWS=N&PAGE=fulltext&D=coch&AN=00075320-100000000-09434 | Other diagnosis (not depression/MDD) |  |
| 506 , Orgeta, Vasiliki,McDonald, Kathryn R., Poliakoff, Ellen,Hindle, Vincent John,Clare, Linda,Leroi, Iracema . Cognitive training interventions for dementia and mild cognitive impairment in Parkinson's disease [Systematic Review]. Cochrane Database of Systematic Reviews. 2020. 2:2 , https://ovidsp.ovid.com/ovidweb.cgi?T=JS&CSC=Y&NEWS=N&PAGE=fulltext&D=coch&AN=00075320-100000000-10350 | Inappropriate study design |  |
| 507 , MacArthur, Georgina,Caldwell, Deborah M., Redmore, James,Watkins, Sarah H., Kipping, Ruth,White, James,Chittleborough, Catherine,Langford, Rebecca,Er, Vanessa,Lingam, Raghu,Pasch, Keryn,Gunnell, David,Hickman, Matthew,Campbell, Rona . Individual-, family-, and school-level interventions targeting multiple risk behaviours in young people [Systematic Review]. Cochrane Database of Systematic Reviews. 2018. 10:10 , https://ovidsp.ovid.com/ovidweb.cgi?T=JS&CSC=Y&NEWS=N&PAGE=fulltext&D=coch&AN=00075320-100000000-08272 | Inappropriate study design |  |
| 508 , Thabrew, Hiran,Stasiak, Karolina,Hetrick, Sarah E., Wong, Stephen,Huss, Jessica H., Merry, Sally N. E-Health interventions for anxiety and depression in children and adolescents with long-term physical conditions [Systematic Review]. Cochrane Database of Systematic Reviews. 2018. 8:8 , https://ovidsp.ovid.com/ovidweb.cgi?T=JS&CSC=Y&NEWS=N&PAGE=fulltext&D=coch&AN=00075320-100000000-11495 | Inappropriate study design |  |
| 509 , Stevenson, Jessica K., Campbell, Zoe C., Webster, Angela C., Chow, Clara K., Tong, Allison,Craig, Jonathan C., Campbell, Katrina L., Lee, W. S. Vincent . eHealth interventions for people with chronic kidney disease [Systematic Review]. Cochrane Database of Systematic Reviews. 2019. 8:8 , https://ovidsp.ovid.com/ovidweb.cgi?T=JS&CSC=Y&NEWS=N&PAGE=fulltext&D=coch&AN=00075320-100000000-10788 | Inappropriate study design |  |
| 510 , Machmutow, Katja,Meister, Ramona,Jansen, Alessa,Kriston, Levente,Watzke, Birgit,Harter, Christian Martin,Liebherz, Sarah . Comparative effectiveness of continuation and maintenance treatments for persistent depressive disorder in adults [Systematic Review]. Cochrane Database of Systematic Reviews. 2019. 5:5 , https://ovidsp.ovid.com/ovidweb.cgi?T=JS&CSC=Y&NEWS=N&PAGE=fulltext&D=coch&AN=00075320-100000000-11280 | Inappropriate study design |  |
| 511 , Loetscher, Tobias,Potter, Kristy-Jane,Wong, Dana,das Nair, Roshan . Cognitive rehabilitation for attention deficits following stroke [Systematic Review]. Cochrane Database of Systematic Reviews. 2019. 11:11 , https://ovidsp.ovid.com/ovidweb.cgi?T=JS&CSC=Y&NEWS=N&PAGE=fulltext&D=coch&AN=00075320-100000000-01848 | Inappropriate study design |  |
| 512 , Radtke, Thomas,Nevitt, Sarah J., Hebestreit, Helge,Kriemler, Susi . Physical exercise training for cystic fibrosis [Systematic Review]. Cochrane Database of Systematic Reviews. 2017. 11:11 , https://ovidsp.ovid.com/ovidweb.cgi?T=JS&CSC=Y&NEWS=N&PAGE=fulltext&D=coch&AN=00075320-100000000-01784 | Other diagnosis (not depression/MDD) |  |
| 513 , Rose, Louise,Schultz, Marcus J., Cardwell, Chris R., Jouvet, Philippe,McAuley, Danny F., Blackwood, Bronagh . Automated versus non-automated weaning for reducing the duration of mechanical ventilation for critically ill adults and children [Systematic Review]. Cochrane Database of Systematic Reviews. 2018. 12:12 , https://ovidsp.ovid.com/ovidweb.cgi?T=JS&CSC=Y&NEWS=N&PAGE=fulltext&D=coch&AN=00075320-100000000-07589 | Inappropriate study design |  |
| 514 , Brown, Sarah J., Khasteganan, Nazanin,Brown, Katherine,Hegarty, Kelsey,Carter, Grace J., Tarzia, Laura,Feder, Gene,O'Doherty, Lorna . Psychosocial interventions for survivors of rape and sexual assault experienced during adulthood [Protocol]. Cochrane Database of Systematic Reviews. 2019. 11:11 , https://ovidsp.ovid.com/ovidweb.cgi?T=JS&CSC=Y&NEWS=N&PAGE=fulltext&D=coch&AN=00075320-100000000-11867 | Other diagnosis (not depression/MDD) |  |
| 515 , Tailor, Vijay,Bossi, Manuela,Bunce, Catey,Greenwood, John A., Dahlmann-Noor, Annegret . Binocular versus standard occlusion or blurring treatment for unilateral amblyopia in children aged three to eight years [Systematic Review]. Cochrane Database of Systematic Reviews. 2015. 8:8 , https://ovidsp.ovid.com/ovidweb.cgi?T=JS&CSC=Y&NEWS=N&PAGE=fulltext&D=coch&AN=00075320-100000000-09765 | Other diagnosis (not depression/MDD) |  |
| 516 , Gates, Nicola J., Rutjes, W. S. Anne,Di Nisio, Marcello,Karim, Salman,Chong, Lee-Yee,March, Evrim,Martinez, Gabriel,Vernooij, W. M. Robin . Computerised cognitive training for maintaining cognitive function in cognitively healthy people in midlife [Systematic Review]. Cochrane Database of Systematic Reviews. 2019. 3:3 , https://ovidsp.ovid.com/ovidweb.cgi?T=JS&CSC=Y&NEWS=N&PAGE=fulltext&D=coch&AN=00075320-100000000-10686 | Inappropriate study design |  |
| 517 , Mehrholz, Jan,Pohl, Marcus,Platz, Thomas,Kugler, Joachim,Elsner, Bernhard . Electromechanical and robot-assisted arm training for improving activities of daily living, arm function, and arm muscle strength after stroke [Systematic Review]. Cochrane Database of Systematic Reviews. 2018. 9:9 , https://ovidsp.ovid.com/ovidweb.cgi?T=JS&CSC=Y&NEWS=N&PAGE=fulltext&D=coch&AN=00075320-100000000-05583 | Other diagnosis (not depression/MDD) |  |
| 518 , Posadzki, Pawel,Mastellos, Nikolaos,Ryan, Rebecca,Gunn, Laura H., Felix, Lambert M., Pappas, Yannis,Gagnon, MariePierre,Julious, Steven A., Xiang, Liming,Oldenburg, Brian,Car, Josip . Automated telephone communication systems for preventive healthcare and management of long-term conditions [Systematic Review]. Cochrane Database of Systematic Reviews. 2016. 12:12 , https://ovidsp.ovid.com/ovidweb.cgi?T=JS&CSC=Y&NEWS=N&PAGE=fulltext&D=coch&AN=00075320-100000000-08305 | Inappropriate study design |  |
| 519 , Gillies, Donna,Maiocchi, Licia,Bhandari, Abhishta P., Taylor, Fiona,Gray, Carl,O'Brien, Louise . Psychological therapies for children and adolescents exposed to trauma [Systematic Review]. Cochrane Database of Systematic Reviews. 2016. 10:10 , https://ovidsp.ovid.com/ovidweb.cgi?T=JS&CSC=Y&NEWS=N&PAGE=fulltext&D=coch&AN=00075320-100000000-10800 | Inappropriate study design |  |
| 520 , Treanor, Charlene J., McMenamin, Una C., O'Neill, Roisin F., Cardwell, Chris R., Clarke, Mike J., Cantwell, Marie,Donnelly, Michael . Non-pharmacological interventions for cognitive impairment due to systemic cancer treatment [Systematic Review]. Cochrane Database of Systematic Reviews. 2016. 8:8 , https://ovidsp.ovid.com/ovidweb.cgi?T=JS&CSC=Y&NEWS=N&PAGE=fulltext&D=coch&AN=00075320-100000000-09750 | Inappropriate study design |  |
| 521 , Scott, David A., Mills, Moyra,Black, Amanda,Cantwell, Marie,Campbell, Anna,Cardwell, Chris R., Porter, Sam,Donnelly, Michael . Multidimensional rehabilitation programmes for adult cancer survivors [Systematic Review]. Cochrane Database of Systematic Reviews. 2016. 9:9 , https://ovidsp.ovid.com/ovidweb.cgi?T=JS&CSC=Y&NEWS=N&PAGE=fulltext&D=coch&AN=00075320-100000000-06327 | Inappropriate study design |  |
| 522 , Hetrick, Sarah E., Cox, Georgina R., Witt, Katrina G., Bir, Julliet J., Merry, Sally N. Cognitive behavioural therapy (CBT), third-wave CBT and interpersonal therapy (IPT) based interventions for preventing depression in children and adolescents [Systematic Review]. Cochrane Database of Systematic Reviews. 2016. 8:8 , https://ovidsp.ovid.com/ovidweb.cgi?T=JS&CSC=Y&NEWS=N&PAGE=fulltext&D=coch&AN=00075320-100000000-02295 | Inappropriate study design |  |
| 523 , Olthuis, Janine V., Watt, Margo C., Bailey, Kristen,Hayden, Jill A., Stewart, Sherry H. Therapist-supported Internet cognitive behavioural therapy for anxiety disorders in adults [Systematic Review]. Cochrane Database of Systematic Reviews. 2016. 3:3 , https://ovidsp.ovid.com/ovidweb.cgi?T=JS&CSC=Y&NEWS=N&PAGE=fulltext&D=coch&AN=00075320-100000000-09995 | Other diagnosis (not depression/MDD) |  |
| 524 , Hawton, Keith,Witt, Katrina G., Taylor Salisbury, Tatiana L., Arensman, Ella,Gunnell, David,Hazell, Philip,Townsend, Ellen,van Heeringen, Kees . Psychosocial interventions for self-harm in adults [Systematic Review]. Cochrane Database of Systematic Reviews. 2016. 5:5 , https://ovidsp.ovid.com/ovidweb.cgi?T=JS&CSC=Y&NEWS=N&PAGE=fulltext&D=coch&AN=00075320-100000000-10591 | Inappropriate study design |  |
| 525 , Devi, Reena,Singh, Sally J., Powell, John,Fulton, Emily A., Igbinedion, Ewemade,Rees, Karen . Internet-based interventions for the secondary prevention of coronary heart disease [Systematic Review]. Cochrane Database of Systematic Reviews. 2015. 12:12 , https://ovidsp.ovid.com/ovidweb.cgi?T=JS&CSC=Y&NEWS=N&PAGE=fulltext&D=coch&AN=00075320-100000000-07753 | Inappropriate study design |  |
| 526 , Candy, Bridget,Jones, Louise,Vickerstaff, Victoria,Tookman, Adrian,King, Michael . Interventions for sexual dysfunction following treatments for cancer in women [Systematic Review]. Cochrane Database of Systematic Reviews. 2016. 2:2 , https://ovidsp.ovid.com/ovidweb.cgi?T=JS&CSC=Y&NEWS=N&PAGE=fulltext&D=coch&AN=00075320-100000000-04517 | Inappropriate study design |  |
| 527 , Gillaizeau, Florence,Chan, Ellis,Trinquart, Ludovic,Colombet, Isabelle,Walton, R. T., RegeWalther, Myriam,Burnand, Bernard,Durieux, Pierre . Computerized advice on drug dosage to improve prescribing practice [Systematic Review]. Cochrane Database of Systematic Reviews. 2013. 11:11 , https://ovidsp.ovid.com/ovidweb.cgi?T=JS&CSC=Y&NEWS=N&PAGE=fulltext&D=coch&AN=00075320-100000000-01968 | Inappropriate study design |  |
| 528 , Valimaki, Maritta,Hatonen, Heli,Lahti, Mari,Kuosmanen, Lauri,Adams, Clive E. Information and communication technology in patient education and support for people with schizophrenia [Systematic Review]. Cochrane Database of Systematic Reviews. 2012. 10:10 , https://ovidsp.ovid.com/ovidweb.cgi?T=JS&CSC=Y&NEWS=N&PAGE=fulltext&D=coch&AN=00075320-100000000-05832 | Inappropriate study design |  |
| 529 , Valimaki, Maritta,Hatonen, Heli,Adams, Clive E., Arifulla, Dinah,Kostiainen, Johanna,Kostamo, Paivi . Information and communication technology-based cognitive remediation for supporting treatment compliance for people with serious mental illness [Protocol]. Cochrane Database of Systematic Reviews. 2012. 5:5 , https://ovidsp.ovid.com/ovidweb.cgi?T=JS&CSC=Y&NEWS=N&PAGE=fulltext&D=coch&AN=00075320-100000000-08222 | Inappropriate study design |  |
| 531 , Loughnan Sa, Newby J. M. Haskelberg H. Mahoney A. Kladnitski N. Smith J. Black E. Holt C. Milgrom J. Austin M. P. Andrews G. Internet-based cognitive behavioural therapy (iCBT) for perinatal anxiety and depression versus treatment as usual: study protocol for two randomised controlled trials. Trials. 2018. 19:https://ovidsp.ovid.com/ovidweb.cgi?T=JS&CSC=Y&NEWS=N&PAGE=fulltext&D=cctr&AN=CN-01450184 | Other diagnosis (not depression/MDD) |  |
| 538 , Tement S, Zorjan S. Lavric M. Postuvan V. Plohl N. A randomized controlled trial to improve psychological detachment from work and well-being among employees: a study protocol comparing online CBT-based and mindfulness interventions. BMC public health. . 20:1708 , https://ovidsp.ovid.com/ovidweb.cgi?T=JS&CSC=Y&NEWS=N&PAGE=fulltext&D=cctr&AN=CN-02201374 | Other diagnosis (not depression/MDD) |  |
| 551 , Kahlke F, Berger T. Schulz A. Baumeister H. Berking M. Cuijpers P. Bruffaerts R. Auerbach R. P. Kessler R. C. Ebert D. D. Efficacy and cost-effectiveness of an unguided, internet-based self-help intervention for social anxiety disorder in university students: protocol of a randomized controlled trial. BMC psychiatry. . 19:197 , https://ovidsp.ovid.com/ovidweb.cgi?T=JS&CSC=Y&NEWS=N&PAGE=fulltext&D=cctr&AN=CN-01958877 | Other diagnosis (not depression/MDD) |  |
| 554 , Van Voorhees B, Gladstone T. R. G. Sobowale K. Brown C. H. Aaby D. A. Terrizzi D. A. Canel J. Ching E. Berry A. D. Cantorna J. Eder M. Beardslee W. Fitzgibbon M. Marko-Holguin M. Schiffer L. Lee M. de Forest S. A. Sykes E. E. Suor J. H. Crawford T. J. Burkhouse K. L. Goodwin B. C. Bell C. 24-Month Outcomes of Primary Care Web-Based Depression Prevention Intervention in Adolescents: randomized Clinical Trial. Journal of medical Internet research. . 22:e16802 , https://ovidsp.ovid.com/ovidweb.cgi?T=JS&CSC=Y&NEWS=N&PAGE=fulltext&D=cctr&AN=CN-02202986 | Inappropriate study design |  |
| 556 , Nct, . Enhancing Processing Speed and Executive Functioning in Depressed Older Adults With Computerized Cognitive Training. https://clinicaltrials.gov/show/NCT04836533. 2021. :https://ovidsp.ovid.com/ovidweb.cgi?T=JS&CSC=Y&NEWS=N&PAGE=fulltext&D=cctr&AN=CN-02253633 | Include this article |  |
|  |  | Other diagnosis (not depression/MDD) |
| 560 , Smith Ka, Bradbury K. Essery R. Pollet S. Mowbray F. Slodkowska-Barabasz J. Denison-Day J. Hayter V. Kelly J. Somerville J. Zhang J. Grey E. Western M. Ferrey A. E. Krusche A. Stuart B. Mutrie N. Robinson S. Yao G. L. Griffiths G. Robinson L. Rossor M. Gallacher J. Griffin S. Kendrick T. Rathod S. Gudgin B. Phillips R. Stokes T. Niven J. Little P. Yardley L. The Active Brains Digital Intervention to Reduce Cognitive Decline in Older Adults: protocol for a Feasibility Randomized Controlled Trial. JMIR research protocols. 2020. 9:https://ovidsp.ovid.com/ovidweb.cgi?T=JS&CSC=Y&NEWS=N&PAGE=fulltext&D=cctr&AN=CN-02236761 | Other diagnosis (not depression/MDD) |  |
| 563 , Actrn, . Action: PACT. Be Active. Online. A trial to promote physical activity in young people with cystic fibrosis. http://www.who.int/trialsearch/Trial2.aspx?TrialID=ACTRN12617001009303. 2017. :https://ovidsp.ovid.com/ovidweb.cgi?T=JS&CSC=Y&NEWS=N&PAGE=fulltext&D=cctr&AN=CN-01884550 | Other diagnosis (not depression/MDD) |  |
| 569 , Isrctn, . Trialling the Active Brains programme to reduce cognitive impairment in older age groups. http://www.who.int/trialsearch/Trial2.aspx?TrialID=ISRCTN17349359. 2020. :https://ovidsp.ovid.com/ovidweb.cgi?T=JS&CSC=Y&NEWS=N&PAGE=fulltext&D=cctr&AN=CN-02172131 | Other diagnosis (not depression/MDD) |  |
| 572 , Smoktunowicz E, Lesnierowska M. Carlbring P. Andersson G. Cieslak R. Resource-Based Internet Intervention (Med-Stress) to Improve Well-Being Among Medical Professionals: randomized Controlled Trial. Journal of medical Internet research. . 23:e21445 , https://ovidsp.ovid.com/ovidweb.cgi?T=JS&CSC=Y&NEWS=N&PAGE=fulltext&D=cctr&AN=CN-02232813 | Other diagnosis (not depression/MDD) |  |
| 579 , de Graaf Le, Gerhards S. A. Evers S. M. Arntz A. Riper H. Severens J. L. Widdershoven G. Metsemakers J. F. Huibers M. J. Clinical and cost-effectiveness of computerised cognitive behavioural therapy for depression in primary care: design of a randomised trial. BMC public health. 2008. 8:https://ovidsp.ovid.com/ovidweb.cgi?T=JS&CSC=Y&NEWS=N&PAGE=fulltext&D=cctr&AN=CN-00649427 | Include this article |  |
| 579 , de Graaf Le, Gerhards S. A. Evers S. M. Arntz A. Riper H. Severens J. L. Widdershoven G. Metsemakers J. F. Huibers M. J. Clinical and cost-effectiveness of computerised cognitive behavioural therapy for depression in primary care: design of a randomised trial. BMC public health. 2008. 8:https://ovidsp.ovid.com/ovidweb.cgi?T=JS&CSC=Y&NEWS=N&PAGE=fulltext&D=cctr&AN=CN-00649427 |  | Inappropriate outcomes |
| 585 , van Spijker Ba, van Straten A. Kerkhof A. J. The effectiveness of a web-based self-help intervention to reduce suicidal thoughts: a randomized controlled trial. Trials. 2010. 11:https://ovidsp.ovid.com/ovidweb.cgi?T=JS&CSC=Y&NEWS=N&PAGE=fulltext&D=cctr&AN=CN-00742453 | Include this article |  |
|  |  | Other diagnosis (not depression/MDD) |
| 590 , Andrews G, Davies M. Titov N. Effectiveness randomized controlled trial of face to face versus Internet cognitive behaviour therapy for social phobia. Australian and New Zealand journal of psychiatry. . 45:337-340 , https://ovidsp.ovid.com/ovidweb.cgi?T=JS&CSC=Y&NEWS=N&PAGE=fulltext&D=cctr&AN=CN-00787500 | Other diagnosis (not depression/MDD) |  |
| 610 , Thorndike Fp, Ritterband L. M. Gonder-Frederick L. A. Lord H. R. Ingersoll K. S. Morin C. M. A randomized controlled trial of an internet intervention for adults with insomnia: effects on comorbid psychological and fatigue symptoms. Journal of clinical psychology. . 69:1078-1093 , https://ovidsp.ovid.com/ovidweb.cgi?T=JS&CSC=Y&NEWS=N&PAGE=fulltext&D=cctr&AN=CN-00989096 | Other diagnosis (not depression/MDD) |  |
| 615 , Schroder J, Bruckner K. Fischer A. Lindenau M. Kother U. Vettorazzi E. Moritz S. Efficacy of a psychological online intervention for depression in people with epilepsy: a randomized controlled trial. Epilepsia. . 55:2069-2076 , https://ovidsp.ovid.com/ovidweb.cgi?T=JS&CSC=Y&NEWS=N&PAGE=fulltext&D=cctr&AN=CN-01040037 | Include this article |  |
|  |  | Other diagnosis (not depression/MDD) |
| 616 , Geisner Im, Varvil-Weld L. Mittmann A. J. Mallett K. Turrisi R. Brief web-based intervention for college students with comorbid risky alcohol use and depressed mood: does it work and for whom?. Addictive behaviors. 2015. 42:36-43 , https://ovidsp.ovid.com/ovidweb.cgi?T=JS&CSC=Y&NEWS=N&PAGE=fulltext&D=cctr&AN=CN-01040278 | Include this article |  |
|  |  | Other diagnosis (not depression/MDD) |
| 620 , Van der Zanden R, Curie K. Van Londen M. Kramer J. Steen G. Cuijpers P. Web-based depression treatment: associations of clients' word use with adherence and outcome. Journal of affective disorders. 2014. 160:10-13 , https://ovidsp.ovid.com/ovidweb.cgi?T=JS&CSC=Y&NEWS=N&PAGE=fulltext&D=cctr&AN=CN-01049080 | Include this article |  |
|  |  | Inappropriate study design |
| 627 , Smith P, Scott R. Eshkevari E. Jatta F. Leigh E. Harris V. Robinson A. Abeles P. Proudfoot J. Verduyn C. Yule W. Computerised CBT for depressed adolescents: randomised controlled trial. Behaviour research and therapy. 2015. 73:104-110 , https://ovidsp.ovid.com/ovidweb.cgi?T=JS&CSC=Y&NEWS=N&PAGE=fulltext&D=cctr&AN=CN-01090136 | Inappropriate study design |  |
| 628 , Morie Kp, Nich C. Hunkele K. Potenza M. N. Carroll K. M. Alexithymia level and response to computer-based training in cognitive behavioral therapy among cocaine-dependent methadone maintained individuals. Drug and alcohol dependence. 2015. 152:157-163 , https://ovidsp.ovid.com/ovidweb.cgi?T=JS&CSC=Y&NEWS=N&PAGE=fulltext&D=cctr&AN=CN-01100146 | Other diagnosis (not depression/MDD) |  |
| 632 , Almeida Op, MacLeod C. Ford A. Grafton B. Hirani V. Glance D. Holmes E. Cognitive bias modification to prevent depression (COPE): study protocol for a randomised controlled trial. Trials. 2014. 15:https://ovidsp.ovid.com/ovidweb.cgi?T=JS&CSC=Y&NEWS=N&PAGE=fulltext&D=cctr&AN=CN-01115266 | Other diagnosis (not depression/MDD) |  |
| 643 , Pugh Ne, Hadjistavropoulos H. D. Dirkse D. A Randomised Controlled Trial of Therapist-Assisted, Internet-Delivered Cognitive Behavior Therapy for Women with Maternal Depression. PloS one. . 11:e0149186 , https://ovidsp.ovid.com/ovidweb.cgi?T=JS&CSC=Y&NEWS=N&PAGE=fulltext&D=cctr&AN=CN-01168154 | Other diagnosis (not depression/MDD) |  |
| 657 , Lewandowski Ke, Sperry S. H. Ongur D. Cohen B. M. Norris L. A. Keshavan M. S. Cognitive remediation versus active computer control in bipolar disorder with psychosis: study protocol for a randomized controlled trial. Trials. . 17:136 , https://ovidsp.ovid.com/ovidweb.cgi?T=JS&CSC=Y&NEWS=N&PAGE=fulltext&D=cctr&AN=CN-01264163 | Other diagnosis (not depression/MDD) |  |
| 673 , Ip P, Chim D. Chan K. L. Li T. M. Ho F. K. Van Voorhees B. W. Tiwari A. Tsang A. Chan C. W. Ho M. Tso W. Wong W. H. Effectiveness of a culturally attuned Internet-based depression prevention program for Chinese adolescents: a randomized controlled trial. Depression and anxiety. . 33:1123-1131 , https://ovidsp.ovid.com/ovidweb.cgi?T=JS&CSC=Y&NEWS=N&PAGE=fulltext&D=cctr&AN=CN-01444344 | Inappropriate study design |  |
| 674 , Schaub Mp, Blankers M. Lehr D. Boss L. Riper H. Dekker J. Goudriaan A. E. Maier L. J. Haug S. Amann M. Dey M. Wenger A. Ebert D. D. Efficacy of an internet-based self-help intervention to reduce co-occurring alcohol misuse and depression symptoms in adults: study protocol of a three-arm randomised controlled trial. BMJ open. . 6:e011457 , https://ovidsp.ovid.com/ovidweb.cgi?T=JS&CSC=Y&NEWS=N&PAGE=fulltext&D=cctr&AN=CN-01444807 | Include this article |  |
|  |  | Other diagnosis (not depression/MDD) |
| 677 , Brodbeck J, Berger T. Znoj H. J. An internet-based self-help intervention for older adults after marital bereavement, separation or divorce: study protocol for a randomized controlled trial. Trials. . 18:21 , https://ovidsp.ovid.com/ovidweb.cgi?T=JS&CSC=Y&NEWS=N&PAGE=fulltext&D=cctr&AN=CN-01454936 | Other diagnosis (not depression/MDD) |  |
| 697 , Brodbeck J, Berger T. Biesold N. Rockstroh F. Znoj H. J. Evaluation of a guided internet-based self-help intervention for older adults after spousal bereavement or separation/divorce: a randomised controlled trial. Journal of affective disorders. 2019. 252:440-449 , https://ovidsp.ovid.com/ovidweb.cgi?T=JS&CSC=Y&NEWS=N&PAGE=fulltext&D=cctr&AN=CN-01937139 | Other diagnosis (not depression/MDD) |  |
| 705 , Ford-Gilboe M, Varcoe C. Scott-Storey K. Perrin N. Wuest J. Wathen C. N. Case J. Glass N. Longitudinal impacts of an online safety and health intervention for women experiencing intimate partner violence: randomized controlled trial. BMC public health. . 20:260 , https://ovidsp.ovid.com/ovidweb.cgi?T=JS&CSC=Y&NEWS=N&PAGE=fulltext&D=cctr&AN=CN-02097844 | Other diagnosis (not depression/MDD) |  |
| 710 , Evans K, Valencia Lcsw L. Cochran C. D. Cochran M. Outcomes and feasibility from the first TMS specific web-based intervention. Brain stimulation. . 13:1861- , https://ovidsp.ovid.com/ovidweb.cgi?T=JS&CSC=Y&NEWS=N&PAGE=fulltext&D=cctr&AN=CN-02212131 | Include this article |  |
|  |  | Inappropriate study design |
| 718 , Nct, . Pilot Testing a Mobile App to Designed to Increase Physical Activity Among Pregnant and Postpartum Women. https://clinicaltrials.gov/show/NCT04480931. 2020. :https://ovidsp.ovid.com/ovidweb.cgi?T=JS&CSC=Y&NEWS=N&PAGE=fulltext&D=cctr&AN=CN-02180942 | Other diagnosis (not depression/MDD) |  |
| 719 , Nct, . A CBT-based Mobile Intervention as First Line Treatment for Adolescent Depression During COVID-19. https://clinicaltrials.gov/show/NCT04524598. 2020. :https://ovidsp.ovid.com/ovidweb.cgi?T=JS&CSC=Y&NEWS=N&PAGE=fulltext&D=cctr&AN=CN-02181129 | Inappropriate study design |  |
| 720 , Morimoto Ss, Altizer R. A. Gunning F. M. Hu W. Liu J. Cote S. E. Nitis J. Alexopoulos G. S. Targeting Cognitive Control Deficits With Neuroplasticity-Based Computerized Cognitive Remediation in Patients With Geriatric Major Depression: a Randomized, Double-Blind, Controlled Trial. American journal of geriatric psychiatry. 2020. :https://ovidsp.ovid.com/ovidweb.cgi?T=JS&CSC=Y&NEWS=N&PAGE=fulltext&D=cctr&AN=CN-02142275 | Include this article |  |
|  |  | Inappropriate outcomes |
| 721 , . A randomized, double-blind, controlled, 6-week trial to assess a novel digital intervention designed to improve cognitive dysfunction as adjunct therapy to antidepressant medication in adults with major depressive disorder. Neuropsychopharmacology. 2019. 44:https://ovidsp.ovid.com/ovidweb.cgi?T=JS&CSC=Y&NEWS=N&PAGE=fulltext&D=cctr&AN=CN-02147397 |  | Inappropriate intervention |
|  | Include this article |  |
| 722 , Nct, . tDCS for Impulsivity and Compulsivity in Obesity. https://clinicaltrials.gov/show/NCT04405089. 2020. :https://ovidsp.ovid.com/ovidweb.cgi?T=JS&CSC=Y&NEWS=N&PAGE=fulltext&D=cctr&AN=CN-02124966 | Other diagnosis (not depression/MDD) |  |
| 723 , Actrn, . Preventing relapse of major depressive disorder in youth: randomised Controlled Trial of a novel mindfulness-based cognitive online social therapy. http://www.who.int/trialsearch/Trial2.aspx?TrialID=ACTRN12619001412123. 2019. :https://ovidsp.ovid.com/ovidweb.cgi?T=JS&CSC=Y&NEWS=N&PAGE=fulltext&D=cctr&AN=CN-02064859 | Include this article |  |
|  |  | Inappropriate study design |
| 724 , Isrctn, . Dementia prevention for older people suffering from mild cognitive impairment using digital brain training tools. 2020. :https://ovidsp.ovid.com/ovidweb.cgi?T=JS&CSC=Y&NEWS=N&PAGE=fulltext&D=cctr&AN=CN-02084471 | Other diagnosis (not depression/MDD) |  |
| 725 , Isrctn, . Mobile mindfulness for asthma. http://www.who.int/trialsearch/Trial2.aspx?TrialID=ISRCTN52212323. 2017. :https://ovidsp.ovid.com/ovidweb.cgi?T=JS&CSC=Y&NEWS=N&PAGE=fulltext&D=cctr&AN=CN-01866995 | Other diagnosis (not depression/MDD) |  |
| 726 , Actrn, . Enhancing Social Functioning in Young People at Ultra High Risk for Psychosis: randomised Controlled Trial of a Novel Strengths-based Online Social Therapy. http://www.who.int/trialsearch/Trial2.aspx?TrialID=ACTRN12619001411134. 2019. :https://ovidsp.ovid.com/ovidweb.cgi?T=JS&CSC=Y&NEWS=N&PAGE=fulltext&D=cctr&AN=CN-02064858 | Other diagnosis (not depression/MDD) |  |
| 727 , Nct, . Effectiveness of Internet-Based Cognitive Behavioral Therapy for Treating Mothers With Depression. https://clinicaltrials.gov/show/NCT00601393. 2008. :https://ovidsp.ovid.com/ovidweb.cgi?T=JS&CSC=Y&NEWS=N&PAGE=fulltext&D=cctr&AN=CN-02019058 | Include this article |  |
|  |  | Inappropriate outcomes |
| 728 , Nct, . Technology-Enhanced Peer Navigation to Improve IDUs' Engagement in HIV Care. https://clinicaltrials.gov/show/NCT01941108. 2013. :https://ovidsp.ovid.com/ovidweb.cgi?T=JS&CSC=Y&NEWS=N&PAGE=fulltext&D=cctr&AN=CN-02032054 | Other diagnosis (not depression/MDD) |  |
| 729 , Nct, . Evaluation of a Program for Eating Disorders That Combines Cognitive-behavioral Therapy With Online Psychological Treatment. https://clinicaltrials.gov/show/NCT03197519. 2017. :https://ovidsp.ovid.com/ovidweb.cgi?T=JS&CSC=Y&NEWS=N&PAGE=fulltext&D=cctr&AN=CN-02042348 | Other diagnosis (not depression/MDD) |  |
| 730 , Nct, . RCT of an Internet-based CBT Program for Sexuality and Intimacy Problems in Women Treated for Breast Cancer. https://clinicaltrials.gov/show/NCT02091765. 2014. :https://ovidsp.ovid.com/ovidweb.cgi?T=JS&CSC=Y&NEWS=N&PAGE=fulltext&D=cctr&AN=CN-02046489 | Other diagnosis (not depression/MDD) |  |
| 731 , Actrn, . A Randomised Controlled Trial (RCT) of AdaptHIV, an internet Cognitive Behavioural Therapy (iCBT) program for the treatment of depression and anxiety in men who have sex with men (MSM) who are living with HIV. http://www.who.int/trialsearch/Trial2.aspx?TrialID=ACTRN12617001549314. 2017. :https://ovidsp.ovid.com/ovidweb.cgi?T=JS&CSC=Y&NEWS=N&PAGE=fulltext&D=cctr&AN=CN-01886010 | Include this article |  |
|  |  | Other diagnosis (not depression/MDD) |
| 732 , Isrctn, . REDUCE Programme WS4: rEviewing long term anti-DEpressant Use by Careful monitoring in Everyday practice. http://www.who.int/trialsearch/Trial2.aspx?TrialID=ISRCTN15036829. 2018. :https://ovidsp.ovid.com/ovidweb.cgi?T=JS&CSC=Y&NEWS=N&PAGE=fulltext&D=cctr&AN=CN-01905893 | Include this article |  |
| 732 , Isrctn, . REDUCE Programme WS4: rEviewing long term anti-DEpressant Use by Careful monitoring in Everyday practice. http://www.who.int/trialsearch/Trial2.aspx?TrialID=ISRCTN15036829. 2018. :https://ovidsp.ovid.com/ovidweb.cgi?T=JS&CSC=Y&NEWS=N&PAGE=fulltext&D=cctr&AN=CN-01905893 |  | Inappropriate intervention |
| 733 , Isrctn, . The Active Brains study - feasibility trial. http://www.who.int/trialsearch/Trial2.aspx?TrialID=ISRCTN23758980. 2018. :https://ovidsp.ovid.com/ovidweb.cgi?T=JS&CSC=Y&NEWS=N&PAGE=fulltext&D=cctr&AN=CN-01909462 | Other diagnosis (not depression/MDD) |  |
| 734 , Isrctn, . SupportBack 2: supporting self-management of low back pain with an internet intervention. http://www.who.int/trialsearch/Trial2.aspx?TrialID=ISRCTN14736486. 2018. :https://ovidsp.ovid.com/ovidweb.cgi?T=JS&CSC=Y&NEWS=N&PAGE=fulltext&D=cctr&AN=CN-01910116 | Other diagnosis (not depression/MDD) |  |
| 735 , Nct, . Repetitive TMS & Cognitive Training in Adults With Schizophrenia. https://clinicaltrials.gov/show/NCT03741751. 2018. :https://ovidsp.ovid.com/ovidweb.cgi?T=JS&CSC=Y&NEWS=N&PAGE=fulltext&D=cctr&AN=CN-01918382 | Other diagnosis (not depression/MDD) |  |
| 736 , Actrn, . Cool Kids Taking Control Online Program for Children Experiencing Bullying and Anxiety. http://www.who.int/trialsearch/Trial2.aspx?TrialID=ACTRN12618001793202. 2018. :https://ovidsp.ovid.com/ovidweb.cgi?T=JS&CSC=Y&NEWS=N&PAGE=fulltext&D=cctr&AN=CN-01946970 | Other diagnosis (not depression/MDD) |  |
| 737 , Nct, . A Study on Better Cognitive Functioning Through Braintraining on the Internet. https://clinicaltrials.gov/show/NCT04006756. 2019. :https://ovidsp.ovid.com/ovidweb.cgi?T=JS&CSC=Y&NEWS=N&PAGE=fulltext&D=cctr&AN=CN-01953004 | Other diagnosis (not depression/MDD) |  |
| 738 , Nct, . Computerized Cognitive Training in Neurodegenerative Diseases (NDD2019). https://clinicaltrials.gov/show/NCT04111640. 2019. :https://ovidsp.ovid.com/ovidweb.cgi?T=JS&CSC=Y&NEWS=N&PAGE=fulltext&D=cctr&AN=CN-01992453 | Other diagnosis (not depression/MDD) |  |
| 739 , Nct, . Non-Pharmacological Treatments and Cognitive Impairment (NPT-CI2019). https://clinicaltrials.gov/show/NCT04118686. 2019. :https://ovidsp.ovid.com/ovidweb.cgi?T=JS&CSC=Y&NEWS=N&PAGE=fulltext&D=cctr&AN=CN-01992646 | Other diagnosis (not depression/MDD) |  |
| 740 , Nct, . An Online Self-help Intervention for Prevention of Depression in Primary Care. https://clinicaltrials.gov/show/NCT04139785. 2019. :https://ovidsp.ovid.com/ovidweb.cgi?T=JS&CSC=Y&NEWS=N&PAGE=fulltext&D=cctr&AN=CN-02001381 | Include this article |  |
|  |  | Other diagnosis (not depression/MDD) |
| 742 , Nct, . Development and Testing of a Behavioral Activation Mobile Therapy for Elevated Depressive Systems. https://clinicaltrials.gov/show/NCT02498132. 2015. :https://ovidsp.ovid.com/ovidweb.cgi?T=JS&CSC=Y&NEWS=N&PAGE=fulltext&D=cctr&AN=CN-01490982 | Include this article |  |
|  |  | Other diagnosis (not depression/MDD) |
| 743 , Nct, . The Impact of Piano Training on Cognitive Performance and Psychosocial Well-Being in Older Adults. https://clinicaltrials.gov/show/NCT02564601. 2015. :https://ovidsp.ovid.com/ovidweb.cgi?T=JS&CSC=Y&NEWS=N&PAGE=fulltext&D=cctr&AN=CN-01492642 | Other diagnosis (not depression/MDD) |  |
| 744 , Nct, . Pilot RCT of Pos4Health for Nonadherent HIV+ Substance Users. https://clinicaltrials.gov/show/NCT02837250. 2016. :https://ovidsp.ovid.com/ovidweb.cgi?T=JS&CSC=Y&NEWS=N&PAGE=fulltext&D=cctr&AN=CN-01506897 | Other diagnosis (not depression/MDD) |  |
| 745 , Nct, . Manualized Group Cognitive-behavioral Therapy for Social Anxiety in First Episode Psychosis. https://clinicaltrials.gov/show/NCT02294409. 2014. :https://ovidsp.ovid.com/ovidweb.cgi?T=JS&CSC=Y&NEWS=N&PAGE=fulltext&D=cctr&AN=CN-01550319 | Other diagnosis (not depression/MDD) |  |
| 746 , Nct, . Cognitive Training in Patients With Trichotillomania (Hair-pulling Disorder). https://clinicaltrials.gov/show/NCT02794753. 2016. :https://ovidsp.ovid.com/ovidweb.cgi?T=JS&CSC=Y&NEWS=N&PAGE=fulltext&D=cctr&AN=CN-01558868 | Other diagnosis (not depression/MDD) |  |
| 747 , Nct, . Presential Vs Online Group-based Psychosocial Treatment for Breast Cancer Survivors. https://clinicaltrials.gov/show/NCT03010371. 2016. :https://ovidsp.ovid.com/ovidweb.cgi?T=JS&CSC=Y&NEWS=N&PAGE=fulltext&D=cctr&AN=CN-01561027 | Other diagnosis (not depression/MDD) |  |
| 748 , Nct, . Electronic Bridge to Mental Health for College Students. https://clinicaltrials.gov/show/NCT03380117. 2017. :https://ovidsp.ovid.com/ovidweb.cgi?T=JS&CSC=Y&NEWS=N&PAGE=fulltext&D=cctr&AN=CN-01566895 | Other diagnosis (not depression/MDD) |  |
| 749 , Nct, . Dementia Caregiver Chronic Grief Management: a Live Online Video Intervention. https://clinicaltrials.gov/show/NCT03593070. 2018. :https://ovidsp.ovid.com/ovidweb.cgi?T=JS&CSC=Y&NEWS=N&PAGE=fulltext&D=cctr&AN=CN-01625744 | Other diagnosis (not depression/MDD) |  |
| 750 , Isrctn, . Effectiveness of "home-but not alone" mobile-health application on parental outcomes. http://www.who.int/trialsearch/Trial2.aspx?TrialID=ISRCTN99092313. 2016. :https://ovidsp.ovid.com/ovidweb.cgi?T=JS&CSC=Y&NEWS=N&PAGE=fulltext&D=cctr&AN=CN-01798602 | Other diagnosis (not depression/MDD) |  |
| 751 , Isrctn, . An initial study of an online self-help programme for individuals seeking employment. http://www.who.int/trialsearch/Trial2.aspx?TrialID=ISRCTN10316077. 2014. :https://ovidsp.ovid.com/ovidweb.cgi?T=JS&CSC=Y&NEWS=N&PAGE=fulltext&D=cctr&AN=CN-01801094 | Other diagnosis (not depression/MDD) |  |
| 752 , Isrctn, . Indicated web-based prevention of mental disorders in undergraduate university students. http://www.who.int/trialsearch/Trial2.aspx?TrialID=ISRCTN15570935. 2016. :https://ovidsp.ovid.com/ovidweb.cgi?T=JS&CSC=Y&NEWS=N&PAGE=fulltext&D=cctr&AN=CN-01803119 | Other diagnosis (not depression/MDD) |  |
| 753 , Isrctn, . Will an online life skills package be helpful for individuals with bulimia nervosa?. http://www.who.int/trialsearch/Trial2.aspx?TrialID=ISRCTN41034162. 2009. :https://ovidsp.ovid.com/ovidweb.cgi?T=JS&CSC=Y&NEWS=N&PAGE=fulltext&D=cctr&AN=CN-01820055 | Other diagnosis (not depression/MDD) |  |
| 754 , Isrctn, . Investigating the effects of a self-guided, internet-based self-help program for people with anxiety disorders. http://www.who.int/trialsearch/Trial2.aspx?TrialID=ISRCTN81412545. 2016. :https://ovidsp.ovid.com/ovidweb.cgi?T=JS&CSC=Y&NEWS=N&PAGE=fulltext&D=cctr&AN=CN-01822159 | Other diagnosis (not depression/MDD) |  |
| 755 , Isrctn, . Evidence-based information and communications technology tools for weight loss maintenance. http://www.who.int/trialsearch/Trial2.aspx?TrialID=ISRCTN88405328. 2016. :https://ovidsp.ovid.com/ovidweb.cgi?T=JS&CSC=Y&NEWS=N&PAGE=fulltext&D=cctr&AN=CN-01837858 | Other diagnosis (not depression/MDD) |  |
| 762 , Foster C, Grimmett C. May C. M. Ewings S. Myall M. Hulme C. Smith P. Powers C. Calman L. Armes J. Breckons M. Corner J. Fenlon D. Lennan E. May C. R. Morris C. Ream E. Turner L. Yardley L. Richardson A. Restore: testing feasibility and acceptability of an online intervention to support self-management of cancer-related fatigue in a multi-centre proof of concept randomised controlled trial. Psycho oncology. 2014. 23:https://ovidsp.ovid.com/ovidweb.cgi?T=JS&CSC=Y&NEWS=N&PAGE=fulltext&D=cctr&AN=CN-01023326 | Other diagnosis (not depression/MDD) |  |
| 766 , Devanand Dp, Reynolds C. F. Pelton G. H. Doraiswamy P. M. Mulsant B. H. Morimoto S. S. Alexopoulos G. S. Gunning F. Wexler B. E. Therapeutic strategies in depression with cognitive impairment. American journal of geriatric psychiatry. . 26:S18-S19 , https://ovidsp.ovid.com/ovidweb.cgi?T=JS&CSC=Y&NEWS=N&PAGE=fulltext&D=cctr&AN=CN-01570843 | Inappropriate intervention |  |
| 767 , Sander L, Paganini S. Lin J. Schlicker S. Ebert D. D. Buntrock C. Baumeister H. Effectiveness and cost-effectiveness of a guided Internet- and mobile-based intervention for the indicated prevention of major depression in patients with chronic back pain-study protocol of the PROD-BP multicenter pragmatic RCT. BMC psychiatry. 2017. 17:https://ovidsp.ovid.com/ovidweb.cgi?T=JS&CSC=Y&NEWS=N&PAGE=fulltext&D=cctr&AN=CN-01331129 | Include this article |  |
|  |  | Other diagnosis (not depression/MDD) |
| 768 , de Beurs Dp, de Groot M. H. Bosmans J. E. de Keijser J. Mokkenstorm J. Verwey B. van Duijn E. de Winter R. F. P. Kerkhof Ajfm . Reducing patients' suicide ideation through training mental health teams in the application of the Dutch multidisciplinary practice guideline on assessment and treatment of suicidal behavior: study protocol of a randomized controlled trial. Trials. 2013. 14:https://ovidsp.ovid.com/ovidweb.cgi?T=JS&CSC=Y&NEWS=N&PAGE=fulltext&D=cctr&AN=CN-00915144 | Inappropriate intervention |  |
| 769 , Lillevoll Kr, Vangberg H. C. B. Griffiths K. M. Waterloo K. Eisemann M. R. Uptake and adherence of a self-directed internet-based mental health intervention with tailored e-mail reminders in senior high schools in Norway. BMC psychiatry. 2014. 14:https://ovidsp.ovid.com/ovidweb.cgi?T=JS&CSC=Y&NEWS=N&PAGE=fulltext&D=cctr&AN=CN-00979113 | Inappropriate study design |  |
| 774 , Imamura K, Kawakami N. Furukawa T. A. Matsuyama Y. Shimazu A. Umanodan R. Kawakami S. Kasai K. Does Internet-based cognitive behavioral therapy (iCBT) prevent major depressive episode for workers? A 12-month follow-up of a randomized controlled trial. Psychological medicine. . 45:1907-17 , https://ovidsp.ovid.com/ovidweb.cgi?T=JS&CSC=Y&NEWS=N&PAGE=fulltext&D=cctr&AN=CN-01209005 | Other diagnosis (not depression/MDD) |  |
| 775 , Rosso I, Olson E. Killgore W. D. S. Fukunaga R. Webb C. Rauch S. A randomized trial of internet-based cognitive behavioral therapy for major depressive disorder. Neuropsychopharmacology. . Vol.40, pp.S170-S171, CONFERENCE START:2015 Dec 6 CONFERENCE END: 2015 Dec 10 , https://ovidsp.ovid.com/ovidweb.cgi?T=JS&CSC=Y&NEWS=N&PAGE=fulltext&D=cctr&AN=CN-01163265 |  | Inappropriate outcomes |
|  | Include this article |  |
| 776 , Centre for, Reviews,Dissemination, . A systematic review of interactive computer-assisted technology in diabetes care: interactive information technology in diabetes care (Structured abstract). Database of Abstracts of Reviews of Effects. 2015. 2:2 , https://ovidsp.ovid.com/ovidweb.cgi?T=JS&CSC=Y&NEWS=N&PAGE=fulltext&D=dare&AN=00125498-100000000-19627 | Inappropriate study design |  |
| 777 , Centre for, Reviews,Dissemination, . The efficacy of internet interventions for depression and anxiety disorders: a review of randomised controlled trials (Structured abstract). Database of Abstracts of Reviews of Effects. 2015. 2:2 , https://ovidsp.ovid.com/ovidweb.cgi?T=JS&CSC=Y&NEWS=N&PAGE=fulltext&D=dare&AN=00125498-100000000-25223 | Inappropriate study design |  |
| 778 , Centre for, Reviews,Dissemination, . Exploratory economic analyses of two primary care mental health projects: implications for sustainability (Structured abstract). NHS Economic Evaluation Database. 2015. :https://ovidsp.ovid.com/ovidweb.cgi?T=JS&CSC=Y&NEWS=N&PAGE=fulltext&D=cleed&AN=NHSEED-22005001906 | Other diagnosis (not depression/MDD) |  |
| 783 , H Christensen, KM Griffiths, AF Jorm . Delivering interventions for depression by using the internet: randomised controlled trial. BMJ (Clinical research ed.). 2004. 328: 10.1136/bmj.37945.566632.EE 14742346 | Include this article |  |
|  |  | Other diagnosis (not depression/MDD) |
| 784 , LE de Graaf, SA Gerhards, A Arntz, H Riper, JF Metsemakers, SM Evers, JL Severens, G Widdershoven, MJ Huibers . Clinical effectiveness of online computerised cognitive-behavioural therapy without support for depression in primary care: randomised trial. The British journal of psychiatry : the journal of mental science. 2009. 195: 10.1192/bjp.bp.108.054429 19567900 | Include this article |  |
|  |  | Inappropriate outcomes |
| 785 , L Farrer, H Christensen, KM Griffiths, A Mackinnon . Internet-based CBT for depression with and without telephone tracking in a national helpline: randomised controlled trial. PloS one. 2011. 6: 10.1371/journal.pone.0028099 22140514 | Include this article |  |
|  |  | Other diagnosis (not depression/MDD) |
| 16938144 , H Christensen, KM Griffiths, AJ Mackinnon, K Brittliffe . Online randomized controlled trial of brief and full cognitive behaviour therapy for depression. Psychological medicine. 2006. 36: 10.1017/S0033291706008695 16938144 | Include this article |  |
|  |  | Inappropriate study design |
| 18245031 , A Mackinnon, KM Griffiths, H Christensen . Comparative randomised trial of online cognitive-behavioural therapy and an information website for depression: 12-month outcomes. The British journal of psychiatry : the journal of mental science. 2008. 192: 10.1192/bjp.bp.106.032078 18245031 | Include this article |  |
|  |  | Other diagnosis (not depression/MDD) |
| 23451231 , AD Williams, G Andrews . The effectiveness of Internet cognitive behavioural therapy (iCBT) for depression in primary care: a quality assurance study. PloS one. 2013. 8: 10.1371/journal.pone.0057447 23451231 | Include this article |  |
|  |  | Inappropriate study design |
| 34283039 , . Pilot randomised controlled trial of a real time, personalised, transdiagnostic smartphone intervention targeting repetitive negative thinking in young people with depression and anxiety. 2021. : | Inappropriate study design |  |
| 34283040 , . Enhancing Processing Speed and Executive Functioning in Depressed Older Adults With Computerized Cognitive Training. 2021. : | Inappropriate intervention |  |
| 34283041 , . Supporting employees with insomnia and emotional regulation problems. 2021. : | Other diagnosis (not depression/MDD) |  |
| 34283042 , . Digital health interventions based on app for cell phones or e-book for health promotion. 2021. : | Other diagnosis (not depression/MDD) |  |
| 34283043 , . Reducing stress in the workplace using a digital intervention designed to improve employee wellbeing and help them stay engaged and productive in work. 2021. : | Other diagnosis (not depression/MDD) |  |
| 34283044 , . Testing an internet intervention for preventing and reducing perinatal depressive symptoms and promoting subjective well-being. 2021. : | Other diagnosis (not depression/MDD) |  |
| 34283045 , . Web-based Self-help Intervention Promoting Mental Health in Adolescents. 2021. : | Inappropriate study design |  |
| 34283046 , . CANreduce 3.0 - a web-based self-help program for reducing cannabis use that explores the effectiveness of mindfulness and cognitive behavioral therapy approaches. 2021. : | Inappropriate intervention |  |
| 34283047 , . BrainFit-Nutrition: Intervention study for people suffering from mild cognitive impairment using computerized cognitive training tools and a nutrition intervention. 2021. : | Other diagnosis (not depression/MDD) |  |
| 34283048 , . OurRelationship for Veteran Couples. 2021. : | Other diagnosis (not depression/MDD) |  |
| 34283049 , . Clinical Assessment of the Flash - Eye Movement Desensitization and Reprocessing. 2022. : | Inappropriate intervention |  |
| 34283050 , . Promoting Self-Management of Breast and Nipple Pain With Technology (PROMPT) for Breastfeeding Women Study. 2022. : | Other diagnosis (not depression/MDD) |  |
| 34283051 , Ahlers, J. Cannabis Use in Adults Who Screen Positive for Attention Deficit/Hyperactivity Disorder: CANreduce 2.0 Randomized Controlled Trial Subgroup Analysis. Journal of Medical Internet Research. 2022. : | Other diagnosis (not depression/MDD) |  |
| 34283052 , Alavi, N. Developing and Implementing a Web-Based Psychotherapy Program to Address Mental Health Challenges Among Patients Receiving Oncologic and Palliative Care: Protocol for an Open-Label Randomized Controlled Trial. JMIR Research Protocols. 2021. : | Other diagnosis (not depression/MDD) |  |
| 34283053 , Allen, K. D. Pilot study of an internet-based pain coping skills training program for patients with systemic Lupus Erythematosus. BMC Rheumatology. 2021. : | Other diagnosis (not depression/MDD) |  |
| 34283054 , Alvarez-Jimenez, M. The Horyzons project: a randomized controlled trial of a novel online social therapy to maintain treatment effects from specialist first-episode psychosis services. World Psychiatry. . : | Other diagnosis (not depression/MDD) |  |
| 34283055 , Araya, R. Effect of a Digital Intervention on Depressive Symptoms in Patients with Comorbid Hypertension or Diabetes in Brazil and Peru: Two Randomized Clinical Trials. JAMA - Journal of the American Medical Association. 2021. : | Include this article |  |
|  |  | Other diagnosis (not depression/MDD) |
| 34283056 , Arnobit, C. I. Recruiting cancer survivors to a mobile mindfulness intervention in the united states: Exploring online and face-to-face recruitment strategies. International Journal of Environmental Research and Public Health. 2021. : | Other diagnosis (not depression/MDD) |  |
| 34283057 , Bade, B. C. Randomized trial of physical activity on quality of life and lung cancer biomarkers in patients with advanced stage lung cancer: a pilot study. BMC Cancer. . : | Other diagnosis (not depression/MDD) |  |
| 34283058 , Barkham, M. Person-centred experiential therapy versus cognitive behavioural therapy delivered in the English Improving Access to Psychological Therapies service for the treatment of moderate or severe depression (PRaCTICED): a pragmatic, randomised, non-inferiority trial. The Lancet Psychiatry. 2021. : | Inappropriate intervention |  |
| 34283059 , Batterham, P. J. Efficacy of a Transdiagnostic Self-Help Internet Intervention for Reducing Depression, Anxiety, and Suicidal Ideation in Adults: randomized Controlled Trial. Journal of Medical Internet Research. . : | Other diagnosis (not depression/MDD) |  |
| 34283061 , Bauerle, A. Evaluation of the E-Mental Health Mindfulness-Based and Skills-Based CoPE It Intervention to Reduce Psychological Distress in Times of COVID-19: Results of a Bicentre Longitudinal Study. Frontiers in psychiatry Frontiers Research Foundation. 2021. : | Other diagnosis (not depression/MDD) |  |
| 34283062 , Baumeister, H. Effectiveness of a Guided Internet- And Mobile-Based Intervention for Patients with Chronic Back Pain and Depression (WARD-BP): A Multicenter, Pragmatic Randomized Controlled Trial. Psychotherapy and Psychosomatics. 2021. : | Include this article |  |
|  |  | Other diagnosis (not depression/MDD) |
| 34283063 , Baumgartner, C. Take Care of You - Efficacy of integrated, minimal-guidance, internet-based self-help for reducing co-occurring alcohol misuse and depression symptoms in adults: Results of a three-arm randomized controlled trial. Drug and Alcohol Dependence. 2021. : | Inappropriate intervention |  |
| 34283064 , Ben-Zeev, D. A smartphone intervention for people with serious mental illness: Fully remote randomized controlled trial of CORE. Journal of Medical Internet Research. 2021. : | Other diagnosis (not depression/MDD) |  |
| 34283065 , Bendig, E. ACTonDiabetes - A guided psychological internet intervention based on Acceptance and Commitment Therapy (ACT) for adults living with type 1 or 2 diabetes: Results of a randomised controlled feasibility trial. BMJ Open. 2021. : | Other diagnosis (not depression/MDD) |  |
| 34283066 , Beukes, E. W. Internet-Based Audiologist-Guided Cognitive Behavioral Therapy for Tinnitus: Randomized Controlled Trial. Journal of Medical Internet Research. 2022. : | Other diagnosis (not depression/MDD) |  |
| 34283067 , Beukes, E. W. Internet-based cognitive behavioural therapy for tinnitus in Spanish: a global feasibility trial. International journal of audiology. 2021:1-10. : | Other diagnosis (not depression/MDD) |  |
| 34283068 , Bowers, H. A digital intervention for primary care practitioners to support antidepressant discontinuation (advisor for health professionals): Development study. Journal of Medical Internet Research. 2021. : | Other diagnosis (not depression/MDD) |  |
| 34283069 , Braun, L. Are guided internet-based interventions for the indicated prevention of depression in green professions effective in the long run? Longitudinal analysis of the 6- and 12-month follow-up of a pragmatic randomized controlled trial (PROD-A). Internet Interventions. 2021. : | Include this article |  |
|  |  | Inappropriate study design |
| 34283070 , Brazeau, B. W. Augmenting an online self-directed intervention for gambling disorder with a single motivational interview: study protocol for a randomized controlled trial. Trials. 2021. : | Other diagnosis (not depression/MDD) |  |
| 34283071 , Bretschneider, M. P. Impact of a Digital Lifestyle Intervention on Diabetes Self-Management: A Pilot Study. Nutrients. 2022. : | Other diagnosis (not depression/MDD) |  |
| 34283072 , Brog, N. A. Effects of an internet-based self-help intervention for psychological distress due to COVID-19: Results of a randomized controlled trial. Internet Interventions. 2022. : | Other diagnosis (not depression/MDD) |  |
| 34283073 , Browne, J. Engagement with a digital therapeutic for smoking cessation designed for persons with psychiatric illness fully mediates smoking outcomes in a pilot randomized controlled trial. Translational Behavioral Medicine. 2021. : | Other diagnosis (not depression/MDD) |  |
| 34283076 , Carullo, P. C. The impact of a smartphone meditation application on anesthesia trainee well-being. Journal of Clinical Anesthesia. 2021. : | Other diagnosis (not depression/MDD) |  |
| 34283077 , Castilla, D. Ecological momentary intervention to enhance emotion regulation in healthcare workers via smartphone: a randomized controlled trial protocol. BMC Psychiatry. 2022. : | Other diagnosis (not depression/MDD) |  |
| 34283078 , Centre for, R. A systematic review of interactive computer-assisted technology in diabetes care: interactive information technology in diabetes care (Structured abstract). Database of Abstracts of Reviews of Effects. 2015. : | Other diagnosis (not depression/MDD) |  |
| 34283079 , Centre for, R. The efficacy of internet interventions for depression and anxiety disorders: a review of randomised controlled trials (Structured abstract). Database of Abstracts of Reviews of Effects. 2015. : | Inappropriate study design |  |
| 34283080 , Colomer-Carbonell, A. Study protocol for a randomised, double-blinded, placebo-controlled phase III trial examining the add-on efficacy, cost-utility and neurobiological effects of low-dose naltrexone (LDN) in patients with fibromyalgia (INNOVA study). BMJ Open. 2022. : | Other diagnosis (not depression/MDD) |  |
| 34283081 , Costa, F. Telerehabilitation of acute musculoskeletal multi-disorders: prospective, single-arm, interventional study. BMC Musculoskeletal Disorders. 2022. : | Other diagnosis (not depression/MDD) |  |
| 34283082 , De Kock, J. H. Brief Digital Interventions to Support the Psychological Well-being of NHS Staff During the COVID-19 Pandemic: 3-Arm Pilot Randomized Controlled Trial. JMIR Mental Health. 2022. : | Other diagnosis (not depression/MDD) |  |
| 34283083 , Domhardt, M. Mobile-based interventions for common mental disorders in youth: a systematic evaluation of pediatric health apps. Child and Adolescent Psychiatry and Mental Health. 2021. : | Inappropriate study design |  |
| 34283084 , Dreyer, R. P. PreScription DigitaL ThErapEutic for Patients with Insomnia (SLEEP-I): A Protocol for a Pragmatic Randomized Controlled Trial. medRxiv. 2022. : | Other diagnosis (not depression/MDD) |  |
| 34283085 , Duan, Y. The Effectiveness of Sequentially Delivered Web-Based Interventions on Promoting Physical Activity and Fruit-Vegetable Consumption among Chinese College Students: Mixed Methods Study. Journal of Medical Internet Research. 2022. : | Other diagnosis (not depression/MDD) |  |
| 34283086 , E, W. B. Internet-Based Audiologist-Guided Cognitive Behavioral Therapy for Tinnitus: Randomized Controlled Trial. Journal of Medical Internet Research. 2022. : | Other diagnosis (not depression/MDD) |  |
| 34283087 , Enrique, A. Are Changes in Beliefs About Rumination and in Emotion Regulation Skills Mediators of the Effects of Internet-Delivered Cognitive-Behavioral Therapy for Depression and Anxiety? Results from a Randomized Controlled Trial. Cognitive Therapy and Research. 2021. : | Include this article |  |
|  |  | Inappropriate outcomes |
| 34283088 , Evans, K. Remotely Delivered Interventions to Support Women With Symptoms of Anxiety in Pregnancy: Mixed Methods Systematic Review and Meta-analysis. Journal of Medical Internet Research. 2022. : | Inappropriate study design |  |
| 34283089 , Fang, Y. Day-to-day variability in sleep parameters and depression risk: a prospective cohort study of training physicians. npj Digital Medicine. 2021. : | Other diagnosis (not depression/MDD) |  |
| 34283090 , Gardner, T. The Effect of Adjunct Telephone Support on Adherence and Outcomes of the Reboot Online Pain Management Program: Randomized Controlled Trial. Journal of Medical Internet Research. 2022. : | Other diagnosis (not depression/MDD) |  |
| 34283091 , Gawlytta, R. Internet-based cognitive-behavioural writing therapy for reducing post-traumatic stress after severe sepsis in patients and their spouses (REPAIR): Results of a randomised-controlled trial. BMJ Open. 2022. : | Other diagnosis (not depression/MDD) |  |
| 34283092 , Gladstone, T. R. G. PATH 2 Purpose: Design of a comparative effectiveness study of prevention programs for adolescents at-risk for depression in the primary care setting. Contemporary Clinical Trials. 2022:106763. : | Inappropriate study design |  |
| 34283093 , Grieve, P. The impact of internet-based cognitive behaviour therapy for perfectionism on different measures of perfectionism: a randomised controlled trial. Cognitive behaviour therapy. 2022. : | Other diagnosis (not depression/MDD) |  |
| 34283094 , Gulliver, A. Predictors of acceptability and engagement in a self-guided online program for depression and anxiety. Internet Interventions. 2021. : | Include this article |  |
|  |  | Inappropriate outcomes |
| 34283095 , Hagen, B. I. Goal Management Training and Computerized Cognitive Training in Depression-a 2-Year Follow-Up of a Randomized Controlled Trial. Frontiers in Psychiatry. 2021. : | Include this article |  |
|  |  | Inappropriate outcomes |
| 34283096 , Hale, L. Clinical and cost-effectiveness of an online-delivered group-based pain management programme in improving pain-related disability for people with persistent pain - Protocol for a non-inferiority randomised controlled trial (iSelf-help trial). BMJ Open. 2021. : | Other diagnosis (not depression/MDD) |  |
| 34283097 , Han, A. Effects of a coach-guided videoconferencing acceptance and commitment therapy intervention combined with psychoeducation on distressed individuals living with spinal cord injury: a preliminary mixed-methods study. Disability and rehabilitation. 2022:1-11. : | Other diagnosis (not depression/MDD) |  |
| 34283098 , Hanano, M. Measuring Adherence within a Self-Guided Online Intervention for Depression and Anxiety: Secondary Analyses of a Randomized Controlled Trial. JMIR Mental Health. 2022. : | Include this article |  |
|  |  | Other diagnosis (not depression/MDD) |
| 34283099 , Hentati, A. The effect of user interface on treatment engagement in a self-guided digital problem-solving intervention: A randomized controlled trial. Internet Interventions. 2021. : | Other diagnosis (not depression/MDD) |  |
| 34283100 , Hirten, R. P. Factors associated with longitudinal psychological and physiological stress in health care workers during the covid-19 pandemic: Observational study using apple watch data. Journal of Medical Internet Research. 2021. : | Other diagnosis (not depression/MDD) |  |
| 34283101 , Holtdirk, F. Results of the Optimune trial: A randomized controlled trial evaluating a novel Internet intervention for breast cancer survivors. PLoS ONE. 2021. : | Other diagnosis (not depression/MDD) |  |
| 34283102 , Huang, Q. The effect of online training-based continuous nursing care for rectal cancer-patients undergoing permanent colostomy. American Journal of Translational Research. 2021. : | Other diagnosis (not depression/MDD) |  |
| 34283103 , Humphries, S. M. Internet-based cognitive behavioral therapy for patients reporting symptoms of anxiety and depression after myocardial infarction: U-CARE heart randomized controlled trial twelve-month follow-up. Journal of Medical Internet Research. 2021. : | Other diagnosis (not depression/MDD) |  |
| 34283104 , Hunt, M. Efficacy of Zemedy, a Mobile Digital Therapeutic for the Self-management of Irritable Bowel Syndrome: crossover Randomized Controlled Trial. JMIR mHealth and uHealth. . : | Other diagnosis (not depression/MDD) |  |
| 34283105 , Hwang, H. The Efficacy of a Smartphone-Based App on Stress Reduction: Randomized Controlled Trial. Journal of Medical Internet Research. 2022. : | Other diagnosis (not depression/MDD) |  |
| 34283106 , Janela, D. Asynchronous and Tailored Digital Rehabilitation of Chronic Shoulder Pain: A Prospective Longitudinal Cohort Study. Journal of Pain Research. 2022. : | Other diagnosis (not depression/MDD) |  |
| 34283107 , Janjua, S. Digital interventions for the management of chronic obstructive pulmonary disease. Cochrane Database of Systematic Reviews. 2021. : | Other diagnosis (not depression/MDD) |  |
| 34283108 , Jensen, E. S. Effect of Sleep Disturbance Symptoms on Treatment Outcome in Blended Cognitive Behavioral Therapy for Depression (E-COMPARED Study): Secondary Analysis. Journal of Medical Internet Research. 2022. : | Other diagnosis (not depression/MDD) |  |
| 34283109 , Jonathan, G. K. A smartphone-based self-management intervention for bipolar disorder (livewell): User-centered development approach. JMIR Mental Health. 2021. : | Other diagnosis (not depression/MDD) |  |
| 34283110 , Joubert, A. E. Managing rumination and worry: A pilot study of an internet intervention targeting repetitive negative thinking in Australian adults. Journal of Affective Disorders. 2021. : | Other diagnosis (not depression/MDD) |  |
| 34283111 , Kang, S. H. Management of cardiovascular disease using an mHealth tool: a randomized clinical trial. npj Digital Medicine. 2021. : | Other diagnosis (not depression/MDD) |  |
| 34283113 , Keefe, R. S. E. Digital Intervention for Cognitive Deficits in Major Depression: A Randomized Controlled Trial to Assess Efficacy and Safety in Adults. The American journal of psychiatry. 2022:appiajp21020125. : | Other diagnosis (not depression/MDD) |  |
| 34283114 , Khan, K. Fidelity of delivery and contextual factors influencing children's level of engagement: Process evaluation of the online remote behavioral intervention for tics trial. Journal of Medical Internet Research. 2021. : | Other diagnosis (not depression/MDD) |  |
| 34283115 , Kim, M. Machine learning analysis to identify digital behavioral phenotypes for engagement and health outcome efficacy of an mHealth intervention for obesity: Randomized controlled trial. Journal of Medical Internet Research. 2021. : | Other diagnosis (not depression/MDD) |  |
| 34283116 , Koc, Z. The effectiveness of telehealth programs on the mental health of women with breast cancer: A systematic review. Journal of telemedicine and telecare. 2022:1357633X211069663. : | Other diagnosis (not depression/MDD) |  |
| 34283117 , Kohnen, M. Effectiveness and acceptance of technology-based psychological interventions for the acute treatment of unipolar depression: Systematic review and meta-analysis. Journal of Medical Internet Research. 2021. : | Inappropriate study design |  |
| 34283119 , Kramer, L. V. Effectiveness of a psychological online training to promote physical activity among students: protocol of a randomized-controlled trial. Trials. 2021. : | Other diagnosis (not depression/MDD) |  |
| 34283121 , Lakhtakia, T. Current directions in digital interventions for mood and anxiety disorders. Current Opinion in Psychiatry. 2022. : | Inappropriate study design |  |
| 34283122 , Laursen, S. L. Mobile diary app versus paper-based diary cards for patients with borderline personality disorder: Economic evaluation. Journal of Medical Internet Research. 2021. : | Other diagnosis (not depression/MDD) |  |
| 34283123 , Li, B. C. M. Utility of MyHEARTSMAP in Youth Presenting to the Emergency Department with Mental Health Concerns. Journal of Pediatrics. 2021. : | Other diagnosis (not depression/MDD) |  |
| 34283124 , Li, X. Development of a mobile application of internet-based support program on parenting outcomes for primiparous women. International Journal of Environmental Research and Public Health. 2021. : | Other diagnosis (not depression/MDD) |  |
| 34283125 , Li, Y. Mediating effects of stigma and depressive symptoms in a social media-based intervention to improve long-term quality of life among people living with hiv: Secondary analysis of a randomized controlled trial. Journal of Medical Internet Research. 2021. : | Other diagnosis (not depression/MDD) |  |
| 34283126 , Logie, C. H. Kukaa Salama (Staying Safe): Study protocol for a pre/post-trial of an interactive mHealth intervention for increasing COVID-19 prevention practices with urban refugee youth in Kampala, Uganda. BMJ Open. 2021. : | Other diagnosis (not depression/MDD) |  |
| 34283127 , Mahncke, H. W. A randomized clinical trial of plasticity-based cognitive training in mild traumatic brain injury. Brain. . : | Other diagnosis (not depression/MDD) |  |
| 34283128 , Marthick, M. Supportive care interventions for people with cancer assisted by digital technology: Systematic review. Journal of Medical Internet Research. 2021. : | Other diagnosis (not depression/MDD) |  |
| 34283129 , Michalski, D. The PostStroke-Manager - combining mobile, digital and sensor-based technology with personal assistance: protocol of the feasibility study. Neurological Research and Practice. 2021. : | Other diagnosis (not depression/MDD) |  |
| 34283130 , Milgrom, J. Internet and Face-to-face Cognitive Behavioral Therapy for Postnatal Depression Compared with Treatment as Usual: Randomized Controlled Trial of MumMoodBooster. Journal of Medical Internet Research. 2021. : | Other diagnosis (not depression/MDD) |  |
| 34283131 , Miller, H. N. The Nourish Protocol: A digital health randomized controlled trial to promote the DASH eating pattern among adults with hypertension. Contemporary Clinical Trials. 2021. : | Other diagnosis (not depression/MDD) |  |
| 34283132 , Moshe, I. Digital interventions for the treatment of depression: A meta-analytic review. Psychological bulletin. 2021. : | Inappropriate study design |  |
| 34283134 , Murray, E. Development, deployment and evaluation of digitally enabled, remote, supported rehabilitation for people with long COVID-19 (Living With COVID-19 Recovery): protocol for a mixed-methods study. BMJ Open. 2022. : | Other diagnosis (not depression/MDD) |  |
| 34283135 , Murray, G. Mindfulness-Based Online Intervention to Improve Quality of Life in Late-Stage Bipolar Disorder: A Randomized Clinical Trial. Journal of Consulting and Clinical Psychology. 2021. : | Other diagnosis (not depression/MDD) |  |
| 34283136 , Nadort, E. Internet-based treatment for depressive symptoms in hemodialysis patients: A cluster randomized controlled trial. General Hospital Psychiatry. 2022. : | Other diagnosis (not depression/MDD) |  |
| 34283137 , Navarra-Ventura, G. Virtual Reality-Based Early Neurocognitive Stimulation in Critically Ill Patients: A Pilot Randomized Clinical Trial. Journal of Personalized Medicine. 2021. : | Other diagnosis (not depression/MDD) |  |
| 34283138 , Navarra-Ventura, G. Virtual Reality-Based Early Neurocognitive Stimulation in Critically Ill Patients: A Pilot Randomized Clinical Trial. Journal of Personalized Medicine. 2021. : | Other diagnosis (not depression/MDD) |  |
| 34283139 , Nieto, I. 'Relearning how to think': A brief online intervention to modify biased interpretations in emotional disorders-study protocol for a randomised controlled trial. Trials. 2021. : | Other diagnosis (not depression/MDD) |  |
| 34283140 , Oehler, C. How are guide profession and routine care setting related to adherence and symptom change in iCBT for depression? - an explorative log-data analysis. Internet Interventions. 2021. : | Include this article |  |
|  |  | Inappropriate study design |
| 34283141 , ouml . Web-based self-help intervention for partners of cancer patients based on acceptance and commitment therapy and self-compassion training: a randomized controlled trial with automated versus personal feedback. Supportive Care in Cancer. . : | Other diagnosis (not depression/MDD) |  |
| 34283142 , Overas, C. K. Multimorbidity and co-occurring musculoskeletal pain do not modify the effect of the selfBACK app on low back pain-related disability. BMC Medicine. 2022. : | Other diagnosis (not depression/MDD) |  |
| 34283143 , Paalimaki-Paakki, K. Effectiveness of Digital Counseling Environments on Anxiety, Depression, and Adherence to Treatment Among Patients Who Are Chronically Ill: Systematic Review. Journal of Medical Internet Research. 2022. : | Other diagnosis (not depression/MDD) |  |
| 34283144 , Pan, C. 'Mindfulness Living with Insomnia': An mHealth intervention for individuals with insomnia in China: A study protocol of a randomised controlled trial. BMJ Open. 2022. : | Other diagnosis (not depression/MDD) |  |
| 34283145 , Pang, L. Role of Telemedicine in Inflammatory Bowel Disease: Systematic Review and Meta-analysis of Randomized Controlled Trials. Journal of Medical Internet Research. 2022. : | Inappropriate study design |  |
| 34283146 , Park, T. Digital Health Interventions by Clinical Pharmacists: A Systematic Review. International Journal of Environmental Research and Public Health. 2022. : | Inappropriate study design |  |
| 34283147 , Perkins, A. M. An enhanced psychological mindset intervention to promote adolescent wellbeing within educational settings: a feasibility randomized controlled trial. Journal of clinical psychology. . : | Other diagnosis (not depression/MDD) |  |
| 34283148 , Possemato, K. Web-Based Problem-solving Training With and Without Peer Support in Veterans With Unmet Mental Health Needs: Pilot Study of Feasibility, User Acceptability, and Participant Engagement. Journal of Medical Internet Research. 2022. : | Other diagnosis (not depression/MDD) |  |
| 34283149 , Pruessner, L. Integrating a web-based intervention into routine care of binge-eating disorder: Study protocol for a randomized controlled trial. Internet Interventions. 2022. : | Other diagnosis (not depression/MDD) |  |
| 34283150 , Ramallo-Farina, Y. Patient-reported outcome measures for knowledge transfer and behaviour modification interventions in type 2 diabetes - The INDICA study: A multiarm cluster randomised controlled trial. BMJ Open. 2021. : | Other diagnosis (not depression/MDD) |  |
| 34283151 , Ren, D. Internet-based interventions to promote help-seeking for mental health in LGBTQ+ young adults: Protocol for a randomized controlled trial. Internet Interventions. 2022. : | Other diagnosis (not depression/MDD) |  |
| 34283152 , Retzer, L. Anonymous online cognitive behavioral therapy for sleep disorders in shift workers-a study protocol for a randomized controlled trial. Trials. 2021. : | Other diagnosis (not depression/MDD) |  |
| 34283153 , Ruiz-Segovia, N. Healthy moms and babies preventive psychological intervention application: A study protocol. International Journal of Environmental Research and Public Health. 2021. : | Other diagnosis (not depression/MDD) |  |
| 34283154 , Sabri, B. Development, feasibility, acceptability and preliminary evaluation of the internet and mobile phone-based BSHAPE intervention for Immigrant survivors of cumulative trauma. Contemporary Clinical Trials. 2021. : | Other diagnosis (not depression/MDD) |  |
| 34283155 , Sadeghi, N. Innovating Care in Multiple Sclerosis: Feasibility of Synchronous Internet-Based Teleconsultation for Longitudinal Clinical Monitoring. Journal of Personalized Medicine. 2022. : | Other diagnosis (not depression/MDD) |  |
| 34283156 , Sato, D. Effectiveness of Unguided Internet-Based Cognitive Behavioral Therapy and the Three Good Things Exercise for Insomnia: 3-Arm Randomized Controlled Trial. Journal of Medical Internet Research. 2022. : | Other diagnosis (not depression/MDD) |  |
| 34283157 , Sauer, C. EHealth intervention to manage symptoms for patients with cancer on immunotherapy (SOFIA): A study protocol for a randomised controlled external pilot trial. BMJ Open. 2021. : | Other diagnosis (not depression/MDD) |  |
| 34283158 , Savard, J. Efficacy of a stepped care approach to deliver cognitive-behavioral therapy for insomnia in cancer patients: A noninferiority randomized controlled trial. Sleep. 2021. : | Other diagnosis (not depression/MDD) |  |
| 34283159 , Sawdon, O. L. Testing an early online intervention for the treatment of disturbed sleep during the COVID-19 pandemic in self-reported good and poor sleepers (Sleep COVID-19): study protocol for a randomised controlled trial. Trials. 2021. : | Other diagnosis (not depression/MDD) |  |
| 34283160 , Seth, D. Pediatric Inner-City Asthma. Immunology and Allergy Clinics of North America. 2021. : | Other diagnosis (not depression/MDD) |  |
| 34283161 , Shin, B. Effectiveness of self-guided virtual reality-based cognitive behavioral therapy for panic disorder: Randomized controlled trial. JMIR Mental Health. 2021. : | Other diagnosis (not depression/MDD) |  |
| 34283162 , Siebenhuner, A. R. Improvements in Health Might Contradict Adherence to Mobile Health Interventions: Findings from a Self-Care Cancer App Study. Journal of Alternative and Complementary Medicine. 2021. : | Other diagnosis (not depression/MDD) |  |
| 34283163 , Sikorski, F. The efficacy of automated feedback after internet-based depression screening: Study protocol of the German, three-armed, randomised controlled trial DISCOVER. Internet Interventions. 2021. : | Inappropriate intervention |  |
| 34283164 , Sim, W. H. The Role of Parent Engagement in a Web-Based Preventive Parenting Intervention for Child Mental Health in Predicting Parenting, Parent and Child Outcomes. International Journal of Environmental Research and Public Health. 2022. : | Other diagnosis (not depression/MDD) |  |
| 34283165 , Smith, S. K. A SMART approach to optimizing delivery of an mHealth intervention among cancer survivors with posttraumatic stress symptoms. Contemporary Clinical Trials. 2021. : | Other diagnosis (not depression/MDD) |  |
| 34283166 , Smoktunowicz, E. Resource-Based Internet Intervention (Med-Stress) to Improve Well-Being Among Medical Professionals: randomized Controlled Trial. Journal of Medical Internet Research. . : | Other diagnosis (not depression/MDD) |  |
| 34283167 , Spanhel, K. Effectiveness of an internet-based intervention to improve sleep difficulties in a culturally diverse sample of international students: A randomised controlled pilot study. Journal of Sleep Research. 2022. : | Other diagnosis (not depression/MDD) |  |
| 34283168 , Spanhel, K. Engaging Refugees With a Culturally Adapted Digital Intervention to Improve Sleep: A Randomized Controlled Pilot Trial. Frontiers in Psychiatry. 2022. : | Other diagnosis (not depression/MDD) |  |
| 34283169 , Sugunasingha, N. Evaluating an online self-help intervention for parents of children with food allergies. Pediatric Allergy and Immunology. 2022. : | Other diagnosis (not depression/MDD) |  |
| 34283170 , Syed Sheriff, R. J. A cultural experience to support mental health in people aged 16-24 during the COVID-19 pandemic compared to a typical museum website: study protocol of an online randomised controlled trial. Trials. 2021. : | Other diagnosis (not depression/MDD) |  |
| 34283171 , Sylvia, L. G. An online intervention for increasing physical activity in individuals with mood disorders at risk for cardiovascular disease: Design considerations. Journal of Affective Disorders. 2021. : | Other diagnosis (not depression/MDD) |  |
| 34283172 , Tamplin, J. ParkinSong Online: Protocol for a telehealth feasibility study of therapeutic group singing for people with Parkinson's disease. BMJ Open. 2021. : | Other diagnosis (not depression/MDD) |  |
| 34283173 , Thesen, T. Effectiveness of Internet-Based Cognitive Behavioral Therapy with Telephone Support for Noncardiac Chest Pain: Randomized Controlled Trial. Journal of Medical Internet Research. 2022. : | Other diagnosis (not depression/MDD) |  |
| 34283174 , Thorndike, F. Characteristics of respondents to a virtual trial of a digital behavioral treatment for insomnia during the COVID-19 pandemic. Sleep. . : | Other diagnosis (not depression/MDD) |  |
| 34283175 , Tort, S. In people with multiple sclerosis, what are the effects of memory rehabilitation? [Miscellaneous]. Cochrane Clinical Answers. 2022. : | Other diagnosis (not depression/MDD) |  |
| 34283176 , Tsiouris, A. An emotion-based online intervention for reducing anxiety and depression in cancer patients: Study protocol for a randomized controlled trial. Internet Interventions. 2021. : | Other diagnosis (not depression/MDD) |  |
| 34283177 , uuml . A randomized controlled trial on a self-guided Internet-based intervention for gambling problems. Scientific reports. . : | Other diagnosis (not depression/MDD) |  |
| 34283178 , van der Boom, B. Internet-delivered interventions for personality disorders - A scoping review. Internet Interventions. 2022. : | Other diagnosis (not depression/MDD) |  |
| 34283179 , Vance, D. E. A Randomized Clinical Trial on the Impact of Individually Targeted Computerized Cognitive Training on Quality of Life Indicators in Adults With HIV-Associated Neurocognitive Disorder in the Southeastern United States. Journal of the Association of Nurses in AIDS Care. 2022. : | Other diagnosis (not depression/MDD) |  |
| 34283180 , Verdam, M. G. E. Re-evaluating randomized clinical trials of psychological interventions: Impact of response shift on the interpretation of trial results. PLoS ONE. 2021. : | Inappropriate study design |  |
| 34283181 , Vermeir, J. F. Gamified Web-Delivered Attentional Bias Modification Training for Adults With Chronic Pain: Protocol for a Randomized, Double-blind, Placebo-Controlled Trial. JMIR Research Protocols. 2022. : | Other diagnosis (not depression/MDD) |  |
| 34283182 , Vigod, S. N. Mother Matters: Pilot randomized wait-list controlled trial of an online therapist-facilitated discussion board and support group for postpartum depression symptoms. Depression and Anxiety. 2021. : | Other diagnosis (not depression/MDD) |  |
| 34283183 , Vitger, T. Digital Shared Decision-Making Interventions in Mental Healthcare: A Systematic Review and Meta-Analysis. Frontiers in Psychiatry. 2021. : | Inappropriate study design |  |
| 34283184 , Welzel, F. D. Loss and bereavement in late life (60+): Study protocol for a randomized controlled trial regarding an internet-based self-help intervention. Internet Interventions. 2021. : | Other diagnosis (not depression/MDD) |  |
| 34283185 , Wen, S. Subtypes of smokers in a randomized controlled trial of a web-based smoking cessation program and their role in predicting intervention non-usage attrition: Implications for the development of tailored interventions. Internet Interventions. 2021. : | Other diagnosis (not depression/MDD) |  |
| 34283187 , Yang, M. Emphasizing mindfulness training in acceptance relieves anxiety and depression during pregnancy. Psychiatry Research. 2022. : | Other diagnosis (not depression/MDD) |  |
| 34283188 , Zheng, Q. Cost-effectiveness of Web-Based and Home-Based Postnatal Psychoeducational Interventions for First-time Mothers: Economic Evaluation Alongside Randomized Controlled Trial. Journal of Medical Internet Research. 2022. : | Other diagnosis (not depression/MDD) |  |

MDD, major depressive disorder.
