# Supplementary material for: Assessing Patient Adherence to and Engagement With Digital Interventions for Depression in Clinical Trials: Systematic Literature Review
Source: J Med Internet Res. 2023 Aug 11;25:e43727. doi: 10.2196/43727 (PMC10457707; doi:10.2196/43727)
Supplement: Multimedia Appendix 3 [file jmir_v25i1e43727_app3.docx]

**Forbes et al. 2023 Multimedia Appendix 3: Table S3**

Table S3. Sources and study types of each reference included in the SLR.

| **Articles Included in this Systematic Literature Review** | **Source** | **Study Type** |
| --- | --- | --- |
| [74], Heim E, Ramia JA, Hana RA, Burchert S, Carswell K, Cornelisz I, et al. Step-by-step: Feasibility randomised controlled trial of a mobile-based intervention for depression among populations affected by adversity in Lebanon. Internet Interv. 2021 Apr;24:100380. PMID: 33747798. doi: 10.1016/j.invent.2021.100380. | Original database search | Feasibility study |
| [108], Geraedts AS, Kleiboer AM, Wiezer NM, Cuijpers P, van Mechelen W, Anema JR. Feasibility of a worker-directed web-based intervention for employees with depressive symptoms. Internet Interventions. 2014;1(3):132-40. doi: [10.1016/j.invent.2014.07.001](https://doi.org/10.1016/j.invent.2014.07.001). | Original database search | Feasibility study |
| [119], Mohr DC, Duffecy J, Jin L, Ludman EJ, Lewis A, Begale M, et al. Multimodal e-mental health treatment for depression: A feasibility trial. J Med Internet Res. 2010 Dec 19;12(5):e48. PMID: 21169164. doi: 10.2196/jmir.1370. | Original database search | Feasibility study |
| [134], Addington EL, Cheung EO, Bassett SM, Kwok I, Schuette SA, Shiu E, et al. The MARIGOLD study: Feasibility and enhancement of an online intervention to improve emotion regulation in people with elevated depressive symptoms. J Affect Disord. 2019 Oct 1;257:352-64. PMID: [31302525](http://www.ncbi.nlm.nih.gov/pubmed/31302525). doi: [10.1016/j.jad.2019.07.049](https://doi.org/10.1016/j.jad.2019.07.049). | Original database search | Pilot study |
| [89], Gorges F, Oehler C, von Hirschhausen E, Hegerl U, Rummel-Kluge C. GET.HAPPY - Acceptance of an internet-based self-management positive psychology intervention for adult primary care patients with mild and moderate depression or dysthymia: A pilot study. Internet Interv. 2018 Jun;12:26-35. PMID: [30135766](http://www.ncbi.nlm.nih.gov/pubmed/30135766). doi: [10.1016/j.invent.2018.03.001](https://doi.org/10.1016/j.invent.2018.03.001). | Original database search | Pilot study |
| [94], Tomasino KN, Lattie EG, Ho J, Palac HL, Kaiser SM, Mohr DC. Harnessing peer support in an online intervention for older adults with depression. Am J Geriatr Psychiatry. 2017 Oct;25(10):1109-19. PMID: [28571785](http://www.ncbi.nlm.nih.gov/pubmed/28571785). doi: [10.1016/j.jagp.2017.04.015](https://doi.org/10.1016/j.jagp.2017.04.015). | Original database search | Pilot study |
| [127], Wilkinson ST, Ostroff RB, Sanacora G. Computer-assisted cognitive behavior therapy to prevent relapse following electroconvulsive therapy. J ECT. 2017 Mar;33(1):52-7. PMID: 27564424. doi: 10.1097/YCT.0000000000000348. | Original database search | Pilot study |
| [147], Williams C, McClay CA, Martinez R, Morrison J, Haig C, Jones R, et al. Online CBT life skills programme for low mood and anxiety: Study protocol for a pilot randomized controlled trial. Trials. 2016 Apr 27;17(1):220. PMID: 27121090. doi: 10.1186/s13063-016-1336-y. | Original database search | Pilot study |
| [150], Rickhi B, Kania-Richmond A, Moritz S, Cohen J, Paccagnan P, Dennis C, et al. Evaluation of a spirituality informed e-mental health tool as an intervention for major depressive disorder in adolescents and young adults - A randomized controlled pilot trial. BMC Complement Altern Med. 2015 Dec 24;15:450. PMID: [26702639](http://www.ncbi.nlm.nih.gov/pubmed/26702639). doi: [10.1186/s12906-015-0968-x](https://doi.org/10.1186/s12906-015-0968-x). | Original database search | Pilot study |
| [153], Santucci LC, McHugh RK, Elkins RM, Schechter B, Ross MS, Landa CE, et al. Pilot implementation of computerized cognitive behavioral therapy in a university health setting. Adm Policy Ment Health. 2014 Jul;41(4):514-21. PMID: [23592231](http://www.ncbi.nlm.nih.gov/pubmed/23592231). doi: [10.1007/s10488-013-0488-2](https://doi.org/10.1007/s10488-013-0488-2). | Original database search | Pilot study |
| [116], Burns MN, Begale M, Duffecy J, Gergle D, Karr CJ, Giangrande E, et al. Harnessing context sensing to develop a mobile intervention for depression. J Med Internet Res. 2011 Aug 12;13(3):e55. PMID: 21840837. doi: 10.2196/jmir.1838. | Original database search | Pilot study |
| [75], Rahmadiana M, Karyotaki E, Schulte M, Ebert DD, Passchier J, Cuijpers P, et al. Transdiagnostic internet intervention for Indonesian university students with depression and anxiety: Evaluation of feasibility and acceptability. JMIR Ment Health. 2021 Mar 5;8(3):e20036. PMID: 33666553. doi: 10.2196/20036. | Original database search | Pilot study |
| [125], Lukas CA, Berking M. Blending group-based psychoeducation with a smartphone intervention for the reduction of depressive symptoms: Results of a randomized controlled pilot study. Pilot Feasibility Stud. 2021 Feb 24;7(1):57. PMID: 33627198. doi: 10.1186/s40814-021-00799-y. | Original database search | Pilot study |
| [76], Grinberg A. Mobile cognitive training for the cognitive symptoms of depression in young adults: A double-blind, randomized pilot study with active control [dissertation]. The City University of New York: Degree of Doctor of Philosophy; 2020. 2020. | Original database search | Pilot study |
| [62], Mohr DC, Duffecy J, Ho J, Kwasny M, Cai X, Burns MN, et al. A randomized controlled trial evaluating a manualized TeleCoaching protocol for improving adherence to a web-based intervention for the treatment of depression. PLoS One. 2013;8(8):e70086. PMID: [23990896](http://www.ncbi.nlm.nih.gov/pubmed/23990896). doi: [10.1371/journal.pone.0070086](https://doi.org/10.1371/journal.pone.0070086). | Original database search | Pilot study |
| [90], Lambert JD, Greaves CJ, Farrand P, Price L, Haase AM, Taylor AH. Web-based intervention using behavioral activation and physical activity for adults with depression (the eMotion study): Pilot randomized controlled trial. J Med Internet Res. 2018 Jul 16;20(7):e10112. PMID: [30012547](http://www.ncbi.nlm.nih.gov/pubmed/30012547). doi: [10.2196/10112](https://doi.org/10.2196/10112). | Original database search | Pilot study |
| [135], Karyotaki E, Klein AM, Riper H, Wit L, Krijnen L, Bol E, et al. Examining the effectiveness of a web-based intervention for symptoms of depression and anxiety in college students: Study protocol of a randomised controlled trial. BMJ Open. 2019 May 14;9(5):e028739. PMID: [31092668](http://www.ncbi.nlm.nih.gov/pubmed/31092668). doi: [10.1136/bmjopen-2018-028739](https://doi.org/10.1136/bmjopen-2018-028739). | Original database search | RCT |
| [151], Littlewood E, Duarte A, Hewitt C, Knowles S, Palmer S, Walker S, et al. A randomised controlled trial of computerised cognitive behaviour therapy for the treatment of depression in primary care: The Randomised Evaluation of the Effectiveness and Acceptability of Computerised Therapy (REEACT) trial. Health Technol Assess. 2015 Dec;19(101):viii, xxi-171. PMID: [26685904](http://www.ncbi.nlm.nih.gov/pubmed/26685904). doi: [10.3310/hta191010](https://doi.org/10.3310/hta191010). | Original database search | RCT |
| [136], Mira A, Soler C, Alda M, Banos R, Castilla D, Castro A, et al. Exploring the relationship between the acceptability of an internet-based intervention for depression in primary care and clinical outcomes: Secondary analysis of a randomized controlled trial. Front Psychiatry. 2019;10:325. PMID: [31133899](http://www.ncbi.nlm.nih.gov/pubmed/31133899). doi: [10.3389/fpsyt.2019.00325](https://doi.org/10.3389/fpsyt.2019.00325). | Original database search | RCT |
| [77], Kramer LV, Grunzig SD, Baumeister H, Ebert DD, Bengel J. Effectiveness of a guided web-based intervention to reduce depressive symptoms before outpatient psychotherapy: a pragmatic randomized controlled trial. Psychother Psychosom. 2021;90(4):233-42. PMID: [33946072](http://www.ncbi.nlm.nih.gov/pubmed/33946072). doi: [10.1159/000515625](https://doi.org/10.1159/000515625). | Original database search | RCT |
| [126], Grafe V, Berger T, Hautzinger M, Hohagen F, Lutz W, Meyer B, et al. Health economic evaluation of a web-based intervention for depression: The EVIDENT-trial, a randomized controlled study. Health Econ Rev. 2019 Jun 7;9(1):16. PMID: [31175475](http://www.ncbi.nlm.nih.gov/pubmed/31175475). doi: [10.1186/s13561-019-0233-y](https://doi.org/10.1186/s13561-019-0233-y). | Original database search | RCT |
| [137], Kraepelien M, Blom K, Lindefors N, Johansson R, Kaldo V. The effects of component-specific treatment compliance in individually tailored internet-based treatment. Clin Psychol Psychother. 2019 May;26(3):298-308. PMID: [30650232](http://www.ncbi.nlm.nih.gov/pubmed/30650232). doi: [10.1002/cpp.2351](https://doi.org/10.1002/cpp.2351). | Original database search | RCT |
| [91], Thase ME, Wright JH, Eells TD, Barrett MS, Wisniewski SR, Balasubramani GK, et al. Improving the efficiency of psychotherapy for depression: Computer-assisted versus standard CBT. Am J Psychiatry. 2018 Mar 1;175(3):242-50. PMID: [28969439](http://www.ncbi.nlm.nih.gov/pubmed/28969439). doi: [10.1176/appi.ajp.2017.17010089](https://doi.org/10.1176/appi.ajp.2017.17010089). | Original database search | RCT |
| [141], Grunzig SD, Baumeister H, Bengel J, Ebert D, Kramer L. Effectiveness and acceptance of a web-based depression intervention during waiting time for outpatient psychotherapy: Study protocol for a randomized controlled trial. Trials. 2018 May 22;19(1):285. PMID: [29788996](http://www.ncbi.nlm.nih.gov/pubmed/29788996). doi: [10.1186/s13063-018-2657-9](https://doi.org/10.1186/s13063-018-2657-9). | Original database search | RCT |
| [142], Lobner M, Pabst A, Stein J, Dorow M, Matschinger H, Luppa M, et al. Computerized cognitive behavior therapy for patients with mild to moderately severe depression in primary care: A pragmatic cluster randomized controlled trial (@ktiv). J Affect Disord. 2018 Oct 1;238:317-26. PMID: 29902736. doi: 10.1016/j.jad.2018.06.008. | Original database search | RCT |
| [143], Fuhr K, Schroder J, Berger T, Moritz S, Meyer B, Lutz W, et al. The association between adherence and outcome in an internet intervention for depression. J Affect Disord. 2018 Mar 15;229:443-9. PMID: [29331706](http://www.ncbi.nlm.nih.gov/pubmed/29331706). doi: [10.1016/j.jad.2017.12.028](https://doi.org/10.1016/j.jad.2017.12.028). | Original database search | RCT |
| [144], Weisel KK, Zarski AC, Berger T, Schaub MP, Krieger T, Moser CT, et al. Transdiagnostic tailored internet- and mobile-based guided treatment for major depressive disorder and comorbid anxiety: Study protocol of a randomized controlled trial. Front Psychiatry. 2018;9:274. PMID: [30022954](http://www.ncbi.nlm.nih.gov/pubmed/30022954). doi: [10.3389/fpsyt.2018.00274](https://doi.org/10.3389/fpsyt.2018.00274). | Original database search | RCT |
| [95], Beevers CG, Pearson R, Hoffman JS, Foulser AA, Shumake J, Meyer B. Effectiveness of an internet intervention (Deprexis) for depression in a united states adult sample: A parallel-group pragmatic randomized controlled trial. J Consult Clin Psychol. 2017 Apr;85(4):367-80. PMID: [28230390](http://www.ncbi.nlm.nih.gov/pubmed/28230390). doi: [10.1037/ccp0000171](https://doi.org/10.1037/ccp0000171). | Original database search | RCT |
| [96], Klein JP, Spath C, Schroder J, Meyer B, Greiner W, Hautzinger M, et al. Time to remission from mild to moderate depressive symptoms: One year results from the EVIDENT-study, an RCT of an internet intervention for depression. Behav Res Ther. 2017 Oct;97:154-62. PMID: 28797829. doi: 10.1016/j.brat.2017.07.013. | Original database search | RCT |
| [148], Brabyn S, Araya R, Barkham M, Bower P, Cooper C, Duarte A, et al. The second randomised evaluation of the effectiveness, cost-effectiveness and acceptability of computerised therapy (REEACT-2) trial: Does the provision of telephone support enhance the effectiveness of computer-delivered cognitive behaviour therapy? A randomised controlled trial. Health Technol Assess. 2016 Nov;20(89):1-64. PMID: [27922448](http://www.ncbi.nlm.nih.gov/pubmed/27922448). doi: [10.3310/hta20890](https://doi.org/10.3310/hta20890). | Original database search | RCT |
| [102], Meyer B, Bierbrodt J, Schröder J, Berger T, Beevers CG, Weiss M, et al. Effects of an Internet intervention (Deprexis) on severe depression symptoms: Randomized controlled trial. Internet Interventions. 2015;2(1):48-59. doi: 10.1016/j.invent.2014.12.003. | Original database search | RCT |
| [103], Gilbody S, Littlewood E, Hewitt C, Brierley G, Tharmanathan P, Araya R, et al. Computerised cognitive behaviour therapy (cCBT) as treatment for depression in primary care (REEACT trial): large scale pragmatic randomised controlled trial. BMJ. 2015 Nov 11;351:h5627. PMID: 26559241. doi: 10.1136/bmj.h5627. | Original database search | RCT |
| [104], Hoifodt RS, Mittner M, Lillevoll K, Katla SK, Kolstrup N, Eisemann M, et al. Predictors of response to web-based cognitive behavioral therapy with high-intensity face-to-face therapist guidance for depression: A bayesian analysis. J Med Internet Res. 2015 Sep 2;17(9):e197. PMID: 26333818. doi: 10.2196/jmir.4351. | Original database search | RCT |
| [83], Oehler C, Gorges F, Rogalla M, Rummel-Kluge C, Hegerl U. Efficacy of a guided web-based self-management intervention for depression or dysthymia: Randomized controlled trial with a 12-month follow-up using an active control condition. J Med Internet Res. 2020 Jul 14;22(7):e15361. PMID: 32673233. doi: 10.2196/15361. | Original database search | RCT |
| [109], Schneider J, Sarrami Foroushani P, Grime P, Thornicroft G. Acceptability of online self-help to people with depression: Users' views of MoodGYM versus informational websites. J Med Internet Res. 2014 Mar 28;16(3):e90. PMID: 24681717. doi: 10.2196/jmir.2871. | Original database search | RCT |
| [110], Kivi M, Eriksson MC, Hange D, Petersson EL, Vernmark K, Johansson B, et al. Internet-based therapy for mild to moderate depression in Swedish primary care: Short term results from the PRIM-NET randomized controlled trial. Cogn Behav Ther. 2014;43(4):289-98. PMID: 24911260. doi: 10.1080/16506073.2014.921834. | Original database search | RCT |
| [78], Batterham PJ, Calear AL, Sunderland M, Kay-Lambkin F, Farrer LM, Christensen H, et al. A brief intervention to increase uptake and adherence of an internet-based program for depression and anxiety (enhancing engagement with psychosocial interventions): Randomized controlled trial. J Med Internet Res. 2021 Jul 27;23(7):e23029. PMID: 34313595. doi: 10.2196/23029 | Original database search | RCT |
| [71], Bur OT, Krieger T, Moritz S, Klein JP, Berger T. Optimizing the context of support of web-based self-help in individuals with mild to moderate depressive symptoms: A randomized full factorial trial. Behav Res Ther. 2022 May;152:104070. PMID: 35306266. doi: 10.1016/j.brat.2022.104070. | Original database search | RCT |
| [79], Perez JC, Fernandez O, Caceres C, Carrasco AE, Moessner M, Bauer S, et al. An adjunctive internet-based intervention to enhance treatment for depression in adults: Randomized controlled trial. JMIR Ment Health. 2021 Dec 16;8(12):e26814. PMID: [34927594](http://www.ncbi.nlm.nih.gov/pubmed/34927594). doi: [10.2196/26814](https://doi.org/10.2196/26814). | Original database search | RCT |
| [72], Karyotaki E, Klein AM, Ciharova M, Bolinski F, Krijnen L, de Koning L, et al. Guided internet-based transdiagnostic individually tailored cognitive behavioral therapy for symptoms of depression and/or anxiety in college students: A randomized controlled trial. Behav Res Ther. 2022 Mar;150:104028. PMID: [35066365](http://www.ncbi.nlm.nih.gov/pubmed/35066365). doi: [10.1016/j.brat.2021.104028](https://doi.org/10.1016/j.brat.2021.104028). | Original database search | RCT |
| [81], Kramer LV, Grunzig SD, Baumeister H, Ebert DD, Bengel J. Effectiveness of a guided web-based intervention to reduce depressive symptoms before outpatient psychotherapy: a pragmatic randomized controlled trial. Psychother Psychosom. 2021;90(4):233-42. PMID: 33946072. doi: 10.1159/000515625. | Original database search | RCT |
| [130], Kramer LV, Mueller-Weinitschke C, Zeiss T, Baumeister H, Ebert DD, Bengel J. Effectiveness of a web-based behavioural activation intervention for individuals with depression based on the Health Action Process Approach: Protocol for a randomised controlled trial with a 6-month follow-up. BMJ Open. 2022 Jan 24;12(1):e054775. PMID: 35074820. doi: 10.1136/bmjopen-2021-054775. | Original database search | RCT |
| [80], Moskowitz JT, Addington EL, Shiu E, Bassett SM, Schuette S, Kwok I, et al. Facilitator contact, discussion boards, and virtual badges as adherence enhancements to a web-based, self-guided, positive psychological intervention for depression: Randomized controlled trial. J Med Internet Res. 2021 Sep 22;23(9):e25922. PMID: 34550076. doi: 10.2196/25922. | Original database search | RCT |
| [73], Wright JH, Owen J, Eells TD, Antle B, Bishop LB, Girdler R, et al. Effect of computer-assisted cognitive behavior therapy vs usual care on depression among adults in primary care: A randomized clinical trial. JAMA Netw Open. 2022 Feb 1;5(2):e2146716. PMID: 35142833. doi: 10.1001/jamanetworkopen.2021.46716. | Original database search | RCT |
| [61], Donkin L, Hickie IB, Christensen H, Naismith SL, Neal B, Cockayne NL, et al. Rethinking the dose-response relationship between usage and outcome in an online intervention for depression: Randomized controlled trial. J Med Internet Res. 2013 Oct 17;15(10):e231. PMID: 24135213. doi: 10.2196/jmir.2771. | Original database search | RCT |
| [112], Hoifodt RS, Lillevoll KR, Griffiths KM, Wilsgaard T, Eisemann M, Waterloo K, et al. The clinical effectiveness of web-based cognitive behavioral therapy with face-to-face therapist support for depressed primary care patients: Randomized controlled trial. J Med Internet Res. 2013 Aug 5;15(8):e153. PMID: 23916965. doi: 10.2196/jmir.2714. | Original database search | RCT |
| [114], Bolier L, Haverman M, Kramer J, Westerhof GJ, Riper H, Walburg JA, et al. An Internet-based intervention to promote mental fitness for mildly depressed adults: Randomized controlled trial. J Med Internet Res. 2013 Sep 16;15(9):e200. PMID: [24041479](http://www.ncbi.nlm.nih.gov/pubmed/24041479). doi: [10.2196/jmir.2603](https://doi.org/10.2196/jmir.2603). | Original database search | RCT |
| [63], Moritz S, Schilling L, Hauschildt M, Schroder J, Treszl A. A randomized controlled trial of internet-based therapy in depression. Behav Res Ther. 2012 Aug;50(7-8):513-21. PMID: 22677231. doi: 10.1016/j.brat.2012.04.006. | Original database search | RCT |
| [117], Levin W, Campbell DR, McGovern KB, Gau JM, Kosty DB, Seeley JR, et al. A computer-assisted depression intervention in primary care. Psychol Med. 2011 Jul;41(7):1373-83. PMID: 20961474. doi: 10.1017/S0033291710001935. | Original database search | RCT |
| [118], Berger T, Hammerli K, Gubser N, Andersson G, Caspar F. Internet-based treatment of depression: A randomized controlled trial comparing guided with unguided self-help. Cogn Behav Ther. 2011;40(4):251-66. PMID: 22060248. doi: 10.1080/16506073.2011.616531. | Original database search | RCT |
| [160], Titov N, Andrews G, Davies M, McIntyre K, Robinson E, Solley K. Internet treatment for depression: A randomized controlled trial comparing clinician vs. technician assistance. PLoS One. 2010 Jun 8;5(6):e10939. PMID: 20544030. doi: 10.1371/journal.pone.0010939. | Original database search | RCT |
| [161], Hatcher S, Whittaker R, Patton M, Miles WS, Ralph N, Kercher K, et al. Web-based therapy plus support by a coach in depressed patients referred to secondary mental health care: Randomized controlled trial. JMIR Ment Health. 2018 Jan 23;5(1):e5. PMID: [29362207](http://www.ncbi.nlm.nih.gov/pubmed/29362207). doi: [10.2196/mental.8510](https://doi.org/10.2196/mental.8510). | Original database search | RCT |
| [84], Salamanca-Sanabria A, Richards D, Timulak L, Connell S, Mojica Perilla M, Parra-Villa Y, et al. A culturally adapted cognitive behavioral internet-delivered intervention for depressive symptoms: Randomized controlled trial. JMIR Ment Health. 2020 Jan 31;7(1):e13392. PMID: 32003749. doi: 10.2196/13392. | Original database search | RCT |
| [131], Fitzsimmons-Craft EE, Taylor CB, Newman MG, Zainal NH, Rojas-Ashe EE, Lipson SK, et al. Harnessing mobile technology to reduce mental health disorders in college populations: A randomized controlled trial study protocol. Contemp Clin Trials. 2021 Apr;103:106320. PMID: 33582295. doi: 10.1016/j.cct.2021.106320. | Original database search | RCT |
| [132], Kramer R, Kohler S. Evaluation of the online-based self-help programme "Selfapy" in patients with unipolar depression: Study protocol for a randomized, blinded parallel group dismantling study. Trials. 2021 Apr 9;22(1):264. PMID: 33836810. doi: 10.1186/s13063-021-05218-4. | Original database search | RCT |
| [133], Lopes RT, Meyer B, Berger T, Svacina MA. Effectiveness of an internet-based self-guided program to treat depression in a sample of Brazilian users: A study protocol. Braz J Psychiatry. 2020;42(3):322-8. PMID: 32159711. doi: 10.1590/1516-4446-2019-0582. | Original database search | RCT |
| [99], Kenter RM, Cuijpers P, Beekman A, van Straten A. Effectiveness of a web-based guided self-help intervention for outpatients with a depressive disorder: Short-term Results from a randomized controlled trial. J Med Internet Res. 2016 Mar 31;18(3):e80. PMID: [27032449](http://www.ncbi.nlm.nih.gov/pubmed/27032449). doi: [10.2196/jmir.4861](https://doi.org/10.2196/jmir.4861). | Original database search | RCT |
| [114], Kelders SM, Bohlmeijer ET, Van Gemert-Pijnen JE. Participants, usage, and use patterns of a web-based intervention for the prevention of depression within a randomized controlled trial. J Med Internet Res. 2013 Aug 20;15(8):e172. PMID: 23963284. doi: 10.2196/jmir.2258. | Original database search | RCT |
| [105], Kelders SM, Bohlmeijer ET, Pots WT, van Gemert-Pijnen JE. Comparing human and automated support for depression: Fractional factorial randomized controlled trial. Behav Res Ther. 2015 Sep;72:72-80. PMID: [26196078](http://www.ncbi.nlm.nih.gov/pubmed/26196078). doi: [10.1016/j.brat.2015.06.014](https://doi.org/10.1016/j.brat.2015.06.014). | Original database search | RCT |
| [154], Richards D, Timulak L, Doherty G, Sharry J, Colla A, Joyce C, et al. Internet-delivered treatment: Its potential as a low-intensity community intervention for adults with symptoms of depression: protocol for a randomized controlled trial. BMC Psychiatry. 2014 May 21;14:147. PMID: [24886179](http://www.ncbi.nlm.nih.gov/pubmed/24886179). doi: [10.1186/1471-244x-14-147](https://doi.org/10.1186/1471-244X-14-147). | Original database search | RCT |
| [100], Kordy H, Wolf M, Aulich K, Burgy M, Hegerl U, Husing J, et al. Internet-delivered disease management for recurrent depression: A multicenter randomized controlled trial. Psychother Psychosom. 2016;85(2):91-8. PMID: 26808817. doi: 10.1159/000441951. | Original database search | RCT |
| [145], Richards D, Duffy D, Blackburn B, Earley C, Enrique A, Palacios J, et al. Digital IAPT: The effectiveness & cost-effectiveness of internet-delivered interventions for depression and anxiety disorders in the Improving Access to Psychological Therapies programme: study protocol for a randomised control trial. BMC Psychiatry. 2018 Mar 2;18(1):59. PMID: [29499675](http://www.ncbi.nlm.nih.gov/pubmed/29499675). doi: [10.1186/s12888-018-1639-5](https://doi.org/10.1186/s12888-018-1639-5). | Original database search | RCT |
| [138], Motter JN, Grinberg A, Lieberman DH, Iqnaibi WB, Sneed JR. Computerized cognitive training in young adults with depressive symptoms: Effects on mood, cognition, and everyday functioning. J Affect Disord. 2019 Feb 15;245:28-37. PMID: 30366235. doi: 10.1016/j.jad.2018.10.109. | Original database search | RCT |
| [139], Antle BF, Owen JJ, Eells TD, Wells MJ, Harris LM, Cappiccie A, et al. Dissemination of computer-assisted cognitive-behavior therapy for depression in primary care. Contemp Clin Trials. 2019 Mar;78:46-52. PMID: [30572162](http://www.ncbi.nlm.nih.gov/pubmed/30572162). doi: [10.1016/j.cct.2018.11.001](https://doi.org/10.1016/j.cct.2018.11.001). | Original database search | RCT |
| [81], Quinonez-Freire C, Vara MD, Herrero R, Mira A, Garcia-Palacios A, Botella C, et al. Cultural adaptation of the Smiling is Fun program for the treatment of depression in the Ecuadorian public health care system: A study protocol for a randomized controlled trial. Internet Interv. 2021 Mar;23:100352. PMID: [33335847](http://www.ncbi.nlm.nih.gov/pubmed/33335847). doi: [10.1016/j.invent.2020.100352](https://doi.org/10.1016/j.invent.2020.100352). | Original database search | RCT |
| [85], Gili M, Castro A, Garcia-Palacios A, Garcia-Campayo J, Mayoral-Cleries F, Botella C, et al. Efficacy of three low-intensity, internet-based psychological interventions for the treatment of depression in primary care: Randomized controlled trial. J Med Internet Res. 2020 Jun 5;22(6):e15845. PMID: 32501276. doi: 10.2196/15845. | Original database search | RCT |
| [90], MacLean S, Corsi DJ, Litchfield S, Kucharski J, Genise K, Selaman Z, et al. Coach-facilitated web-based therapy compared with information about web-based resources in patients referred to secondary mental health care for depression: Randomized controlled trial. J Med Internet Res. 2020 Jun 9;22(6):e15001. PMID: 32515740. doi: 10.2196/15001. | Original database search | RCT |
| [129], Robertson L, Smith M, Castle D, Tannenbaum D. Using the Internet to enhance the treatment of depression. Australasian Psychiatry. 2006;14(4):413-7. doi: 10.1111/j.1440-1665.2006.02315.x. | Relevant review article | Feasibility study |
| [128], Anguera JA, Jordan JT, Castaneda D, Gazzaley A, Arean PA. Conducting a fully mobile and randomised clinical trial for depression: Access, engagement and expense. BMJ Innov. 2016 Jan;2(1):14-21. PMID: 27019745. doi: 10.1136/bmjinnov-2015-000098. | Relevant review article | Feasibility study |
| [64], Stiles-Shields C, Montague E, Kwasny MJ, Mohr DC. Behavioral and cognitive intervention strategies delivered via coached apps for depression: Pilot trial. Psychol Serv. 2019 May;16(2):233-8. PMID: [30407055](http://www.ncbi.nlm.nih.gov/pubmed/30407055). doi: [10.1037/ser0000261](https://doi.org/10.1037/ser0000261). | Relevant review article | Feasibility study |
| [92], Caplan S, Sosa Lovera A, Reyna Liberato P. A feasibility study of a mental health mobile app in the Dominican Republic: The untold story. International Journal of Mental Health. 2019;47(4):311-45. doi: 10.1080/00207411.2018.1553486. | Relevant review article | Feasibility study |
| [156], Watts S, Mackenzie A, Thomas C, Griskaitis A, Mewton L, Williams A, et al. CBT for depression: A pilot RCT comparing mobile phone vs. computer. BMC Psychiatry. 2013 Feb 7;13:49. PMID: 23391304. doi: 10.1186/1471-244x-13-49. | Relevant review article | Pilot study |
| [97], Mohr DC, Tomasino KN, Lattie EG, Palac HL, Kwasny MJ, Weingardt K, et al. IntelliCare: an eclectic, skills-based app suite for the treatment of depression and anxiety. J Med Internet Res. 2017 Jan 5;19(1):e10. PMID: 28057609. doi: 10.2196/jmir.6645. | Relevant review article | Pilot study |
| [146], Mehrotra S, Sudhir P, Rao G, Thirthalli J, Srikanth TK. Development and pilot testing of an internet-based self-help intervention for depression for Indian users. Behav Sci (Basel). 2018 Mar 22;8(4). PMID: 29565278. doi: 10.3390/bs8040036. | Relevant review article | Pilot study |
| [140], J Dahne, A Collado, CW Lejuez, CM Risco, VA Diaz, L Coles, J Kustanowitz, MJ Zvolensky, MJ Carpenter. Pilot randomized controlled trial of a Spanish-language Behavioral Activation mobile app (¡Aptívate!) for the treatment of depressive symptoms among United States Latinx adults with limited English proficiency. Journal of affective disorders. 2019. 250: 10.1016/j.jad.2019.03.009, PMID = 30870770 | Relevant review article | Pilot study |
| [82], Lukas CA, Eskofier B, Berking M. A gamified smartphone-based intervention for depression: Randomized controlled pilot trial. JMIR Ment Health. 2021 Jul 20;8(7):e16643. PMID: 34283037. doi: 10.2196/16643. | Relevant review article | Pilot study |
| [124], Warmerdam L, van Straten A, Twisk J, Riper H, Cuijpers P. Internet-based treatment for adults with depressive symptoms: Randomized controlled trial. J Med Internet Res. 2008 Nov 20;10(4):e44. PMID: [19033149](http://www.ncbi.nlm.nih.gov/pubmed/19033149). doi: [10.2196/jmir.1094](https://doi.org/10.2196/jmir.1094). | Relevant review article | RCT |
| [121], Ruwaard J, Schrieken B, Schrijver M, Broeksteeg J, Dekker J, Vermeulen H, et al. Standardized web-based cognitive behavioural therapy of mild to moderate depression: A randomized controlled trial with a long-term follow-up. Cogn Behav Ther. 2009;38(4):206-21. PMID: 19221919. doi: 10.1080/16506070802408086. | Relevant review article | RCT |
| [122], Perini S, Titov N, Andrews G. Clinician-assisted Internet-based treatment is effective for depression: Randomized controlled trial. Aust N Z J Psychiatry. 2009 Jun;43(6):571-8. PMID: 19440890. doi: 10.1080/00048670902873722. | Relevant review article | RCT |
| [123], de Graaf LE, Gerhards SA, Arntz A, Riper H, Metsemakers JF, Evers SM, et al. Clinical effectiveness of online computerised cognitive-behavioural therapy without support for depression in primary care: Randomised trial. Br J Psychiatry. 2009 Jul;195(1):73-80. PMID: 19567900. doi: 10.1192/bjp.bp.108.054429. | Relevant review article | RCT |
| [120], Vernmark K, Lenndin J, Bjarehed J, Carlsson M, Karlsson J, Oberg J, et al. Internet administered guided self-help versus individualized e-mail therapy: A randomized trial of two versions of CBT for major depression. Behav Res Ther. 2010 May;48(5):368-76. PMID: 20152960. doi: 10.1016/j.brat.2010.01.005. | Relevant review article | RCT |
| [158], Johansson R, Sjoberg E, Sjogren M, Johnsson E, Carlbring P, Andersson T, et al. Tailored vs. standardized internet-based cognitive behavior therapy for depression and comorbid symptoms: A randomized controlled trial. PLoS One. 2012;7(5):e36905. PMID: 22615841. doi: 10.1371/journal.pone.0036905. | Relevant review article | RCT |
| [115], Williams AD, Blackwell SE, Mackenzie A, Holmes EA, Andrews G. Combining imagination and reason in the treatment of depression: A randomized controlled trial of internet-based cognitive-bias modification and internet-CBT for depression. J Consult Clin Psychol. 2013 Oct;81(5):793-9. PMID: 23750459. doi: 10.1037/a0033247. | Relevant review article | RCT |
| [155], Wagner B, Horn AB, Maercker A. Internet-based versus face-to-face cognitive-behavioral intervention for depression: A randomized controlled non-inferiority trial. J Affect Disord. 2014 Jan;152-154:113-21. PMID: [23886401](http://www.ncbi.nlm.nih.gov/pubmed/23886401). doi: [10.1016/j.jad.2013.06.032](https://doi.org/10.1016/j.jad.2013.06.032). | Relevant review article | RCT |
| [152], Roepke AM, Jaffee SR, Riffle OM, McGonigal J, Broome R, Maxwell B. Randomized controlled trial of SuperBetter, a smartphone-based/internet-based self-help tool to reduce depressive symptoms. Games Health J. 2015 Jun;4(3):235-46. PMID: 26182069. doi: 10.1089/g4h.2014.0046. | Relevant review article | RCT |
| [106], Richards D, Timulak L, O'Brien E, Hayes C, Vigano N, Sharry J, et al. A randomized controlled trial of an internet-delivered treatment: Its potential as a low-intensity community intervention for adults with symptoms of depression. Behav Res Ther. 2015 Dec;75:20-31. PMID: 26523885. doi: 10.1016/j.brat.2015.10.005. | Relevant review article | RCT |
| [149], Birney AJ, Gunn R, Russell JK, Ary DV. MoodHacker mobile web app with email for adults to self-manage mild-to-moderate depression: Randomized controlled trial. JMIR Mhealth Uhealth. 2016 Jan 26;4(1):e8. PMID: [26813737](http://www.ncbi.nlm.nih.gov/pubmed/26813737). doi: [10.2196/mhealth.4231](https://doi.org/10.2196/mhealth.4231). | Relevant review article | RCT |
| [93], Pratap A, Renn BN, Volponi J, Mooney SD, Gazzaley A, Arean PA, et al. Using mobile apps to assess and treat depression in Hispanic and Latino populations: Fully remote randomized clinical trial. J Med Internet Res. 2018 Aug 9;20(8):e10130. PMID: 30093372. doi: 10.2196/10130. | Relevant review article | RCT |
| [98], Smith J, Newby JM, Burston N, Murphy MJ, Michael S, Mackenzie A, et al. Help from home for depression: A randomised controlled trial comparing internet-delivered cognitive behaviour therapy with bibliotherapy for depression. Internet Interv. 2017 Sep;9:25-37. PMID: 30135834. doi: 10.1016/j.invent.2017.05.001. | Relevant review article | RCT |
| [88], Moberg C, Niles A, Beermann D. Guided self-help works: Randomized waitlist controlled trial of pacifica, a mobile app integrating cognitive behavioral therapy and mindfulness for stress, anxiety, and depression. J Med Internet Res. 2019 Jun 8;21(6):e12556. PMID: 31199319. doi: 10.2196/12556. | Relevant review article | RCT |
| [91], Graham AK, Greene CJ, Kwasny MJ, Kaiser SM, Lieponis P, Powell T, et al. Coached mobile app platform for the treatment of depression and anxiety among primary care patients: A randomized clinical trial. JAMA Psychiatry. 2020 Sep 1;77(9):906-14. PMID: [32432695](http://www.ncbi.nlm.nih.gov/pubmed/32432695). doi: [10.1001/jamapsychiatry.2020.1011](https://doi.org/10.1001/jamapsychiatry.2020.1011). | Relevant review article | RCT |
| [101], Montero-Marin J, Araya R, Perez-Yus MC, Mayoral F, Gili M, Botella C, et al. An internet-based intervention for depression in primary care in Spain: A randomized controlled trial. J Med Internet Res. 2016 Aug 26;18(8):e231. PMID: [27565118](http://www.ncbi.nlm.nih.gov/pubmed/27565118). doi: [10.2196/jmir.5695](https://doi.org/10.2196/jmir.5695). | Relevant review article | RCT |
| [157], Andersson G, Hesser H, Veilord A, Svedling L, Andersson F, Sleman O, et al. Randomised controlled non-inferiority trial with 3-year follow-up of internet-delivered versus face-to-face group cognitive behavioural therapy for depression. J Affect Disord. 2013 Dec;151(3):986-94. PMID: 24035673. doi: 10.1016/j.jad.2013.08.022. | Relevant review article | RCT |
| [159], Choi I, Zou J, Titov N, Dear BF, Li S, Johnston L, et al. Culturally attuned internet treatment for depression amongst Chinese Australians: A randomised controlled trial. J Affect Disord. 2012 Feb;136(3):459-68. PMID: 22177742. doi: 10.1016/j.jad.2011.11.003. | Relevant review article | RCT |
| [111], Geraedts AS, Kleiboer AM, Twisk J, Wiezer NM, van Mechelen W, Cuijpers P. Long-term results of a web-based guided self-help intervention for employees with depressive symptoms: Randomized controlled trial. J Med Internet Res. 2014 Jul 9;16(7):e168. PMID: [25008127](http://www.ncbi.nlm.nih.gov/pubmed/25008127). doi: [10.2196/jmir.3539](https://doi.org/10.2196/jmir.3539). | Relevant review article | RCT |
| [107], Hallgren M, Kraepelien M, Ojehagen A, Lindefors N, Zeebari Z, Kaldo V, et al. Physical exercise and internet-based cognitive-behavioural therapy in the treatment of depression: Randomised controlled trial. Br J Psychiatry. 2015 Sep;207(3):227-34. PMID: 26089305. doi: 10.1192/bjp.bp.114.160101. | Relevant review article | RCT |

RCT, randomized controlled trial.
