# Supplementary material for: Assessing Patient Adherence to and Engagement With Digital Interventions for Depression in Clinical Trials: Systematic Literature Review
Source: J Med Internet Res. 2023 Aug 11;25:e43727. doi: 10.2196/43727 (PMC10457707; doi:10.2196/43727)
Supplement: Multimedia Appendix 4 [file jmir_v25i1e43727_app4.docx]

**Forbes et al. 2023 Multimedia Appendix 4: Table S4**

Table S4. Study characteristics of references included in this SLR.

| **Reference** | **Primary Diagnosis** | **Inclusion Test** | **Cutoff Score on Inclusion Test** | **Type of Digital Intervention** | **Primary Efficacy Outcome** | **Financial Incentive*** |
| --- | --- | --- | --- | --- | --- | --- |
| Littlewood et al 2015 [151] | Depression | PHQ-9 | > 10 | Web | PHQ-9 |  |
| Heim et al 2021 [74] | Depression | PHQ-9 | ≥ 10 | Both | PHQ-9, WHODAS 2.0 |  |
| Oehler et al 2020 [83] | Depression | PHQ-9 | 5–14 | Web | IDS-SR |  |
| Rahmadiana et al 2021 [71] | Depression | PHQ-9 | > 4 | Web | CSQ-8, SUS | Y |
| Salamanca-Sanabria et al 2020 [84] | Depression | PHQ-9 | 10–19 | Web | PHQ-9 |  |
| Fitzsimmons-Craft et al 2021 [131] | Depression | PHQ-9 | ≥ 5 | Both | Presence of mental health disorders at 2 years |  |
| Lukas et al 2021 [125] | Depression | PHQ-9 | ≥ 10 | App | NA |  |
| Kenter et al 2016 [99] | MDD | DSM-IV | Diagnosis of MDD | Web | CES-D |  |
| Quinonez-Freire et al 2021 [81] | MDD | MINI | Diagnosis of MDD | Web | BDI-II |  |
| Gili et al 2020 [85] | Depression | PHQ-9 | 5–14 | Web | Spanish version of PHQ-9 |  |
| Addington et al 2019 [134] | Depression | PHQ-8 | ≥ 5 | Web | PHQ-8, CES-D |  |
| Karyotaki et al 2019 [135] | Depression | PHQ-9 | > 4 and ≤ 14 | Web | PHQ-9, GAD-7 |  |
| Mira et al 2019 [136] | Depression | BDI-II | 14–28 | Web | BDI-II |  |
| Grafe et al 2019 [126] | Depression | PHQ-9 | 5–14 | Web | Health-related resource use,  use of medication, incapacity  to work |  |
| Kraepelien et al 2019 [137] | Depression | PHQ-9 | ≥ 10 | Web | MADRS‐S |  |
| Thase et al 2018 [91] | MDD | DSM-IV | Diagnosis of MDD | Web | HAMD |  |
| Grunzig et al 2018 [141] | Depression | CES-D | > 22 | Web | CES-D |  |
| Lobner et al 2018 [142] | Depression | PHQ-9 | 5–9 | Web | BDI-II, PHQ-9 |  |
| Fuhr et al 2018 [143] | Depression | PHQ-9 | 5–14 | Web | NA |  |
| Weisel et al 2018 [144] | MDD | MINI | Diagnosis of MDD | Web | QIDS-C |  |
| Gorges et al 2018 [89] | MDD | PHQ-9 | 5–14 | Web | German version of the CSQ-8,  ZUF-8 |  |
| Beevers et al 2017 [95] | Depression | QIDS | ≥ 10 | Web | QIDS-SR |  |
| Klein et al 2017 [96] | Depression | PHQ-9 | 5–14 | Web | PHQ-9 time to remission |  |
| Tomasino et al 2017 [94] | Depression | PHQ-8 | ≥ 8 | Web | Change in depression, use, self-reported usability, and coach time: PHQ-9, GAD-7, PROMIS,  SPS, SUS |  |
| Wilkinson et al 2017 [127] | Depression | Responded or remitted from MDE after ECT | Mean baseline MADRS score 14.7 | Web | NA |  |
| Brabyn et al 2016 [148] | Depression | PHQ-9 | > 10, and > 3 on question 9 | Web | PHQ-9 |  |
| Williams et al 2016 [147] | Depression | PHQ-9 | ≥ 10 | Web | NA |  |
| Meyer et al 2015 [102] | Depression | PHQ-9 | ≥ 15 | Web | PHQ-9 |  |
| Gilbody et al 2015 [103] | Depression | PHQ-9 | ≥ 10 | Web | PHQ-9 |  |
| Rickhi et al 2015 [150] | MDD | DSM-IV-TR and 40-70 on CDRS-R or 12-14 on HAMD | Diagnosis of MDD | Web | CDRS-R, HAMD |  |
| Hoifodt et al 2015 [104] | Depression | BDI-II | 10–40 | Web | NA |  |
| Schneider et al 2014 [109] | Depression | PHQ-9 | ≥10, with at least a 2 on specific questions | Web | WSAS |  |
| Kivi et al 2014 [110] | Depression | MINI and MADRS-S | Depression diagnosed with MINI and  MADRS-S < 35 | Web | BDI-II |  |
| Santucci et al 2014 [153] | Depression | PHQ-9 | ≥ 5 | Web | NA |  |
| Donkin et al 2013 [61] | Depression | Screening process to ensure current level of depressive symptoms | Not described | Web | PHQ-9 |  |
| Hoifodt et al 2013 [112] | Depression | BDI-II | 10–40 | Web | BDI-II |  |
| Boiler et al 2013 [114] | Depression | CES-D | 10–24 | Web | MHC-SF, WHO-5 |  |
| Moritz et al 2012 [63] | Depression | No specific depression cut-off | 97% had depression based on BDI | Web | BDI | Y |
| Levin et al 2011 [117] | Depression | DSM-IV | Depressed mood | CD | NA | Y |
| Berger et al 2011 [118] | Depression | BDI-II | > 13 | Web | BDI-II |  |
| Burns et al 2011 [116] | MDD | PHQ-8 and QIDS-C | PHQ-8 ≥ 8 and QIDS-C ≥ 10 | Both | NA |  |
| Mohr et al 2010 [119] | Depression | PHQ-8 | ≥ 10 | Web | NA |  |
| Titov et al 2010 [160] | MDD | PHQ-9 | ≥ 10 | Web | PHQ-9, BDI-II |  |
| Hatcher et 2018 [161] | Depression | Referred from mental health center with depression or dysthymia | On a waiting list for treatment in a psychiatric program | Web | PHQ-9 |  |
| Kramer et al 2021 [132] | MDD | MINI | Diagnosis of MDD or dysthymia | Web | BDI-II |  |
| Grinberg et al 2020 [76] | Depression | HDRS | ≥ 10 | App | NA | Y |
| Lopes et al 2020 [133] | MDD | DSM-V | Diagnosis of MDD or dysthymia | Web | PHQ-9 |  |
| Kelders et al 2013 [114] | Depression | CES-D | > 9 and < 39 | Web | NA |  |
| Mohr et al 2013 [62] | MDD | MINI and QIDS | Diagnosis of MDD on MINI and QIDS ≥ 11 | Web | PHQ-9 | Y |
| Kelders et al 2015 [105] | Depression | CES-D | > 9 and < 39 | Web | NA |  |
| Richards et al 2014 [154] | Depression | BDI-II | 14–29 | Web | BDI-II |  |
| Kordy et al 2016 [100] | MDD | DSM-IV | Diagnosis of MDD | Web | PSR of the LIFE |  |
| Richards et al 2018 [145] | Depression | PHQ-9 | ≥ 9 | Web | GAD-7, PHQ-9 |  |
| Motter et al 2019 [138] | Depression | HDRS | ≥ 10 | App | ADIS, HDRS, BDI-II, SDS, WAIS-IV, CWIT CNR, CWIT Inhibition, coding, letter fluency, Trails 2, Trails 4 | Y |
| Antle et al 2019 [139] | Depression | PHQ-9 | ≥ 10 | Web | CCBT program completion and satisfaction rates, CSQ-8,  PHQ-9, ATQ, CAQ, SWLS |  |
| Lambert et al 2018 [90] | Depression | PHQ-8 | ≥ 10 | Web | PHQ-9 |  |
| Graham et al 2020 [113] | Depression | PHQ-8 or GAD-7 | PHQ-8 ≥1 0 or GAD-7 ≥ 8 | App | GAD-7, PHQ-9 | Y |
| Montero-Marín et al 2016 [101] | MDD | BDI-II | 14–28 | Web | BDI-II |  |
| Andersson et al 2013 [157] | Depression | MADRS-S | 15–35 | Web | MADRS‐S |  |
| Choi et al 2012 [159] | MDD | DSM-IV | MDE | Web | CBDI, CB-PHQ-9 |  |
| Geraedts et al 2014 [108] | Depression | CES-D | ≥ 16 | Web | CES-D |  |
| Hallgren et al 2015 [107] | Depression | PHQ-9 | > 9 | Web | MADRS |  |
| Robertson et al 2006 [129] | Depression | Prior or current MDE and currently receiving treatment for depression | | Web | Adherence, depression severity (DSS, DASS), clinician and patient ratings of satisfaction |  |
| Warmerdam et al 2008 [124] | Depression | CES-D | ≥ 16 | Web | CES-D |  |
| Ruwaard et al 2009 [121] | Depression | BDI-IA | 10–29 | Web | BDI-IA, SCL-90-R, DEP |  |
| Perini et al 2009 [122] | Depression | PHQ-9 and MINI | 23 > PHQ-9 > 5, MINI depression | Web | BDI-II, PHQ-9 |  |
| de Graaf et al 2009 [123] | Depression | BDI-II | ≥ 16 | Web | BDI-II |  |
| Vernmark et al 2010 [120] | MDD | DSM-IV and MADRS-S | Diagnosis of MDD and 31 > MADRS-S > 14 | Web | BDI-II |  |
| Johansson et al 2012 [158] | Depression | MADRS-S | > 14 and < 36 | Web | BDI |  |
| Watts et al 2013 [156] | Depression | PHQ-9 | Mild to moderate | App | PHQ-9 |  |
| Williams et al 2013 [115] | Depression | MINI | MDE | Web | BDI-II, PHQ-9, K10 |  |
| Wagner et al 2014 [155] | Depression | BDI-II | ≥ 12 | Web | BDI-II |  |
| Roepke et al 2015 [152] | Depression | CES-D | ≥ 16 | Both | CES-D |  |
| Richards et al 2015 [106] | Depression | BDI-II | 14–29 | Web | BDI-II |  |
| Birney et al 2016 [149] | Depression | PHQ-9 | 10–19 | Both | PHQ-9 | Y |
| Anguera et al 2016 [128] | Depression | PHQ-9 | ≥ 5 overall or ≥ 2 on item 10 | App | NA | Y |
| Mohr et al 2017 [97] | Depression | PHQ-9 | ≥ 10 | App | PHQ-9, GAD-7 | Y |
| Mehrotra et al 2018 [146] | Depression | BDI-II | ≥ 13 | Web | BDI-II, PHQ-9, WSAS, GAF |  |
| Pratap et al 2018 [93] | Depression | PHQ-9 | ≥ 5 overall or ≥ 2 on item 10 | App | PHQ-9, SDS | Y |
| Smith et al 2017 [198] | MDD | MINI and PHQ-9 | Diagnosis of MDD and PHQ-9 5–23 | Web | PHQ-9 |  |
| Stiles-Shields et al 2019 [64] | Depression | PHQ-9 | ≥ 10 | App | PHQ-9 |  |
| Dahne et al 2019 [140] | Depression | PHQ-8 | ≥ 10 | App | BDI-II | Y |
| Moberg et al 2019 [88] | Depression | PHQ-8 | 5–14 | App | DASS-21, PHQ-8, GAD-7, General Self-Efficacy Scale |  |
| Lukas et al 2021 [82] | Depression | PHQ-9 | ≥ 5 | App | PHQ-9 |  |
| Caplan et al 2019 [92] | Depression | PHQ-9 | 15 > PHQ-9 ≥ 5 | App | PHQ-9 |  |
| Batterham et al 2021 [78] | Depression | PHQ-9 | 5–14 | Web | Uptake, adherence | Y |
| Bur et al 2022 [75] | Depression | PHQ-9 | 5–14 | Web | PHQ-9 |  |
| Perez et al 2021 [79] | MDD | MINI | MDE | Web | BDI-IA |  |
| Karyotaki et al 2022 [72] | Depression | PHQ-9 | > 4 | Web | PHQ-9, GAD-7 |  |
| Kramer et al 2021 [77] | Depression | CES-D | > 22 | Web | CES-D |  |
| Kramer et al 2022 [130] | Depression | PHQ-9 | ≥ 10 | Web | QID-C | Y |
| Moskowitz et al 2021 [80] | Depression | PHQ-8 | ≥ 5 | Web | Adherence |  |
| Wright et al 2022 [73] | Depression | PHQ-9 | ≥ 10 | Web | PHQ-9 |  |

* Financial incentives ranged from $11.30 per assessment to $250.

ADIS, Anxiety Disorders Interview Schedule for DSM-IV; App, application; ATQ, Automatic Thoughts Questionnaire; BDI-IA, Beck Depression Inventory-Spanish version, BDI-II, Beck Depression Inventory-II; CAQ, Computer Attitudes Questionnaire; CB-PHQ-9, Chinese version of the Patient Health Questionnaire-9 item; CBDI, Chinese version of the Beck Depression Inventory; CCBT, computerized cognitive behavioral therapy; CDRS-R, Children’s Depression Rating Scale Revised; CES-D, Center for Epidemiological Studies Depression scale; CSQ-8, 8-item Client Satisfaction Questionnaire; CWIT CNR, Color-Word Interference Test: Combined Naming and Reading scale; DASS, Depression, Anxiety and Stress Scale; DSM-IV-TR/V, Diagnostic and Statistical Manual of Mental Disorders-Fourth Edition-Text Revision/Fifth Edition; DSS, Depression Severity Scale; ECT, electroconvulsive therapy; GAD-7, Generalized Anxiety Disorder scale-7; GAF, Global Assessment of Functioning; HAMD, Hamilton Rating Scale for Depression; HDRS, Hamilton Depression Rating Scale; IDS-SR, Inventory of Depressive Symptomatology–self-rating; K10, 10-item Kessler Psychological Distress Scale; LIFE, Longitudinal Interval Follow-Up Evaluation; MADRS-S, Montgomery-Åsberg Depression Rating Scale–self-rated; MDD, major depressive disorder; MDE, major depressive episode; MHC-SF, Mental Health Continuum-Short Form; MINI, Mini International Neuropsychiatric Interview; NA, not available; PHQ-8/9, Patient Health Questionnaire-8/9; PROMIS, Patient Reported Outcome Measurement Information System; PSR, Psychiatric Status Rating; QIDS-C, Quick Inventory of Depressive Symptoms–Clinician Rating; QIDS-SR, Quick Inventory of Depressive Symptoms–Self-Report; SCL-90-R DEP, Depression scale of the Symptom Checklist-90-Revised; SDS, Sheehan Disability Scale; SPS, Social Provisions Scale; SUS, System Usability Scale; SWLS, Satisfaction with Life Scale; WAIS-IV, Wechsler Adult Intelligence Scale; WHO-5, World Health Organization Wellbeing Index; WHODAS 2.0, World Health Organization Disability Assessment Schedule 2.0; WSAS, Work and Social Adjustment Scale; Y, yes; ZUF-8, Patient Satisfaction Questionnaire (German version).
