# Supplementary material for: Assessing Patient Adherence to and Engagement With Digital Interventions for Depression in Clinical Trials: Systematic Literature Review
Source: J Med Internet Res. 2023 Aug 11;25:e43727. doi: 10.2196/43727 (PMC10457707; doi:10.2196/43727)
Supplement: Multimedia Appendix 5 [file jmir_v25i1e43727_app5.docx]

**Forbes et al. 2023 Multimedia Appendix 5: Figures**

Figure S1. Publication year for web-based and app-based studies. The published studies of app-based interventions for depression are more recent than the studies of web-based interventions.


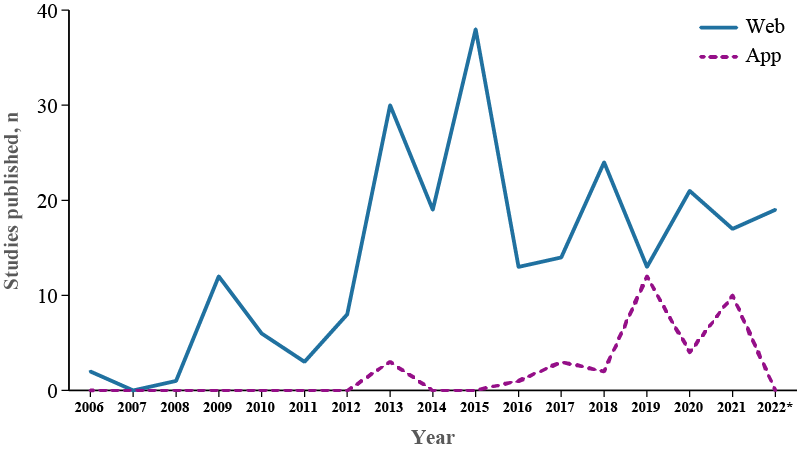


*January 1, 2022 to April 15, 2022
n: number of studies.

Figure S2. Tools used by multiple studies to assess depression.


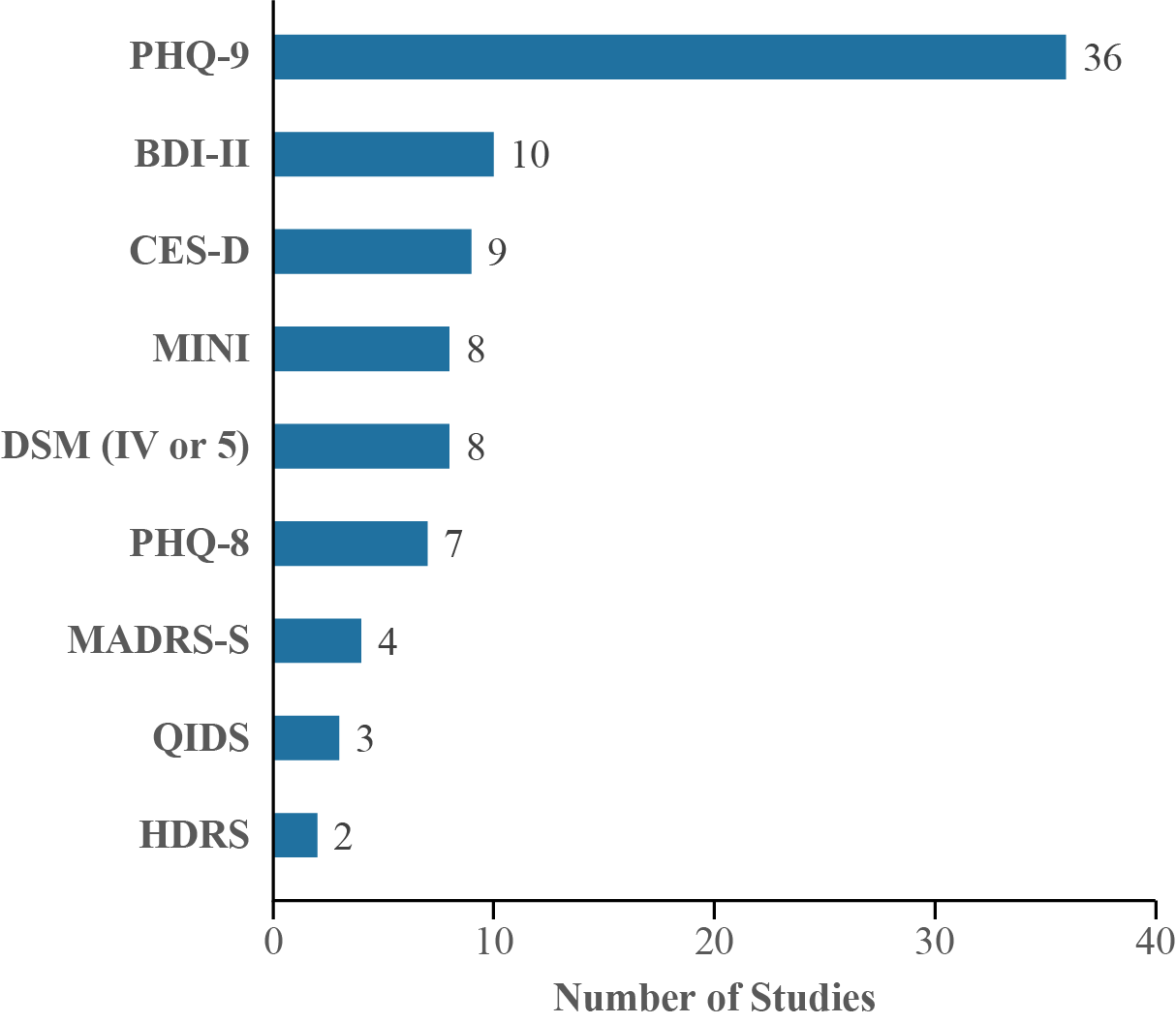


BDI: Beck Depression Inventory; CES-D: Center for Epidemiologic Studies Depression Scale; DSM: Diagnostic and Statistical Manual of Mental Disorders; HDRS: Hamilton Depression Rating Scale; MADRS-S: Montgomery-Asberg Depression Rating Scale-Self Assessment; MINI: Mini International Neuropsychiatric Interview; PHQ: Patient Health Questionnaire; QIDS: Quick Inventory of Depressive Symptomatology.

Figure S3. Most common metrics used to measure adherence and engagement.


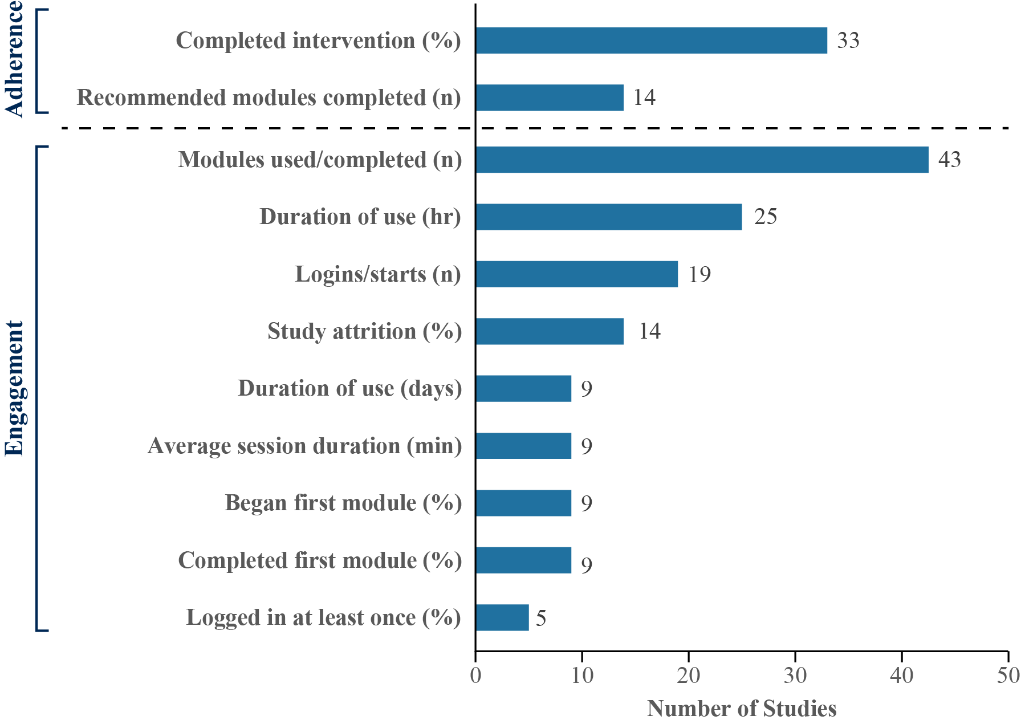


hr: hours; min: minutes; n: number.


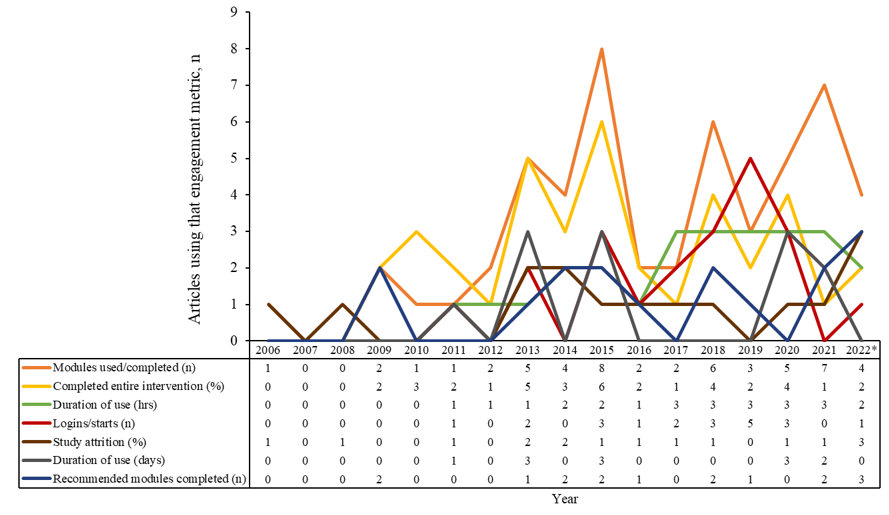
Figure S4. Use of engagement metrics over time. Number of modules used and attrition from the study were the earliest terms used by the studies reviewed here.

*January 1, 2022 to April 15, 2022
hrs: hours; n: number of studies.

Figure S5. Commonly used efficacy outcomes.


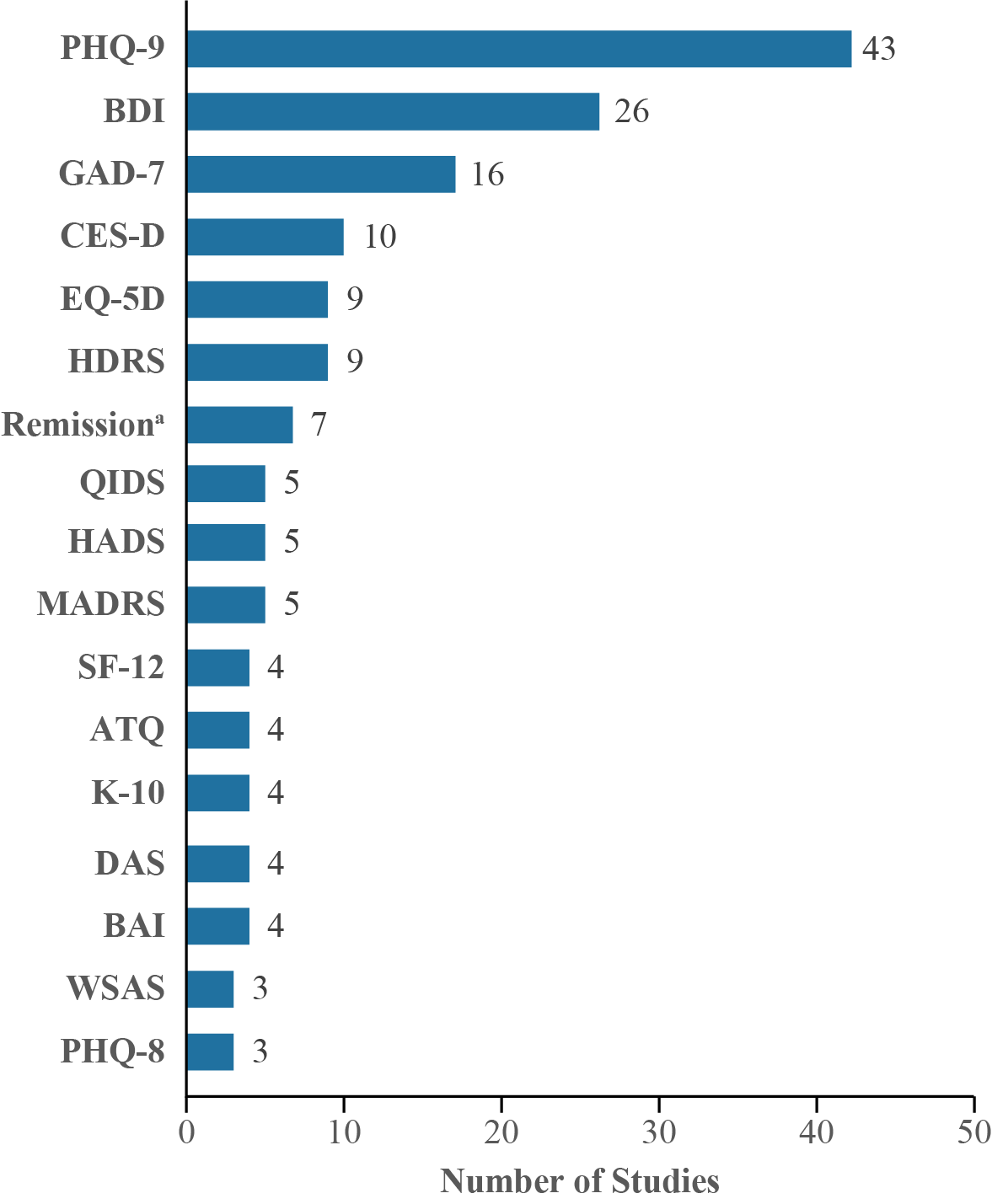


^a^Remission was defined as an Hamilton Depression Rating Scale (HDRS) score ≤7 in 2 studies, a Patient Health Questionnaire-9 (PHQ-9) score of <5 in 2 studies, episode remission (with no further description) in 2 studies, and no longer fulfilling diagnostic criteria for a major depressive episode according to the Structured Clinical Interview for DSM-5 Disorders (SCID-5-CV) in 1 study.

ATQ: Automatic Thoughts Questionnaire; BAI: Beck Anxiety Inventory; BDI: Beck Depression Inventory; CES-D: Center for Epidemiologic Studies Depression Scale; DAS: Dysfunctional Attitude Scale; EQ-5D: EuroQol- 5 Dimension; GAD-7: General Anxiety Disorder-7; HDRS: Hamilton Depression Rating Scale; HADS: Hospital Anxiety and Depression Scale; K-10: Kessler Psychological Distress Scale; MADRS: Montgomery-Asberg Depression Rating Scale; PHQ: Patient Health Questionnaire; QIDS: Quick Inventory of Depressive Symptomatology; SCID-5-CV: Structured Clinical Interview for DSM-5 Disorders; SF-12: 12-Item Short Form Survey; WSAS: Work and Social Adjustment Scale.

Figure S6. Days given to use the digital intervention versus hrs patients spent engaging with intervention. Across the studies, patients engaged with the intervention more when given a longer period of time to use the intervention (r = 0.56, *P =* .012).


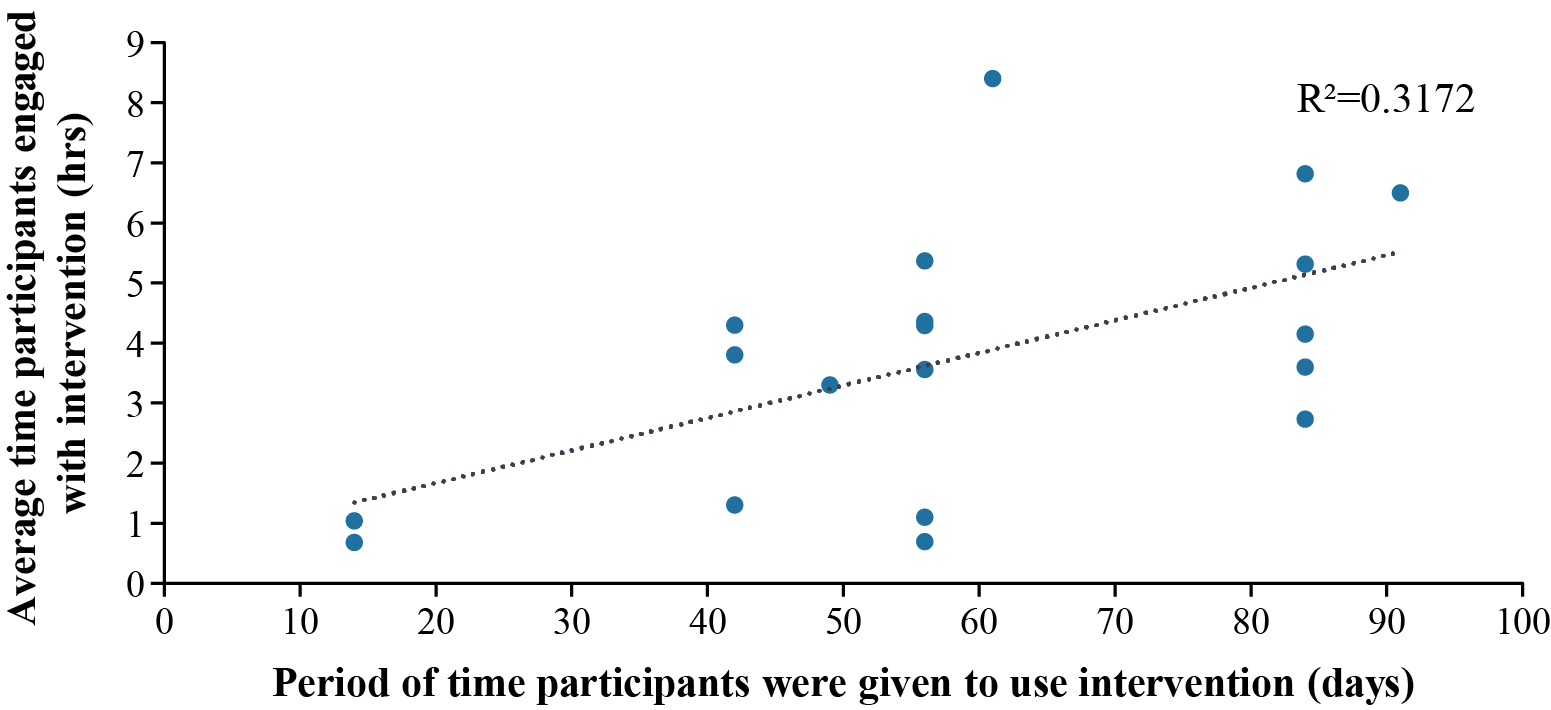


hrs: hours.
